# Supplementary material for: Deficits in Prenatal Serine Biosynthesis Underlie the Mitochondrial Dysfunction Associated with the Autism-Linked FMR1 Gene
Source: Int J Mol Sci. 2021 May 30;22(11):5886. doi: 10.3390/ijms22115886 (PMC8198117; doi:10.3390/ijms22115886)
Supplement: Supplementary file 1 [file ijms-22-05886-s001.zip › ijms-1214785-supplementary.pdf]

# Supplementary Material

This supplement has been provided by the authors to give readers additional information about their work.

Supplement to "***Deficits in prenatal serine biosynthesis underlie the mitochondrial dysfunction associated with the autism-linked FMR1 gene"*** by Nolin S., Napoli E., Flores A., Hagerman R.J., and Giulivi C***.***

**Table of contents**


| | |
|---|---|
| Supplementary Methods | 3 |
| Supplementary Tables | 4 |
| Supplementary Figures | 8 |
| Supplementary References | 18 |
| Supplementary Dataset 1 | 27 |
| Supplementary Dataset 2 | 75 |

## Supplementary Methods

### *Metabolomics and proteomics*

For metabolomic analysis of AF, plasma biosamples (AF or plasma) or UCF, aliquots were extracted by 1 ml of degassed acetonitrile:isopropanol:water (3:3:2, V/V/V) at -20°C, centrifuged and decanted with subsequent evaporation of the solvent to complete dryness. After treatment with acetonitrile/water (1:1), to remove membrane lipids and triglycerides, the supernatant was dried down a second time. Internal standards C08-C30 FAMEs were added and the samples derivatized with methoxyamine hydrochloride in pyridine and subsequently with *N*-methyl-*N*-trimethylsilyltrifluoroacetamide for trimethylsilylation of acidic protons. Data were acquired using the chromatographic parameters previously reported in detail [1, 2]. Metabolites were identified by matching ion chromatographic retention index, accurate mass, and mass spectral fragmentation signatures against library entries created from authentic standard metabolites obtained with the identical analytical procedure as the experimental samples. For proteomics analysis, total proteins from either AF samples or dermal fibroblasts were precipitated by the addition of 4 volumes of -20°C acetone as described before in detail [3]. All samples were submitted to the UC Davis Mass Spectrometry Core Facility, where this analysis was performed essentially as described before [4, 5].

### *Validation cohorts characteristics and analyses*

Two external validation cohorts were identified constituted by dermal fibroblasts and plasma obtained from subjects recruited at the MIND Institute, Sacramento, CA. Demographic details of donors are reported in **Supplementary Table S2**. Twenty-five primary dermal fibroblasts were obtained from either carriers of the premutation (n = 14) or controls (n = 11; **Supplementary Table S2**). Their age (average ± SEM) was 46 ± 17 y (24-67 y old) for controls, and 50 ± 16 y (24-71 y old) for premutation carriers, a M-to F ratio of 1.3 with no differences between the two diagnostic groups. From the carriers (n = 14), 6 had FXTAS. The other external validation cohort was constituted by plasma samples from (non-carriers) controls (n = 16) and carriers (n = 23). [6] Their age (average ± SEM) was 37 ± 13 for controls (ranging from 24 to 57 y old), and 37 ± 19 y (ranging from 8 to 63 y old) for premutation carriers, with a M-to-F ratio of 1 and no differences between the two diagnostic groups. From the carriers (n = 23), only 4 subjects were diagnosed with FXTAS. These external validation cohorts (to prevent bias-corrected estimates of model performance [7]) were chosen based on their availability on proteomics and metabolomics. One was constituted by proteomics data from 25 dermal fibroblasts obtained through the MIND Institute, Sacramento, CA (**Supplementary Table S2**). The other one was constituted by plasma metabolomics obtained from non-carriers and carriers (**Supplementary Table S2**) obtained at the same Institution [6]. The ROC analyses were performed by utilizing MetaboAnalyst [8]. The ROC curves with AF data were generated by Monte-Carlo cross validation (MCCV) using balanced sub-sampling. In each MCCV, two thirds (2/3) of the samples were used to evaluate the feature importance. The top 2, 3, 5, 10 ...100 (max) important features were then used to build the classification model which is validated on the 1/3 the (randomly selected) samples that were left out. The procedure was repeated multiple times to calculate the performance and confidence interval of the model. We utilized the SVM linear method for both the classification and the feature ranking method. As indicate din the text, two external cohorts were used to validate the model by utilizing the hold-out feature within the analysis. The predictive performance of each model was then quantified by measures of discrimination. We determined discrimination by the area under the receiver operating characteristic curve (AUROC) with 95% confidence interval (CI). Discrimination is the ability of the model to correctly separate samples from subjects who develop FXTAS (or associated phenotype) from those who will not. For comparability of the models, we used the validation cohort with our inclusion and exclusion criteria. A sensitivity analysis was performed to assess the predictive performance of each model according to its additionally defined eligibility criteria. The potential clinical utility was evaluated for the best discriminative models by means of decision curve analysis. Decision curve analysis provides insight into the net benefit (net proportion of true positives) of the models over a range of threshold risks as opposed to designating all or no women at high risk of having a child who may develop FXTAS [9, 10]. Finally, we composed tables for the models with the highest net benefit comparing sensitivity, specificity, and positive and negative predictive values for different risk thresholds.

## Supplementary Tables

**Table S1.** Proteins identified in this and another study utilizing AF

| Protein name | Gene official name | This study | Cho et al. |
|---|---|---|---|
| | | Rank | |
| Albumin | ALB | 1 | 1 |
| Immunoglobulins* | | 2 | 2 |
| Alpha-1-antitrypsin | SERPRINA1 | 3 | 6 |
| Serotransferrin | TF | 4 | 4 |
| Fibronectin 1 isoform 1 | FN1 | 5 | 3 |
| Vitamin D-binding protein | GC | 6 | 9 |
| Insulin growth factor binding protein 1 | IGFBP1 | 7 | |
| Alpha-fetoprotein | AFP | 8 | 8 |
| Complement C3 | C3 | 9 | 5 |
| Ceruloplasmin | CP | 10 | 7 |
| AMBP | AMBP | 11 | 14 |
| Apolipoprotein A1 | APOA1 | 12 | 11 |
| Alpha-2_HS-glycoprotein | AHSG | 13 | |
| Plasminogen | PLG | 14 | 15 |
| ORM1-like protein | ORM1 | 15 | |
| Transforming growth factor-beta-induced protein ig-h3 | TGFBI | 20 | 13 |
| Antithrombin III | SERPINC1 | 26 | 12 |
| Periostin | POSTN | 37 | 10 |

*Collectively, immunoglobulin chains. Of the proteins identified by mass spectrometry in our study, 12 (in gray) matched the top 15 most abundant ones in AF samples as reported by Cho et al [11]. Rank is abundance with 1 as the most abundant protein group in each set.

**Table S2.** Demographics of the fibroblasts' donors included in the present study

| Clinical Group | Age (y) | Sex | Diagnosis | Outcome |
|---|---|---|---|---|
| C1 | 23 | F | Control | Metabolomics |
| C2 | 24 | F | Control | Metabolomics |
| C3 | 24 | M | Control | Metabolomics, Proteomics |
| C4 | 25 | F | Control | Metabolomics |
| C5 | 26 | F | Control | Metabolomics, Proteomics |
| C6 | 26 | M | Control | Metabolomics |
| C7 | 29 | F | Control | Metabolomics |
| C8 | 29 | F | Control | Metabolomics, Proteomics |
| *C9 | 34 | F | Control | Proteomics |
| *C10 | 39 | F | Control | Proteomics |
| *C11 | 40 | F | Control | Proteomics |
| C12 | 41 | M | Control | Metabolomics |
| C13 | 42 | M | Control | Metabolomics |
| C14 | 45 | F | Control | Metabolomics |
| C15 | 51 | M | Control | Metabolomics |
| C16 | 54 | M | Control | Metabolomics |
| C17 | 54 | M | Control | Metabolomics |
| C18 | 57 | F | Control | Metabolomics |
| C19 | 59 | M | Control | Proteomics |
| C20 | 60 | F | Control | Metabolomics |
| C21 | 61 | M | Control | Proteomics |
| C22 | 62 | M | Control | Proteomics |
| C23 | 63 | M | Control | Proteomics |
| C24 | 67 | M | Control | Proteomics |
| P1 | 8 | M | Premutation | Metabolomics |
| P2 | 8 | M | Premutation | Metabolomics |
| P3 | 9 | M | Premutation | Metabolomics |
| P4 | 10 | F | Premutation | Metabolomics |
| P5 | 17 | F | Premutation | Metabolomics |
| P6 | 20 | M | Premutation | Metabolomics |
| P7 | 24 | F | Premutation | Metabolomics, Proteomics |
| P8 | 24 | F | Premutation | Metabolomics, Proteomics |
| P9 | 25 | M | Premutation | Metabolomics |
| P10 | 33 | F | Premutation | Proteomics |
| P11 | 38 | F | Premutation | Metabolomics, Proteomics |
| P12 | 43 | F | Premutation | Metabolomics, Proteomics |
| P13 | 45 | M | Premutation | Metabolomics |
| P14 | 46 | M | Premutation | Metabolomics |
| P15 | 49 | F | Premutation | Metabolomics |
| P16 | 50 | F | Premutation | Metabolomics, Proteomics |
| P17 | 50 | M | Premutation | Proteomics |
| P18 | 52 | M | Premutation | Metabolomics, Proteomics |
| P19 | 53 | F | Premutation | Metabolomics |
| P20 | 55 | F | Premutation | Metabolomics |
| P21 | 56 | M | Premutation | Metabolomics |
| P22 | 58 | M | FXTAS | Metabolomics, Proteomics |
| P23 | 59 | F | FXTAS | Metabolomics |
| P24 | 60 | M | FXTAS | Proteomics |
| P25 | 61 | M | FXTAS | Metabolomics |
| P26 | 62 | M | FXTAS | Proteomics |
| P27 | 63 | M | FXTAS | Metabolomics |
| P28 | 67 | M | FXTAS | Proteomics |
| P29 | 69 | M | FXTAS | Proteomics |
| P30 | 71 | M | FXTAS | Proteomics |

*Cell lines obtained from Coriell repository. All other samples (fibroblasts and plasmas) were obtained from participants previously enrolled in Genotype/Phenotype study [6].

**Table S3.** Human disorders associated with premutation-linked proteins and metabolic patterns

| ID | LABEL | Degree | Betweeness |
|---|---|---|---|
| **181500** | Schizophrenia | 31 | 14381.05 |
| **104300** | Alzheimer Disease | 18 | 2604.56 |
| *616299* | ***Lipoyltransferase 1 Deficiency*** | ***12*** | ***1389.02*** |
| **211980** | Lung Cancer | 11 | 2549.07 |
| **246900** | *Dihydrolipoamide Dehydrogenase Deficiency* | 7 | 414.13 |
| **271900** | Canavan Disease | 6 | 723.64 |
| **607196** | Microcephaly, Amish Type | 6 | 264.28 |
| *203740* | ***Alpha-Ketoglutarate Dehydrogenase Deficiency*** | ***5*** | ***238.93*** |
| **606054** | Propionic Acidemia | 4 | 374.73 |
| **266150** | Pyruvate Carboxylase Deficiency | 4 | 322.24 |
| **220500** | Deafness, Onychodystrophy, Osteodystrophy, Mental Retardation, And Seizures Syndrome | 4 | 135.69 |
| **248600** | Maple Syrup Urine Disease | 4 | 31.68 |
| **605911** | 3-Hydroxy-3-Methylglutaryl-Coa Synthase-2 Deficiency | 3 | 977.07 |
| *245450* | ***Lactic Aciduria Due To D-Lactic Acid*** | ***3*** | ***841.85*** |
| *600721* | ***D-2-Hydroxyglutaric Aciduria 1*** | ***3*** | ***710.56*** |
| **126200** | Multiple Sclerosis, Susceptibility To | 3 | 552.23 |
| **125853** | Diabetes Mellitus, Noninsulin-Dependent | 3 | 523.49 |
| **145500** | Hypertension, Essential | 3 | 521.6 |
| **608611** | Ribose 5-Phosphate Isomerase Deficiency | 3 | 473 |
| **246450** | 3-Hydroxy-3-Methylglutaryl-Coa Lyase Deficiency | 3 | 433.46 |
| **237300** | Carbamoyl Phosphate Synthetase I Deficiency, Due to Hyperammonemia | 3 | 207.25 |
| **614741** | Mitochondrial Pyruvate Carrier Deficiency | 3 | 148.09 |
| **242600** | Iminoglycinuria | 3 | 138.39 |
| **252150** | Molybdenum Cofactor Deficiency, Complementation Group A | 3 | 123.31 |
| **557000** | Pearson Marrow-Pancreas Syndrome | 3 | 110.55 |
| **605814** | Citrullinemia, Type Ii, Neonatal-Onset | 3 | 109.97 |
| **261680** | Phosphoenolpyruvate Carboxykinase Deficiency, Cytosolic | 3 | 108.9 |
| **276700** | Tyrosinemia, Type I | 3 | 64.83 |
| **617290** | Epilepsy, Early-Onset, Vitamin B6-Dependent | 3 | 32.1 |
| **222730** | Dicarboxylic Aminoaciduria | 3 | 26.95 |
| **137215** | Gastric Cancer, Hereditary Diffuse | 2 | 705 |
| **105200** | Amyloidosis, Familial Visceral | 2 | 472 |
| **167320** | Inclusion Body Myopathy Early Onset Paget Disease with or without Frontotemporal Dementia 1 | 2 | 401.11 |
| **220110** | Mitochondrial Complex Iv Deficiency | 2 | 286.15 |
| **614105** | Methylmalonate Semialdehyde Dehydrogenase Deficiency | 2 | 237 |
| **614462** | Hyperglycinemia, Lactic Acidosis, And Seizures | 2 | 147.94 |
| **229600** | Fructose Intolerance, Hereditary | 2 | 136.85 |
| **229700** | Fructose-1,6-Bisphosphatase Deficiency | 2 | 136.85 |
| **612736** | Cerebral Creatine Deficiency Syndrome 2 | 2 | 136.85 |
| **616034** | 2,4-Dienoyl-Coa Reductase Deficiency | 2 | 115.5 |
| **600462** | Myopathy, Lactic Acidosis, And Sideroblastic Anemia 1 | 2 | 55.38 |
| **614300** | Hypermethioninemia Due to Adenosine Kinase Deficiency | 2 | 47.88 |
| **608782** | Pyruvate Dehydrogenase Phosphatase Deficiency | 2 | 38.69 |
| **614654** | Coenzyme Q10 Deficiency, Primary, 5 | 2 | 38.69 |
| **278300** | Xanthinuria, Type I | 2 | 27.18 |

| ID | LABEL | Degree | Betweeness |
|---|---|---|---|
| **300322** | Lesch-Nyhan Syndrome | 2 | 27.18 |
| **300661** | Phosphoribosylpyrophosphate Synthetase Superactivity | 2 | 27.18 |
| **613752** | Hypermethioninemia with S-Adenosylhomocysteine Hydrolase Deficiency | 2 | 27.17 |
| **312170** | Pyruvate Dehydrogenase E1-Alpha Deficiency | 2 | 17.6 |
| **266130** | Glutathione Synthetase Deficiency | 2 | 17.57 |
| **219150** | Cutis Laxa, Autosomal Recessive, Type Iiia | 2 | 16.08 |
| **606812** | Fumarase Deficiency | 2 | 4.58 |
| **256520** | Neu-Laxova Syndrome 1 | 2 | 2.32 |
| *601815* | ***Phosphoglycerate Dehydrogenase Deficiency*** | ***2*** | ***2.32*** |
| **606904** | Epilepsy, Myoclonic Juvenile | 2 | 2.32 |
| *610992* | ***Phosphoserine Aminotransferase Deficiency*** | ***2*** | ***2.32*** |
| *614023* | ***Phosphoserine Phosphatase Deficiency*** | ***2*** | ***2.32*** |
| **614923** | Branched-Chain Keto Acid Dehydrogenase Kinase Deficiency | 2 | 0.92 |

The combined omics dataset was analyzed against databases of human diseases. Schizophrenia and Alzheimer’s disease were identified, along with lipolyltransferase deficiency, AKG deficiency, amyloidosis as well as diseases associated with altered Ser biosynthesis. In bold, diseases discussed in the text.

## Supplementary Figures

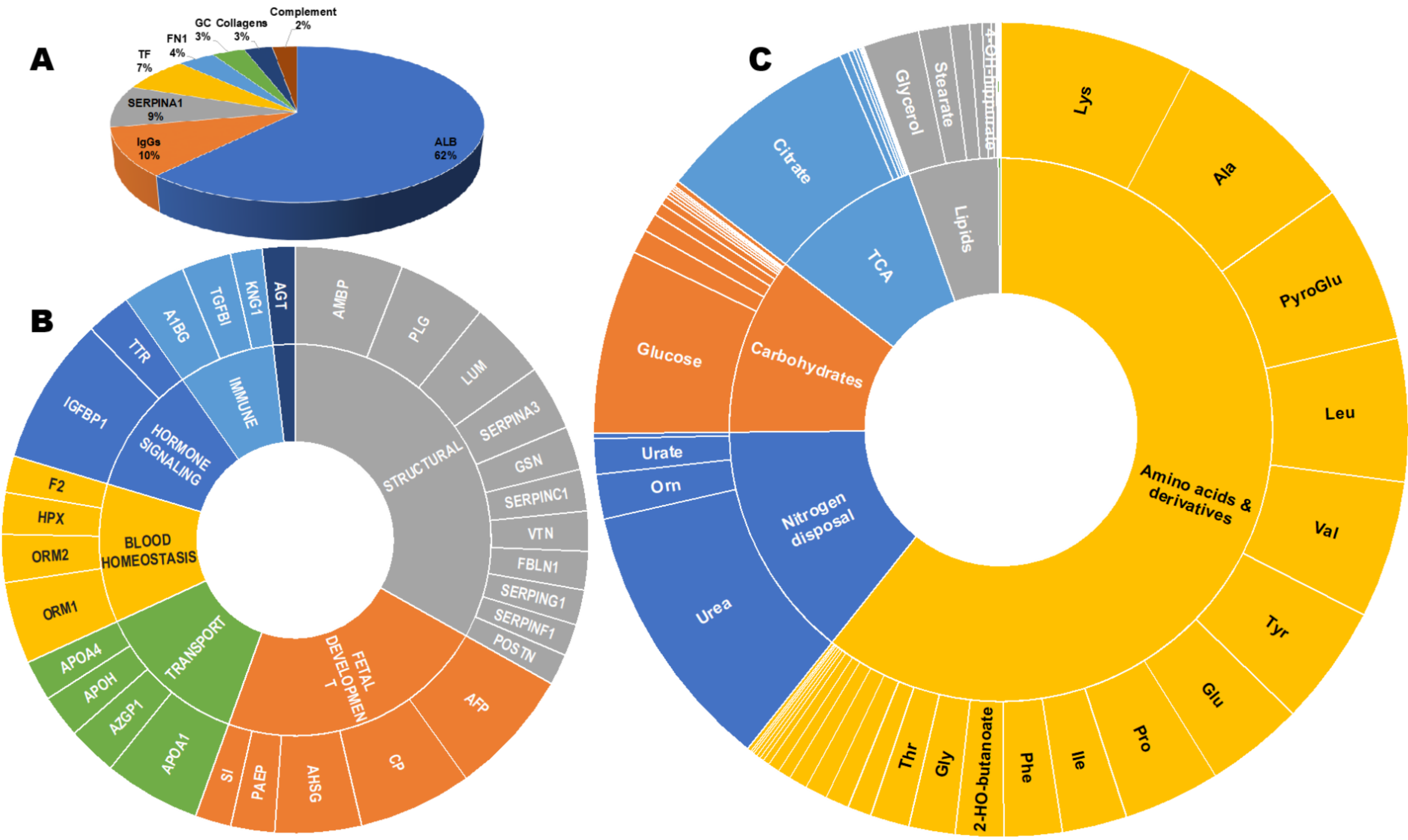


**Figure S1. Identification of the most abundant proteins and metabolites in AF. A.** A total of 423 unique proteins were identified by mass spectrometry in the AF supernatant samples. Albumin, immunoglobulins, SERPINA1, transferrin, fibronectin, vitamin D3 binding protein, collagens (all isoforms) and complement subunits were the most abundant proteins identified (with a relative abundance of ≥ 2%). **B.** Analysis performed with the gene ontology molecular function feature. A significant fraction of the proteome was represented by proteins necessary for fetal development including structural ones. The rest were involved in blood homeostasis, hormone signaling, immune response and other functions. **C.** Of the total 129 metabolites identified, the largest category was represented by amino acids and derivatives, followed by carbohydrates and nitrogen disposal, tricarboxylic acid cycle (TCA) and lipids.

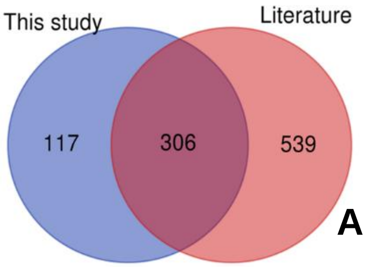


A

**Figure S2. Comparison of proteomic data from this study and those from other studies utilizing AF.** Proteomic data were collected from >150 studies [11-164], compared to those obtained in our study, and visualized by using a Venn diagram (**A**). List of proteins from AF supernatants and literature is reported in **B**.

B

| Literature AND This study (306) | | | | | | This study (117) | | Literature (539) | | | | | | | | | |
|---|---|---|---|---|---|---|---|---|---|---|---|---|---|---|---|---|---|
| elements | | | | | | elements | | elements | | | | | | | | | |
| A1BG | CDH11 | F11R | KNG1 | PRDX2 | TNXB | A2ML1 | IGKV3D-11 | ABAT | C1QB | CTHRC1 | FCGR3A | HIST1H4 | LRRC19 | P4HA1 | PSMC5 | SOD1 | VDAC3 |
| A2M | CDH13 | F12 | KRT1 | PRDX6 | TPI1 | ACE | IGKV4-1 | ABP1 | CA2 | CTNNA1 | FCGR3B | HIST1H4E | LTBP4 | P4HA2 | PSMC6 | SOD2 | VIL2 |
| ABI3BP | CDH5 | F13B | KRT19 | PRG2 | TTR | ADAMTS2 | IGLC7 | ABRA | CALD1 | CTSB | FDXR | HIST2H3C | LTF | PA2G4 | PSMD11 | SPAG9 | VPS4B |
| ACE2 | CEL | F2 | KRT2 | PRL | TXN | AGRN | IGLL5 | ACAA2 | CALM2 | CTSD | FES | HNRNPA1 | MAT2A | PAK2 | PSMD13 | SRSF1 | WARS |
| ACTB | CFB | FABP1 | KRT8 | PROCR | VASN | AHSP | IGLV1-47 | ACADM | CAP1 | CTSG | FH | HNRNPA2B1 | MATN3 | PAPSS1 | PSME1 | SSB | WDR1 |
| ACTG2 | CFD | FAM3C | KRT9 | PROS1 | VIM | ANXA13 | IGLV1-51 | ACAT1 | CAPG | CTSL | FHL2 | HNRNPC | MATN4 | PBEF1 | PTK7 | ST13 | XLKD1 |
| ACTN4 | CFH | FBLN1 | LAMC1 | PRSS8 | VTN | AOC1 | IGLV10-54 | ACLY | CAPZA1 | CTSL1 | FK506 | HNRNPCL1 | MCCC2 | PCBP1 | PTPN23 | STCH | XRCC5 |
| ADAMTS1 | CFHR1 | FBLN2 | LBP | PSAP | WFDC2 | APOB | IGLV2-11 | ACO1 | CAPZA2 | CTSZ | FKBP10 | HNRNPH1 | MDH1 | PCCA | PVRL2 | STIP1 | YWHAB |
| AFM | CFHR2 | FBN1 | LCN2 | PSG9 | YWHAE | C1RL | IGLV2-8 | ACO2 | CAPZB | CXCL10 | FKBP1A | HNRNPH2 | MDH2 | PCK2 | PXDN | STRAP | YWHAQ |
| AFP | CFI | FCGBP | LCP1 | PTGDS | YWHAG | C4B | IGLV3-10 | ACOT2 | CASP14 | CXCL11 | FKBP4 | HNRNPK | MDN1 | PCNA | PYCR1 | SUCLG1 | ZBTB33 |
| AGT | CFL1 | FETUB | LDHA | PVR | YWHAZ | CD9 | IGLV3-19 | ACOT9 | CAT | CXCL8 | FLNA | HNRNPL | ME1 | PDHA1 | RAB7A | SULF2 | ZYX |
| AHSG | CHGA | FGA | LDHB | QSOX1 | | CDC5L | IGLV3-21 | ACTA1 | CAVIN1 | CXCL9 | FNDC1 | HNRPA1 | METAP1 | PDHB | RAC2 | SUSD5 | |
| ALB | CHRDL1 | FGB | LGALS1 | RBP4 | | CDH15 | IGSF1 | ACTA2 | CCT2 | CYCS | FOLR1 | HNRPA2B1 | MICAL2 | PDIA4 | RACK1 | SYCP3 | |
| AMBP | CLCA1 | FGG | LGALS3BP | RCN1 | | CDHR5 | IL1R2 | ACTG1 | CCT3 | DAAM2 | FOLR2 | HPRP | MMP8 | PDIA6 | RAN | TAF1C | |
| ANGPTL2 | CLEC3B | FLT1 | LMAN2 | RNASE1 | | CELA3A | IL1RAP | ACTN1 | CCT4 | DBN1 | FRZB | HSD17B10 | MMP9 | PDLIM1 | RANBP1 | TAF3 | |
| ANXA1 | CLIC1 | FMOD | LOX | S100A11 | | CGA | INA | ACTR1A | CCT5 | DCTN2 | FSCN1 | HSD17B4 | MMRN2 | PDXK | RAP1B | TAGLN2 | |
| ANXA2 | CLIC4 | FN1 | LRG1 | S100A8 | | CHRDL2 | ISLR | ACTR3 | CCT6A | DDAH1 | FTL | HSP90AE | MPO | PEBP1 | RBBP4 | TBCA | |
| ANXA4 | CLPS | FSTL1 | LTBP1 | S100A9 | | COL16A1 | ISM2 | ADH5 | CCT7 | DDAH2 | FUBP1 | HSPA1A | MPST | PGD | RBMX | TCP1 | |
| ANXA5 | CLSTN1 | FSTL3 | LTBP2 | SCGB1A1 | | EEF1A1 | KLKB1 | ADK | CD33L3 | DDX1 | G6PD | HSPA1B | MRC1 | PGLS | RCN2 | TFF2 | |
| APOA1 | CLU | GAPDH | LUM | SCGB3A1 | | EFNA1 | KRT10 | AEBP1 | CD44 | DDX17 | GALK1 | HSPA2 | MRLC2 | PGLYRP1 | RCN3 | THSA | |
| APOA2 | COL12A1 | GC | LYPD3 | SCGB3A2 | | F10 | KRT13 | AGPAT1 | CDH2 | DECR1 | GALNT2 | HSPA4 | MROH8 | PGM1 | RETN | TINAGL1 | |
| APOA4 | COL15A1 | GM2A | LYZ | SDF4 | | FABP5 | KRT14 | AGRIN | CEP192 | DEFA1 | GANAB | HSPA4L | MRPS22 | PHB | RNASET2 | TMOD3 | |
| APOC1 | COL1A1 | GP2 | MAN1A1 | SERPINA1 | | FAM151A | KRT5 | AK2 | CGB | DES | GARS1 | HSPA5 | MTHFD1 | PKM | RNH1 | TMSL3 | |
| APOC3 | COL1A2 | GPX3 | MARCKS | SERPINA3 | | FLG | KRT79 | AK4 | CHI3L1 | DKK2 | GCN1L1 | HSPA6 | MUC 5AC | PKM1 | RPLP0 | TNC | |
| APOD | COL2A1 | GRN | MASP2 | SERPINA4 | | FOLH1 | KRT84 | AKR1A1 | CHP1 | DKK3 | GDF15 | HSPA9 | MUTED | PKM2 | RPS27A | TNFRSF19 | |
| APOE | COL3A1 | GSN | MEP1A | SERPINA6 | | FUCA2 | LAMB1 | AKR1B1 | CKAP4 | DLD | GFAP | HSPD1 | MVP | PLAUR | RPSA | TNFRSF1A | |
| APOH | COL4A2 | GSTP1 | MFAP4 | SERPINA7 | | GGT1 | LYVE1 | ALDH1B1 | CKB | DLST | GH2 | HYOU1 | MX1 | PLIN3 | RRBP1 | TNFRSF8 | |
| ATRN | COL5A1 | HABP2 | MIF | SERPINC1 | | GOLM1 | MATN2 | ALDH4A1 | CLPB | DNAJB11 | GIPC1 | ICAM1 | MXRA5 | PLOD2 | RTCB | TOMM40 | |
| AXL | COL5A2 | HAPLN1 | MMP2 | SERPIND1 | | HAPLN3 | MUC1 | ALDH7A1 | CLPP | DNASE1 | GLO1 | IDH1 | MYH9 | PLOD3 | RYR2 | TPM1 | |
| AZGP1 | COL6A1 | HBA1 | MSLN | SERPINE2 | | HPR | MUC16 | ALDOA | CMPK1 | DPYSL2 | GLS | IDH2 | MYL12A | PLUNC | S100A12 | TPM2 | |
| B2M | COL6A3 | HBB | MSN | SERPINF1 | | HRNR | MUC2 | ALDOC | CNDP2 | DPYSL3 | GLUD1 | IDH3A | MYL9 | PMPCA | SAA4 | TPM3 | |
| B4GALT1 | COLEC12 | HBD | MUC13 | SERPINF2 | | IGHA2 | MUC5B | ANKAR | CNN2 | DUS3L | GNB1 | IGFALS | MYRF | PNP | SAMM50 | TPM4 | |
| BASP1 | COMP | HP | MUC5AC | SERPING1 | | IGHG2 | NOTUM | ANPEP | CNN3 | DYNC1I2 | GNB2 | IGSF4C | NAMPT | PPA1 | SCOTIN | TPT1 | |
| BMP1 | CP | HPX | MYDGF | SFTPB | | IGHG3 | NT5E | ANXA3 | COIL | ECH1 | GOLPH2 | IL1RL1 | NCL | PPBP | SDHA | TRAP1 | |
| BPIFB1 | CPA1 | HRG | MYL6 | SHBG | | IGHV1-18 | PAPLN | ANXA6 | COL11A1 | ECHS1 | GOT1 | IL6 | NDST3 | PPP1CA | SECTM1 | TUBA1A | |
| BTD | CPM | HSP90AA1 | NID1 | SI | | IGHV1-3 | PLS3 | APEX1 | COL11A2 | EEF1B2 | GOT2 | IMMT | NDUFA8 | PPP1CB | SEPP1 | TUBA1B | |
| C1R | CRISP3 | HSP90B1 | NID2 | SLPI | | IGHV1OR15-1 | PON1 | APOC2 | COL14A1 | EEF1D | GP340 | IRF7 | NDUFS1 | PRDX3 | SEPTIN11 | TUBA4A | |
| C1S | CST3 | HSPA8 | NPC2 | SOD3 | | IGHV3-15 | POTEI | APOM | COL6A2 | EEF1G | GPI | ITGA3 | NDUFS3 | PRDX4 | SEPTIN2 | TUBA8 | |
| C2 | CST6 | HSPB1 | NUCB1 | SPARC | | IGHV3-38 | PPIAL4A | APP | CORO1C | EEF2 | GPR115 | ITGAM | NDUFV1 | PRKCSH | SERPINA5 | TUBB | |
| C3 | CSTB | HSPG2 | OGN | SPARCL1 | | IGHV3-43 | PSG1 | APRT | COTL1 | EFNB2 | GPR135 | ITGAV | NDUFV2 | PRND | SERPINB1 | TUBB4A | |
| C4A | CTGF | HTRA1 | OLFML3 | SPINK1 | | IGHV3-49 | PTMA | ARF1 | CPA2 | EHD1 | GRB2 | ITIH3 | NEXN | PRPF19 | SERPINB4 | TUBB4B | |
| C5 | DAG1 | IGF2 | ORM1 | SPINK5 | | IGHV3-53 | PZP | ARHGDIA | CPB2 | EIF3I | GRHPR | IVD | NFKBIZ | PRPS1 | SERPINE1 | TUFM | |
| C6 | DBI | IGFBP1 | ORM2 | SPINT1 | | IGHV3-66 | S100A6 | ARHGEF19 | CRISPLD1 | EIF6 | GSS | KLK3 | NGFR | PSAT1 | SERPINH1 | TWF2 | |
| C7 | DCN | IGFBP2 | P4HB | SPON1 | | IGHV3-7 | SCUBE2 | ARPC2 | CRK | ELA2 | GSTA1 | KLK6 | NIPSNAP3A | PSG10 | SET | TWSG1 | |
| C8A | DLK1 | IGFBP3 | PAEP | SPON2 | | IGHV3-72 | SERPINA2 | ARPC4 | CRNN | ELA3A | GSTK1 | KRT18 | NME1 | PSG11 | SFTPA1 | TXNRD1 | |
| C8B | DMBT1 | IGFBP4 | PCOLCE | SPP1 | | IGHV3-73 | SERPINB2 | ASGR2 | CRP | EMILIN2 | GSTO1 | KRT7 | NME2 | PSG2 | SGCE | UBA1 | |
| C8G | DMKN | IGFBP5 | PDIA3 | SPRR1B | | IGHV3-9 | SFN | ATIC | CRYAB | ENDOD1 | GVIN1 | LACTB | NNMT | PSMA1 | SH2D3C | UBE2O | |
| C9 | DPP4 | IGFBP6 | PFN1 | SPRR3 | | IGHV3OR16-12 | SHISA5 | ATP5F1A | CRYZ | ENO2 | HADHA | LAMA2 | NOV | PSMA2 | SH3BGRL | UBE2V2 | |
| CA1 | DSC2 | IGFBP7 | PGAM1 | STC2 | | IGHV4-28 | SLC15A1 | ATP5F1B | CS | ERLIN2 | HAUS3 | LAP3 | NRP1 | PSMA3 | SH3BGRL3 | UCHL1 | |
| CALM1 | DSC3 | IGHA1 | PGK1 | TF | | IGHV5-51 | SLC38A10 | ATP5PD | CSF1R | ERP29 | HBE1 | LF | NT5C3L | PSMA4 | SHMT2 | UGDH | |
| CALR | DSG2 | IGHG1 | PGLYRP2 | TFF3 | | IGHV6-1 | SLC9A3R1 | ATP6V1A | CSH1 | ESD | HBE2 | LGALS3 | OAT | PSMA5 | SIGLEC5 | UNC13A | |
| CALU | ECM1 | IGHG4 | PI16 | TGFBI | | IGKV1-16 | TAGLN | AZU1 | CSPG2 | ETFA | HBG1 | LMNA | OBP2A | PSMA6 | SLC12A7 | UQCR | |
| CAMP | EFEMP1 | IGHV1-69 | PIGR | TGFBR3 | | IGKV2-24 | THBS4 | BAMBI | CSPG4 | ETFB | HDLBP | LMNB1 | OGDH | PSMB1 | SLC25A24 | UQCRC1 | |
| CD14 | EFEMP2 | IGKC | PLG | THBS1 | | IGKV2-28 | TMSB10 | BCAT2 | CST1 | ETHE1 | HGFL | LOC139249 | OLA1 | PSMB2 | SLC27A6 | UQCRC2 | |
| CD163 | EFNB1 | IGKV1-5 | PLOD1 | THY1 | | IGKV2-30 | TTBK2 | BP1 | CST2 | FAM71F1 | HIBCH | LOC143244 | OLFM4 | PSMB5 | SLC39A12 | VCAM1 | |
| CD248 | ENO1 | ITIH1 | PLTP | TIMP1 | | IGKV3-15 | TTN | BPNT1 | CST4 | FAM83B | HINT1 | LOC730855 | OTUB1 | PSMC1 | SLIRP | VCL | |
| CD55 | ENPP2 | ITIH2 | POSTN | TIMP2 | | IGKV3-20 | TXNDC5 | BSG | CSTA | FBLN5 | HIST1H1D | LONP1 | OXCT1 | PSMC2 | SND1 | VCP | |
| CD59 | ENPP7 | ITIH4 | PPIA | TKT | | IGKV3-7 | VCAN | BST1 | CSTF1 | FBN2 | HIST1H2BL | LOXL2 | OXRP | PSMC3 | SNRPA1 | VDAC1 | |
| CDH1 | EZR | IVL | PRDX1 | TMSB4X | | | VSIG4 | BUB3 | | | | LPHN1 | OXT | PSMC4 | SNX6 | VDAC2 | |

**A**

| FROM PROTEOME | Fold Enrichment | p-value |
|---|---|---|
| Plasma | 17.81 | 1.64E-87 |
| Liver | 3.70 | 5.72E-46 |
| Saliva | 20.12 | 5.18E-19 |
| Fetal brain cortex | 6.58 | 3.51E-14 |
| Cajal-Retzius cell | 7.19 | 2.81E-14 |
| Bile | 27.68 | 2.51E-13 |
| Platelet | 3.87 | 4.98E-11 |
| Serum | 31.94 | 1.26E-10 |
| Placenta | 1.84 | 2.10E-10 |
| Urine | 21.29 | 8.69E-09 |
| Osteosarcoma | 11.50 | 1.63E-05 |
| Keratinocyte | 5.67 | 1.92E-05 |
| Cerebrospinal fluid | 41.06 | 4.50E-05 |
| Amniotic fluid | 38.32 | 1.40E-03 |
| Colon | 1.79 | 6.14E-03 |
| Pancreas | 1.84 | 8.74E-03 |
| Intestine | 8.21 | 8.93E-03 |
| Blood | 2.04 | 9.23E-03 |
| Pituitary | 3.27 | 3.64E-02 |
| Fetal liver | 3.01 | 3.54E-02 |
| Ascites | 28.74 | 3.87E-02 |
| Fibroblasts | 3.47 | 3.91E-02 |
| Cartilage | 8.84 | 7.56E-02 |
| Lung | 1.32 | 9.75E-02 |
| Skin | 1.40 | 9.63E-02 |
| Endothelial cell | 4.79 | 1.30E-01 |
| Heart | 1.72 | 1.99E-01 |
| Decidua | 57.48 | 1.95E-01 |
| Tracheobronchial mucosa | 57.48 | 1.95E-01 |
| Adipose tissue | 2.46 | 2.42E-01 |
| Leukocyte | 2.58 | 2.81E-01 |
| Adult brain | 28.74 | 3.29E-01 |
| Erythrocyte | 6.90 | 3.29E-01 |
| Foreskin | 4.18 | 3.25E-01 |
| Epididymis | 6.39 | 3.53E-01 |
| Aorta | 2.61 | 3.49E-01 |
| Seminal plasma | 22.99 | 3.56E-01 |
| Synovial fluid | 22.99 | 3.56E-01 |
| Subthalamic nucleus | 3.71 | 3.80E-01 |

| FROM METABOLOME | Fold Enrichment | p-value |
|---|---|---|
| Prostate | 2.62 | 1.47E-05 |
| Small Intestine | 6.67 | 6.07E-02 |
| Bladder | 2.30 | 7.49E-02 |
| Muscle | 1.88 | 1.37E-01 |
| Skeletal Muscle | 1.95 | 1.42E-01 |
| Placenta | 1.64 | 2.93E-01 |
| Epidermis | 1.57 | 3.38E-01 |
| Spleen | 1.53 | 4.16E-01 |
| Sperm | 2.50 | 4.43E-01 |
| Pancreas | 1.53 | 4.52E-01 |
| Nervous Tissue | 1.67 | 5.85E-01 |
| Erythrocyte | 1.18 | 7.05E-01 |
| Lung | 1.00 | 7.71E-01 |
| Adrenal Gland | 0.67 | 8.91E-01 |
| Skin | 0.96 | 8.96E-01 |
| Nerve Cells | 0.57 | 9.25E-01 |
| Platelet | 0.74 | 9.59E-01 |
| Fibroblasts | 0.87 | 9.63E-01 |
| Intestine | 0.96 | 9.65E-01 |
| Testes | 0.56 | 9.70E-01 |
| Kidney | 0.61 | 9.95E-01 |
| Brain | 0.33 | 9.99E-01 |
| Liver | 0.09 | 1.00E+00 |

**B**

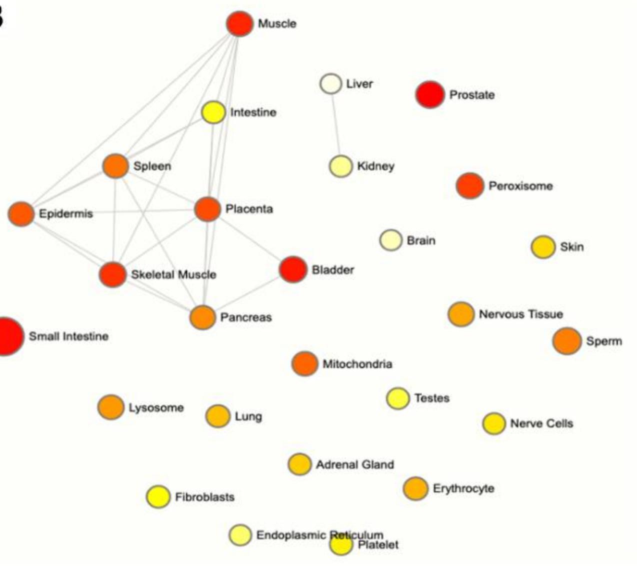


**Figure S3. Differential expression of metabolites and proteins and their tissue and subcellular distribution.** **A.** The enrichment of proteins and metabolites in AF was utilized to identify the associated tissues and subcellular compartments. In pink, tissues identified by the differential omics. **B**. Visualization of the combined data from A. Each node represents a protein-metabolite set with its color based on its *p* value and its size based on the fold enrichment. Two sets are connected by an edge if the number of their shared items is over 25% of the total number of their combined item sets. Analyses performed with DAVID and MetaboAnalyst.

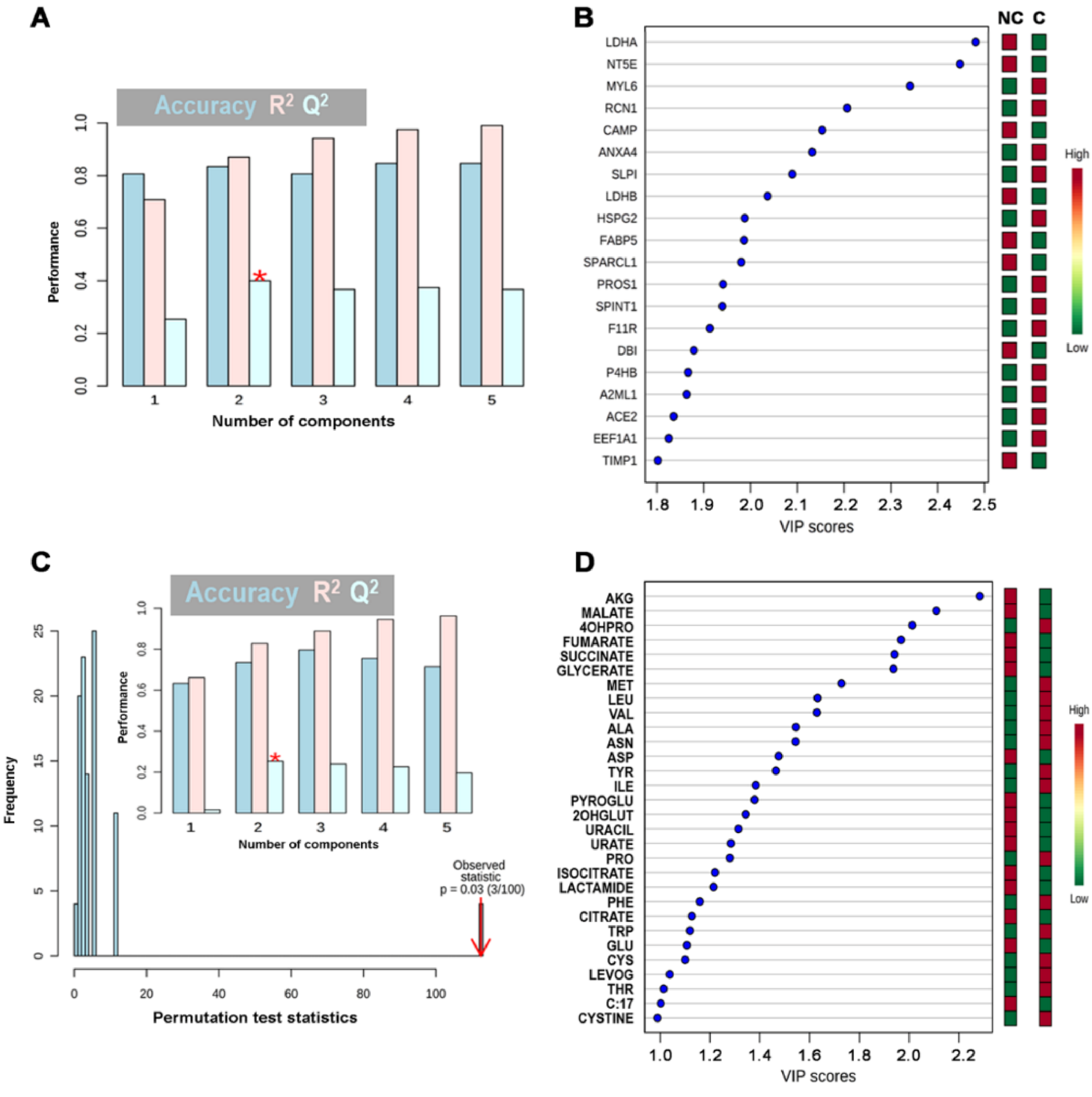


**Figure S4. Differential expression of proteins and metabolites identified in AF of carriers and non-carrier fetuses.** To identify the proteins and metabolites that separated the most carriers and non-carriers (see **Figure 2**), a PLS-DA was performed. The two groups were separated by utilizing 2 components (**A** and **C**) which gave the most accurate separation. Asterisk identifies the component with the highest predictive value. The top 20 proteins (**B**) and the top 30 metabolites (**D**) identified based on the VIP scores are shown. Abbreviations: amino acids are referred by their 3-letter code; AKG, alpha-ketoglutarate; 4OHPRO, 4-hydroxyproline; PYROGLU, pyroglutamate; 2HOGLUT, 2-hydroxyglutarate; LEVOG, levoglucosan; C:17, heptanoic acid. Analysis was performed by utilizing MetaboAnalyst [8].

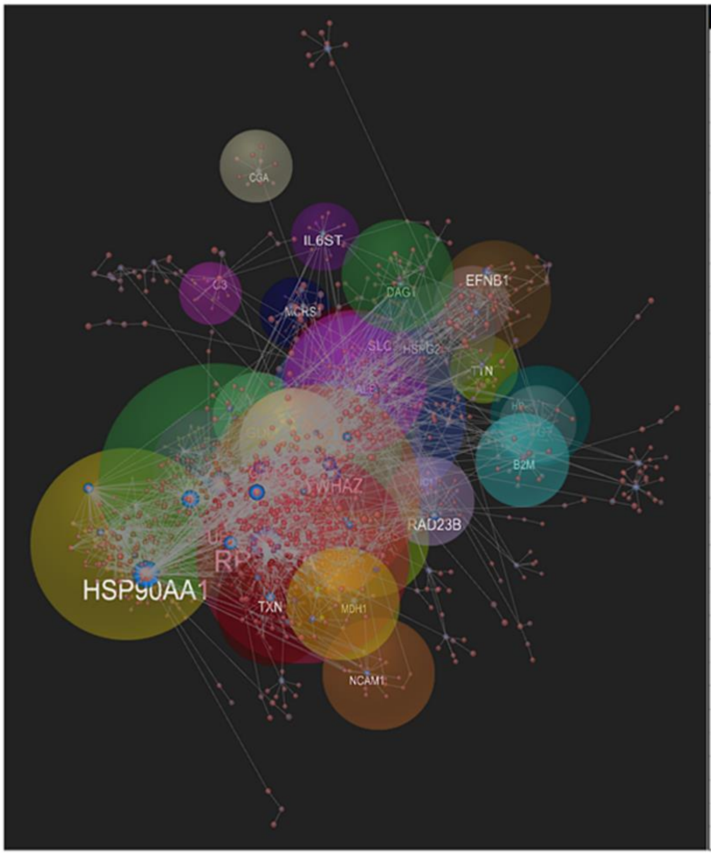


| MODULE | SIZE | -LOG P | PATHWAY |
|---|---|---|---|
| 0 | 292 | 91.94 | Translation |
| 1 | 190 | 37.48 | Rho GTPase cycle |
| 2 | 181 | 50.70 | Unfolded Protein Response |
| 3 | 172 | 11.40 | Integrin cell surface interactions |
| 4 | 112 | 18.05 | NGF signalling via TRKA from the plasma membrane |
| 5 | 86 | 20.70 | Protein folding |
| 6 | 65 | 15.22 | Class A/1 (Rhodopsin-like receptors) |
| 7 | 54 | 7.27 | Lipoprotein metabolism |
| 8 | 51 | 12.96 | G alpha (s) signalling events |
| 9 | 31 | 4.80 | Formation of Fibrin Clot (Clotting Cascade) |
| 10 | 30 | 3.78 | Antigen Presentation: Folding, assembly and peptide loading of class I MHC |
| 11 | 30 | 2.36 | Global Genomic NER (GG-NER) |
| 12 | 27 | 4.47 | FCGR activation |
| 13 | 23 | 3.02 | Glycolysis |
| 14 | 22 | 3.05 | Synthesis and interconversion of nucleotide di- and triphosphates |
| 15 | 21 | 4.03 | Rho GTPase cycle |
| 16 | 21 | 1.52 | TGF-beta receptor signaling activates SMADs |
| 17 | 20 | 3.65 | p75 NTR receptor-mediated signalling |
| 18 | 20 | 3.43 | Downregulation of TGF-beta receptor signaling |
| 19 | 20 | 3.22 | Molecules associated with elastic fibres |
| 20 | 20 | 1.65 | Axon guidance |
| 21 | 17 | 2.97 | Integrin cell surface interactions |
| 22 | 16 | 3.86 | Pyruvate metabolism and Citric Acid (TCA) cycle |
| 23 | 16 | 3.04 | Regulation of Insulin Secretion by Free Fatty Acids |
| 24 | 16 | 0.71 | NCAM signaling for neurite out-growth |
| 25 | 15 | 3.57 | Interleukin-6 signaling |
| 26 | 14 | 2.88 | Antigen Presentation: Folding, assembly and peptide loading of class I MHC |
| 27 | 12 | 3.99 | Regulation of Complement cascade |
| 28 | 12 | 3.99 | Striated Muscle Contraction |
| 29 | 12 | 3.32 | Metabolism of Angiotensinogen to Angiotensins |
| 30 | 11 | 3.37 | Scavenging of Heme from Plasma |
| 31 | 11 | 2.00 | Activation of Matrix Metalloproteinases |
| 32 | 11 | 1.97 | Formation of Fibrin Clot (Clotting Cascade) |
| 33 | 10 | 3.91 | Glycoprotein hormones |
| 34 | 10 | 2.13 | Utilization of Ketone Bodies |

**Figure S5. Overrepresented pathways and energy metabolism in prenatal PM.** Differentially affected pathways between the genotypes were identified through a joint metabolome-proteome analysis performed with those with a VIP≥0.8 by mining the WikiPathways and REACTOME pathway databases. Each module (**Supplementary Dataset 2**) was analyzed for pathway over-representation mining the REACTOME database. This last analysis revealed the presence of 34 modules (shown with size of nodes and significance) highlighting multiple interconnected pathways. The main pathway (292 between proteins and metabolites), regardless of the direction, was associated with translation, followed by unfolded protein response (UPR) and protein folding.

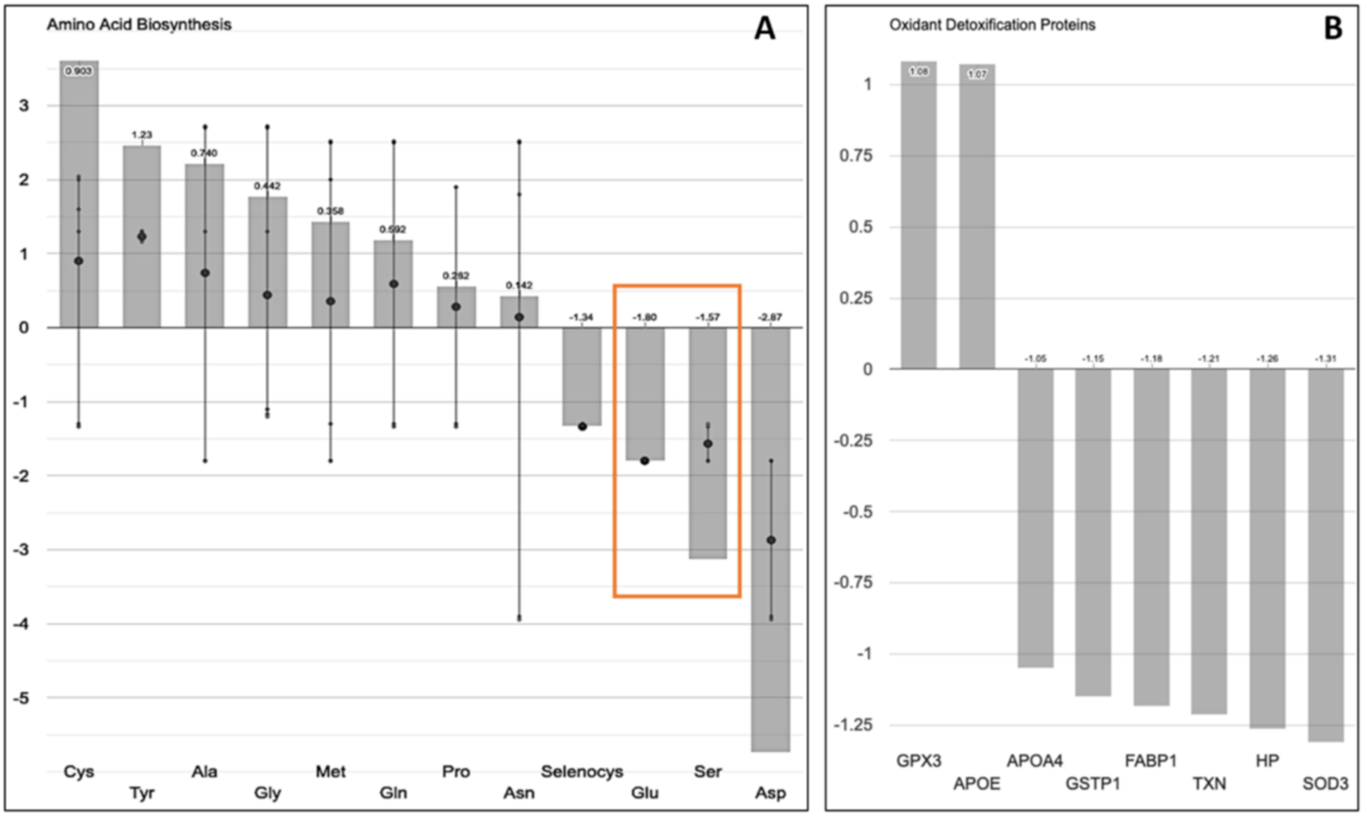


**Figure S6. Differentially expressed cellular processes linked to serine metabolism and antioxidant defenses.** The metabolism of Serine (**A**) as well as the detoxification pathway (**B**), the latter centered on antioxidant defenses against reactive oxygen species and derivatives, were down-regulated in AF from PM carriers. Analysis was obtained with BioCyc [165]. Abbreviations: APOA4, Apolipoprotein A4; APOE, Apolipoprotein E; GPX3, Glutathione Peroxidase 3; GSTP1, Glutathione S-Transferase Pi 1; FABP1, Fatty Acid-Binding Protein 1; HP, Haptoglobin; SOD3, Superoxide Dismutase 3; TXN, Thioredoxin.

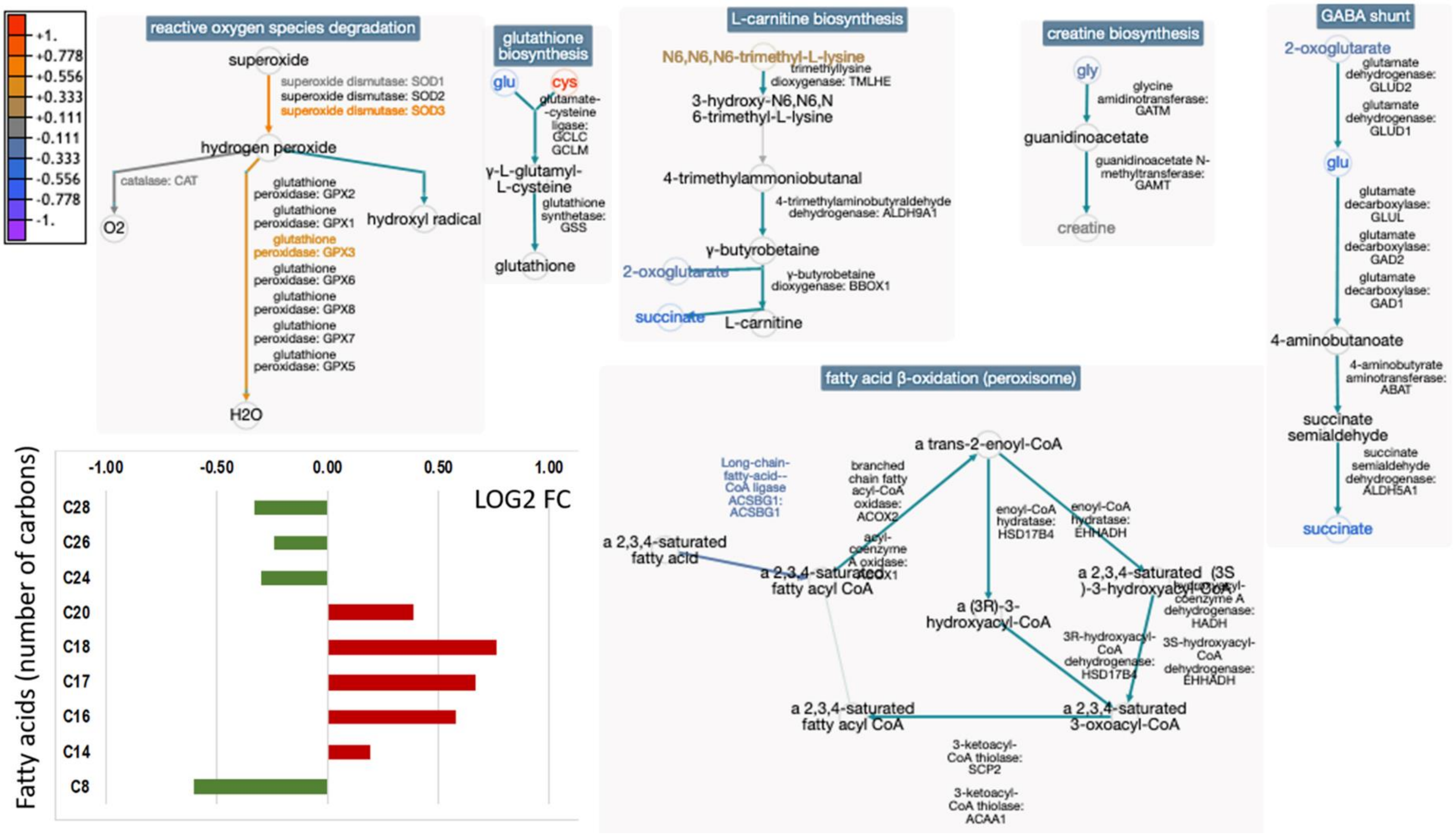


**Figure S7. Serine- and glycine-dependent pathways in AF from carriers and non-carriers.** Identification of Ser- and Gly-dependent pathways affected in premutation carriers. Glutathione biosynthesis (which requires Glu) was downregulated, along with creatine biosynthesis (requiring Gly), carnitine biosynthesis (requiring AKG), and the GABA shunt (AKG, Glu, succinate). The detoxification of ROS seemed hampered by lower glutathione synthesis. On the other hand, the expression of glutathione peroxidase 3 and the extracellular superoxide dismutase (SOD3) were increased. The content of very long and long chain fatty acids was lower in carriers and mirrored by an accumulation of long and medium-chain fatty acids, that are processed at the mitochondrial level.

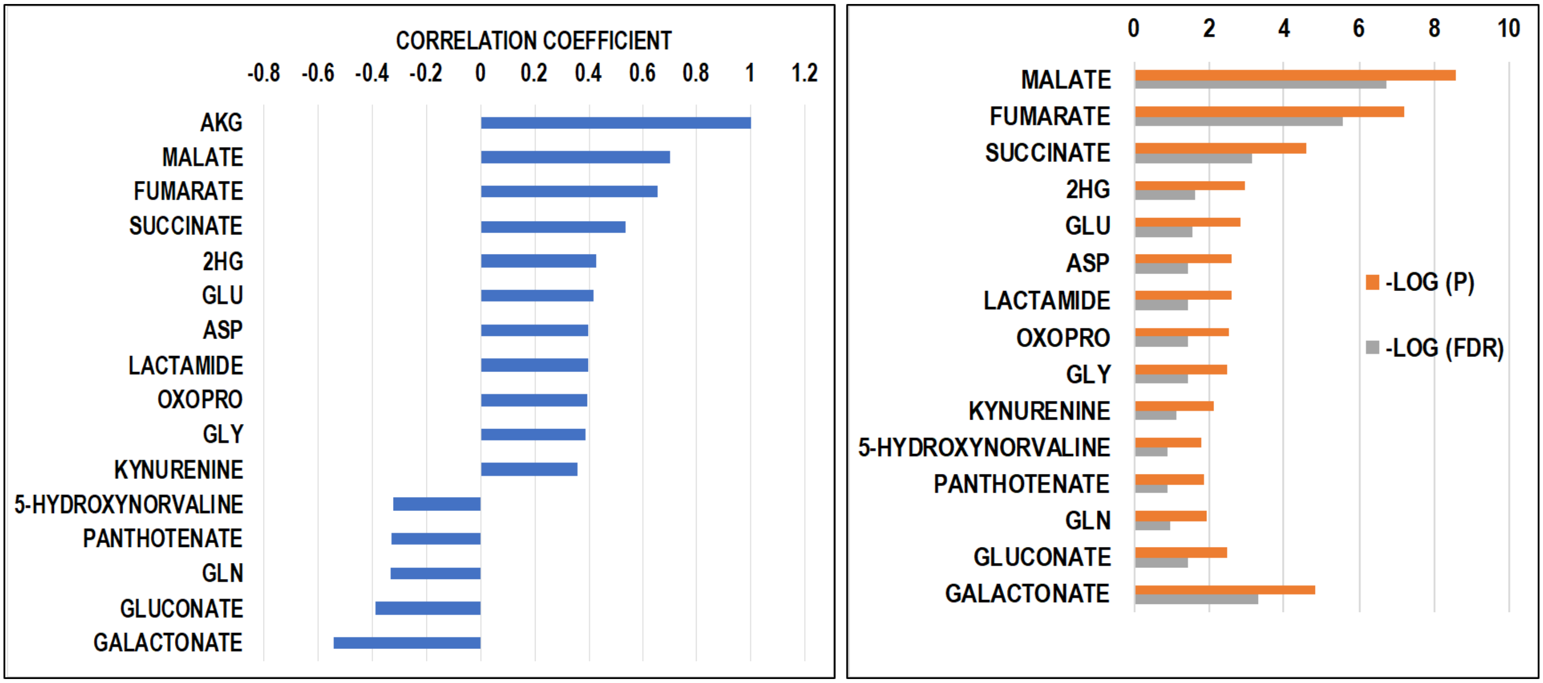


**Figure S8. TCA cycle metabolites correlation analysis.** A linear correlation analysis (Pearson's) was performed to explore whether a correlation existed among levels of metabolites belonging to the TCA cycle. The compound AKG directly correlated with malate, fumarate, succinate, 2-hydroxyglutarate, Glu, Asp and Gly, and inversely correlated with Gln and panthotenate. Log P values and false discovery rate (FDR) are shown, along with the correlation coefficients.

**A**

| Pathways | Present | PDH3 KO | CHOP/ ATF4 | OGD | Mito stress |
|---|---|---|---|---|---|
| ***RESPONSE TO DAMAGE AND STRESS*** | | | | | |
| Response to DNA damage | ↑ | | ↑ | | |
| Ubiquitin-mediated proteolysis | ↑ | | | ↑ | |
| Unfolded protein response | ↑ | | ↑ | | |
| Apoptosis | ↑ | ↑ | ↑ | | |
| Response to oxidative stress | ↓ | | ↓ | | |
| ***TRANSLATION AND PROTEIN SYNTHESIS*** | | | | | |
| Aminoacyl-tRNA biosynthesis | ↑ | | ↑ | | ↑ |
| mRNA processing | ↑ | ↑ | | | |
| Ribosomes | ↑ | ↑ | ↑ | | ↓ |
| Translation initiation | ↑ | | ↑ | ↑ | |
| ***ENERGY*** | | | | | |
| Glycolysis / Gluconeogenesis | ↓ | ↓ | | | |
| TCA cycle and OXPHOS | ↓ | ↑ | | ↓ | ↓ |
| ***DISEASES*** | | | | | |
| Alzheimer's | ↑ | | | ↑ | ↑ |
| Breast cancer | ↑ | | | | ↑ |
| Parkinson's disease | ↑ | | | ↑ | ↑ |
| Non-alcoholic fatty liver disease | ↓ | | ↓ | | |
| ***METABOLISM and TRANSPORT*** | | | | | |
| Amino acid transport | ↑ | | ↑ | | |
| Cys and Met metabolism | ↑ | | ↑ | | ↑ |
| Lipid transport | ↑ | | ↓ | | |
| Nicotinamide metabolism | ↑ | ↑ | | | |
| One carbon pool by folate | ↑ | | | | ↑ |
| Vitamin B6 metabolism | ↑ | | | | ↑ |
| Ala, Asp and Glu metabolism | ↓ | | ↑ | | ↑ |
| Alpha-linolenic acid metabolism | ↓ | | | | ↓ |
| Biosynthesis of amino acids | ↓ | | ↑ | | ↑ |
| Gly, Ser and Thr metabolism | ↓ | | ↑ | | ↑ |
| Nitrogen metabolism | ↓ | | ↑ | | |
| Steroid biosynthesis | ↓ | | ↓ | | ↓ |
| BCAA degradation | ↓ | | | | ↑ |
| ***SIGNALING*** | | | | | |
| HIF-1 signaling pathway | ↓ | ↓ | ↓ | ↑ | |
| mTOR signaling | ↓ | | | | ↑ |
| ***OTHERS*** | | | | | |
| Regulation of actin cytoskeleton | ↑ | ↑ | | ↓ | |
| Development | ↓ | | ↓ | | |

**B**

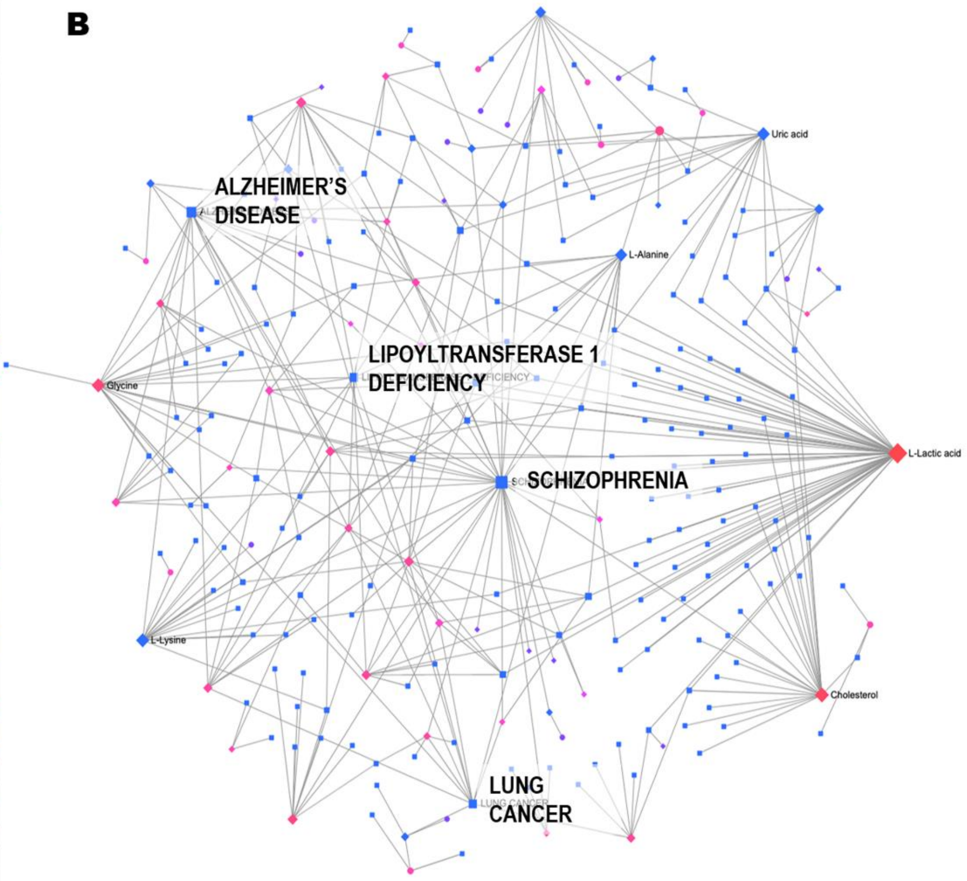


**Figure S9. Overlap of stress-related biological pathways and human disorders with the PM prenatal metabolic footprint. A.** Differentially regulated pathways from our study and those observed as a result of four stressors, namely (from left to right): PHD3 silencing [166]; tunicamycin-dependent ER stress [167]; OGD [168]; and mitochondrial stressors [169]. Up- and down-regulated in red and blue, respectively. Darker colors indicate a match with the direction of change observed in our study. Top, 95% confidence intervals (95%CI) of pathways that matched our study over all pathways for each of the stressors. **B.** Our combined omics dataset was analyzed via a network analysis utilizing databases of human diseases. The most significant ones associated with the PM were those with underlying MD (see also **Supplementary Table S3**).

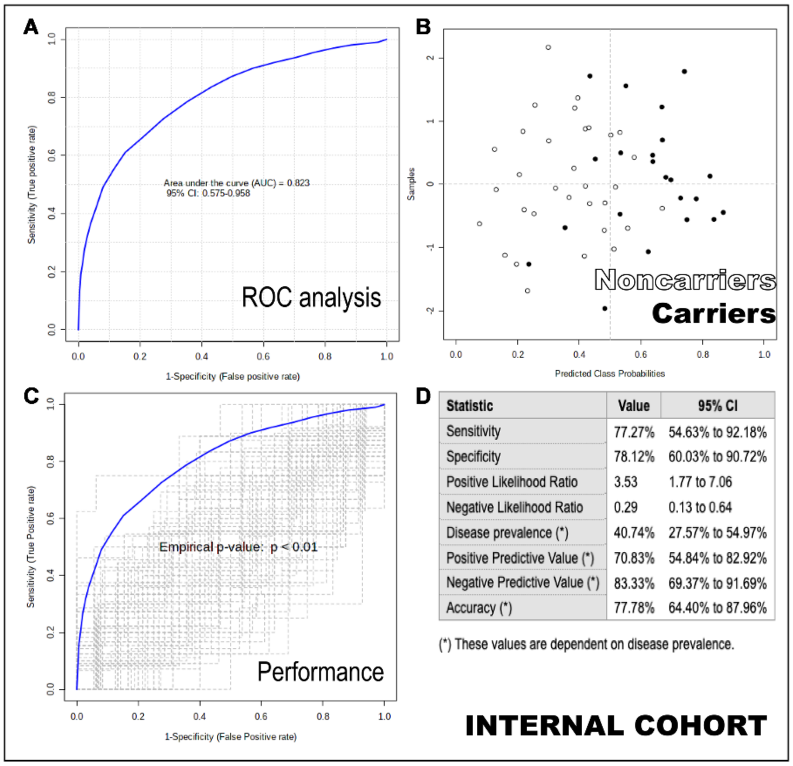


| Statistic | Value | 95% CI |
|---|---|---|
| Sensitivity | 77.27% | 54.63% to 92.18% |
| Specificity | 78.12% | 60.03% to 90.72% |
| Positive Likelihood Ratio | 3.53 | 1.77 to 7.06 |
| Negative Likelihood Ratio | 0.29 | 0.13 to 0.64 |
| Disease prevalence (*) | 40.74% | 27.57% to 54.97% |
| Positive Predictive Value (*) | 70.83% | 54.84% to 82.92% |
| Negative Predictive Value (*) | 83.33% | 69.37% to 91.69% |
| Accuracy (*) | 77.78% | 64.40% to 87.96% |

(*) These values are dependent on disease prevalence.

**Figure S10. Receiver operating characteristic (ROC) curves built with proteomics and metabolomics data from AF, plasma and fibroblasts.** A ROC curve (**A**) for the predictive performance of the prenatal PM metabolic footprint was built utilizing 2/3 of the AF samples (internal cohort) and the top VIP predictors from both proteomic and metabolomic data, and then further tested by incorporating the remaining AF samples as holdout ones (internal cohort; **B-D**). In a second approach, we utilized the proteomics data from all AF samples and used as holdout samples those constituted by proteomics of skin fibroblasts from 25 subjects (**E-H**). We also utilized metabolomics from all AF samples and used as holdout ones those constituted by metabolomics data from 39 subjects (**I-L**). The performance of each model is shown after 100-cross validations (**C** with internal validation cohorts and **G** and **K** with external validation cohorts). The ROC curves (pink, **E**, **I**) and the predicted class probabilities for the AF (black) and their hold-out samples (red) are shown in panels F, J. From the confusion matrices, the diagnostic test evaluations, based on accuracy, were calculated (**D**, **H**, **L**).

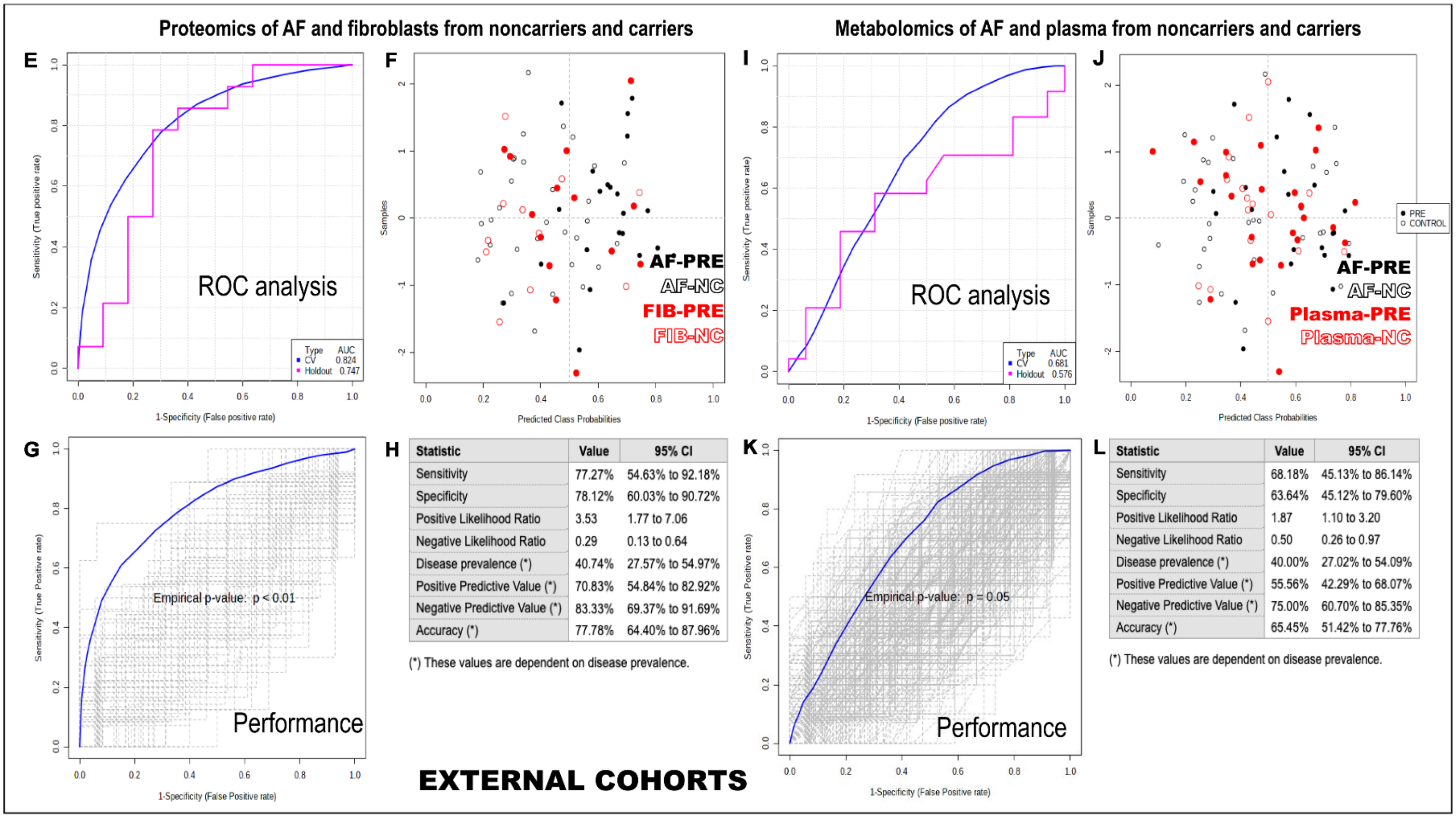


| Statistic | Value | 95% CI |
|---|---|---|
| Sensitivity | 77.27% | 54.63% to 92.18% |
| Specificity | 78.12% | 60.03% to 90.72% |
| Positive Likelihood Ratio | 3.53 | 1.77 to 7.06 |
| Negative Likelihood Ratio | 0.29 | 0.13 to 0.64 |
| Disease prevalence (*) | 40.74% | 27.57% to 54.97% |
| Positive Predictive Value (*) | 70.83% | 54.84% to 82.92% |
| Negative Predictive Value (*) | 83.33% | 69.37% to 91.69% |
| Accuracy (*) | 77.78% | 64.40% to 87.96% |

(*) These values are dependent on disease prevalence.

| Statistic | Value | 95% CI |
|---|---|---|
| Sensitivity | 68.18% | 45.13% to 86.14% |
| Specificity | 63.64% | 45.12% to 79.60% |
| Positive Likelihood Ratio | 1.87 | 1.10 to 3.20 |
| Negative Likelihood Ratio | 0.50 | 0.26 to 0.97 |
| Disease prevalence (*) | 40.00% | 27.02% to 54.09% |
| Positive Predictive Value (*) | 55.56% | 42.29% to 68.07% |
| Negative Predictive Value (*) | 75.00% | 60.70% to 85.35% |
| Accuracy (*) | 65.45% | 51.42% to 77.76% |

(*) These values are dependent on disease prevalence.

## Supplementary References


1. Fiehn, O.; Wohlgemuth, G.; Scholz, M.; Kind, T.; Lee, D. Y.; Lu, Y.; Moon, S.; Nikolau, B. Quality control for plant metabolomics: reporting MSI-compliant studies. *Plant J.* **2008,** *53*, 691-704,doi:10.1111/j.1365-313X.2007.03387.x
2. Napoli, E.; Tassone, F.; Wong, S.; Angkustsiri, K.; Simon, T. J.; Song, G.; Giulivi, C. Mitochondrial Citrate Transporter-dependent Metabolic Signature in the 22q11.2 Deletion Syndrome. *J. Biol. Chem.* **2015,** *290*, 23240-53,doi:10.1074/jbc.M115.672360
3. Fujisawa, Y.; Napoli, E.; Wong, S.; Song, G.; Yamaguchi, R.; Matsui, T.; Nagasaki, K.; Ogata, T.; Giulivi, C. Impact of a novel homozygous mutation in nicotinamide nucleotide transhydrogenase on mitochondrial DNA integrity in a case of familial glucocorticoid deficiency. *BBA Clin.* **2015,** *3*, 70-78,doi:10.1016/j.bbacli.2014.12.003
4. Napoli, E.; Song, G.; Schneider, A.; Hagerman, R.; Eldeeb, M. A.; Azarang, A.; Tassone, F.; Giulivi, C. Warburg effect linked to cognitive-executive deficits in FMR1 premutation. *FASEB J.* **2016,** *30*, 3334-3351,doi:10.1096/fj.201600315R
5. Napoli, E.; Song, G.; Panoutsopoulos, A.; Riyadh, M. A.; Kaushik, G.; Halmai, J.; Levenson, R.; Zarbalis, K. S.; Giulivi, C. Beyond autophagy: a novel role for autism-linked Wdfy3 in brain mitophagy. *Sci. Rep.* **2018,** *8*, 11348,doi:10.1038/s41598-018-29421-7
6. Giulivi, C.; Napoli, E.; Tassone, F.; Halmai, J.; Hagerman, R. Plasma metabolic profile delineates roles for neurodegeneration, pro-inflammatory damage and mitochondrial dysfunction in the FMR1 premutation. *Biochem. J.* **2016,** *473*, 3871-3888,doi:10.1042/BCJ20160585
7. Bleeker, S. E.; Moll, H. A.; Steyerberg, E. W.; Donders, A. R. T.; Derksen-Lubsen, G.; Grobbee, D. E.; Moons, K. G. M. External validation is necessary in, prediction research: A clinical example. *J. Clin. Epidemiol.* **2003,** *56*, 826-832,doi:10.1016/S0895-4356(03)00207-5
8. Chong, J.; Wishart, D. S.; Xia, J. Using MetaboAnalyst 4.0 for Comprehensive and Integrative Metabolomics Data Analysis. *Curr. Protoc. Bioinformatics* **2019,** *68*, e86,doi:10.1002/cpbi.86
9. Vickers, A. J.; Cronin, A. M.; Elkin, E. B.; Gonen, M. Extensions to decision curve analysis, a novel method for evaluating diagnostic tests, prediction models and molecular markers. *BMC Med. Inform. Decis. Mak.* **2008,** *8*, 53,doi:10.1186/1472-6947-8-53
10. Vickers, A. J.; Elkin, E. B. Decision curve analysis: a novel method for evaluating prediction models. *Med. Decis. Making* **2006,** *26*, 565-74,doi:10.1177/0272989X06295361
11. Cho, C. K.; Shan, S. J.; Winsor, E. J.; Diamandis, E. P. Proteomics analysis of human amniotic fluid. *Mol. Cell. Proteomics: MCP* **2007,** *6*, 1406-15,doi:10.1074/mcp.M700090-MCP200
12. Abdel-Razeq, S. S.; Buhimschi, I. A.; Bahtiyar, M. O.; Rosenberg, V. A.; Dulay, A. T.; Han, C. S.; Werner, E. F.; Thung, S.; Buhimschi, C. S. Interpretation of amniotic fluid white blood cell count in "bloody tap" amniocenteses in women with symptoms of preterm labor. *Obstet. Gynecol.* **2010,** *116*, 344-54,doi:10.1097/AOG.0b013e3181e8fec6
13. Abu Shehab, M.; Inoue, S.; Han, V. K. M.; Gupta, M. B. Site Specific Phosphorylation of Insulin-Like Growth Factor Binding Protein-1 (IGFBP-1) for Evaluating Clinical Relevancy in Fetal Growth Restriction. *J. Proteome Res.* **2009,** *8*, 5325-5335,doi:10.1021/pr900633x
14. Abu Shehab, M.; Khosravi, J.; Han, V. K. M.; Shilton, B. H.; Gupta, M. B. Site-Specific IGFBP-1 Hyper-Phosphorylation in Fetal Growth Restriction: Clinical and Functional Relevance. *J. Proteome Res.* **2010,** *9*, 1873-1881,doi:10.1021/pr900987n
15. Aiello, D.; Giambona, A.; Leto, F.; Passarello, C.; Damiani, G.; Maggio, A.; Siciliano, C.; Napoli, A. Human coelomic fluid investigation: A MS-based analytical approach to prenatal screening. *Sci. Rep.* **2018,** *8*,doi:10.1038/s41598-018-29384-9
16. Alam, M.; Mahajan, M.; Raziuddin, M.; Singh, T. P.; Yadav, S. Proteomics-based approach for identification and purification of human phosphate binding apolipoprotein from amniotic fluid. *Genet. Mol. Res.* **2009,** *8*, 929-937,doi:10.4238/vol8-3gmr620
17. Anagnostopoulos, A. K.; Kolialexi, A.; Mavrou, A.; Vougas, K.; Papantoniou, N.; Antsaklis, A.; Kanavakis, E.; Fountoulakis, M.; Tsangaris, G. T. Proteomic analysis of amniotic fluid in pregnancies with Klinefelter syndrome foetuses. *J. Proteomics* **2010,** *73*, 943-950,doi:10.1016/j.jprot.2009.12.009
18. Arnon, S.; Litmanovitz, I. Diagnostic tests in neonatal sepsis. *Curr. Opin. Infect. Dis.* **2008,** *21*, 223-227,doi:DOI 10.1097/QCO.0b013e3282fa15dd

19. Bahtiyar, M. O.; Copel, J. A.; Mahoney, M. J.; Buhimschi, I. A.; Buhimschi, C. S. Proteomics: A novel methodology to complement prenatal diagnosis of chromosomal abnormalities and inherited human diseases. *Am. J. Perinat.* **2007,** *24*, 167-181,doi:10.1055/s-2007-972927
20. Bazrafshan, A.; Owji, M.; Yazdani, M.; Varedi, M. Activation of mitosis and angiogenesis in diabetes-impaired wound healing by processed human amniotic fluid. *J. Surg. Res.* **2014,** *188*, 545-552,doi:10.1016/j.jss.2014.01.041
21. Bitsika, V.; Roubelakis, M. G.; Zagoura, D.; Trohatou, O.; Makridakis, M.; Pappa, K. I.; Marini, F. C.; Vlahou, A.; Anagnou, N. P. Human Amniotic Fluid-Derived Mesenchymal Stem Cells As Therapeutic Vehicles: A Novel Approach For the Treatment of Bladder Cancer. *Stem Cells Dev.* **2012,** *21*, 1097-1111,doi:10.1089/scd.2011.0151
22. Bredeson, S.; Papaconstantinou, J.; Deford, J. H.; Kechichian, T.; Syed, T. A.; Saade, G. R.; Menon, R. HMGB1 Promotes a p38MAPK Associated Non-Infectious Inflammatory Response Pathway in Human Fetal Membranes. *Plos One* **2014,** *9*,doi:10.1371/journal.pone.0113799
23. Brown, K. J.; Formolo, C. A.; Seol, H.; Marathi, R. L.; Duguez, S.; An, E.; Pillai, D.; Nazarian, J.; Rood, B. R.; Hathout, Y. Advances in the proteomic investigation of the cell secretome. *Expert Rev. Proteomic* **2012,** *9*, 337-345,doi:10.1586/Epr.12.21
24. Buhimschi, C. S.; Abdel-Razeq, S.; Cackovic, M.; Pettker, C. M.; Dulay, A. T.; Bahtiyar, M. O.; Zambrano, E.; Martin, R.; Norwitz, E. R.; Bhandari, V., et al. Fetal heart rate monitoring patterns in women with amniotic fluid proteomic profiles indicative of inflammation. *Am. J. Perinat.* **2008,** *25*, 359-372,doi:10.1055/s-2008-1078761
25. Buhimschi, C. S.; Bhandari, V.; Hamar, B. D.; Bahtiyar, M. O.; Zhao, G. M.; Sfakianaki, A. K.; Pettker, C. M.; Magloire, L.; Funai, E.; Norwitz, E. R., et al. Proteomic profiling of the amniotic fluid to detect inflammation, infection, and neonatal sepsis. *Plos Med.* **2007,** *4*, 84-94,doi:10.1371/journal.pmed.0040018
26. Buhimschi, C. S.; Bhandari, V.; Han, Y. W.; Dulay, A. T.; Balumbusch, M. A.; Madri, J. A.; Buhimschi, I. A. Using proteomics in perinatal and neonatal sepsis: hopes and challenges for the future. *Curr. Opin. Infect. Dis.* **2009,** *22*, 235-243,doi:10.1097/QCO.0b013e32832a5963
27. Buhimschi, C. S.; Buhimschi, I. A.; Abdel-Razeq, S.; Rosenberg, V. A.; Thung, S. F.; Zhao, G. M.; Wang, E.; Bhandari, V. Proteomic biomarkers of intra-amniotic inflammation: Relationship with funisitis and early-onset sepsis in the premature neonate. *Ped. Res.* **2007,** *61*, 318-324,doi:10.1203/01.pdr.0000252439.48564.37
28. Buhimschi, C. S.; Dulay, A. T.; Abdel-Razeq, S.; Zhao, G.; Lee, S.; Hodgson, E. J.; Bhandari, V.; Buhimschi, I. A. Fetal inflammatory response in women with proteomic biomarkers characteristic of intra-amniotic inflammation and preterm birth. *Bjog-Int. J. Obstet. Gy.* **2009,** *116*, 257-267,doi:10.1111/j.1471-0528.2008.01925.x
29. Buhimschi, C. S.; Weiner, C. P.; Buhimschi, I. A. Proteomics, part II: The emerging role of proteomics over genomics in spontaneous preterm labor/birth. *Obstet. Gynecol. Surv.* **2006,** *61*, 543-553,doi:DOI 10.1097/01.ogx.0000228779.39568.59
30. Buhimschi, I. A. Using SELDI-TOF Mass Spectrometry on Amniotic Fluid and for Clinical Proteomics and Theranostics in Disorders of Pregnancy. *Methods Mol. Biol.* **2012,** *818*, 171-197,doi:10.1007/978-1-61779-418-6_13
31. Buhimschi, I. A.; Buhimschi, C. S. Proteomics of the amniotic fluid in assessment of the placenta. Relevance for preterm birth. *Placenta* **2008,** *29*, S95-S101,doi:10.1016/j.placenta.2007.12.001
32. Buhimschi, I. A.; Buhimschi, C. S. Proteomics/diagnosis of chorioamnionitis and of relationships with the fetal exposome. *Semin. Fetal Neonat. M.* **2012,** *17*, 36-45,doi:10.1016/j.siny.2011.10.002
33. Buhimschi, I. A.; Buhimschi, C. S.; Christner, R.; Norwitz, E.; Weiner, C. P. Proteomic profiling and intra-amniotic infection. *Jama-J. Am. Med. Assoc.* **2004,** *292*, 2338-2338,doi:DOI 10.1001/jama.292.19.2338-a
34. Buhimschi, I. A.; Buhimschi, C. S.; Christner, R.; Weiner, C. P. Proteomics technology for the accurate diagnosis of inflammation in twin pregnancies. *Bjog-Int. J. Obstet. Gy.* **2005,** *112*, 250-255,doi:10.1111/j.1471-0528.2004.00341.x
35. Buhimschi, I. A.; Buhimschi, C. S.; Weiner, C. P.; Kimura, T.; Hamar, B. D.; Sfakianaki, A. K.; Norwitz, E. R.; Funai, E. F.; Ratner, E. Proteomic but not enzyme-linked immunosorbent assay technology detects amniotic fluid monomeric calgranulins from their complexed calprotectin form. *Clin. Diagn. Lab. Immun.* **2005,** *12*, 837-844,doi:10.1128/Cdli.12.7.837-844.2005
36. Buhimschi, I. A.; Christner, R.; Buhimschi, C. S. Proteomic biomarker analysis of amniotic fluid for identification of intra-amniotic inflammation. *Bjog-Int. J. Obstet. Gy.* **2005,** *112*, 173-181,doi:10.1111/j.1471-0528.2004.00340.x
37. Buhimschi, I. A.; Zambrano, E.; Pettker, C. M.; Bahtiyar, M. O.; Paidas, M.; Rosenberg, V. A.; Thung, S.; Salafia, C. M.; Buhimschi, C. S. Using proteomic analysis of the human amniotic fluid to identify histologic chorioamnionitis. *Obstet. Gynecol.* **2008,** *111*, 403-412,doi:DOI 10.1097/AOG.0b013e31816102aa

38. Buhimschi, I. A.; Zhao, G.; Rosenberg, V. A.; Abdel-Razeq, S.; Thung, S.; Buhimschi, C. S. Multidimensional Proteomics Analysis of Amniotic Fluid to Provide Insight into the Mechanisms of Idiopathic Preterm Birth. *Plos One* **2008,** *3*,doi:10.1371/journal.pone.0002049
39. Bujold, E.; Romero, R.; Kusanovic, J. P.; Erez, O.; Gotsch, F.; Chaiworapongsa, T.; Gomez, R.; Espinoza, J.; Vaisbuch, E.; Kim, Y. M., et al. Proteomic profiling of amniotic fluid in preterm labor using two-dimensional liquid separation and mass spectrometry. *J. Matern.-Fetal Neo. M.* **2008,** *21*, 697-713,doi:10.1080/14767050802053289
40. Cecconi, D.; Lonardoni, F.; Favretto, D.; Cosmi, E.; Tucci, M.; Visentin, S.; Cecchetto, G.; Fais, P.; Viel, G.; Ferrara, S. D. Changes in amniotic fluid and umbilical cord serum proteomic profiles of foetuses with intrauterine growth retardation. *Electrophoresis* **2011,** *32*, 3630-3637,doi:10.1002/elps.201100256
41. Cen, J.; Lv, L.; Wei, Y.; Deng, L.; Huang, L.; Deng, X.; Qin, Q.; Sun, Y.; Pang, L. Comparative proteome analysis of amniotic fluids and placentas from patients with idiopathic polyhydramnios. *Placenta* **2020,** *89*, 67-77,doi:10.1016/j.placenta.2019.10.010
42. Chen, C. P.; Lai, T. C.; Chern, S. R.; Li, S. H.; Chou, H. C.; Chen, Y. W.; Lin, S. T.; Lu, Y. C.; Wu, C. L.; Li, J. M., et al. Proteome differences between male and female fetal cells in amniotic fluid. *OMICS* **2013,** *17*, 16-26,doi:10.1089/omi.2010.0145
43. Chen, W. Q.; Siegel, N.; Li, L.; Pollak, A.; Hengstschlager, M.; Lubec, G. Variations of protein levels in human amniotic fluid stem cells CD117/2 over passages 5-25. *J. Proteome Res.* **2009,** *8*, 5285-95,doi:10.1021/pr900630s
44. Cheng, P. J.; Wang, T. H.; Huang, S. Y.; Kao, C. C.; Lu, J. H.; Hsiao, C. H.; Shaw, S. W. Differential proteomics analysis of amniotic fluid in pregnancies of increased nuchal translucency with normal karyotype. *Prenat. Diagn.* **2011,** *31*, 274-81,doi:10.1002/pd.2719
45. Chevalier, R. L. Biomarkers of congenital obstructive nephropathy: past, present and future. *J. Urol.* **2004,** *172*, 852-7,doi:10.1097/01.ju.0000129542.22043.ef
46. Chevalier, R. L. Prognostic factors and biomarkers of congenital obstructive nephropathy. *Pediatr. Nephrol.* **2016,** *31*, 1411-20,doi:10.1007/s00467-015-3291-3
47. Cho, C. K.; Diamandis, E. P. Application of proteomics to prenatal screening and diagnosis for aneuploidies. *Clin. Chem. Lab. Med.* **2011,** *49*, 33-41,doi:10.1515/CCLM.2011.002
48. Cho, C. K.; Drabovich, A. P.; Batruch, I.; Diamandis, E. P. Verification of a biomarker discovery approach for detection of Down syndrome in amniotic fluid via multiplex selected reaction monitoring (SRM) assay. *J. Proteomics* **2011,** *74*, 2052-9,doi:10.1016/j.jprot.2011.05.025
49. Cho, C. K.; Smith, C. R.; Diamandis, E. P. Amniotic fluid proteome analysis from Down syndrome pregnancies for biomarker discovery. *J. Proteome Res.* **2010,** *9*, 3574-82,doi:10.1021/pr100088k
50. Choolani, M.; Narasimhan, K.; Kolla, V.; Hahn, S. Proteomic technologies for prenatal diagnostics: advances and challenges ahead. *Expert Rev. Proteomics* **2009,** *6*, 87-101,doi:10.1586/14789450.6.1.87
51. Cobo, T.; Palacio, M.; Grande, M.; Sanchez-Garcia, A. B.; Estanyol, J. M.; Lopez, M.; Bosch, J.; Marti, C.; Gratacos, E. Cervical Alpha-Actinin-4 Is Upregulated in Women with Threatened Preterm Labor and Microbial Invasion of the Amniotic Cavity. *Fetal Diagn. Ther.* **2018,** *44*, 36-43,doi:10.1159/000478259
52. Cobo, T.; Palacio, M.; Navarro-Sastre, A.; Ribes, A.; Bosch, J.; Filella, X.; Gratacos, E. Predictive value of combined amniotic fluid proteomic biomarkers and interleukin-6 in preterm labor with intact membranes. *Am. J. Obstet. Gynecol.* **2009,** *200*, 499 e1-6,doi:10.1016/j.ajog.2008.12.036
53. Conde-Agudelo, A.; Papageorghiou, A. T.; Kennedy, S. H.; Villar, J. Novel biomarkers for the prediction of the spontaneous preterm birth phenotype: a systematic review and meta-analysis. *BJOG* **2011,** *118*, 1042-54,doi:10.1111/j.1471-0528.2011.02923.x
54. Consonni, S.; Mainini, V.; Pizzardi, A.; Gianazza, E.; Chinello, C.; Locatelli, A.; Magni, F. Non-invasively collected amniotic fluid as a source of possible biomarkers for premature rupture of membranes investigated by proteomic approach. *Arch. Gynecol. Obstet.* **2014,** *289*, 299-306,doi:10.1007/s00404-013-2967-9
55. Crawford, J. T.; Pereira, L.; Buckmaster, J.; Gravett, M. G.; Tolosa, J. E. Amniocentesis results and novel proteomic analysis in a case of occult candidal chorioamnionitis. *J. Matern. Fetal Neonatal Med.* **2006,** *19*, 667-70,doi:10.1080/14767050600738289
56. Croitor-Sava, A.; Beck, V.; Sandaite, I.; Van Huffel, S.; Dresselaers, T.; Claus, F.; Himmelreich, U.; Deprest, J. High-Resolution (1)H NMR Spectroscopy Discriminates Amniotic Fluid of Fetuses with Congenital Diaphragmatic Hernia from Healthy Controls. *J. Proteome Res.* **2015,** *14*, 4502-10,doi:10.1021/acs.jproteome.5b00131
57. Cruickshank, J. K.; Mbanya, J. C.; Wilks, R.; Balkau, B.; McFarlane-Anderson, N.; Forrester, T. Sick genes, sick individuals or sick populations with chronic disease? The emergence of diabetes and high blood pressure in African-origin populations. *Int. J. Epidemiol.* **2001,** *30*, 111-7,doi:10.1093/ije/30.1.111

58. Da Silva, M.; Dombre, C.; Brionne, A.; Monget, P.; Chesse, M.; De Pauw, M.; Mills, M.; Combes-Soia, L.; Labas, V.; Guyot, N., et al. The Unique Features of Proteins Depicting the Chicken Amniotic Fluid. *Mol. Cell Proteomics* **2019,** *18*, S174-S190,doi:10.1074/mcp.RA117.000459
59. Dasari, S.; Pereira, L.; Reddy, A. P.; Michaels, J. E.; Lu, X.; Jacob, T.; Thomas, A.; Rodland, M.; Roberts, C. T., Jr.; Gravett, M. G., et al. Comprehensive proteomic analysis of human cervical-vaginal fluid. *J. Proteome Res.* **2007,** *6*, 1258-68,doi:10.1021/pr0605419
60. De Seta, F.; Banco, R.; Guaschino, S.; De Santo, D.; Turrisi, A.; Piva, C. [Proteomic applications in gynecology-obstetrics]. *Minerva Ginecol.* **2011,** *63*, 39-46
61. Delanghe, J. R.; Speeckaert, M. M. Translational research and biomarkers in neonatal sepsis. *Clin. Chim. Acta* **2015,** *451*, 46-64,doi:10.1016/j.cca.2015.01.031
62. Deng, B.; Dong, Z.; Liu, Y.; Wang, C.; Liu, J.; Wang, C.; Qu, X. Effects of pretreatment protocols on human amniotic fluid protein profiling with SELDI-TOF MS using protein chips and magnetic beads. *Clin. Chim. Acta* **2010,** *411*, 1051-7,doi:10.1016/j.cca.2010.03.036
63. Di Venere, M.; Viglio, S.; Cagnone, M.; Bardoni, A.; Salvini, R.; Iadarola, P. Advances in the analysis of "less-conventional" human body fluids: An overview of the CE- and HPLC-MS applications in the years 2015-2017. *Electrophoresis* **2018,** *39*, 160-178,doi:10.1002/elps.201700276
64. Dixon, C. L.; Sheller-Miller, S.; Saade, G. R.; Fortunato, S. J.; Lai, A.; Palma, C.; Guanzon, D.; Salomon, C.; Menon, R. Amniotic Fluid Exosome Proteomic Profile Exhibits Unique Pathways of Term and Preterm Labor. *Endocrinology* **2018,** *159*, 2229-2240,doi:10.1210/en.2018-00073
65. Dudzik, D.; Revello, R.; Barbas, C.; Bartha, J. L. LC-MS-based metabolomics identification of novel biomarkers of chorioamnionitis and its associated perinatal neurological damage. *J. Proteome Res.* **2015,** *14*, 1432-44,doi:10.1021/pr501087x
66. Fotopoulou, C.; Kyeyamwa, S.; Linder, M.; Thieme, D.; Hartenstein, S.; Klein, O.; Dudenhausen, J. W.; Henrich, W.; Kalache, K. D.; Bamberg, C. Proteomic analysis of midtrimester amniotic fluid to identify novel biomarkers for preterm delivery. *J. Matern. Fetal Neonatal Med.* **2012,** *25*, 2488-93,doi:10.3109/14767058.2012.712565
67. Fu, W. Y.; Xu, L. H.; Yu, Y. N. Proteomic analysis of cellular response to microcystin in human amnion FL cells. *J. Proteome Res.* **2005,** *4*, 2207-15,doi:10.1021/pr050325k
68. Gianazza, E.; Miller, I.; Guerrini, U.; Palazzolo, L.; Parravicini, C.; Eberini, I. Gender proteomics II. Which proteins in sexual organs. *J. Proteomics* **2018,** *178*, 18-30,doi:10.1016/j.jprot.2017.10.001
69. Gianazza, E.; Wait, R.; Begum, S.; Eberini, I.; Campagnoli, M.; Labo, S.; Galliano, M. Mapping the 5-50-kDa fraction of human amniotic fluid proteins by 2-DE and ESI-MS. *Proteomics Clin. Appl.* **2007,** *1*, 167-75,doi:10.1002/prca.200600543
70. Gonzalez-Buitrago, J. M.; Ferreira, L.; Muniz, M. C. [Clinical proteomics and new biomarkers in biological fluids]. *Med. Clin. (Barc)* **2008,** *131*, 426-34,doi:10.1157/13126219
71. Govia, R. N. M.; Birse, K. D.; Sepehri, S.; Khafipour, E.; Menticoglou, S. M.; Burgener, A. D.; Poliquin, V. Amniotic fluid proteomic signatures of cervical insufficiency and their association with length of latency. *Am. J. Reprod. Immunol.* **2018,** *80*, e13030,doi:10.1111/aji.13030
72. Graca, G.; Duarte, I. F.; Barros, A. S.; Goodfellow, B. J.; Diaz, S.; Carreira, I. M.; Couceiro, A. B.; Galhano, E.; Gil, A. M. (1)H NMR based metabonomics of human amniotic fluid for the metabolic characterization of fetus malformations. *J. Proteome Res.* **2009,** *8*, 4144-50,doi:10.1021/pr900386f
73. Graca, G.; Duarte, I. F.; Barros, A. S.; Goodfellow, B. J.; Diaz, S. O.; Pinto, J.; Carreira, I. M.; Galhano, E.; Pita, C.; Gil, A. M. Impact of prenatal disorders on the metabolic profile of second trimester amniotic fluid: a nuclear magnetic resonance metabonomic study. *J. Proteome Res.* **2010,** *9*, 6016-24,doi:10.1021/pr100815q
74. Gravett, M. G.; Novy, M. J.; Rosenfeld, R. G.; Reddy, A. P.; Jacob, T.; Turner, M.; McCormack, A.; Lapidus, J. A.; Hitti, J.; Eschenbach, D. A., et al. Diagnosis of intra-amniotic infection by proteomic profiling and identification of novel biomarkers. *JAMA* **2004,** *292*, 462-9,doi:10.1001/jama.292.4.462
75. Hallingstrom, M.; Lenco, J.; Vajrychova, M.; Link, M.; Tambor, V.; Liman, V.; Bullarbo, M.; Nilsson, S.; Tsiartas, P.; Cobo, T., et al. Proteomic Analysis of Early Mid-Trimester Amniotic Fluid Does Not Predict Spontaneous Preterm Delivery. *Plos One* **2016,** *11*, e0155164,doi:10.1371/journal.pone.0155164
76. Hampton, T. Comprehensive "proteomic profile" of amniotic fluid may aid prenatal diagnosis. *JAMA* **2007,** *298*, 1751,doi:10.1001/jama.298.15.1751
77. Han, Y. W.; Shen, T.; Chung, P.; Buhimschi, I. A.; Buhimschi, C. S. Uncultivated bacteria as etiologic agents of intra-amniotic inflammation leading to preterm birth. *J. Clin. Microbiol.* **2009,** *47*, 38-47,doi:10.1128/JCM.01206-08
78. Hassan, M. I.; Kumar, V.; Singh, T. P.; Yadav, S. Proteomic analysis of human amniotic fluid from Rh(-) pregnancy. *Prenat. Diagn.* **2008,** *28*, 102-8,doi:10.1002/pd.1941

79. Hitti, J.; Lapidus, J. A.; Lu, X.; Reddy, A. P.; Jacob, T.; Dasari, S.; Eschenbach, D. A.; Gravett, M. G.; Nagalla, S. R. Noninvasive diagnosis of intraamniotic infection: proteomic biomarkers in vaginal fluid. *Am. J. Obstet. Gynecol.* **2010,** *203*, 32 e1-8,doi:10.1016/j.ajog.2010.03.037
80. Hsu, T. Y.; Lin, H.; Hung, H. N.; Yang, K. D.; Ou, C. Y.; Tsai, C. C.; Cheng, H. H.; Chung, S. H.; Cheng, B. H.; Wong, Y. H., et al. Two-Dimensional Differential Gel Electrophoresis to Identify Protein Biomarkers in Amniotic Fluid of Edwards Syndrome (Trisomy 18) Pregnancies. *Plos One* **2016,** *11*, e0145908,doi:10.1371/journal.pone.0145908
81. Hu, S.; Loo, J. A.; Wong, D. T. Human body fluid proteome analysis. *Proteomics* **2006,** *6*, 6326-53,doi:10.1002/pmic.200600284
82. Hui, L.; Bianchi, D. W. Cell-free fetal nucleic acids in amniotic fluid. *Hum. Reprod. Update* **2011,** *17*, 362-71,doi:10.1093/humupd/dmq049
83. Iadarola, P.; Fumagalli, M.; Bardoni, A. M.; Salvini, R.; Viglio, S. Recent applications of CE- and HPLC-MS in the analysis of human fluids. *Electrophoresis* **2016,** *37*, 212-30,doi:10.1002/elps.201500272
84. Isani, G.; Ferlizza, E.; Cuoghi, A.; Bellei, E.; Monari, E.; Bianchin Butina, B.; Castagnetti, C. Identification of the most abundant proteins in equine amniotic fluid by a proteomic approach. *Anim. Reprod. Sci.* **2016,** *174*, 150-160,doi:10.1016/j.anireprosci.2016.10.003
85. Joo, W. A.; Lee, D. Y.; Kim, C. W. Development of an effective sample preparation method for the proteome analysis of body fluids using 2-D gel electrophoresis. *Biosci. Biotechnol. Biochem.* **2003,** *67*, 1574-7,doi:10.1271/bbb.67.1574
86. Kamath-Rayne, B. D.; Smith, H. C.; Muglia, L. J.; Morrow, A. L. Amniotic fluid: the use of high-dimensional biology to understand fetal well-being. *Reprod. Sci.* **2014,** *21*, 6-19,doi:10.1177/1933719113485292
87. Klein, J.; Buffin-Meyer, B.; Mullen, W.; Carty, D. M.; Delles, C.; Vlahou, A.; Mischak, H.; Decramer, S.; Bascands, J. L.; Schanstra, J. P. Clinical proteomics in obstetrics and neonatology. *Expert Rev. Proteomics* **2014,** *11*, 75-89,doi:10.1586/14789450.2014.872564
88. Kolialexi, A.; Anagnostopoulos, A. K.; Mavrou, A.; Tsangaris, G. T. Application of proteomics for diagnosis of fetal aneuploidies and pregnancy complications. *J. Proteomics* **2009,** *72*, 731-9,doi:10.1016/j.jprot.2009.03.005
89. Kolialexi, A.; Mavrou, A.; Spyrou, G.; Tsangaris, G. T. Mass spectrometry-based proteomics in reproductive medicine. *Mass Spectrom Rev.* **2008,** *27*, 624-34,doi:10.1002/mas.20181
90. Kolialexi, A.; Mavrou, A.; Tsangaris, G. T. Proteomic analysis of human reproductive fluids. *Proteomics Clin. Appl.* **2007,** *1*, 853-60,doi:10.1002/prca.200700040
91. Kolialexi, A.; Tounta, G.; Mavrou, A.; Tsangaris, G. T. Proteomic analysis of amniotic fluid for the diagnosis of fetal aneuploidies. *Expert Rev. Proteomics* **2011,** *8*, 175-85,doi:10.1586/epr.10.112
92. Kolialexi, A.; Tsangaris, G. T.; Mavrou, A. Proteomics in prenatal diagnosis. *Expert Rev. Proteomics* **2009,** *6*, 111-3,doi:10.1586/epr.09.6
93. Kossowska, B.; Dudka, I.; Gancarz, R.; Antonowicz-Juchniewicz, J., [Proteomic analysis of protein profiles in some pathological stages of the human organism]. In *Postepy Hig. Med. Dosw. (Online)*, 2009/11/27 ed.; 2009; Vol. 63, pp 549-63.
94. Law, K. P.; Han, T. L.; Tong, C.; Baker, P. N. Mass spectrometry-based proteomics for pre-eclampsia and preterm birth. *Int. J. Mol. Sci.* **2015,** *16*, 10952-85,doi:10.3390/ijms160510952
95. Lee, J.; Park, J. S.; Norwitz, E. R.; Kim, B. J.; Park, C. W.; Jun, J. K.; Syn, H. C. Identification and characterization of proteins in amniotic fluid that are differentially expressed before and after antenatal corticosteroid administration. *Am. J. Obstet. Gynecol.* **2010,** *202*, 388 e1-388 e10,doi:10.1016/j.ajog.2010.01.056
96. Nath, A. K.; Krauthammer, M.; Li, P.; Davidov, E.; Butler, L. C.; Copel, J.; Katajamaa, M.; Oresic, M.; Buhimschi, I.; Buhimschi, C., et al. Proteomic-based detection of a protein cluster dysregulated during cardiovascular development identifies biomarkers of congenital heart defects. *Plos One* **2009,** *4*, e4221,doi:10.1371/journal.pone.0004221
97. Liu, H.; Shen, J.; Feng, L.; Yu, Y. Low concentration of anti-7,8-dihydroxy-9,10-epoxy-7,8,9,10-tetrahydrobenzo[a]pyrene induces alterations of extracellular protein profile of exposed epithelial cells. *Proteomics* **2009,** *9*, 4259-64,doi:10.1002/pmic.200700886
98. Liu, H.; Wang, H.; Zhu, H.; Zhang, H.; Liu, S. Preliminary study of protein changes in trisomy 21 fetus by proteomics analysis in amniocyte. *Prenat. Diagn.* **2018,** *38*, 435-444,doi:10.1002/pd.5259
99. Liu, X.; Song, Y.; Guo, Z.; Sun, W.; Liu, J. A comprehensive profile and inter-individual variations analysis of the human normal amniotic fluid proteome. *J. Proteomics* **2019,** *192*, 1-9,doi:10.1016/j.jprot.2018.04.023
100. Liu, Z.; Yuan, Z.; Zhao, Q. SELDI-TOF-MS proteomic profiling of serum, urine, and amniotic fluid in neural tube defects. *Plos One* **2014,** *9*, e103276,doi:10.1371/journal.pone.0103276

101. Lygirou, V.; Makridakis, M.; Vlahou, A. Biological sample collection for clinical proteomics: existing SOPs. *Methods Mol. Biol.* **2015,** *1243*, 3-27,doi:10.1007/978-1-4939-1872-0_1
102. Makridakis, M.; Roubelakis, M. G.; Vlahou, A. Stem cells: insights into the secretome. *Biochim. Biophys. Acta* **2013,** *1834*, 2380-4,doi:10.1016/j.bbapap.2013.01.032
103. Martinez-Morillo, E.; Cho, C. K.; Drabovich, A. P.; Shaw, J. L.; Soosaipillai, A.; Diamandis, E. P. Development of a multiplex selected reaction monitoring assay for quantification of biochemical markers of down syndrome in amniotic fluid samples. *J. Proteome Res.* **2012,** *11*, 3880-7,doi:10.1021/pr300355a
104. Mavrou, A.; Anagnostopoulos, A. K.; Kolialexi, A.; Vougas, K.; Papantoniou, N.; Antsaklis, A.; Fountoulakis, M.; Tsangaris, G. T. Proteomic analysis of amniotic fluid in pregnancies with Turner syndrome fetuses. *J. Proteome Res.* **2008,** *7*, 1862-6,doi:10.1021/pr700588u
105. Mellows, B.; Mitchell, R.; Antonioli, M.; Kretz, O.; Chambers, D.; Zeuner, M. T.; Denecke, B.; Musante, L.; Ramachandra, D. L.; Debacq-Chainiaux, F., et al. Protein and Molecular Characterization of a Clinically Compliant Amniotic Fluid Stem Cell-Derived Extracellular Vesicle Fraction Capable of Accelerating Muscle Regeneration Through Enhancement of Angiogenesis. *Stem Cells Dev.* **2017,** *26*, 1316-1333,doi:10.1089/scd.2017.0089
106. Miceli, M.; Dell'Aversana, C.; Russo, R.; Rega, C.; Cupelli, L.; Ruvo, M.; Altucci, L.; Chambery, A. Secretome profiling of cytokines and growth factors reveals that neuro-glial differentiation is associated with the down-regulation of Chemokine Ligand 2 (MCP-1/CCL2) in amniotic fluid derived-mesenchymal progenitor cells. *Proteomics* **2016,** *16*, 674-88,doi:10.1002/pmic.201500223
107. Michaels, J. E.; Dasari, S.; Pereira, L.; Reddy, A. P.; Lapidus, J. A.; Lu, X.; Jacob, T.; Thomas, A.; Rodland, M.; Roberts, C. T., Jr., et al. Comprehensive proteomic analysis of the human amniotic fluid proteome: gestational age-dependent changes. *J. Proteome Res.* **2007,** *6*, 1277-85,doi:10.1021/pr060543t
108. Michel, P. E.; Crettaz, D.; Morier, P.; Heller, M.; Gallot, D.; Tissot, J. D.; Reymond, F.; Rossier, J. S. Proteome analysis of human plasma and amniotic fluid by Off-Gel isoelectric focusing followed by nano-LC-MS/MS. *Electrophoresis* **2006,** *27*, 1169-81,doi:10.1002/elps.200500680
109. Montemurro, T.; Andriolo, G.; Montelatici, E.; Weissmann, G.; Crisan, M.; Colnaghi, M. R.; Rebulla, P.; Mosca, F.; Peault, B.; Lazzari, L. Differentiation and migration properties of human foetal umbilical cord perivascular cells: potential for lung repair. *J. Cell. Mol. Med.* **2011,** *15*, 796-808,doi:10.1111/j.1582-4934.2010.01047.x
110. Mukhopadhyay, R. Biomarkers for preterm birth. *J. Proteome Res.* **2005,** *4*, 1900,doi:10.1021/pr050532k
111. Musilova, I.; Kacerovsky, M.; Tambor, V.; Tosner, J. [Proteomics and biomarkers for detection of preterm labor: a systematic review]. *Ceska Gynekol.* **2011,** *76*, 37-45
112. Nakayama, A. [Proteomic analysis of urinary exosomes]. *Rinsho Byori* **2014,** *62*, 684-91
113. Nardelli, C.; Granata, I.; Iaffaldano, L.; D'Argenio, V.; Del Monaco, V.; Maruotti, G. M.; Del Vecchio, L.; Martinelli, P.; Salvatore, F.; Guarracino, M. R., et al. Sex-Comparative Analysis of the miRNome of Human Amniotic Mesenchymal Stem Cells During Obesity. *Stem Cells Dev.* **2017,** *26*, 1-3,doi:10.1089/scd.2016.0134
114. Nilsson, S.; Ramstrom, M.; Palmblad, M.; Axelsson, O.; Bergquist, J. Explorative study of the protein composition of amniotic fluid by liquid chromatography electrospray ionization Fourier transform ion cyclotron resonance mass spectrometry. *J Proteome Res.* **2004,** *3*, 884-9,doi:10.1021/pr0499545
115. Nissum, M.; Abu Shehab, M.; Sukop, U.; Khosravi, J. M.; Wildgruber, R.; Eckerskorn, C.; Han, V. K.; Gupta, M. B. Functional and complementary phosphorylation state attributes of human insulin-like growth factor-binding protein-1 (IGFBP-1) isoforms resolved by free flow electrophoresis. *Mol. Cell. Proteomics* **2009,** *8*, 1424-35,doi:10.1074/mcp.M800571-MCP200
116. Ogge, G.; Romero, R.; Lee, D. C.; Gotsch, F.; Than, N. G.; Lee, J.; Chaiworapongsa, T.; Dong, Z.; Mittal, P.; Hassan, S. S., et al. Chronic chorioamnionitis displays distinct alterations of the amniotic fluid proteome. *J. Pathol.* **2011,** *223*, 553-65,doi:10.1002/path.2825
117. Park, J.; Cha, D. H.; Jung, J. W.; Kim, Y. H.; Lee, S. H.; Kim, Y.; Kim, K. P. Comparative proteomic analysis of human amniotic fluid supernatants with Down syndrome using mass spectrometry. *J. Microbiol. Biotechnol.* **2010,** *20*, 959-67,doi:10.4014/jmb.0912.12035
118. Park, J. S.; Oh, K. J.; Norwitz, E. R.; Han, J. S.; Choi, H. J.; Seong, H. S.; Kang, Y. D.; Park, C. W.; Kim, B. J.; Jun, J. K., et al. Identification of proteomic biomarkers of preeclampsia in amniotic fluid using SELDI-TOF mass spectrometry. *Reprod. Sci.* **2008,** *15*, 457-68,doi:10.1177/1933719108316909
119. Park, S. J.; Yoon, W. G.; Song, J. S.; Jung, H. S.; Kim, C. J.; Oh, S. Y.; Yoon, B. H.; Jung, G.; Kim, H. J.; Nirasawa, T. Proteome analysis of human amnion and amniotic fluid by two-dimensional electrophoresis and matrix-assisted laser desorption/ionization time-of-flight mass spectrometry. *Proteomics* **2006,** *6*, 349-63,doi:10.1002/pmic.200500084

120. Perluigi, M.; Di Domenico, F.; Buttterfield, D. A. Unraveling the complexity of neurodegeneration in brains of subjects with Down syndrome: insights from proteomics. *Proteomics Clin. Appl.* **2014,** *8*, 73-85,doi:10.1002/prca.201300066
121. Perluigi, M.; di Domenico, F.; Fiorini, A.; Cocciolo, A.; Giorgi, A.; Foppoli, C.; Butterfield, D. A.; Giorlandino, M.; Giorlandino, C.; Schinina, M. E., et al. Oxidative stress occurs early in Down syndrome pregnancy: A redox proteomics analysis of amniotic fluid. *Proteomics Clin. Appl.* **2011,** *5*, 167-78,doi:10.1002/prca.201000121
122. Pettker, C. M.; Buhimschi, I. A.; Magloire, L. K.; Sfakianaki, A. K.; Hamar, B. D.; Buhimschi, C. S. Value of placental microbial evaluation in diagnosing intra-amniotic infection. *Obstet. Gynecol.* **2007,** *109*, 739-49,doi:10.1097/01.AOG.0000255663.47512.23
123. Pipino, C.; Pierdomenico, L.; Di Tomo, P.; Di Giuseppe, F.; Cianci, E.; D'Alimonte, I.; Morabito, C.; Centurione, L.; Antonucci, I.; Mariggio, M. A., et al. Molecular and phenotypic characterization of human amniotic fluid-derived cells: a morphological and proteomic approach. *Stem Cells Dev.* **2015,** *24*, 1415-28,doi:10.1089/scd.2014.0453
124. Pogorelova, T. N.; Orlov, V. I.; Gunko, V. O. New approaches to molecular diagnostics of prenatal pathology. *Bull. Exp. Biol. Med.* **2011,** *151*, 567-70,doi:10.1007/s10517-011-1384-y
125. Queloz, P. A.; Crettaz, D.; Thadikkaran, L.; Sapin, V.; Gallot, D.; Jani, J.; Deprest, J.; Lemery, D.; Barelli, S.; Tissot, J. D. Proteomic analyses of amniotic fluid: potential applications in health and diseases. *J. Chromatogr. B Analyt. Technol. Biomed. Life Sci.* **2007,** *850*, 336-42,doi:10.1016/j.jchromb.2006.12.006
126. Raimondo, F.; Morosi, L.; Chinello, C.; Magni, F.; Pitto, M. Advances in membranous vesicle and exosome proteomics improving biological understanding and biomarker discovery. *Proteomics* **2011,** *11*, 709-20,doi:10.1002/pmic.201000422
127. Ramstrom, M.; Bergquist, J. Miniaturized proteomics and peptidomics using capillary liquid separation and high resolution mass spectrometry. *FEBS Lett* **2004,** *567*, 92-5,doi:10.1016/j.febslet.2004.04.074
128. Ridsdale, R.; Lewis, D. F.; Weaver, T. E.; Akinbi, H. T. Proteomic analysis of lamellar bodies isolated from amniotic fluid: implications for function. *Am. J. Perinatol.* **2012,** *29*, 419-28,doi:10.1055/s-0032-1304822
129. Romani, R.; Fallarino, F.; Pirisinu, I.; Calvitti, M.; Caselli, A.; Fiaschi, T.; Gamberi, T.; Matino, D.; Talesa, V. N.; Donti, E., et al. Comparative proteomic analysis of two distinct stem-cell populations from human amniotic fluid. *Mol. Biosyst.* **2015,** *11*, 1622-32,doi:10.1039/c5mb00018a
130. Romero, R.; Espinoza, J.; Gotsch, F.; Kusanovic, J. P.; Friel, L. A.; Erez, O.; Mazaki-Tovi, S.; Than, N. G.; Hassan, S.; Tromp, G. The use of high-dimensional biology (genomics, transcriptomics, proteomics, and metabolomics) to understand the preterm parturition syndrome. *BJOG* **2006,** *113 Suppl 3*, 118-35,doi:10.1111/j.1471-0528.2006.01150.x
131. Romero, R.; Espinoza, J.; Rogers, W. T.; Moser, A.; Nien, J. K.; Kusanovic, J. P.; Gotsch, F.; Erez, O.; Gomez, R.; Edwin, S., et al. Proteomic analysis of amniotic fluid to identify women with preterm labor and intra-amniotic inflammation/infection: the use of a novel computational method to analyze mass spectrometric profiling. *J. Matern. Fetal Neonatal Med.* **2008,** *21*, 367-88,doi:10.1080/14767050802045848
132. Romero, R.; Kusanovic, J. P.; Gotsch, F.; Erez, O.; Vaisbuch, E.; Mazaki-Tovi, S.; Moser, A.; Tam, S.; Leszyk, J.; Master, S. R., et al. Isobaric labeling and tandem mass spectrometry: a novel approach for profiling and quantifying proteins differentially expressed in amniotic fluid in preterm labor with and without intra-amniotic infection/inflammation. *J. Matern. Fetal Neonatal Med.* **2010,** *23*, 261-80,doi:10.3109/14767050903067386
133. Romero, R.; Mazaki-Tovi, S.; Vaisbuch, E.; Kusanovic, J. P.; Chaiworapongsa, T.; Gomez, R.; Nien, J. K.; Yoon, B. H.; Mazor, M.; Luo, J., et al. Metabolomics in premature labor: a novel approach to identify patients at risk for preterm delivery. *J. Matern. Fetal Neonatal Med.* **2010,** *23*, 1344-59,doi:10.3109/14767058.2010.482618
134. Roubelakis, M. G.; Pappa, K. I.; Bitsika, V.; Zagoura, D.; Vlahou, A.; Papadaki, H. A.; Antsaklis, A.; Anagnou, N. P. Molecular and proteomic characterization of human mesenchymal stem cells derived from amniotic fluid: comparison to bone marrow mesenchymal stem cells. *Stem Cells Dev.* **2007,** *16*, 931-52,doi:10.1089/scd.2007.0036
135. Roubelakis, M. G.; Trohatou, O.; Roubelakis, A.; Mili, E.; Kalaitzopoulos, I.; Papazoglou, G.; Pappa, K. I.; Anagnou, N. P. Platelet-rich plasma (PRP) promotes fetal mesenchymal stem/stromal cell migration and wound healing process. *Stem Cell Rev. Rep.* **2014,** *10*, 417-28,doi:10.1007/s12015-013-9494-8
136. Roubelakis, M. G.; Tsaknakis, G.; Pappa, K. I.; Anagnou, N. P.; Watt, S. M. Spindle shaped human mesenchymal stem/stromal cells from amniotic fluid promote neovascularization. *Plos One* **2013,** *8*, e54747,doi:10.1371/journal.pone.0054747
137. Ruetschi, U.; Rosen, A.; Karlsson, G.; Zetterberg, H.; Rymo, L.; Hagberg, H.; Jacobsson, B. Proteomic analysis using protein chips to detect biomarkers in cervical and amniotic fluid in women with intra-amniotic inflammation. *J. Proteome Res.* **2005,** *4*, 2236-42,doi:10.1021/pr050139e

138. Savickiene, J.; Treigyte, G.; Baronaite, S.; Valiuliene, G.; Kaupinis, A.; Valius, M.; Arlauskiene, A.; Navakauskiene, R. Human Amniotic Fluid Mesenchymal Stem Cells from Second- and Third-Trimester Amniocentesis: Differentiation Potential, Molecular Signature, and Proteome Analysis. *Stem Cells Int.* **2015,** *2015*, 319238,doi:10.1155/2015/319238
139. Shtam, T. A.; Burdakov, V. S.; Landa, S. B.; Naryzhny, S. N.; Bairamukov, V. Y.; Malek, A. V.; Orlov, Y. N.; Filatov, M. V. Aggregation by Lectin-Methodical Approach for Effective Isolation of Exosomes from Cell Culture Supernatant for Proteome Profiling. *Tsitologiia* **2017,** *59*, 5-12
140. Simpson, R. J.; Jensen, S. S.; Lim, J. W. Proteomic profiling of exosomes: current perspectives. *Proteomics* **2008,** *8*, 4083-99,doi:10.1002/pmic.200800109
141. Simpson, R. J.; Lim, J. W.; Moritz, R. L.; Mathivanan, S. Exosomes: proteomic insights and diagnostic potential. *Expert Rev. Proteomics* **2009,** *6*, 267-83,doi:10.1586/epr.09.17
142. Spivak, M.; Weston, J.; Tomazela, D.; MacCoss, M. J.; Noble, W. S. Direct maximization of protein identifications from tandem mass spectra. *Mol. Cell. Proteomics* **2012,** *11*, M111 012161,doi:10.1074/mcp.M111.012161
143. Stawicki, S. P.; Papadimos, T. J. Challenges in managing amniotic fluid embolism: an up-to-date perspective on diagnostic testing with focus on novel biomarkers and avenues for future research. *Curr. Pharm. Biotechnol.* **2014,** *14*, 1168-78,doi:10.2174/1389201015666140430163921
144. Tambor, V.; Hunter, C. L.; Seymour, S. L.; Kacerovsky, M.; Stulik, J.; Lenco, J. CysTRAQ - A combination of iTRAQ and enrichment of cysteinyl peptides for uncovering and quantifying hidden proteomes. *J. Proteomics* **2012,** *75*, 857-67,doi:10.1016/j.jprot.2011.09.027
145. Tambor, V.; Kacerovsky, M.; Andrys, C.; Musilova, I.; Hornychova, H.; Pliskova, L.; Link, M.; Stulik, J.; Lenco, J. Amniotic fluid cathelicidin in PPROM pregnancies: from proteomic discovery to assessing its potential in inflammatory complications diagnosis. *Plos One* **2012,** *7*, e41164,doi:10.1371/journal.pone.0041164
146. Tambor, V.; Kacerovsky, M.; Lenco, J.; Bhat, G.; Menon, R. Proteomics and bioinformatics analysis reveal underlying pathways of infection associated histologic chorioamnionitis in pPROM. *Placenta* **2013,** *34*, 155-61,doi:10.1016/j.placenta.2012.11.028
147. Tambor, V.; Vajrychova, M.; Kacerovsky, M.; Link, M.; Domasinska, P.; Menon, R.; Lenco, J. Potential Peripartum Markers of Infectious-Inflammatory Complications in Spontaneous Preterm Birth. *Biomed Res. Int.* **2015,** *2015*, 343501,doi:10.1155/2015/343501
148. Thadikkaran, L.; Crettaz, D.; Siegenthaler, M. A.; Gallot, D.; Sapin, V.; Iozzo, R. V.; Queloz, P. A.; Schneider, P.; Tissot, J. D. The role of proteomics in the assessment of premature rupture of fetal membranes. *Clin. Chim. Acta* **2005,** *360*, 27-36,doi:10.1016/j.cccn.2005.04.018
149. Tsangaris, G.; Weitzdorfer, R.; Pollak, D.; Lubec, G.; Fountoulakis, M. The amniotic fluid cell proteome. *Electrophoresis* **2005,** *26*, 1168-73,doi:10.1002/elps.200406183
150. Tsangaris, G. T.; Karamessinis, P.; Kolialexi, A.; Garbis, S. D.; Antsaklis, A.; Mavrou, A.; Fountoulakis, M. Proteomic analysis of amniotic fluid in pregnancies with Down syndrome. *Proteomics* **2006,** *6*, 4410-9,doi:10.1002/pmic.200600085
151. Tsangaris, G. T.; Kolialexi, A.; Karamessinis, P. M.; Anagnostopoulos, A. K.; Antsaklis, A.; Fountoulakis, M.; Mavrou, A. The normal human amniotic fluid supernatant proteome. *In Vivo* **2006,** *20*, 479-90
152. Vajrychova, M.; Kacerovsky, M.; Tambor, V.; Hornychova, H.; Lenco, J. Microbial invasion and histological chorioamnionitis upregulate neutrophil-gelatinase associated lipocalin in preterm prelabor rupture of membranes. *J. Matern. Fetal Neonatal Med.* **2016,** *29*, 12-21,doi:10.3109/14767058.2014.991305
153. Vascotto, C.; Salzano, A. M.; D'Ambrosio, C.; Fruscalzo, A.; Marchesoni, D.; di Loreto, C.; Scaloni, A.; Tell, G.; Quadrifoglio, F. Oxidized transthyretin in amniotic fluid as an early marker of preeclampsia. *J. Proteome Res.* **2007,** *6*, 160-70,doi:10.1021/pr060315z
154. Vento, G.; Tirone, C.; Aurilia, C.; Tana, M.; Piras, A.; Lio, A.; Tesfagabir, G.; Castagnola, M.; Fanali, C.; Romagnoli, C. Proteomics and neonatal infection. *Minerva Pediatr.* **2010,** *62*, 47-9
155. Virgiliou, C.; Gika, H. G.; Witting, M.; Bletsou, A. A.; Athanasiadis, A.; Zafrakas, M.; Thomaidis, N. S.; Raikos, N.; Makrydimas, G.; Theodoridis, G. A. Amniotic Fluid and Maternal Serum Metabolic Signatures in the Second Trimester Associated with Preterm Delivery. *J. Proteome Res.* **2017,** *16*, 898-910,doi:10.1021/acs.jproteome.6b00845
156. Vuadens, F.; Benay, C.; Crettaz, D.; Gallot, D.; Sapin, V.; Schneider, P.; Bienvenut, W. V.; Lemery, D.; Quadroni, M.; Dastugue, B., et al. Identification of biologic markers of the premature rupture of fetal membranes: proteomic approach. *Proteomics* **2003,** *3*, 1521-5,doi:10.1002/pmic.200300455
157. Wang, T.; Zhou, R.; Zhang, L.; Wang, Y.; Song, C.; Lin, W.; Niu, X.; Lin, Y.; Hu, H. Proteins in leaked amniotic fluid as biomarkers diagnostic for prelabor rupture of membranes. *Proteomics Clin. Appl.* **2011,** *5*, 415-21,doi:10.1002/prca.201000123

158. Wang, T. H.; Chang, Y. L.; Peng, H. H.; Wang, S. T.; Lu, H. W.; Teng, S. H.; Chang, S. D.; Wang, H. S. Rapid detection of fetal aneuploidy using proteomics approaches on amniotic fluid supernatant. *Prenat. Diagn.* **2005,** *25*, 559-66,doi:10.1002/pd.1186
159. Wang, T. H.; Chao, A. S.; Chen, J. K.; Chao, A.; Chang, Y. L.; Cheng, P. J.; Chang, S. D.; Wang, H. S. Network analyses of differentially expressed proteins in amniotic fluid supernatant associated with abnormal human karyotypes. *Fertil. Steril.* **2009,** *92*, 96-107,doi:10.1016/j.fertnstert.2008.05.038
160. Wang, Y.; Luo, H.; Che, G.; Li, Y.; Gao, J.; Yang, Q.; Zhou, B.; Gao, L.; Wang, T.; Liang, Y., et al. Placental protein 14 as a potential biomarker for diagnosis of preterm premature rupture of membranes. *Mol. Med. Rep.* **2018,** *18*, 113-122,doi:10.3892/mmr.2018.8967
161. Xanthopoulou, A. G.; Anagnostopoulos, A. K.; Thanasopoulou, A.; Anastasiadou, E.; Sifakis, S.; Siafaka-Kapadai, A.; Tsangaris, G. T. The proteome of normal human chorionic villus sampling cells. *In Vivo* **2011,** *25*, 945-61
162. Yu, B.; Zhang, B.; Wang, J.; Wang, Q. W.; Huang, R. P.; Yang, Y. Q.; Shao, S. H. Preliminary proteomic-based identification of a novel protein for Down's syndrome in maternal serum. *Exp. Biol. Med. (Maywood)* **2012,** *237*, 530-9,doi:10.1258/ebm.2012.011312
163. Zagoura, D. S.; Roubelakis, M. G.; Bitsika, V.; Trohatou, O.; Pappa, K. I.; Kapelouzou, A.; Antsaklis, A.; Anagnou, N. P. Therapeutic potential of a distinct population of human amniotic fluid mesenchymal stem cells and their secreted molecules in mice with acute hepatic failure. *Gut* **2012,** *61*, 894-906,doi:10.1136/gutjnl-2011-300908
164. Zhao, M.; Yang, Y.; Guo, Z.; Shao, C.; Sun, H.; Zhang, Y.; Sun, Y.; Liu, Y.; Song, Y.; Zhang, L., et al. A Comparative Proteomics Analysis of Five Body Fluids: Plasma, Urine, Cerebrospinal Fluid, Amniotic Fluid, and Saliva. *Proteomics Clin. Appl.* **2018,** *12*, e1800008,doi:10.1002/prca.201800008
165. Caspi, R.; Billington, R.; Ferrer, L.; Foerster, H.; Fulcher, C. A.; Keseler, I. M.; Kothari, A.; Krummenacker, M.; Latendresse, M.; Mueller, L. A., et al. The MetaCyc database of metabolic pathways and enzymes and the BioCyc collection of pathway/genome databases. *Nucl. Acids Res.* **2016,** *44*, D471-80,doi:10.1093/nar/gkv1164
166. Miikkulainen, P.; Hogel, H.; Rantanen, K.; Suomi, T.; Kouvonen, P.; Elo, L. L.; Jaakkola, P. M. HIF prolyl hydroxylase PHD3 regulates translational machinery and glucose metabolism in clear cell renal cell carcinoma. *Cancer Metab.* **2017,** *5*, 5,doi:10.1186/s40170-017-0167-y
167. Han, J.; Back, S. H.; Hur, J.; Lin, Y. H.; Gildersleeve, R.; Shan, J.; Yuan, C. L.; Krokowski, D.; Wang, S.; Hatzoglou, M., et al. ER-stress-induced transcriptional regulation increases protein synthesis leading to cell death. *Nat. Cell Biol.* **2013,** *15*, 481-90,doi:10.1038/ncb2738
168. Andreev, D. E.; O'Connor, P. B.; Zhdanov, A. V.; Dmitriev, R. I.; Shatsky, I. N.; Papkovsky, D. B.; Baranov, P. V. Oxygen and glucose deprivation induces widespread alterations in mRNA translation within 20 minutes. *Genome Biol.* **2015,** *16*, 90,doi:10.1186/s13059-015-0651-z
169. Quiros, P. M.; Prado, M. A.; Zamboni, N.; D'Amico, D.; Williams, R. W.; Finley, D.; Gygi, S. P.; Auwerx, J. Multi-omics analysis identifies ATF4 as a key regulator of the mitochondrial stress response in mammals. *J. Cell. Biol.* **2017,** *216*, 2027-2045,doi:10.1083/jcb.201702058

# Supplementary Dataset 1

Settings for the analysis: https://biit.cs.ut.ee/clustvis/?s=qgUMwjwZQZIkbkY

Analysis performed with ClustVis v.2.0. Metsalu, Tauno and Vilo, Jaak. Clustvis: a web tool for visualizing clustering of multivariate data using Principal Component Analysis and heatmap. Nucleic Acids Res. 2015;  43, W566–W570,doi: 10.1093/nar/gkv468.

**Principal component analysis of all noncarrier samples based on the proteomics' data.**

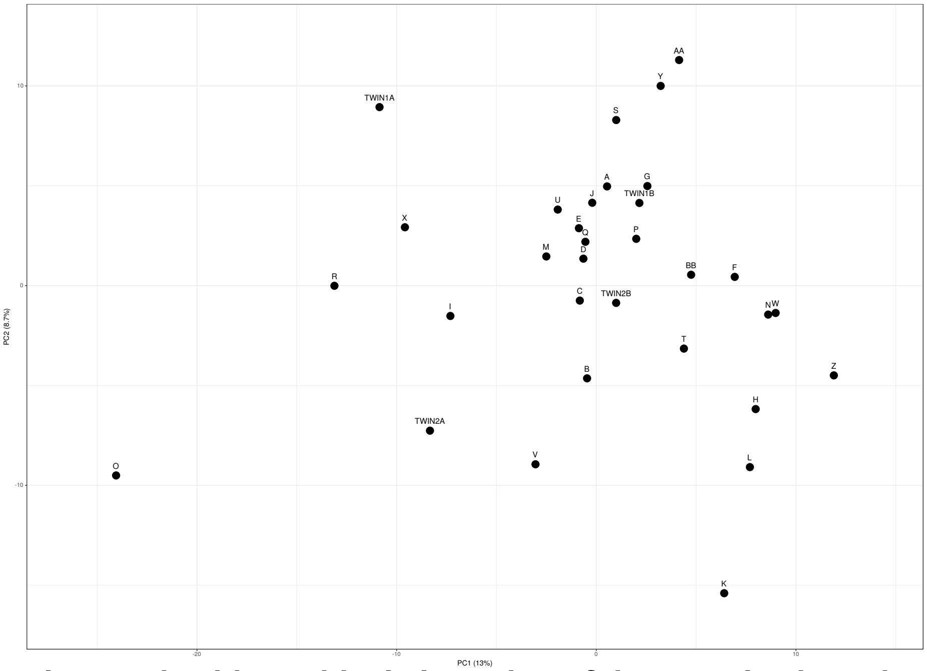


**Agglomerative hierarchical clustering of the samples based on the proteomics' data visualized as a heat map.**

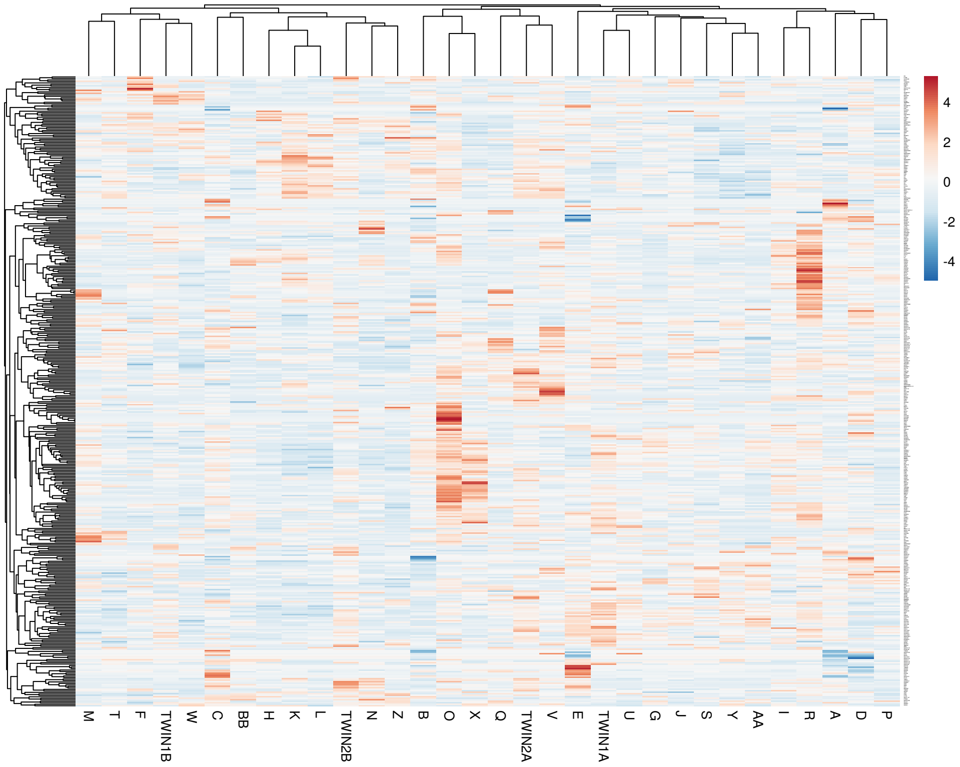

**PCA SCORES**

| | PC1 | PC2 | PC3 | PC4 | PC5 | PC6 | PC7 | PC8 | PC9 | PC10 | PC11 | PC12 | PC13 | PC14 | PC15 | PC16 | PC17 | PC18 | PC19 | PC20 | PC21 | PC22 | PC23 | PC24 | PC25 | PC26 | PC27 |
|---|---|---|---|---|---|---|---|---|---|---|---|---|---|---|---|---|---|---|---|---|---|---|---|---|---|---|---|
| A | -0.54 | 4.97 | -4.28 | 0.15 | -1.00 | 2.74 | -10.17 | 2.93 | -5.18 | 8.23 | 3.42 | -7.52 | -6.42 | 2.27 | 5.28 | -2.82 | -0.75 | -2.51 | -0.60 | 1.26 | 1.69 | 1.70 | -0.75 | -0.18 | -0.48 | -0.58 | -0.23 |
| B | 0.46 | -4.64 | 8.75 | -6.79 | -1.80 | -1.36 | 9.91 | -5.93 | 7.50 | -3.93 | 5.19 | -3.62 | -7.03 | 0.37 | 3.57 | -1.84 | -0.69 | -4.13 | 0.53 | 1.08 | -0.59 | 1.67 | -1.47 | 0.59 | -0.44 | -0.97 | 1.15 |
| C | 0.83 | -0.75 | 6.58 | 10.25 | -4.97 | -9.01 | 1.68 | -8.70 | -2.11 | 8.49 | -7.10 | 2.31 | -4.85 | -1.30 | 0.05 | -0.69 | 1.34 | 3.56 | 0.68 | 0.05 | -1.44 | -1.04 | -1.36 | 1.14 | 0.23 | -0.12 | -0.39 |
| D | 0.65 | 1.35 | -2.56 | -9.22 | -5.99 | 9.50 | -8.17 | -0.97 | 7.80 | 3.05 | -0.76 | 9.55 | -3.30 | -4.21 | -1.02 | -1.29 | -1.17 | 0.83 | -1.78 | -2.03 | -1.68 | -1.52 | -1.18 | 0.69 | -0.69 | -0.01 | -0.56 |
| E | 0.87 | 2.87 | -6.09 | 15.42 | 3.56 | -1.21 | 2.44 | 8.22 | 4.53 | -3.93 | -0.58 | 8.17 | -3.25 | 3.56 | 3.13 | -4.11 | -2.43 | -1.64 | 1.53 | 0.92 | 0.46 | 1.00 | -0.09 | 1.05 | -0.22 | -0.74 | 0.80 |
| F | -6.93 | 0.44 | 2.25 | -0.76 | 2.90 | 6.04 | 3.46 | 3.36 | -8.14 | -7.64 | -3.07 | -0.34 | -6.98 | -5.76 | 0.79 | 0.58 | 0.62 | 5.61 | -0.93 | 0.94 | -2.15 | 0.65 | 1.71 | -0.45 | -2.52 | 0.75 | 0.02 |
| G | -2.56 | 4.98 | 4.61 | 1.17 | -2.01 | -1.49 | 1.00 | 0.35 | 4.22 | 0.03 | 1.58 | 0.29 | 0.94 | 2.32 | 0.63 | 1.69 | 1.89 | 1.25 | -5.61 | -0.74 | 0.72 | -3.23 | 0.48 | -4.00 | 0.98 | -4.63 | 1.74 |
| H | -7.98 | -6.18 | 0.24 | -0.54 | -3.40 | -2.93 | -2.04 | 4.54 | -0.14 | -2.80 | -3.15 | -1.69 | -0.76 | -2.25 | 1.34 | 1.68 | 0.94 | -3.93 | -1.36 | -2.91 | -0.13 | 1.70 | -1.01 | 1.38 | 5.48 | 2.79 | -0.51 |
| I | 7.31 | -1.52 | -3.30 | -3.44 | 0.41 | -1.37 | 1.36 | 0.23 | -3.54 | -1.96 | 0.02 | -2.94 | 1.64 | 1.32 | -0.64 | -8.63 | 0.33 | -1.09 | 3.03 | -4.23 | -5.19 | -5.84 | 1.74 | -0.05 | -1.48 | -2.96 | -3.42 |
| J | 0.20 | 4.15 | -0.21 | -0.52 | 3.53 | 1.90 | 0.53 | 1.53 | 0.15 | -2.71 | -3.29 | -3.28 | -3.06 | 2.07 | -2.17 | 4.27 | 0.65 | 1.66 | -0.10 | -6.14 | 6.86 | -1.85 | -3.06 | 2.30 | 0.88 | -4.29 | -2.08 |
| K | -6.41 | -15.41 | -5.04 | -0.55 | 1.46 | -2.43 | -2.14 | 2.60 | 1.62 | 0.79 | -0.86 | -3.52 | 1.76 | -1.78 | 2.61 | -1.55 | -1.73 | 3.45 | -4.77 | 2.42 | -1.74 | -0.24 | 0.91 | -0.66 | 0.95 | -0.78 | 2.15 |
| L | -7.69 | -9.09 | -3.53 | -2.33 | 2.18 | -3.51 | -0.17 | 1.26 | 3.47 | 2.27 | -1.67 | -0.95 | 3.50 | 1.65 | 1.02 | 0.81 | 1.81 | 3.78 | 0.30 | -3.16 | 0.55 | 1.84 | 1.30 | 0.10 | -3.81 | -2.32 | 2.18 |
| M | 2.50 | 1.46 | 5.45 | -9.33 | 4.47 | -1.30 | 4.13 | 6.55 | -3.23 | 5.14 | 2.22 | 3.01 | -0.84 | 7.91 | -3.06 | 2.84 | -4.15 | 3.82 | 0.89 | 1.47 | -4.36 | 0.77 | -2.55 | 0.61 | 2.31 | 0.51 | 1.00 |
| N | -8.61 | -1.45 | -2.43 | 2.63 | -9.56 | -1.22 | -3.80 | -0.18 | -3.08 | -3.45 | 3.76 | 0.91 | 2.51 | -2.12 | -3.39 | 0.45 | -0.61 | -0.38 | 6.17 | 2.77 | -0.37 | 1.36 | -3.55 | -4.18 | 1.45 | -5.14 | 1.62 |
| O | 24.05 | -9.51 | 9.68 | 0.29 | -6.47 | 5.52 | -2.02 | 2.62 | -2.17 | -2.12 | -5.63 | 0.20 | 4.40 | 1.24 | 5.70 | 2.23 | -1.01 | -0.52 | 1.63 | 1.19 | 1.63 | -0.01 | -0.41 | -0.05 | -0.28 | -0.07 | 0.54 |
| P | -2.00 | 2.34 | -2.89 | -5.03 | 2.65 | -2.41 | -4.12 | -2.41 | 6.38 | -1.19 | -2.71 | -0.73 | 1.11 | 3.18 | 2.04 | -0.98 | 3.78 | 3.30 | 6.04 | 0.10 | 0.33 | 4.55 | 4.22 | 0.87 | 1.99 | 1.56 | -2.35 |
| Q | 0.55 | 2.19 | 2.29 | -0.80 | 7.33 | -4.70 | -4.40 | -4.22 | -4.96 | -1.03 | 8.89 | 5.89 | 2.74 | -3.34 | 6.79 | 4.73 | 1.49 | -0.30 | 1.32 | -0.77 | -0.24 | -0.89 | 0.29 | 3.93 | -1.86 | -1.31 | 0.56 |
| R | 13.12 | -0.01 | -21.01 | -6.10 | -6.61 | -5.24 | 6.43 | -2.39 | -3.17 | -1.21 | 1.15 | 1.92 | -2.46 | 0.58 | -0.57 | 2.72 | 1.65 | 0.89 | -1.20 | 2.43 | 2.85 | -0.90 | 0.40 | -0.58 | 0.49 | 1.86 | 0.14 |
| TWIN1A | 10.86 | 8.94 | -0.35 | 1.29 | 4.66 | -2.54 | 4.46 | 3.26 | 3.26 | 3.94 | 0.55 | -2.44 | 3.02 | -9.20 | -0.04 | 0.99 | -3.31 | -0.11 | -1.71 | 0.98 | -1.28 | 2.72 | 2.10 | -1.16 | 3.70 | -2.73 | -3.53 |
| TWIN1B | -2.16 | 4.14 | -3.17 | -1.33 | 0.70 | 6.41 | 7.27 | -2.48 | -3.48 | 3.30 | -2.64 | 0.60 | 6.13 | -0.94 | -1.37 | -5.68 | 1.43 | -2.29 | -2.76 | -0.15 | 1.69 | 4.15 | -2.22 | 4.73 | -1.40 | -1.27 | 3.89 |
| S | -1.00 | 8.29 | 0.33 | -2.02 | 2.62 | -1.27 | -4.46 | -0.47 | 2.19 | -4.17 | -5.36 | -3.21 | 1.46 | -0.01 | -2.68 | 1.20 | 3.52 | -1.32 | 0.76 | 6.04 | -3.51 | 0.33 | -4.95 | 2.68 | -1.09 | -0.17 | -0.96 |
| T | -4.39 | -3.16 | 4.01 | -3.11 | 3.40 | -3.00 | 0.51 | 5.39 | -2.16 | 2.69 | -1.60 | 3.74 | -0.40 | -0.51 | -1.92 | 0.26 | 7.66 | -6.95 | -1.06 | 1.66 | 0.42 | -4.31 | 2.26 | -0.66 | 1.60 | 1.16 | 0.67 |
| U | 1.93 | 3.81 | 0.22 | -1.23 | 4.31 | -1.46 | 0.80 | 2.84 | 3.88 | 3.57 | -2.62 | -3.29 | -0.63 | -4.01 | -2.34 | 2.21 | -3.08 | -2.05 | 5.74 | 0.07 | 2.42 | -2.72 | 1.01 | -3.07 | -5.34 | 2.89 | 4.52 |
| TWIN2A | 8.33 | -7.27 | -1.78 | 7.93 | 6.83 | 3.90 | -0.31 | -1.06 | 2.87 | 0.58 | 6.69 | -2.21 | 1.20 | -1.32 | -2.14 | -1.29 | 4.37 | 2.92 | -0.40 | -2.10 | -0.57 | -0.67 | -6.59 | -2.34 | 0.51 | 4.90 | -1.00 |
| TWIN2B | -0.99 | -0.87 | -0.66 | 8.96 | -6.11 | 10.88 | 3.93 | 0.19 | 1.27 | 3.21 | 4.26 | -2.75 | -0.33 | 2.27 | -3.16 | 5.80 | 3.92 | -0.68 | 1.24 | 0.36 | -3.34 | 0.83 | 5.47 | 1.96 | -0.72 | -0.62 | -0.69 |
| V | 3.04 | -8.95 | -2.81 | 2.07 | 11.48 | 3.52 | -4.90 | -10.12 | -2.31 | -1.80 | -2.88 | 1.73 | -1.82 | 2.63 | -4.97 | 1.55 | -4.66 | -4.33 | -1.68 | 1.37 | 0.09 | 1.34 | 2.41 | -2.05 | 1.14 | -1.63 | -0.28 |
| W | -8.98 | -1.37 | 6.06 | -3.27 | -0.04 | 2.62 | 4.81 | -0.45 | -4.16 | 2.67 | 0.72 | 4.55 | 1.78 | 0.09 | 0.55 | -2.96 | 0.65 | -0.03 | 1.77 | -0.35 | 4.60 | 3.26 | 0.00 | -5.40 | -0.38 | 1.62 | -4.53 |
| X | 9.59 | 2.92 | 9.45 | 0.63 | -4.10 | -6.53 | -6.75 | 1.72 | -0.71 | -4.16 | 5.09 | -0.34 | -0.66 | 0.14 | -7.75 | -4.58 | 0.32 | 2.28 | -3.42 | 0.18 | 3.24 | 2.51 | 3.67 | 1.78 | -1.18 | 1.58 | 1.55 |
| Y | -3.22 | 10.00 | 0.10 | 1.04 | -0.76 | 3.19 | -0.10 | -4.96 | -2.05 | -2.32 | -0.28 | -1.39 | 1.83 | 0.41 | 1.92 | -1.20 | -2.13 | 1.20 | 1.87 | -4.12 | -1.15 | -2.26 | 1.62 | -0.84 | 6.19 | 3.41 | 5.56 |
| Z | -11.90 | -4.50 | 1.15 | 1.54 | -2.62 | 1.37 | 1.56 | -0.63 | 1.50 | 0.58 | 2.50 | -1.50 | 3.01 | -0.41 | -1.14 | -0.65 | -5.91 | 1.40 | 1.19 | 4.42 | 4.13 | -5.92 | 0.52 | 5.41 | 0.81 | 2.05 | -3.30 |
| AA | -4.15 | 11.29 | 0.54 | 1.29 | -1.01 | 1.06 | -0.52 | -3.26 | 1.24 | -2.88 | -1.64 | -0.89 | 3.76 | 4.35 | 4.19 | 0.48 | -0.14 | 0.57 | -5.05 | 3.00 | -0.29 | -1.78 | 0.26 | -3.83 | -2.75 | 2.34 | -1.98 |
| BB | -4.75 | 0.54 | -1.61 | 1.72 | -6.06 | -5.67 | -0.21 | 0.67 | -1.30 | -1.22 | -0.20 | -0.25 | 1.99 | 0.80 | -1.22 | 3.80 | -4.62 | -4.29 | -2.24 | -6.00 | -3.66 | 2.80 | -1.17 | 0.26 | -4.07 | 2.93 | -2.28 |

| PC28 | PC29 | PC30 | PC31 | PC32 |
|---|---|---|---|---|
| 1.80 | 0.24 | 0.14 | -0.80 | 0.00 |
| -0.16 | 1.56 | -0.63 | -0.05 | 0.00 |
| -0.81 | -0.22 | -0.69 | 0.19 | 0.00 |
| -0.73 | 0.61 | -0.31 | -0.10 | 0.00 |
| -0.27 | 0.56 | 0.88 | -0.16 | 0.00 |
| 2.79 | -0.96 | -1.34 | -0.28 | 0.00 |
| 4.49 | -5.85 | 2.94 | 0.03 | 0.00 |
| -4.06 | -5.28 | -0.91 | -3.45 | 0.00 |
| -1.67 | -1.53 | 0.38 | -0.46 | 0.00 |
| -2.34 | 2.34 | -0.74 | 2.47 | 0.00 |
| -3.35 | 1.65 | 2.36 | 5.21 | 0.00 |
| 1.35 | 3.56 | -0.56 | -6.33 | 0.00 |
| -0.96 | -0.75 | -1.29 | -0.08 | 0.00 |
| -0.03 | 0.62 | -3.31 | 0.70 | 0.00 |
| 1.54 | 0.37 | 0.06 | -0.01 | 0.00 |
| 2.77 | -2.50 | -1.98 | 3.21 | 0.00 |
| -1.93 | -1.25 | 0.83 | 0.45 | 0.00 |
| 0.24 | -0.22 | 1.15 | -0.73 | 0.00 |
| 0.65 | 1.96 | -1.16 | -1.05 | 0.00 |
| 0.40 | -2.47 | -1.82 | 1.33 | 0.00 |
| -0.13 | 1.35 | 5.64 | -1.27 | 0.00 |
| 2.52 | 3.73 | -2.54 | 1.42 | 0.00 |
| -2.19 | -3.01 | 0.32 | 0.79 | 0.00 |
| 1.66 | -0.51 | -1.35 | -0.02 | 0.00 |
| -2.75 | 0.09 | 1.42 | 0.11 | 0.00 |
| 0.61 | -0.85 | -0.36 | -1.07 | 0.00 |
| -1.91 | 0.70 | 5.42 | -0.17 | 0.00 |
| -1.47 | 0.62 | 0.18 | -0.41 | 0.00 |
| 0.91 | 3.86 | 2.38 | -0.51 | 0.00 |
| 3.07 | -0.57 | -0.45 | -1.04 | 0.00 |
| -4.12 | 0.98 | -5.29 | -0.58 | 0.00 |
| 4.03 | 1.15 | 0.64 | 2.66 | 0.00 |

**PCA LOADINGS**

| | PC1 | PC2 | PC3 | PC4 | PC5 | PC6 | PC7 | PC8 | PC9 | PC10 | PC11 | PC12 |
|---|---|---|---|---|---|---|---|---|---|---|---|---|
| A1BG | 0.04 | -0.08 | 0.02 | 0.00 | 0.06 | 0.06 | 0.03 | 0.02 | 0.04 | 0.00 | 0.08 | 0.05 |
| AFP | 0.01 | 0.05 | -0.08 | 0.05 | 0.02 | 0.10 | -0.06 | -0.07 | 0.08 | -0.03 | 0.01 | 0.00 |
| ALB | 0.00 | 0.01 | 0.01 | -0.02 | -0.07 | 0.02 | -0.08 | -0.08 | 0.00 | 0.15 | -0.05 | 0.04 |
| APOA1 | 0.01 | -0.04 | 0.04 | 0.04 | 0.03 | -0.02 | 0.02 | 0.07 | 0.00 | 0.05 | -0.02 | 0.04 |
| APOC1 | -0.03 | -0.11 | -0.03 | 0.07 | -0.03 | -0.03 | 0.00 | 0.03 | 0.03 | 0.02 | -0.02 | -0.03 |
| APOH | 0.00 | -0.02 | 0.03 | 0.00 | 0.09 | 0.02 | -0.07 | 0.11 | -0.03 | 0.02 | -0.10 | 0.02 |
| B2M | 0.05 | 0.09 | 0.05 | -0.06 | 0.05 | 0.00 | -0.03 | 0.04 | -0.02 | -0.04 | -0.01 | -0.04 |
| CD59 | 0.00 | 0.09 | -0.02 | -0.06 | -0.02 | 0.06 | 0.08 | 0.01 | 0.08 | 0.00 | -0.05 | 0.01 |
| CDH1 | 0.09 | -0.01 | -0.05 | -0.03 | 0.01 | 0.06 | 0.03 | -0.05 | -0.05 | -0.01 | 0.06 | 0.06 |
| CLU | 0.02 | -0.11 | -0.01 | -0.04 | 0.05 | -0.03 | -0.01 | -0.01 | 0.05 | 0.03 | -0.04 | -0.05 |
| COL5A1 | 0.02 | 0.06 | -0.07 | 0.07 | -0.02 | 0.06 | 0.02 | 0.03 | 0.03 | -0.01 | -0.02 | -0.03 |
| COL6A3 | 0.04 | 0.09 | -0.08 | 0.05 | 0.05 | 0.05 | 0.03 | -0.01 | 0.02 | 0.03 | -0.01 | -0.04 |
| CPM | 0.04 | 0.02 | -0.07 | -0.09 | 0.04 | 0.06 | 0.00 | -0.05 | -0.01 | 0.07 | -0.07 | 0.02 |
| FAM3C | -0.04 | 0.07 | -0.02 | -0.02 | 0.02 | 0.04 | -0.03 | -0.06 | 0.03 | 0.04 | 0.05 | -0.03 |
| FBLN1 | 0.11 | 0.04 | 0.04 | -0.02 | -0.02 | -0.03 | 0.05 | 0.01 | 0.02 | 0.03 | -0.02 | -0.04 |
| GC | -0.04 | -0.05 | 0.03 | -0.02 | 0.03 | 0.06 | 0.08 | 0.02 | 0.02 | 0.09 | -0.03 | 0.01 |
| GP2 | 0.05 | 0.00 | 0.03 | 0.01 | 0.03 | 0.05 | -0.06 | -0.03 | -0.10 | -0.07 | -0.12 | -0.06 |
| GSN | 0.03 | -0.05 | 0.01 | -0.03 | 0.04 | 0.00 | 0.12 | 0.07 | 0.04 | -0.03 | -0.08 | 0.02 |
| HBD | 0.01 | -0.01 | -0.02 | 0.15 | -0.04 | -0.01 | -0.02 | 0.02 | 0.00 | 0.08 | 0.01 | 0.04 |
| HP | -0.01 | -0.05 | -0.03 | -0.05 | 0.01 | -0.02 | 0.07 | -0.01 | -0.01 | 0.05 | -0.08 | -0.02 |
| HSPB1 | 0.02 | -0.01 | 0.06 | 0.04 | -0.01 | 0.02 | 0.00 | -0.09 | -0.10 | 0.01 | 0.07 | 0.04 |
| IGFBP1 | 0.08 | 0.06 | 0.08 | -0.04 | -0.03 | -0.05 | -0.01 | 0.04 | -0.04 | -0.02 | -0.01 | -0.03 |
| IGHG1 | 0.03 | -0.03 | 0.00 | -0.05 | -0.02 | -0.02 | -0.12 | 0.04 | -0.06 | 0.01 | 0.05 | 0.07 |
| ITIH4 | -0.04 | -0.08 | -0.01 | 0.03 | -0.03 | 0.04 | -0.01 | 0.06 | 0.03 | 0.10 | 0.08 | -0.05 |
| LMAN2 | 0.05 | 0.09 | -0.02 | -0.01 | 0.00 | 0.09 | -0.05 | 0.04 | -0.01 | 0.01 | 0.02 | 0.05 |
| LTBP1 | 0.02 | 0.05 | -0.04 | 0.03 | -0.02 | -0.07 | 0.06 | -0.01 | -0.05 | 0.11 | -0.02 | -0.04 |
| MSLN | 0.01 | 0.05 | -0.01 | 0.00 | -0.01 | 0.06 | 0.07 | 0.02 | -0.05 | 0.07 | 0.06 | -0.11 |
| PAEP | 0.06 | 0.03 | 0.02 | -0.07 | -0.07 | 0.04 | -0.08 | -0.01 | 0.11 | -0.03 | -0.03 | 0.07 |
| RNASE1 | 0.08 | 0.05 | -0.03 | 0.01 | 0.05 | -0.04 | -0.07 | 0.01 | 0.04 | 0.08 | 0.06 | -0.04 |

| | | | | | | | | | | | | |
|---|---|---|---|---|---|---|---|---|---|---|---|---|
| SERPINA1 | 0.00 | -0.09 | 0.04 | 0.03 | 0.03 | 0.08 | -0.01 | 0.04 | 0.06 | 0.06 | 0.03 | 0.04 |
| SPARC | 0.07 | -0.01 | -0.04 | 0.12 | 0.02 | 0.06 | 0.01 | 0.00 | 0.01 | 0.01 | 0.00 | -0.04 |
| SPINT1 | 0.03 | 0.04 | -0.06 | -0.03 | 0.04 | 0.01 | -0.06 | -0.05 | 0.08 | 0.01 | -0.02 | 0.01 |
| TF | 0.00 | 0.01 | 0.07 | -0.07 | 0.06 | -0.02 | 0.06 | -0.03 | -0.02 | 0.04 | 0.01 | 0.01 |
| TNXB | 0.04 | -0.02 | -0.04 | -0.04 | 0.10 | 0.00 | 0.02 | 0.05 | 0.00 | -0.03 | -0.05 | 0.01 |
| VIM | 0.06 | -0.03 | 0.07 | 0.06 | -0.10 | -0.02 | -0.06 | 0.00 | -0.05 | 0.03 | -0.01 | -0.03 |
| A2M | -0.01 | -0.04 | -0.05 | 0.05 | -0.03 | -0.04 | -0.05 | 0.04 | 0.04 | 0.00 | 0.05 | -0.01 |
| A2ML1 | 0.04 | -0.07 | -0.03 | -0.01 | 0.05 | 0.11 | -0.06 | -0.01 | -0.01 | 0.01 | 0.00 | -0.03 |
| ABI3BP | 0.11 | -0.01 | 0.07 | -0.01 | -0.02 | -0.01 | -0.03 | 0.06 | -0.02 | -0.03 | -0.01 | -0.02 |
| ACE | 0.03 | -0.01 | -0.02 | -0.04 | 0.01 | -0.04 | -0.03 | 0.01 | -0.04 | -0.01 | -0.03 | 0.03 |
| ACE2 | 0.01 | 0.04 | -0.11 | -0.04 | -0.09 | 0.04 | -0.03 | 0.00 | 0.01 | 0.05 | 0.01 | 0.03 |
| ACTB | 0.10 | -0.04 | -0.03 | -0.03 | -0.02 | -0.05 | -0.02 | 0.08 | -0.04 | -0.02 | -0.01 | -0.07 |
| ACTG2 | 0.00 | 0.00 | 0.00 | -0.12 | -0.04 | 0.07 | -0.09 | -0.04 | -0.03 | 0.08 | 0.03 | -0.08 |
| ACTN4 | 0.08 | -0.06 | -0.05 | -0.06 | -0.01 | 0.03 | -0.01 | -0.03 | -0.04 | -0.02 | -0.06 | 0.02 |
| ADAMTS1 | 0.05 | 0.00 | 0.06 | -0.03 | -0.08 | -0.04 | 0.05 | -0.02 | 0.00 | -0.03 | 0.02 | -0.04 |
| ADAMTS2 | 0.06 | 0.01 | -0.02 | 0.03 | 0.03 | -0.07 | 0.03 | 0.02 | 0.05 | 0.01 | -0.06 | -0.01 |
| AFM | -0.05 | -0.01 | 0.01 | -0.03 | -0.01 | 0.03 | 0.04 | 0.05 | -0.01 | 0.08 | 0.08 | 0.14 |
| AGRN | -0.02 | 0.10 | -0.04 | 0.02 | 0.01 | 0.11 | 0.01 | 0.04 | -0.04 | -0.05 | -0.04 | 0.01 |
| AGT | 0.02 | -0.08 | 0.03 | -0.03 | 0.04 | 0.03 | 0.01 | -0.05 | 0.09 | 0.03 | 0.08 | 0.02 |
| AHSG | 0.04 | -0.08 | -0.02 | 0.00 | 0.06 | -0.01 | 0.02 | 0.01 | 0.04 | 0.03 | -0.01 | -0.01 |
| AHSP | 0.02 | 0.02 | -0.03 | 0.03 | 0.05 | 0.05 | -0.07 | 0.03 | 0.05 | -0.02 | 0.02 | -0.02 |
| AMBP | 0.03 | 0.07 | -0.03 | -0.02 | 0.04 | 0.06 | 0.03 | 0.02 | 0.04 | -0.10 | -0.03 | -0.09 |
| ANGPTL2 | 0.04 | -0.03 | -0.09 | -0.02 | 0.03 | -0.02 | -0.01 | 0.01 | 0.00 | 0.09 | -0.01 | -0.07 |
| ANXA1 | 0.07 | -0.03 | -0.03 | -0.06 | 0.00 | 0.05 | 0.05 | -0.08 | -0.08 | 0.00 | -0.04 | 0.05 |
| ANXA13 | 0.00 | 0.03 | -0.06 | -0.01 | -0.02 | -0.01 | -0.05 | 0.06 | -0.02 | -0.07 | -0.07 | -0.10 |
| ANXA2 | 0.08 | -0.06 | -0.06 | -0.05 | -0.04 | 0.00 | 0.07 | 0.01 | -0.03 | -0.04 | -0.09 | 0.02 |
| ANXA4 | 0.04 | -0.01 | -0.11 | -0.05 | -0.05 | -0.06 | 0.06 | -0.03 | 0.01 | -0.03 | 0.04 | -0.01 |
| ANXA5 | 0.02 | -0.01 | -0.12 | -0.05 | -0.03 | -0.02 | 0.01 | -0.02 | -0.03 | 0.05 | 0.00 | -0.04 |
| AOC1 | 0.02 | 0.00 | 0.05 | -0.03 | 0.06 | 0.08 | 0.08 | -0.01 | -0.10 | 0.09 | 0.03 | 0.06 |
| APOA2 | -0.07 | -0.03 | 0.02 | -0.01 | -0.01 | 0.05 | 0.06 | 0.01 | 0.03 | 0.10 | -0.02 | 0.03 |
| APOA4 | -0.05 | -0.09 | -0.02 | 0.08 | -0.01 | 0.00 | -0.05 | 0.01 | -0.02 | 0.04 | -0.07 | -0.02 |

| | | | | | | | | | | | | |
|---|---|---|---|---|---|---|---|---|---|---|---|---|
| APOB | -0.02 | 0.01 | -0.09 | -0.02 | -0.03 | -0.04 | -0.03 | 0.00 | 0.01 | -0.09 | -0.08 | -0.04 |
| APOC3 | -0.05 | -0.08 | -0.01 | 0.02 | -0.01 | -0.07 | 0.01 | 0.03 | 0.08 | 0.04 | -0.03 | -0.03 |
| APOD | 0.08 | 0.01 | 0.07 | -0.02 | -0.03 | -0.07 | -0.02 | 0.01 | -0.02 | -0.06 | 0.04 | 0.00 |
| APOE | 0.00 | -0.01 | -0.03 | 0.02 | -0.07 | 0.00 | -0.11 | 0.01 | 0.01 | -0.01 | 0.06 | 0.00 |
| ATRN | 0.00 | -0.03 | -0.05 | 0.01 | 0.05 | -0.01 | 0.06 | 0.03 | 0.04 | 0.00 | -0.01 | -0.03 |
| AXL | 0.07 | 0.02 | 0.07 | 0.02 | 0.08 | -0.03 | 0.06 | -0.06 | -0.03 | 0.05 | 0.03 | -0.02 |
| AZGP1 | 0.00 | -0.13 | -0.01 | -0.02 | 0.04 | 0.00 | 0.02 | 0.00 | 0.01 | -0.01 | 0.01 | -0.02 |
| B4GALT1 | 0.00 | 0.03 | -0.01 | 0.00 | 0.05 | 0.01 | -0.11 | -0.06 | -0.06 | -0.07 | -0.06 | -0.06 |
| BASP1 | 0.07 | 0.01 | 0.02 | 0.00 | -0.10 | 0.04 | 0.00 | -0.01 | 0.09 | 0.01 | 0.06 | -0.03 |
| BMP1 | 0.03 | -0.05 | 0.05 | 0.02 | -0.06 | 0.04 | -0.01 | 0.01 | 0.01 | 0.00 | -0.03 | 0.00 |
| BPIFB1 | 0.08 | -0.04 | 0.05 | -0.02 | -0.06 | 0.07 | -0.04 | 0.03 | 0.00 | -0.02 | -0.08 | 0.04 |
| BTD | 0.03 | -0.03 | 0.01 | 0.06 | 0.04 | 0.10 | -0.02 | -0.06 | -0.04 | -0.04 | 0.07 | 0.09 |
| C1R | 0.09 | -0.01 | -0.03 | 0.08 | -0.02 | 0.00 | 0.02 | 0.06 | 0.03 | -0.03 | 0.05 | 0.01 |
| C1RL | -0.02 | -0.01 | 0.04 | 0.01 | -0.08 | -0.01 | 0.08 | -0.07 | 0.02 | 0.04 | -0.01 | -0.05 |
| C1S | 0.04 | 0.08 | -0.04 | 0.04 | 0.00 | 0.04 | 0.04 | 0.01 | 0.05 | -0.03 | 0.01 | -0.06 |
| C2 | 0.02 | -0.10 | 0.01 | 0.05 | 0.07 | 0.06 | -0.03 | -0.02 | 0.04 | 0.02 | 0.02 | -0.03 |
| C3 | 0.02 | -0.13 | 0.03 | -0.02 | 0.00 | 0.00 | -0.01 | -0.02 | 0.03 | -0.01 | 0.04 | 0.05 |
| C4A | -0.02 | -0.02 | 0.04 | 0.06 | -0.04 | -0.08 | 0.10 | -0.09 | 0.06 | 0.00 | -0.09 | 0.06 |
| C4B | -0.01 | -0.11 | 0.03 | 0.01 | 0.02 | -0.01 | 0.03 | 0.00 | 0.05 | 0.02 | -0.11 | 0.02 |
| C5 | -0.06 | -0.10 | -0.03 | 0.04 | -0.01 | 0.03 | 0.01 | 0.05 | -0.02 | 0.00 | -0.04 | -0.03 |
| C6 | 0.05 | -0.12 | -0.02 | -0.02 | 0.03 | -0.04 | 0.02 | 0.02 | 0.05 | 0.01 | -0.03 | -0.07 |
| C7 | 0.00 | -0.06 | 0.00 | 0.02 | -0.02 | 0.05 | 0.04 | 0.04 | -0.04 | -0.06 | -0.01 | -0.03 |
| C8A | 0.04 | -0.07 | 0.01 | 0.00 | 0.03 | -0.01 | -0.04 | 0.12 | 0.01 | 0.06 | 0.03 | 0.00 |
| C8B | 0.04 | -0.07 | -0.02 | 0.04 | 0.08 | 0.05 | -0.01 | -0.04 | -0.01 | 0.00 | -0.02 | 0.01 |
| C8G | -0.04 | -0.04 | -0.03 | 0.06 | 0.00 | 0.05 | -0.04 | 0.01 | -0.03 | 0.07 | 0.01 | 0.00 |
| C9 | 0.00 | -0.06 | 0.02 | 0.01 | -0.03 | 0.10 | 0.04 | -0.01 | -0.06 | -0.04 | 0.07 | -0.09 |
| CA1 | 0.01 | -0.01 | -0.05 | 0.14 | -0.06 | 0.02 | 0.00 | 0.01 | -0.01 | 0.02 | 0.03 | 0.03 |
| CALM1 | 0.00 | 0.02 | -0.05 | -0.02 | -0.02 | 0.08 | -0.12 | 0.05 | 0.01 | 0.06 | -0.04 | 0.12 |
| CALR | 0.04 | -0.08 | -0.06 | 0.02 | -0.11 | -0.05 | -0.04 | 0.04 | 0.00 | -0.02 | 0.00 | -0.04 |
| CALU | 0.06 | 0.04 | -0.08 | 0.06 | 0.04 | 0.01 | 0.05 | -0.02 | 0.05 | 0.00 | 0.05 | 0.06 |
| CAMP | 0.08 | -0.01 | 0.08 | 0.06 | -0.09 | 0.01 | -0.04 | 0.02 | 0.00 | -0.02 | 0.01 | -0.02 |

| | | | | | | | | | | | | |
|---|---|---|---|---|---|---|---|---|---|---|---|---|
| CD14 | 0.08 | 0.04 | 0.05 | -0.03 | -0.03 | -0.06 | -0.05 | -0.04 | 0.03 | -0.05 | 0.10 | 0.01 |
| CD163 | 0.06 | 0.00 | 0.02 | 0.01 | -0.09 | 0.00 | -0.03 | 0.02 | 0.06 | 0.11 | 0.00 | -0.04 |
| CD248 | 0.10 | 0.00 | 0.07 | -0.05 | -0.01 | -0.04 | 0.04 | -0.02 | -0.03 | 0.04 | 0.04 | -0.02 |
| CD55 | 0.10 | 0.02 | 0.00 | -0.06 | -0.04 | 0.04 | 0.02 | 0.03 | 0.01 | 0.00 | -0.06 | 0.02 |
| CD9 | 0.00 | 0.02 | -0.03 | 0.00 | 0.01 | 0.02 | -0.09 | 0.05 | -0.08 | 0.08 | 0.06 | -0.13 |
| CDC5L | -0.03 | -0.09 | 0.00 | 0.00 | 0.00 | -0.08 | 0.03 | -0.04 | 0.05 | 0.05 | -0.05 | -0.05 |
| CDH11 | 0.04 | 0.06 | -0.04 | 0.04 | -0.02 | 0.09 | 0.01 | -0.04 | -0.03 | -0.04 | 0.06 | -0.04 |
| CDH13 | 0.05 | -0.01 | -0.04 | -0.06 | 0.09 | 0.03 | 0.07 | -0.03 | -0.07 | -0.03 | 0.02 | -0.01 |
| CDH15 | 0.03 | -0.08 | -0.10 | 0.01 | 0.05 | -0.03 | 0.00 | -0.05 | -0.01 | 0.00 | -0.02 | 0.07 |
| CDH5 | 0.01 | -0.02 | -0.03 | -0.05 | -0.06 | 0.04 | 0.06 | -0.02 | 0.04 | 0.04 | 0.06 | 0.02 |
| CDHR5 | 0.02 | -0.01 | -0.10 | -0.02 | -0.08 | 0.00 | 0.06 | -0.02 | -0.07 | -0.04 | 0.04 | 0.01 |
| CEL | 0.01 | -0.03 | -0.02 | -0.02 | 0.06 | 0.06 | -0.09 | -0.12 | 0.03 | -0.02 | -0.07 | 0.06 |
| CELA3A | 0.05 | -0.05 | 0.01 | -0.01 | 0.04 | 0.07 | -0.09 | -0.08 | 0.01 | -0.04 | -0.01 | 0.06 |
| CFB | 0.01 | -0.01 | -0.02 | 0.00 | 0.05 | 0.02 | -0.01 | -0.03 | 0.15 | -0.04 | -0.02 | 0.09 |
| CFD | 0.04 | 0.05 | -0.02 | 0.02 | -0.07 | -0.01 | 0.10 | -0.08 | 0.02 | 0.01 | 0.06 | 0.04 |
| CFH | 0.01 | -0.10 | 0.06 | 0.05 | 0.03 | 0.04 | 0.02 | 0.04 | 0.01 | 0.03 | 0.04 | 0.02 |
| CFHR1 | -0.04 | -0.04 | -0.03 | 0.02 | 0.02 | 0.03 | 0.07 | 0.13 | -0.01 | 0.00 | 0.02 | 0.07 |
| CFHR2 | -0.03 | -0.06 | -0.01 | 0.08 | -0.04 | 0.08 | 0.01 | 0.07 | 0.00 | -0.08 | 0.02 | -0.03 |
| CFI | -0.01 | -0.10 | 0.02 | 0.05 | -0.04 | 0.06 | -0.02 | 0.02 | 0.04 | -0.02 | 0.01 | -0.01 |
| CFL1 | 0.07 | -0.02 | -0.10 | -0.02 | -0.07 | -0.02 | 0.03 | 0.04 | 0.00 | -0.03 | -0.04 | 0.05 |
| CGA | 0.00 | -0.06 | -0.01 | -0.02 | -0.02 | 0.07 | -0.07 | 0.11 | -0.05 | -0.06 | -0.06 | -0.02 |
| CHGA | 0.03 | -0.05 | -0.13 | -0.04 | -0.02 | -0.06 | 0.04 | -0.04 | 0.00 | -0.04 | 0.02 | -0.03 |
| CHRDL1 | 0.09 | 0.03 | 0.05 | -0.02 | 0.01 | -0.03 | 0.03 | -0.07 | 0.00 | 0.00 | -0.03 | 0.01 |
| CHRDL2 | 0.03 | -0.05 | 0.01 | 0.09 | -0.08 | 0.07 | -0.01 | 0.03 | -0.03 | 0.01 | 0.04 | -0.05 |
| CLCA1 | 0.02 | 0.02 | -0.04 | -0.05 | 0.04 | 0.06 | 0.03 | -0.02 | -0.08 | -0.08 | -0.07 | -0.04 |
| CLEC3B | 0.02 | -0.03 | 0.04 | 0.01 | 0.01 | 0.08 | 0.12 | -0.02 | -0.01 | 0.00 | 0.08 | -0.02 |
| CLIC1 | 0.05 | -0.05 | -0.07 | -0.02 | -0.06 | -0.02 | -0.01 | 0.03 | -0.03 | -0.07 | -0.05 | 0.00 |
| CLIC4 | 0.05 | -0.10 | -0.05 | -0.03 | 0.03 | -0.01 | 0.01 | -0.07 | 0.04 | -0.04 | -0.01 | -0.05 |
| CLPS | 0.05 | 0.03 | 0.02 | 0.06 | 0.07 | 0.05 | -0.04 | -0.07 | -0.02 | -0.01 | -0.03 | 0.00 |
| CLSTN1 | -0.01 | -0.02 | -0.02 | 0.02 | 0.05 | 0.02 | -0.05 | 0.02 | -0.02 | -0.02 | -0.02 | -0.09 |
| COL12A1 | 0.01 | 0.06 | -0.07 | 0.06 | 0.01 | 0.02 | 0.12 | 0.03 | -0.01 | 0.06 | 0.05 | -0.01 |

| | | | | | | | | | | | | |
|---|---|---|---|---|---|---|---|---|---|---|---|---|
| COL15A1 | 0.06 | 0.00 | -0.10 | 0.09 | 0.04 | -0.01 | 0.01 | -0.02 | 0.03 | -0.01 | 0.06 | -0.02 |
| COL16A1 | 0.00 | 0.04 | -0.06 | 0.04 | -0.07 | 0.01 | 0.08 | -0.05 | -0.04 | 0.03 | 0.00 | -0.03 |
| COL1A1 | -0.01 | 0.08 | -0.02 | 0.10 | 0.04 | 0.06 | 0.03 | -0.02 | -0.02 | -0.01 | 0.01 | -0.08 |
| COL1A2 | 0.01 | 0.09 | -0.04 | 0.11 | 0.02 | 0.03 | 0.03 | 0.01 | 0.02 | 0.00 | -0.03 | -0.06 |
| COL2A1 | 0.01 | -0.02 | -0.10 | 0.03 | -0.02 | 0.04 | 0.04 | 0.02 | 0.00 | 0.04 | 0.06 | -0.02 |
| COL3A1 | 0.03 | 0.05 | -0.04 | 0.11 | 0.02 | 0.04 | 0.01 | -0.02 | -0.03 | -0.07 | -0.02 | -0.05 |
| COL4A2 | 0.04 | -0.03 | -0.05 | -0.02 | 0.07 | -0.08 | 0.07 | -0.01 | 0.02 | 0.07 | 0.05 | 0.05 |
| COL5A2 | 0.05 | 0.09 | 0.00 | 0.06 | 0.07 | 0.02 | 0.00 | -0.05 | 0.07 | 0.03 | -0.07 | -0.02 |
| COL6A1 | 0.06 | 0.09 | 0.00 | 0.06 | 0.05 | 0.01 | 0.00 | 0.01 | 0.00 | -0.03 | 0.02 | 0.01 |
| COLEC12 | 0.07 | -0.01 | -0.06 | -0.05 | 0.09 | -0.03 | -0.04 | -0.01 | 0.04 | -0.04 | 0.09 | -0.04 |
| COMP | 0.08 | 0.05 | 0.00 | 0.02 | 0.00 | 0.02 | 0.02 | 0.04 | 0.10 | 0.02 | -0.02 | 0.03 |
| CP | -0.01 | -0.02 | 0.04 | -0.08 | 0.05 | 0.02 | 0.04 | 0.01 | 0.11 | 0.09 | 0.06 | 0.05 |
| CPA1 | 0.05 | -0.06 | 0.00 | 0.00 | 0.05 | 0.06 | -0.11 | -0.09 | 0.01 | -0.01 | -0.05 | 0.04 |
| CRISP3 | 0.06 | -0.07 | -0.01 | -0.01 | 0.02 | 0.00 | 0.02 | -0.05 | 0.04 | 0.05 | 0.02 | -0.03 |
| CST3 | 0.10 | 0.03 | -0.02 | -0.06 | 0.04 | 0.03 | -0.02 | 0.01 | 0.05 | 0.04 | 0.01 | -0.03 |
| CST6 | 0.05 | 0.01 | -0.02 | -0.11 | 0.05 | 0.00 | 0.08 | -0.05 | 0.04 | -0.02 | 0.05 | 0.02 |
| CSTB | 0.05 | 0.06 | 0.00 | -0.07 | 0.01 | 0.05 | 0.07 | 0.06 | -0.06 | 0.02 | -0.01 | 0.01 |
| CTGF | 0.02 | -0.04 | -0.01 | -0.01 | 0.07 | 0.04 | 0.03 | -0.10 | 0.05 | 0.01 | 0.08 | -0.02 |
| DAG1 | 0.03 | 0.12 | -0.04 | -0.04 | 0.05 | 0.05 | -0.02 | 0.00 | 0.07 | 0.04 | 0.02 | 0.01 |
| DBI | 0.06 | 0.07 | -0.02 | 0.00 | 0.05 | -0.06 | 0.00 | 0.05 | 0.01 | 0.04 | -0.02 | -0.02 |
| DCN | 0.07 | 0.06 | -0.02 | 0.09 | 0.01 | 0.01 | 0.07 | -0.01 | 0.00 | -0.03 | 0.01 | -0.01 |
| DLK1 | 0.04 | -0.04 | -0.12 | -0.03 | -0.01 | 0.03 | 0.03 | -0.10 | 0.02 | 0.01 | -0.02 | -0.01 |
| DMBT1 | 0.03 | 0.01 | -0.01 | -0.04 | 0.07 | 0.00 | -0.06 | -0.08 | -0.06 | -0.06 | 0.03 | -0.04 |
| DMKN | 0.07 | -0.04 | 0.04 | -0.03 | -0.07 | 0.09 | -0.05 | 0.02 | 0.02 | -0.01 | -0.07 | 0.07 |
| DPP4 | 0.03 | -0.03 | -0.11 | -0.03 | 0.00 | -0.03 | -0.04 | 0.02 | 0.01 | 0.01 | -0.06 | -0.09 |
| DSC2 | 0.02 | 0.04 | -0.08 | 0.01 | -0.06 | -0.03 | 0.02 | -0.05 | 0.02 | 0.01 | -0.04 | 0.10 |
| DSC3 | 0.00 | 0.06 | -0.03 | 0.01 | 0.05 | 0.03 | 0.02 | -0.04 | -0.02 | 0.02 | -0.03 | -0.07 |
| DSG2 | 0.02 | 0.06 | -0.04 | 0.03 | 0.08 | 0.03 | -0.03 | -0.02 | 0.03 | -0.08 | 0.07 | -0.01 |
| ECM1 | 0.10 | 0.02 | 0.05 | -0.03 | 0.01 | -0.04 | -0.01 | 0.01 | -0.02 | -0.01 | 0.05 | -0.05 |
| EEF1A1 | 0.00 | -0.01 | -0.07 | -0.05 | -0.08 | -0.05 | 0.00 | 0.01 | -0.04 | -0.04 | 0.01 | 0.04 |
| EFEMP1 | 0.11 | 0.01 | -0.02 | -0.04 | 0.06 | -0.04 | -0.03 | 0.00 | 0.00 | 0.03 | 0.00 | -0.06 |

| | | | | | | | | | | | | |
|---|---|---|---|---|---|---|---|---|---|---|---|---|
| EFEMP2 | 0.06 | 0.02 | 0.01 | 0.07 | 0.00 | 0.06 | 0.01 | 0.07 | 0.04 | 0.04 | 0.01 | -0.08 |
| EFNA1 | 0.03 | 0.08 | -0.02 | 0.00 | -0.02 | 0.04 | -0.07 | 0.05 | 0.08 | -0.05 | -0.04 | -0.01 |
| EFNB1 | 0.04 | 0.00 | -0.07 | 0.04 | 0.07 | -0.01 | -0.03 | -0.04 | -0.03 | -0.01 | 0.07 | 0.03 |
| ENO1 | 0.07 | -0.02 | -0.02 | 0.04 | -0.03 | -0.07 | -0.02 | -0.01 | 0.00 | 0.00 | -0.02 | -0.02 |
| ENPP2 | 0.11 | -0.03 | 0.04 | -0.04 | -0.02 | -0.04 | -0.03 | 0.02 | 0.01 | -0.02 | 0.01 | 0.01 |
| ENPP7 | 0.00 | 0.05 | -0.05 | -0.04 | 0.01 | 0.01 | -0.07 | -0.10 | 0.03 | 0.02 | -0.12 | -0.01 |
| EZR | 0.07 | -0.06 | -0.05 | -0.03 | 0.07 | -0.01 | -0.06 | 0.04 | 0.03 | 0.05 | -0.05 | -0.04 |
| F10 | 0.04 | -0.09 | -0.09 | -0.01 | -0.09 | -0.06 | 0.00 | 0.01 | -0.02 | -0.03 | -0.03 | -0.03 |
| F11R | -0.04 | 0.03 | -0.03 | 0.00 | -0.06 | 0.05 | -0.03 | -0.01 | -0.06 | 0.03 | 0.02 | 0.03 |
| F12 | 0.00 | -0.14 | -0.01 | 0.02 | 0.02 | 0.03 | 0.05 | 0.00 | -0.02 | 0.04 | 0.02 | 0.01 |
| F13B | -0.02 | 0.00 | 0.02 | 0.03 | -0.02 | 0.09 | 0.05 | 0.01 | -0.07 | -0.02 | 0.03 | -0.09 |
| F2 | 0.02 | -0.11 | -0.08 | -0.01 | -0.08 | -0.05 | -0.01 | 0.04 | -0.02 | -0.03 | -0.02 | -0.01 |
| FABP1 | -0.01 | 0.04 | -0.07 | 0.00 | 0.00 | 0.12 | 0.00 | -0.01 | -0.04 | -0.03 | -0.08 | -0.05 |
| FABP5 | 0.10 | -0.04 | 0.00 | 0.01 | 0.00 | 0.04 | 0.02 | -0.01 | -0.06 | -0.01 | -0.02 | -0.03 |
| FAM151A | 0.01 | 0.02 | -0.06 | 0.00 | -0.08 | 0.03 | -0.02 | 0.03 | 0.04 | 0.02 | -0.01 | 0.05 |
| FBLN2 | 0.06 | -0.04 | -0.10 | 0.03 | -0.06 | -0.06 | 0.02 | 0.01 | -0.04 | -0.01 | 0.03 | 0.02 |
| FBN1 | 0.05 | -0.01 | -0.02 | 0.04 | 0.01 | -0.03 | 0.07 | 0.06 | -0.06 | 0.07 | -0.02 | 0.04 |
| FCGBP | 0.03 | -0.03 | 0.02 | -0.08 | 0.04 | 0.04 | 0.02 | -0.06 | -0.07 | -0.08 | 0.05 | -0.03 |
| FETUB | 0.00 | -0.09 | 0.03 | 0.06 | -0.01 | 0.05 | 0.00 | -0.02 | 0.01 | 0.05 | -0.01 | 0.02 |
| FGA | 0.00 | -0.03 | -0.04 | 0.10 | -0.08 | -0.03 | -0.01 | -0.06 | 0.04 | 0.02 | 0.09 | -0.01 |
| FGB | 0.08 | -0.01 | 0.04 | 0.03 | -0.06 | 0.05 | 0.03 | 0.01 | 0.05 | 0.04 | 0.07 | 0.02 |
| FGG | 0.04 | 0.00 | 0.06 | 0.04 | -0.08 | 0.09 | 0.03 | 0.01 | -0.02 | -0.03 | 0.04 | 0.02 |
| FLG | 0.02 | -0.04 | 0.05 | -0.09 | 0.04 | -0.02 | 0.04 | 0.04 | -0.09 | 0.05 | 0.05 | 0.06 |
| FLT1 | 0.02 | 0.04 | 0.04 | -0.03 | 0.04 | 0.11 | 0.09 | 0.01 | -0.11 | 0.03 | -0.01 | 0.04 |
| FMOD | 0.03 | 0.07 | -0.07 | 0.07 | 0.01 | 0.05 | 0.00 | -0.02 | 0.06 | -0.02 | -0.01 | 0.02 |
| FN1 | 0.08 | 0.08 | 0.00 | 0.04 | 0.02 | 0.01 | 0.11 | 0.00 | -0.05 | 0.02 | 0.03 | 0.00 |
| FOLH1 | 0.05 | 0.00 | -0.12 | -0.05 | -0.02 | -0.07 | -0.03 | 0.00 | 0.03 | -0.03 | -0.03 | 0.00 |
| FSTL1 | 0.08 | 0.02 | 0.06 | 0.10 | 0.05 | 0.03 | 0.00 | 0.03 | -0.03 | 0.05 | -0.01 | -0.03 |
| FSTL3 | 0.04 | 0.02 | -0.07 | -0.02 | -0.04 | 0.07 | 0.09 | -0.06 | 0.00 | 0.05 | 0.07 | 0.05 |
| FUCA2 | 0.04 | 0.00 | -0.12 | -0.07 | -0.06 | 0.00 | 0.02 | -0.02 | 0.00 | -0.02 | 0.02 | 0.07 |
| GAPDH | 0.09 | -0.06 | -0.01 | 0.06 | 0.03 | 0.00 | 0.00 | -0.05 | -0.01 | 0.00 | -0.01 | -0.04 |

| | | | | | | | | | | | | |
|---|---|---|---|---|---|---|---|---|---|---|---|---|
| GGT1 | 0.00 | 0.03 | -0.07 | 0.00 | 0.00 | 0.09 | -0.12 | 0.10 | 0.02 | 0.04 | 0.01 | 0.09 |
| GM2A | 0.06 | 0.04 | -0.02 | -0.02 | -0.01 | 0.08 | 0.05 | 0.08 | -0.04 | -0.02 | -0.01 | 0.01 |
| GOLM1 | 0.03 | 0.06 | -0.03 | 0.04 | 0.00 | -0.02 | 0.01 | -0.09 | 0.10 | 0.09 | 0.01 | -0.03 |
| GPX3 | 0.05 | 0.04 | -0.07 | -0.02 | -0.08 | 0.04 | 0.04 | -0.01 | 0.06 | 0.08 | 0.01 | 0.03 |
| GRN | 0.07 | -0.04 | 0.04 | -0.05 | -0.03 | 0.06 | -0.06 | -0.05 | 0.02 | -0.04 | -0.06 | 0.07 |
| GSTP1 | 0.09 | -0.03 | -0.02 | -0.04 | 0.00 | 0.00 | 0.00 | 0.02 | -0.04 | -0.05 | 0.02 | -0.01 |
| HABP2 | -0.03 | -0.12 | -0.05 | 0.01 | 0.02 | 0.04 | 0.01 | -0.01 | 0.00 | -0.02 | 0.02 | -0.02 |
| HAPLN1 | 0.06 | 0.01 | -0.05 | 0.05 | 0.06 | -0.03 | 0.07 | 0.05 | 0.01 | -0.03 | 0.05 | -0.03 |
| HAPLN3 | 0.00 | 0.01 | -0.04 | 0.10 | 0.05 | -0.01 | 0.03 | 0.11 | 0.02 | -0.10 | -0.02 | 0.11 |
| HBA1 | 0.00 | 0.02 | -0.01 | 0.14 | 0.00 | -0.07 | 0.00 | 0.02 | -0.03 | 0.07 | -0.07 | 0.05 |
| HBB | 0.00 | -0.03 | -0.02 | 0.13 | -0.08 | 0.00 | -0.03 | 0.02 | -0.02 | 0.03 | 0.04 | -0.01 |
| HPR | 0.00 | -0.02 | 0.05 | 0.01 | 0.00 | -0.08 | 0.07 | -0.09 | -0.07 | 0.01 | -0.07 | -0.05 |
| HPX | -0.03 | -0.11 | 0.03 | -0.01 | 0.05 | 0.02 | 0.04 | 0.00 | 0.04 | 0.05 | 0.00 | -0.03 |
| HRG | -0.01 | -0.05 | -0.04 | 0.03 | 0.00 | 0.11 | 0.08 | -0.03 | 0.02 | -0.06 | -0.06 | -0.02 |
| HRNR | 0.04 | 0.01 | -0.07 | -0.08 | -0.02 | -0.01 | 0.04 | 0.05 | -0.02 | 0.02 | 0.02 | 0.11 |
| HSP90AA1 | 0.06 | -0.07 | -0.09 | -0.05 | -0.07 | -0.02 | 0.02 | 0.01 | -0.04 | -0.02 | -0.04 | 0.00 |
| HSP90B1 | -0.01 | -0.03 | -0.07 | 0.01 | -0.12 | -0.02 | 0.04 | -0.04 | -0.05 | -0.07 | 0.07 | 0.03 |
| HSPA8 | 0.00 | 0.01 | 0.01 | 0.05 | -0.01 | -0.06 | -0.05 | -0.04 | -0.13 | 0.14 | -0.04 | -0.11 |
| HSPG2 | 0.04 | 0.03 | -0.12 | -0.05 | 0.00 | 0.06 | 0.05 | -0.02 | -0.01 | -0.01 | 0.04 | -0.01 |
| HTRA1 | 0.07 | 0.02 | -0.02 | 0.02 | 0.04 | 0.00 | 0.05 | 0.02 | 0.07 | 0.11 | -0.02 | 0.01 |
| IGF2 | 0.04 | 0.01 | 0.01 | 0.05 | 0.08 | 0.09 | 0.02 | 0.00 | -0.01 | -0.04 | -0.01 | -0.01 |
| IGFBP2 | 0.09 | 0.04 | 0.06 | -0.02 | 0.01 | -0.05 | 0.02 | 0.00 | 0.05 | -0.01 | 0.02 | -0.02 |
| IGFBP3 | 0.08 | 0.03 | -0.04 | 0.03 | -0.06 | -0.08 | 0.03 | 0.07 | 0.02 | 0.00 | 0.04 | 0.02 |
| IGFBP4 | 0.09 | 0.06 | 0.04 | -0.04 | -0.03 | -0.02 | 0.04 | 0.05 | 0.01 | 0.02 | 0.00 | -0.01 |
| IGFBP5 | 0.04 | 0.02 | 0.06 | 0.00 | -0.01 | -0.03 | 0.02 | -0.03 | 0.14 | 0.03 | -0.11 | -0.04 |
| IGFBP6 | 0.07 | -0.04 | 0.05 | 0.01 | 0.04 | -0.03 | 0.03 | -0.02 | -0.01 | 0.02 | 0.01 | 0.01 |
| IGFBP7 | 0.08 | 0.05 | 0.03 | -0.03 | -0.06 | 0.00 | 0.02 | -0.01 | 0.08 | -0.01 | -0.06 | 0.00 |
| IGHA1 | -0.04 | -0.04 | 0.02 | 0.02 | -0.05 | -0.01 | -0.04 | -0.02 | -0.13 | -0.02 | 0.06 | 0.00 |
| IGHA2 | -0.03 | -0.01 | -0.02 | -0.08 | -0.07 | 0.07 | -0.11 | -0.01 | -0.04 | 0.01 | 0.01 | -0.02 |
| IGHG2 | 0.01 | 0.00 | 0.03 | 0.11 | 0.04 | -0.09 | 0.05 | -0.02 | -0.04 | -0.04 | -0.02 | 0.08 |
| IGHG3 | 0.00 | 0.00 | 0.02 | 0.11 | 0.05 | -0.08 | 0.03 | 0.01 | -0.04 | -0.01 | 0.07 | 0.05 |

| | | | | | | | | | | | | |
|---|---|---|---|---|---|---|---|---|---|---|---|---|
| IGHG4 | -0.03 | 0.00 | -0.02 | -0.01 | 0.00 | -0.05 | -0.02 | 0.09 | -0.06 | 0.05 | -0.07 | -0.06 |
| IGHV1-18 | 0.02 | 0.09 | -0.01 | -0.02 | 0.05 | 0.00 | -0.06 | 0.04 | 0.05 | -0.02 | 0.05 | 0.00 |
| IGHV1-3 | 0.00 | 0.00 | 0.04 | 0.01 | -0.04 | -0.06 | 0.00 | -0.10 | -0.06 | 0.13 | -0.02 | -0.11 |
| IGHV1-69 | 0.02 | -0.01 | 0.03 | -0.02 | 0.04 | -0.01 | -0.09 | 0.09 | 0.00 | 0.04 | 0.05 | 0.08 |
| IGHV1OR15- | -0.03 | 0.01 | 0.01 | -0.05 | -0.01 | 0.03 | -0.08 | -0.03 | -0.05 | -0.02 | 0.11 | 0.02 |
| IGHV3-15 | 0.04 | 0.01 | 0.01 | 0.07 | 0.00 | -0.02 | 0.09 | -0.04 | 0.06 | 0.03 | 0.02 | 0.05 |
| IGHV3-38 | 0.01 | 0.00 | 0.03 | 0.07 | 0.03 | 0.01 | -0.01 | -0.11 | -0.01 | 0.04 | -0.09 | 0.00 |
| IGHV3-43 | 0.00 | -0.01 | 0.02 | 0.07 | -0.02 | -0.03 | 0.02 | 0.06 | -0.07 | -0.08 | -0.12 | 0.08 |
| IGHV3-49 | 0.04 | 0.01 | 0.03 | 0.09 | 0.04 | -0.08 | 0.03 | 0.01 | 0.02 | -0.01 | -0.01 | 0.02 |
| IGHV3-53 | 0.01 | -0.02 | 0.01 | 0.03 | 0.08 | -0.03 | 0.01 | -0.07 | -0.03 | 0.01 | -0.08 | -0.03 |
| IGHV3-66 | -0.01 | 0.05 | 0.00 | 0.00 | -0.03 | -0.03 | -0.06 | -0.09 | 0.03 | -0.02 | 0.07 | 0.07 |
| IGHV3-7 | -0.04 | 0.05 | 0.06 | 0.05 | -0.05 | 0.00 | 0.07 | 0.00 | 0.00 | -0.01 | -0.10 | 0.06 |
| IGHV3-72 | 0.04 | 0.04 | 0.04 | 0.03 | 0.02 | 0.00 | -0.01 | 0.02 | 0.00 | -0.04 | -0.09 | 0.08 |
| IGHV3-73 | 0.03 | 0.02 | -0.04 | 0.00 | 0.01 | 0.01 | -0.01 | -0.07 | -0.03 | 0.09 | 0.00 | 0.04 |
| IGHV3-9 | 0.00 | -0.01 | 0.02 | 0.12 | 0.01 | -0.09 | 0.09 | -0.02 | 0.02 | -0.02 | -0.07 | 0.08 |
| IGHV3OR16- | 0.04 | -0.06 | -0.02 | 0.04 | 0.09 | 0.02 | -0.06 | -0.06 | 0.01 | -0.01 | 0.07 | 0.05 |
| IGHV4-28 | 0.02 | -0.03 | 0.03 | 0.02 | 0.07 | -0.02 | -0.04 | 0.02 | -0.03 | -0.01 | 0.02 | 0.03 |
| IGHV5-51 | 0.04 | -0.03 | 0.06 | -0.08 | -0.05 | 0.03 | -0.04 | -0.06 | -0.07 | 0.05 | -0.02 | -0.13 |
| IGHV6-1 | 0.00 | -0.02 | -0.02 | 0.07 | 0.02 | -0.03 | -0.03 | -0.08 | -0.01 | 0.00 | -0.08 | 0.09 |
| IGKC | 0.00 | 0.00 | 0.01 | 0.08 | 0.03 | -0.08 | -0.09 | -0.06 | -0.09 | -0.06 | 0.05 | 0.08 |
| IGKV1-16 | 0.04 | -0.01 | 0.02 | -0.06 | 0.07 | 0.04 | -0.08 | 0.02 | 0.03 | -0.06 | 0.02 | 0.04 |
| IGKV1-5 | 0.04 | -0.07 | -0.07 | 0.04 | 0.06 | 0.03 | -0.05 | -0.10 | -0.02 | -0.05 | -0.01 | 0.03 |
| IGKV2-24 | 0.02 | 0.01 | 0.03 | 0.00 | 0.07 | -0.01 | 0.01 | -0.09 | -0.06 | -0.08 | -0.04 | -0.02 |
| IGKV2-28 | 0.03 | -0.02 | 0.04 | 0.03 | 0.09 | -0.07 | -0.05 | -0.10 | -0.04 | -0.02 | -0.05 | 0.04 |
| IGKV2-30 | 0.03 | 0.00 | 0.08 | 0.03 | 0.04 | -0.04 | -0.03 | -0.06 | 0.00 | -0.05 | -0.07 | 0.06 |
| IGKV3-15 | 0.00 | -0.02 | -0.01 | 0.08 | 0.08 | -0.03 | -0.04 | 0.01 | -0.07 | 0.04 | -0.06 | 0.05 |
| IGKV3-20 | 0.00 | 0.01 | 0.01 | 0.03 | 0.02 | -0.07 | -0.09 | -0.05 | -0.05 | -0.04 | 0.01 | 0.12 |
| IGKV3-7 | 0.00 | 0.02 | -0.06 | 0.01 | 0.02 | 0.06 | -0.07 | 0.10 | 0.00 | -0.04 | -0.03 | 0.11 |
| IGKV3D-11 | 0.05 | 0.00 | 0.04 | -0.01 | 0.07 | -0.12 | -0.03 | -0.04 | -0.03 | 0.05 | 0.04 | 0.06 |
| IGKV4-1 | 0.04 | -0.02 | 0.01 | 0.05 | 0.09 | 0.01 | -0.06 | -0.03 | -0.09 | 0.07 | 0.02 | 0.07 |
| IGLC7 | 0.00 | 0.02 | -0.01 | 0.03 | -0.04 | 0.01 | -0.09 | -0.01 | -0.01 | 0.15 | -0.01 | 0.01 |

| | | | | | | | | | | | | |
|---|---|---|---|---|---|---|---|---|---|---|---|---|
| IGLL5 | -0.04 | 0.04 | 0.00 | 0.05 | -0.02 | 0.00 | -0.06 | -0.09 | -0.04 | 0.11 | 0.10 | 0.03 |
| IGLV1-47 | -0.07 | -0.03 | 0.02 | 0.01 | -0.03 | -0.05 | 0.05 | -0.07 | -0.02 | 0.04 | -0.01 | 0.05 |
| IGLV1-51 | -0.01 | -0.01 | 0.08 | 0.03 | 0.04 | 0.06 | -0.04 | -0.05 | -0.09 | -0.05 | 0.05 | 0.01 |
| IGLV10-54 | -0.02 | 0.00 | 0.02 | 0.00 | 0.01 | 0.05 | 0.04 | 0.01 | -0.06 | -0.09 | -0.06 | 0.05 |
| IGLV2-11 | 0.02 | 0.06 | 0.09 | -0.02 | 0.07 | -0.02 | -0.05 | 0.05 | -0.08 | -0.02 | 0.00 | 0.01 |
| IGLV2-8 | 0.04 | 0.07 | 0.09 | -0.03 | -0.02 | -0.02 | 0.00 | -0.07 | -0.01 | 0.04 | 0.01 | -0.02 |
| IGLV3-10 | 0.01 | 0.01 | 0.02 | -0.01 | 0.01 | -0.05 | -0.07 | 0.00 | 0.02 | -0.02 | -0.03 | 0.02 |
| IGLV3-19 | 0.04 | -0.03 | 0.05 | 0.03 | 0.01 | 0.02 | -0.04 | -0.01 | 0.07 | -0.05 | 0.00 | -0.10 |
| IGLV3-21 | 0.02 | -0.02 | -0.03 | 0.05 | 0.04 | -0.02 | -0.01 | 0.03 | -0.02 | 0.09 | 0.01 | 0.04 |
| IGSF1 | 0.02 | 0.01 | -0.10 | 0.00 | -0.03 | 0.06 | -0.04 | -0.07 | 0.07 | 0.02 | -0.01 | 0.00 |
| IL1R2 | 0.06 | 0.01 | 0.03 | 0.03 | -0.09 | -0.07 | 0.02 | -0.06 | 0.00 | -0.02 | -0.05 | 0.04 |
| IL1RAP | 0.00 | 0.09 | -0.02 | 0.01 | -0.02 | 0.06 | 0.01 | 0.03 | 0.04 | 0.01 | -0.06 | 0.05 |
| INA | -0.03 | -0.03 | 0.04 | -0.03 | -0.02 | 0.00 | 0.07 | -0.05 | 0.07 | -0.04 | 0.07 | -0.06 |
| ISLR | 0.08 | -0.01 | 0.03 | 0.03 | -0.01 | -0.03 | 0.05 | 0.04 | 0.02 | -0.02 | -0.08 | 0.01 |
| ISM2 | 0.09 | -0.01 | -0.01 | -0.04 | 0.00 | -0.03 | 0.06 | -0.01 | -0.03 | 0.05 | -0.06 | 0.02 |
| ITIH1 | 0.05 | -0.08 | 0.07 | 0.02 | 0.00 | 0.07 | 0.03 | 0.06 | 0.04 | -0.03 | -0.03 | -0.01 |
| ITIH2 | 0.00 | -0.08 | 0.00 | 0.01 | 0.01 | 0.06 | 0.07 | 0.03 | 0.05 | 0.01 | -0.06 | 0.02 |
| IVL | 0.07 | -0.04 | 0.01 | -0.07 | 0.07 | 0.00 | 0.03 | -0.02 | -0.03 | 0.04 | 0.01 | 0.01 |
| KLKB1 | 0.01 | -0.08 | 0.02 | -0.02 | 0.03 | 0.01 | 0.04 | 0.05 | -0.05 | 0.01 | -0.07 | 0.03 |
| KNG1 | -0.05 | -0.05 | 0.04 | 0.03 | -0.01 | 0.08 | 0.10 | -0.04 | -0.03 | 0.04 | 0.03 | 0.08 |
| KRT1 | 0.02 | 0.00 | -0.07 | -0.05 | 0.05 | -0.03 | -0.03 | 0.06 | -0.09 | 0.04 | 0.09 | 0.07 |
| KRT10 | 0.02 | 0.01 | -0.05 | -0.02 | 0.06 | -0.04 | -0.01 | 0.05 | -0.10 | 0.03 | 0.10 | 0.09 |
| KRT13 | -0.01 | 0.02 | 0.01 | -0.05 | 0.03 | 0.06 | 0.00 | 0.10 | -0.08 | 0.00 | -0.01 | 0.07 |
| KRT14 | 0.02 | 0.01 | -0.04 | -0.04 | 0.05 | -0.04 | -0.01 | 0.03 | -0.09 | 0.05 | 0.09 | 0.09 |
| KRT19 | 0.10 | 0.00 | -0.03 | 0.00 | 0.01 | 0.01 | -0.04 | 0.01 | 0.01 | 0.00 | 0.04 | 0.01 |
| KRT2 | 0.02 | 0.03 | -0.06 | -0.03 | 0.02 | -0.03 | -0.01 | 0.10 | -0.09 | 0.05 | 0.04 | 0.06 |
| KRT5 | 0.00 | -0.02 | 0.02 | -0.02 | 0.03 | -0.04 | 0.07 | -0.02 | -0.07 | -0.08 | 0.00 | -0.09 |
| KRT79 | 0.00 | 0.01 | -0.03 | 0.12 | 0.05 | -0.05 | 0.05 | 0.09 | -0.01 | -0.08 | -0.02 | 0.05 |
| KRT8 | 0.06 | 0.04 | -0.02 | -0.02 | -0.11 | -0.01 | 0.00 | 0.03 | 0.00 | 0.01 | 0.06 | 0.04 |
| KRT84 | 0.00 | 0.02 | -0.01 | -0.02 | -0.05 | 0.02 | -0.12 | -0.04 | -0.06 | 0.16 | -0.05 | -0.01 |
| KRT9 | 0.02 | 0.03 | -0.09 | -0.02 | -0.01 | -0.05 | 0.03 | 0.08 | -0.04 | 0.03 | 0.01 | 0.06 |

| | | | | | | | | | | | | |
|---|---|---|---|---|---|---|---|---|---|---|---|---|
| LAMB1 | 0.07 | -0.03 | 0.02 | 0.07 | 0.03 | 0.02 | 0.00 | 0.00 | 0.03 | -0.01 | 0.07 | -0.02 |
| LAMC1 | 0.09 | 0.04 | -0.03 | 0.02 | 0.01 | -0.07 | 0.05 | -0.01 | 0.05 | 0.01 | 0.06 | -0.06 |
| LBP | -0.01 | -0.10 | -0.01 | 0.02 | -0.03 | -0.02 | 0.05 | -0.01 | 0.05 | 0.04 | 0.00 | -0.06 |
| LCN2 | 0.09 | 0.04 | 0.04 | 0.04 | -0.05 | -0.01 | 0.00 | -0.04 | -0.02 | 0.07 | 0.09 | -0.03 |
| LCP1 | 0.08 | -0.03 | 0.05 | 0.08 | -0.07 | 0.06 | -0.04 | 0.01 | -0.03 | 0.00 | 0.03 | -0.03 |
| LDHA | 0.10 | -0.05 | 0.03 | 0.04 | -0.04 | -0.01 | 0.00 | -0.03 | 0.02 | -0.04 | 0.00 | 0.00 |
| LDHB | 0.09 | -0.04 | -0.01 | 0.02 | -0.04 | 0.08 | -0.02 | -0.04 | -0.04 | 0.06 | -0.05 | -0.02 |
| LGALS1 | 0.10 | -0.01 | 0.04 | 0.04 | -0.02 | 0.04 | 0.03 | 0.05 | -0.01 | 0.01 | -0.06 | 0.03 |
| LGALS3BP | 0.07 | 0.06 | -0.02 | -0.01 | 0.05 | 0.02 | 0.06 | 0.01 | -0.06 | 0.00 | 0.01 | 0.00 |
| LOX | 0.09 | -0.02 | -0.05 | 0.04 | 0.03 | -0.03 | -0.05 | -0.04 | 0.05 | 0.02 | -0.02 | 0.05 |
| LRG1 | 0.00 | -0.10 | 0.04 | -0.04 | 0.01 | 0.02 | 0.03 | 0.02 | 0.10 | 0.05 | 0.06 | -0.02 |
| LTBP2 | 0.05 | 0.04 | 0.06 | 0.03 | 0.06 | 0.02 | -0.01 | -0.02 | 0.03 | 0.01 | 0.02 | -0.06 |
| LUM | 0.07 | 0.07 | 0.09 | -0.03 | 0.00 | -0.06 | 0.03 | 0.00 | -0.02 | 0.01 | 0.01 | -0.01 |
| LYPD3 | 0.05 | -0.02 | -0.06 | -0.07 | 0.06 | -0.03 | -0.05 | 0.09 | 0.07 | 0.02 | -0.02 | 0.02 |
| LYVE1 | -0.05 | 0.00 | 0.01 | 0.00 | -0.03 | 0.15 | -0.01 | -0.03 | 0.01 | 0.05 | -0.03 | -0.01 |
| LYZ | 0.07 | -0.05 | 0.03 | 0.03 | 0.05 | -0.01 | -0.07 | -0.01 | 0.01 | -0.03 | 0.06 | 0.04 |
| MAN1A1 | 0.00 | 0.02 | -0.12 | -0.04 | -0.01 | -0.01 | -0.01 | 0.01 | -0.05 | -0.05 | -0.05 | -0.08 |
| MARCKS | 0.07 | -0.05 | 0.00 | 0.02 | 0.02 | 0.04 | -0.02 | -0.10 | -0.04 | -0.06 | 0.01 | 0.05 |
| MASP2 | 0.01 | -0.06 | -0.03 | -0.03 | 0.01 | 0.04 | 0.08 | 0.09 | -0.01 | 0.08 | -0.04 | 0.01 |
| MATN2 | 0.06 | -0.01 | 0.02 | 0.09 | -0.03 | 0.04 | 0.03 | 0.04 | -0.05 | -0.03 | 0.06 | 0.03 |
| MEP1A | 0.02 | 0.02 | -0.08 | 0.00 | 0.08 | 0.06 | -0.10 | -0.02 | -0.02 | -0.04 | -0.06 | -0.07 |
| MFAP4 | 0.00 | -0.01 | 0.00 | 0.07 | 0.04 | 0.04 | 0.05 | -0.05 | 0.03 | 0.02 | -0.01 | -0.04 |
| MIF | 0.07 | 0.01 | 0.02 | -0.03 | -0.05 | 0.01 | 0.02 | 0.01 | 0.06 | 0.00 | 0.05 | -0.05 |
| MMP2 | 0.11 | -0.03 | 0.04 | -0.02 | 0.00 | -0.03 | -0.01 | 0.07 | -0.03 | 0.00 | 0.02 | -0.05 |
| MSN | 0.07 | 0.00 | 0.05 | -0.01 | -0.04 | 0.00 | -0.09 | 0.08 | -0.03 | -0.04 | 0.04 | 0.02 |
| MUC1 | 0.06 | -0.07 | -0.05 | 0.00 | 0.06 | 0.00 | -0.02 | -0.01 | -0.01 | -0.02 | 0.03 | 0.11 |
| MUC13 | 0.00 | -0.03 | -0.13 | -0.03 | 0.00 | -0.01 | 0.00 | -0.02 | -0.07 | -0.06 | -0.02 | -0.04 |
| MUC16 | 0.04 | -0.07 | 0.00 | -0.03 | 0.09 | 0.04 | 0.00 | -0.01 | -0.03 | -0.03 | -0.10 | 0.00 |
| MUC2 | 0.03 | -0.03 | -0.04 | -0.05 | 0.00 | 0.01 | 0.08 | -0.15 | 0.00 | 0.01 | -0.02 | 0.04 |
| MUC5AC | 0.02 | 0.01 | -0.11 | -0.08 | 0.00 | -0.03 | 0.06 | -0.06 | -0.04 | -0.03 | -0.01 | -0.01 |
| MUC5B | 0.05 | -0.03 | 0.01 | -0.07 | -0.04 | 0.03 | 0.05 | 0.00 | -0.09 | -0.07 | -0.06 | 0.02 |

| | | | | | | | | | | | | |
|---|---|---|---|---|---|---|---|---|---|---|---|---|
| MYDGF | 0.03 | 0.02 | -0.03 | 0.08 | 0.02 | 0.02 | -0.02 | 0.01 | -0.02 | 0.00 | 0.13 | -0.03 |
| MYL6 | 0.00 | 0.04 | 0.01 | 0.02 | 0.02 | -0.07 | -0.02 | 0.06 | 0.02 | 0.07 | -0.08 | -0.09 |
| NID1 | 0.07 | 0.00 | 0.00 | -0.01 | -0.05 | -0.01 | 0.04 | -0.03 | -0.13 | 0.05 | -0.02 | 0.01 |
| NID2 | 0.08 | 0.04 | 0.06 | -0.02 | 0.03 | 0.02 | 0.01 | 0.01 | -0.01 | -0.06 | 0.01 | -0.09 |
| NOTUM | -0.02 | 0.03 | 0.01 | -0.04 | 0.01 | 0.06 | 0.04 | -0.09 | -0.06 | 0.03 | 0.03 | 0.09 |
| NPC2 | 0.09 | 0.03 | 0.05 | -0.05 | -0.06 | 0.03 | -0.02 | -0.01 | 0.01 | -0.01 | -0.05 | 0.01 |
| NT5E | 0.00 | 0.05 | -0.07 | 0.00 | -0.05 | 0.09 | -0.06 | -0.03 | 0.04 | 0.00 | -0.09 | 0.06 |
| NUCB1 | 0.11 | 0.01 | -0.03 | 0.02 | -0.06 | -0.07 | 0.01 | 0.01 | 0.02 | 0.01 | -0.01 | 0.02 |
| OGN | 0.02 | 0.02 | -0.02 | -0.05 | 0.12 | -0.05 | -0.04 | 0.06 | 0.01 | -0.01 | 0.01 | -0.02 |
| OLFML3 | 0.06 | 0.05 | -0.02 | 0.08 | 0.02 | -0.01 | 0.03 | 0.05 | 0.05 | 0.05 | -0.10 | -0.03 |
| ORM1 | 0.03 | -0.09 | 0.06 | -0.05 | -0.05 | -0.01 | -0.03 | 0.00 | 0.09 | -0.01 | 0.02 | -0.07 |
| ORM2 | 0.00 | -0.08 | 0.06 | -0.06 | -0.03 | 0.01 | -0.04 | 0.01 | 0.07 | -0.01 | 0.05 | 0.00 |
| P4HB | 0.09 | 0.02 | 0.01 | 0.03 | -0.03 | -0.03 | -0.03 | 0.03 | 0.02 | -0.05 | 0.04 | -0.02 |
| PAPLN | 0.06 | -0.01 | 0.10 | -0.01 | -0.05 | -0.05 | -0.03 | 0.02 | 0.00 | -0.05 | 0.04 | -0.01 |
| PCOLCE | 0.04 | 0.07 | -0.03 | 0.10 | 0.02 | 0.05 | 0.05 | -0.01 | -0.06 | -0.04 | -0.02 | -0.03 |
| PDIA3 | 0.03 | 0.03 | 0.04 | -0.06 | 0.02 | -0.03 | -0.01 | 0.10 | 0.01 | 0.05 | 0.05 | 0.07 |
| PFN1 | 0.06 | -0.01 | 0.05 | 0.01 | -0.06 | 0.11 | 0.01 | 0.00 | 0.01 | 0.01 | -0.03 | -0.01 |
| PGAM1 | 0.10 | -0.02 | 0.08 | 0.02 | -0.04 | 0.01 | -0.03 | 0.02 | 0.02 | -0.05 | 0.02 | -0.03 |
| PGK1 | 0.09 | -0.02 | 0.07 | 0.00 | -0.04 | -0.05 | 0.02 | 0.05 | -0.03 | 0.00 | -0.04 | -0.03 |
| PGLYRP2 | 0.03 | -0.04 | -0.02 | 0.04 | 0.03 | 0.07 | -0.01 | 0.04 | -0.05 | -0.07 | -0.09 | -0.07 |
| PI16 | 0.00 | 0.00 | 0.03 | -0.03 | 0.01 | 0.08 | 0.10 | 0.08 | -0.04 | 0.00 | 0.02 | 0.01 |
| PIGR | 0.03 | 0.08 | 0.03 | -0.05 | 0.00 | 0.03 | 0.03 | 0.02 | 0.10 | 0.04 | -0.04 | 0.01 |
| PLG | -0.02 | -0.13 | -0.02 | 0.02 | 0.06 | 0.04 | 0.04 | 0.02 | -0.01 | 0.07 | 0.00 | 0.00 |
| PLOD1 | 0.00 | 0.01 | 0.00 | 0.12 | -0.01 | -0.05 | 0.02 | 0.00 | 0.03 | 0.04 | -0.09 | 0.14 |
| PLS3 | 0.05 | -0.04 | 0.02 | 0.06 | 0.00 | 0.01 | -0.07 | -0.07 | -0.06 | 0.07 | -0.10 | 0.01 |
| PLTP | 0.03 | -0.08 | -0.03 | 0.09 | -0.04 | -0.03 | -0.05 | -0.04 | -0.04 | -0.01 | 0.07 | -0.03 |
| PON1 | -0.05 | -0.06 | 0.00 | 0.03 | -0.08 | 0.00 | -0.03 | 0.09 | -0.03 | 0.00 | -0.01 | -0.06 |
| POSTN | 0.02 | 0.06 | -0.09 | 0.06 | -0.02 | 0.00 | 0.03 | -0.02 | -0.02 | 0.02 | 0.04 | -0.04 |
| POTEI | 0.00 | -0.02 | 0.02 | 0.01 | 0.03 | -0.03 | 0.12 | 0.00 | 0.07 | -0.16 | 0.00 | 0.07 |
| PPIA | 0.06 | -0.03 | -0.02 | 0.04 | 0.01 | 0.03 | -0.03 | -0.06 | 0.01 | 0.10 | -0.08 | 0.00 |
| PPIAL4A | 0.00 | 0.00 | 0.01 | 0.06 | -0.01 | -0.06 | 0.01 | -0.05 | -0.04 | 0.05 | -0.12 | 0.09 |

| | | | | | | | | | | | | |
|---|---|---|---|---|---|---|---|---|---|---|---|---|
| PRDX1 | -0.02 | 0.00 | -0.02 | -0.03 | -0.09 | 0.04 | -0.07 | -0.01 | 0.01 | -0.03 | 0.03 | 0.08 |
| PRDX2 | 0.03 | -0.03 | -0.06 | 0.10 | -0.04 | 0.04 | 0.03 | -0.07 | -0.02 | 0.03 | 0.04 | 0.02 |
| PRDX6 | 0.00 | 0.00 | 0.02 | 0.01 | -0.05 | 0.01 | 0.01 | -0.05 | 0.12 | 0.03 | -0.04 | 0.15 |
| PRG2 | 0.04 | -0.01 | 0.02 | -0.03 | 0.04 | 0.08 | 0.08 | -0.01 | -0.12 | 0.04 | 0.03 | 0.09 |
| PRL | 0.07 | 0.05 | 0.06 | 0.00 | -0.06 | -0.06 | 0.04 | -0.06 | -0.08 | -0.05 | 0.03 | 0.02 |
| PROCR | 0.00 | 0.03 | 0.03 | 0.00 | 0.05 | -0.04 | 0.06 | -0.02 | -0.03 | 0.13 | 0.01 | 0.03 |
| PROS1 | 0.03 | -0.06 | -0.06 | -0.02 | -0.09 | -0.07 | -0.01 | 0.02 | -0.04 | -0.06 | -0.02 | -0.01 |
| PRSS8 | 0.05 | 0.03 | -0.06 | -0.07 | -0.05 | -0.01 | -0.02 | 0.03 | -0.02 | 0.01 | -0.06 | 0.09 |
| PSAP | 0.09 | 0.00 | 0.04 | 0.04 | -0.01 | 0.04 | -0.06 | 0.04 | -0.01 | -0.06 | -0.04 | 0.06 |
| PSG1 | 0.06 | -0.06 | -0.06 | 0.00 | 0.06 | 0.01 | -0.07 | -0.03 | -0.04 | 0.04 | 0.10 | -0.03 |
| PSG9 | 0.00 | 0.03 | -0.04 | 0.10 | 0.00 | 0.00 | -0.09 | 0.06 | -0.05 | 0.11 | -0.05 | 0.05 |
| PTGDS | 0.04 | 0.05 | 0.01 | -0.06 | -0.05 | 0.08 | 0.06 | -0.08 | -0.08 | 0.01 | 0.00 | -0.02 |
| PTMA | -0.05 | 0.00 | 0.00 | 0.07 | -0.12 | -0.03 | -0.01 | -0.04 | -0.02 | 0.00 | 0.02 | -0.01 |
| PVR | 0.03 | 0.02 | 0.05 | -0.05 | 0.03 | -0.01 | -0.02 | 0.10 | -0.02 | 0.08 | -0.03 | -0.01 |
| PZP | 0.00 | -0.02 | 0.02 | -0.10 | 0.00 | 0.05 | 0.06 | -0.01 | 0.11 | -0.13 | 0.07 | 0.02 |
| QSOX1 | 0.04 | -0.04 | -0.10 | -0.07 | 0.07 | -0.03 | 0.03 | 0.04 | -0.02 | 0.04 | 0.00 | 0.00 |
| RBP4 | -0.02 | -0.08 | -0.04 | 0.00 | 0.00 | 0.04 | -0.05 | -0.04 | 0.11 | 0.00 | 0.08 | -0.07 |
| RCN1 | 0.03 | 0.06 | -0.02 | 0.04 | 0.05 | 0.00 | 0.02 | 0.05 | 0.12 | -0.01 | 0.05 | -0.02 |
| S100A11 | 0.00 | 0.03 | -0.07 | 0.03 | -0.11 | 0.02 | 0.04 | 0.01 | -0.09 | -0.02 | -0.06 | 0.01 |
| S100A6 | 0.02 | -0.03 | 0.08 | -0.04 | -0.04 | 0.00 | 0.01 | 0.08 | -0.02 | 0.11 | -0.04 | 0.05 |
| S100A8 | 0.07 | -0.05 | 0.06 | 0.01 | -0.08 | 0.07 | -0.01 | 0.01 | -0.01 | -0.01 | -0.06 | 0.01 |
| S100A9 | 0.08 | -0.06 | 0.06 | 0.03 | -0.05 | 0.09 | 0.00 | -0.01 | 0.04 | -0.04 | 0.00 | 0.02 |
| SCGB1A1 | -0.01 | 0.06 | 0.02 | -0.06 | 0.07 | -0.02 | 0.08 | -0.03 | -0.07 | 0.02 | -0.06 | -0.02 |
| SCGB3A1 | 0.02 | -0.07 | -0.08 | -0.06 | -0.05 | -0.04 | 0.06 | -0.03 | -0.05 | -0.03 | -0.04 | -0.01 |
| SCGB3A2 | 0.01 | -0.10 | 0.03 | -0.05 | 0.04 | 0.02 | 0.04 | -0.07 | 0.01 | 0.06 | -0.02 | -0.03 |
| SCUBE2 | 0.04 | 0.02 | -0.06 | -0.01 | 0.08 | -0.07 | -0.02 | 0.02 | 0.01 | 0.03 | 0.05 | 0.00 |
| SDF4 | -0.01 | 0.05 | -0.03 | 0.04 | -0.04 | 0.11 | -0.06 | 0.02 | 0.00 | 0.06 | 0.05 | -0.02 |
| SERPINA2 | 0.00 | -0.01 | 0.04 | 0.01 | -0.04 | -0.04 | 0.03 | -0.08 | 0.03 | 0.07 | 0.02 | -0.05 |
| SERPINA3 | 0.01 | -0.06 | 0.06 | 0.01 | 0.02 | -0.02 | 0.06 | 0.04 | -0.02 | 0.01 | 0.02 | -0.06 |
| SERPINA4 | -0.06 | -0.06 | -0.04 | 0.03 | -0.03 | 0.08 | 0.06 | 0.06 | -0.08 | 0.04 | -0.02 | 0.04 |
| SERPINA6 | 0.03 | -0.05 | -0.03 | 0.02 | 0.11 | 0.05 | 0.02 | 0.02 | 0.06 | 0.09 | 0.00 | 0.02 |

| | | | | | | | | | | | | |
|---|---|---|---|---|---|---|---|---|---|---|---|---|
| SERPINA7 | 0.02 | 0.00 | 0.00 | -0.05 | 0.00 | 0.04 | 0.01 | 0.11 | -0.02 | 0.09 | -0.05 | -0.04 |
| SERPINB2 | 0.03 | -0.01 | -0.04 | -0.05 | -0.01 | -0.04 | 0.05 | -0.07 | 0.04 | -0.06 | 0.10 | 0.12 |
| SERPINC1 | -0.08 | -0.05 | -0.02 | 0.01 | -0.02 | 0.05 | 0.07 | 0.02 | 0.02 | -0.03 | 0.00 | 0.01 |
| SERPIND1 | -0.02 | -0.09 | -0.03 | 0.06 | -0.03 | 0.00 | 0.04 | 0.04 | 0.03 | 0.01 | 0.01 | 0.01 |
| SERPINE2 | 0.08 | -0.01 | 0.00 | 0.08 | 0.06 | 0.03 | 0.03 | -0.02 | -0.02 | -0.02 | -0.08 | 0.03 |
| SERPINF1 | 0.03 | 0.01 | -0.06 | 0.02 | 0.08 | 0.02 | 0.06 | 0.06 | -0.03 | 0.07 | 0.04 | 0.01 |
| SERPINF2 | 0.03 | -0.11 | 0.01 | -0.01 | 0.04 | 0.05 | -0.04 | -0.01 | 0.06 | 0.02 | -0.02 | 0.01 |
| SERPING1 | 0.10 | 0.03 | 0.09 | -0.04 | -0.02 | 0.01 | 0.04 | 0.00 | 0.01 | -0.02 | -0.01 | 0.01 |
| SFN | 0.03 | -0.03 | 0.04 | -0.01 | -0.10 | 0.10 | -0.02 | 0.05 | -0.01 | -0.01 | -0.04 | 0.06 |
| SFTPB | 0.05 | -0.02 | 0.04 | -0.07 | 0.02 | -0.02 | 0.03 | 0.04 | -0.10 | 0.04 | 0.00 | 0.07 |
| SHBG | 0.02 | -0.07 | 0.01 | 0.07 | 0.08 | 0.07 | 0.02 | -0.01 | -0.01 | -0.04 | 0.08 | -0.01 |
| SHISA5 | 0.01 | 0.03 | -0.04 | -0.05 | -0.02 | 0.04 | 0.03 | 0.00 | 0.08 | 0.09 | -0.08 | 0.01 |
| SI | 0.03 | -0.04 | -0.13 | 0.00 | -0.01 | -0.02 | -0.03 | -0.04 | -0.04 | -0.05 | -0.06 | -0.01 |
| SLC15A1 | 0.00 | -0.01 | 0.01 | 0.03 | 0.05 | -0.06 | 0.06 | 0.01 | -0.10 | -0.06 | 0.00 | -0.12 |
| SLC38A10 | 0.00 | -0.01 | 0.00 | 0.00 | 0.02 | 0.03 | 0.07 | 0.05 | 0.13 | -0.12 | 0.06 | 0.07 |
| SLC9A3R1 | 0.01 | -0.04 | -0.14 | -0.03 | 0.01 | -0.04 | 0.00 | 0.03 | 0.00 | 0.00 | -0.02 | -0.03 |
| SLPI | 0.06 | -0.03 | 0.04 | -0.03 | -0.08 | 0.05 | -0.02 | -0.01 | 0.01 | 0.02 | -0.08 | 0.09 |
| SOD3 | 0.06 | -0.03 | 0.00 | -0.05 | -0.09 | -0.03 | 0.03 | -0.08 | 0.09 | 0.00 | 0.01 | 0.03 |
| SPARCL1 | 0.10 | -0.01 | -0.02 | -0.01 | -0.01 | -0.02 | -0.04 | 0.02 | -0.07 | -0.04 | 0.04 | 0.01 |
| SPINK1 | 0.04 | 0.02 | -0.06 | -0.02 | 0.06 | 0.06 | -0.09 | -0.04 | 0.03 | 0.02 | 0.01 | 0.00 |
| SPINK5 | 0.04 | -0.02 | 0.07 | -0.10 | 0.02 | 0.03 | 0.05 | 0.03 | -0.02 | 0.02 | -0.03 | 0.01 |
| SPON1 | 0.04 | -0.01 | -0.03 | 0.11 | -0.01 | -0.01 | 0.02 | -0.03 | 0.00 | 0.05 | -0.05 | -0.02 |
| SPON2 | 0.06 | -0.04 | 0.06 | -0.01 | 0.05 | 0.05 | 0.01 | -0.04 | 0.01 | 0.00 | 0.00 | 0.02 |
| SPP1 | 0.09 | 0.03 | -0.03 | 0.01 | -0.10 | -0.04 | -0.01 | 0.02 | 0.04 | 0.00 | 0.00 | -0.02 |
| SPRR1B | 0.01 | 0.02 | 0.02 | 0.00 | 0.00 | 0.04 | 0.02 | 0.01 | -0.05 | 0.11 | 0.04 | -0.01 |
| SPRR3 | 0.03 | 0.05 | -0.02 | -0.06 | 0.02 | 0.10 | 0.06 | 0.01 | 0.02 | 0.05 | 0.01 | 0.01 |
| STC2 | 0.08 | -0.01 | -0.01 | 0.04 | 0.03 | -0.09 | 0.00 | -0.01 | 0.05 | 0.04 | -0.03 | -0.02 |
| TAGLN | -0.01 | 0.07 | 0.02 | 0.06 | -0.03 | 0.04 | 0.08 | 0.00 | 0.01 | 0.06 | -0.02 | -0.07 |
| TFF3 | -0.02 | 0.01 | 0.05 | 0.00 | -0.04 | 0.11 | 0.11 | -0.07 | -0.08 | -0.02 | -0.04 | 0.00 |
| TGFBI | 0.04 | -0.01 | -0.06 | 0.06 | 0.05 | -0.04 | 0.01 | 0.06 | 0.03 | 0.06 | -0.05 | -0.09 |
| TGFBR3 | -0.03 | 0.06 | 0.00 | -0.02 | -0.01 | 0.11 | 0.04 | 0.04 | -0.05 | 0.07 | 0.02 | -0.04 |

| | | | | | | | | | | | | |
|---|---|---|---|---|---|---|---|---|---|---|---|---|
| THBS1 | 0.04 | -0.05 | -0.03 | 0.05 | 0.04 | 0.07 | 0.07 | 0.05 | -0.04 | 0.00 | 0.02 | -0.07 |
| THBS4 | 0.00 | 0.01 | -0.04 | 0.10 | 0.05 | -0.01 | 0.03 | 0.11 | 0.03 | -0.09 | 0.01 | 0.07 |
| THY1 | 0.04 | 0.00 | 0.03 | 0.03 | 0.06 | -0.01 | -0.01 | -0.14 | -0.02 | -0.05 | -0.02 | -0.04 |
| TIMP1 | 0.05 | -0.05 | -0.02 | 0.11 | -0.05 | -0.02 | -0.04 | -0.03 | -0.07 | 0.02 | 0.06 | 0.03 |
| TIMP2 | 0.08 | -0.02 | 0.09 | -0.01 | -0.03 | 0.01 | 0.00 | 0.08 | -0.02 | 0.04 | 0.04 | -0.03 |
| TKT | 0.08 | -0.04 | 0.07 | 0.05 | -0.07 | 0.05 | -0.02 | 0.01 | -0.01 | -0.01 | -0.02 | -0.02 |
| TMSB10 | 0.03 | 0.04 | -0.07 | 0.04 | -0.04 | 0.03 | 0.02 | 0.03 | 0.00 | 0.02 | 0.05 | 0.04 |
| TMSB4X | 0.03 | 0.03 | 0.01 | 0.03 | -0.06 | 0.08 | -0.05 | -0.04 | 0.00 | -0.06 | 0.08 | -0.07 |
| TPI1 | 0.06 | 0.01 | 0.01 | 0.03 | -0.02 | 0.11 | 0.00 | -0.03 | 0.04 | 0.00 | -0.07 | -0.04 |
| TTBK2 | 0.00 | -0.01 | 0.01 | -0.02 | -0.03 | 0.00 | -0.03 | -0.07 | -0.02 | 0.05 | -0.09 | 0.08 |
| TTN | -0.01 | 0.04 | 0.03 | 0.04 | 0.06 | 0.03 | 0.05 | 0.08 | -0.02 | -0.03 | -0.03 | 0.03 |
| TTR | -0.06 | -0.04 | 0.02 | 0.01 | -0.02 | 0.03 | 0.02 | 0.02 | 0.01 | -0.10 | -0.04 | -0.02 |
| TXN | 0.07 | 0.06 | 0.01 | 0.02 | -0.02 | 0.04 | -0.06 | 0.04 | -0.04 | -0.03 | 0.00 | -0.03 |
| TXNDC5 | -0.02 | 0.01 | 0.01 | 0.03 | 0.01 | 0.07 | 0.00 | -0.04 | -0.01 | -0.02 | -0.02 | 0.00 |
| VASN | 0.10 | -0.01 | -0.01 | -0.06 | -0.03 | -0.01 | 0.00 | 0.02 | 0.01 | 0.02 | -0.01 | -0.06 |
| VCAN | 0.05 | 0.00 | -0.06 | 0.01 | -0.11 | -0.06 | 0.02 | 0.01 | -0.06 | 0.00 | 0.01 | -0.01 |
| VSIG4 | 0.03 | -0.01 | -0.10 | -0.07 | 0.01 | 0.01 | 0.00 | -0.05 | -0.08 | 0.03 | -0.02 | 0.00 |
| VTN | 0.00 | -0.12 | -0.02 | -0.02 | 0.09 | -0.01 | 0.01 | 0.02 | -0.02 | 0.05 | 0.01 | -0.01 |
| WFDC2 | 0.03 | 0.08 | 0.01 | -0.03 | 0.00 | 0.05 | 0.02 | -0.04 | 0.08 | 0.05 | -0.08 | 0.05 |
| YWHAE | 0.09 | -0.01 | -0.03 | 0.00 | 0.07 | -0.04 | -0.03 | 0.04 | 0.04 | 0.07 | -0.04 | 0.00 |
| YWHAG | 0.11 | -0.01 | 0.01 | 0.03 | -0.08 | -0.02 | -0.01 | 0.06 | 0.00 | 0.01 | 0.03 | -0.01 |
| YWHAZ | 0.09 | -0.05 | 0.00 | 0.06 | -0.06 | 0.03 | 0.00 | -0.04 | -0.03 | 0.00 | 0.06 | 0.05 |

| PC13 | PC14 | PC15 | PC16 | PC17 | PC18 | PC19 | PC20 | PC21 | PC22 | PC23 | PC24 | PC25 |
|---|---|---|---|---|---|---|---|---|---|---|---|---|
| 0.01 | -0.10 | 0.05 | 0.03 | 0.05 | -0.02 | -0.02 | 0.05 | -0.03 | -0.01 | 0.00 | 0.02 | -0.09 |
| 0.04 | 0.05 | 0.00 | 0.00 | 0.03 | 0.06 | -0.03 | -0.01 | 0.00 | 0.02 | 0.00 | -0.01 | -0.03 |
| -0.10 | -0.07 | 0.00 | -0.02 | 0.01 | 0.06 | -0.03 | -0.03 | -0.04 | -0.04 | -0.05 | 0.02 | -0.01 |
| -0.03 | 0.01 | 0.09 | -0.04 | 0.11 | -0.09 | -0.11 | -0.04 | 0.01 | 0.02 | -0.09 | -0.05 | 0.10 |
| 0.00 | 0.03 | 0.03 | 0.02 | -0.02 | 0.00 | -0.07 | -0.01 | -0.01 | 0.02 | -0.02 | 0.03 | 0.06 |
| -0.02 | -0.11 | -0.03 | 0.07 | 0.03 | 0.02 | 0.00 | 0.07 | -0.01 | -0.03 | -0.03 | -0.03 | -0.01 |
| 0.00 | 0.03 | 0.01 | 0.03 | 0.05 | -0.04 | 0.04 | 0.01 | 0.04 | -0.08 | -0.04 | -0.04 | 0.03 |
| -0.02 | 0.01 | 0.00 | 0.06 | -0.02 | -0.02 | 0.02 | -0.06 | 0.01 | 0.05 | -0.05 | -0.04 | 0.01 |
| 0.00 | 0.02 | 0.03 | -0.03 | -0.01 | 0.03 | 0.02 | 0.01 | 0.01 | -0.02 | -0.01 | 0.00 | -0.03 |
| 0.02 | 0.02 | 0.06 | 0.05 | 0.08 | 0.01 | 0.08 | -0.01 | -0.05 | 0.02 | -0.04 | -0.01 | -0.07 |
| 0.07 | -0.06 | 0.04 | -0.01 | 0.11 | -0.04 | -0.01 | 0.06 | 0.00 | 0.10 | 0.05 | -0.02 | -0.01 |
| 0.03 | 0.02 | 0.01 | 0.04 | 0.01 | -0.01 | 0.00 | 0.06 | 0.03 | 0.03 | -0.05 | -0.01 | -0.04 |
| 0.03 | 0.03 | -0.03 | -0.02 | -0.04 | -0.09 | 0.01 | 0.01 | -0.05 | -0.08 | 0.04 | 0.02 | 0.07 |
| -0.03 | 0.09 | 0.06 | 0.10 | 0.02 | -0.06 | 0.02 | -0.07 | -0.03 | -0.04 | 0.12 | -0.05 | -0.05 |
| 0.02 | -0.08 | 0.01 | 0.02 | -0.03 | 0.01 | 0.02 | -0.01 | 0.00 | 0.01 | 0.02 | 0.01 | 0.03 |
| -0.03 | -0.08 | -0.02 | -0.03 | -0.07 | 0.01 | -0.02 | -0.02 | 0.10 | 0.03 | -0.03 | -0.03 | 0.02 |
| -0.05 | 0.01 | -0.01 | -0.01 | -0.04 | -0.07 | 0.00 | -0.04 | 0.09 | 0.00 | -0.02 | 0.04 | 0.04 |
| -0.04 | -0.02 | -0.01 | 0.01 | 0.10 | -0.05 | -0.02 | -0.03 | -0.03 | 0.00 | -0.01 | 0.09 | 0.08 |
| -0.07 | 0.05 | -0.01 | -0.01 | 0.05 | 0.02 | 0.04 | -0.01 | -0.05 | 0.04 | 0.02 | 0.03 | -0.02 |
| 0.12 | -0.02 | -0.02 | -0.05 | 0.02 | 0.02 | -0.06 | -0.09 | 0.06 | 0.00 | 0.00 | 0.06 | -0.08 |
| 0.06 | 0.09 | -0.10 | -0.05 | -0.05 | -0.02 | 0.00 | -0.05 | 0.03 | -0.01 | 0.02 | 0.00 | 0.06 |
| -0.02 | 0.01 | -0.02 | -0.01 | -0.02 | -0.04 | -0.03 | 0.02 | -0.05 | -0.04 | 0.00 | 0.02 | -0.01 |
| -0.04 | -0.05 | 0.06 | 0.03 | 0.02 | 0.08 | 0.06 | -0.02 | 0.05 | 0.03 | -0.02 | -0.04 | -0.05 |
| -0.02 | 0.03 | 0.02 | -0.01 | -0.01 | 0.02 | -0.09 | 0.08 | 0.05 | -0.01 | -0.02 | 0.07 | -0.01 |
| -0.01 | -0.05 | 0.01 | 0.01 | 0.01 | 0.01 | 0.05 | 0.00 | 0.01 | -0.06 | 0.14 | 0.02 | -0.07 |
| 0.03 | -0.07 | -0.02 | 0.06 | -0.10 | 0.01 | 0.09 | 0.09 | 0.02 | 0.00 | 0.05 | -0.03 | 0.01 |
| -0.02 | 0.06 | 0.03 | -0.09 | 0.03 | -0.05 | -0.06 | 0.01 | -0.11 | -0.04 | 0.05 | 0.01 | 0.00 |
| -0.01 | -0.01 | -0.03 | 0.00 | 0.03 | 0.04 | -0.04 | 0.01 | -0.02 | -0.02 | -0.02 | 0.00 | -0.03 |
| 0.02 | 0.03 | -0.03 | 0.01 | 0.00 | -0.08 | -0.01 | 0.02 | 0.07 | -0.01 | 0.03 | 0.02 | -0.02 |

| | | | | | | | | | | | | |
|---|---|---|---|---|---|---|---|---|---|---|---|---|
| -0.09 | -0.04 | 0.01 | -0.06 | -0.05 | -0.02 | -0.05 | 0.05 | 0.00 | 0.04 | -0.06 | 0.00 | -0.01 |
| 0.01 | 0.07 | 0.02 | 0.05 | 0.01 | 0.00 | -0.11 | 0.00 | 0.00 | -0.04 | 0.01 | -0.01 | -0.05 |
| 0.01 | -0.02 | 0.01 | 0.01 | 0.00 | 0.03 | 0.10 | -0.04 | -0.05 | 0.02 | 0.13 | 0.02 | 0.14 |
| 0.00 | -0.01 | -0.05 | -0.06 | 0.02 | -0.04 | -0.08 | 0.08 | 0.08 | 0.03 | 0.04 | -0.14 | -0.02 |
| -0.06 | 0.01 | -0.06 | 0.02 | 0.13 | -0.04 | 0.09 | -0.03 | 0.10 | -0.01 | -0.07 | 0.01 | -0.03 |
| 0.00 | 0.02 | -0.03 | 0.00 | 0.03 | 0.06 | -0.03 | 0.05 | 0.04 | 0.03 | 0.08 | 0.02 | -0.02 |
| 0.05 | -0.05 | 0.03 | 0.11 | -0.17 | -0.02 | 0.04 | 0.00 | 0.05 | -0.10 | -0.04 | -0.01 | -0.03 |
| 0.07 | 0.00 | 0.01 | -0.05 | -0.03 | -0.04 | -0.09 | 0.02 | 0.03 | 0.06 | -0.07 | -0.01 | 0.00 |
| 0.02 | 0.00 | -0.04 | -0.03 | -0.03 | 0.01 | 0.02 | -0.03 | 0.04 | -0.02 | 0.09 | 0.01 | -0.05 |
| 0.07 | 0.02 | 0.09 | 0.10 | 0.14 | -0.04 | -0.01 | 0.10 | 0.02 | -0.05 | -0.02 | 0.04 | -0.09 |
| -0.03 | -0.03 | 0.00 | 0.04 | -0.02 | -0.01 | -0.04 | 0.00 | 0.07 | 0.03 | -0.01 | 0.05 | 0.03 |
| 0.01 | -0.02 | -0.04 | -0.02 | -0.01 | 0.01 | -0.02 | -0.04 | 0.00 | -0.02 | -0.03 | 0.03 | 0.01 |
| 0.00 | -0.06 | -0.02 | 0.04 | 0.02 | 0.01 | -0.05 | -0.03 | 0.00 | -0.02 | -0.01 | -0.02 | -0.01 |
| 0.01 | 0.03 | -0.01 | 0.01 | -0.04 | -0.08 | -0.02 | 0.03 | -0.03 | -0.02 | 0.04 | -0.02 | 0.04 |
| 0.00 | 0.11 | 0.01 | 0.02 | -0.07 | 0.00 | -0.10 | 0.04 | 0.10 | 0.00 | -0.04 | 0.07 | -0.05 |
| 0.01 | 0.10 | -0.02 | -0.03 | -0.05 | 0.00 | -0.04 | -0.11 | 0.04 | 0.07 | -0.02 | -0.02 | -0.04 |
| 0.01 | -0.01 | -0.04 | 0.05 | -0.01 | -0.07 | 0.03 | 0.01 | 0.10 | 0.08 | 0.00 | 0.01 | 0.00 |
| 0.01 | 0.04 | -0.01 | -0.01 | -0.02 | 0.02 | -0.06 | -0.04 | 0.04 | 0.01 | 0.02 | 0.01 | -0.02 |
| 0.01 | 0.03 | 0.04 | 0.01 | 0.12 | 0.01 | 0.03 | -0.06 | 0.05 | 0.00 | 0.03 | -0.06 | 0.01 |
| 0.02 | -0.02 | 0.03 | 0.03 | 0.13 | 0.00 | -0.08 | 0.00 | -0.06 | -0.02 | 0.06 | -0.04 | -0.01 |
| 0.01 | -0.01 | -0.04 | -0.03 | 0.09 | 0.01 | 0.00 | 0.06 | -0.06 | 0.00 | -0.22 | 0.01 | -0.02 |
| -0.04 | 0.00 | -0.05 | 0.02 | 0.08 | 0.00 | -0.03 | 0.01 | 0.01 | -0.04 | -0.06 | 0.05 | -0.03 |
| -0.03 | -0.01 | 0.08 | 0.02 | -0.08 | -0.01 | 0.03 | -0.08 | 0.01 | 0.03 | 0.00 | 0.02 | 0.00 |
| -0.01 | 0.04 | 0.02 | -0.08 | -0.04 | -0.07 | 0.00 | 0.02 | 0.02 | -0.01 | 0.04 | 0.01 | 0.02 |
| 0.04 | -0.02 | 0.00 | -0.08 | 0.02 | -0.01 | 0.02 | -0.01 | -0.03 | 0.06 | -0.11 | 0.06 | 0.02 |
| -0.01 | -0.02 | 0.05 | -0.04 | -0.01 | -0.01 | -0.01 | 0.01 | 0.00 | 0.00 | -0.01 | 0.03 | -0.03 |
| -0.03 | -0.03 | 0.01 | 0.03 | 0.02 | 0.03 | 0.01 | 0.09 | 0.05 | 0.04 | 0.02 | -0.05 | 0.07 |
| -0.02 | 0.03 | 0.05 | -0.04 | 0.05 | 0.03 | -0.04 | 0.08 | 0.07 | 0.09 | 0.03 | 0.04 | 0.02 |
| 0.09 | 0.01 | -0.01 | 0.01 | 0.00 | -0.04 | 0.01 | 0.02 | -0.01 | 0.10 | 0.00 | -0.04 | 0.02 |
| -0.03 | 0.06 | -0.05 | 0.06 | -0.03 | 0.04 | 0.11 | 0.03 | 0.03 | -0.04 | 0.04 | -0.05 | 0.06 |
| 0.04 | 0.06 | 0.04 | 0.03 | 0.01 | 0.01 | -0.05 | 0.00 | 0.02 | -0.07 | -0.09 | -0.04 | 0.06 |

| | | | | | | | | | | | | |
|---|---|---|---|---|---|---|---|---|---|---|---|---|
| -0.03 | 0.01 | -0.02 | 0.05 | 0.08 | 0.06 | 0.12 | 0.07 | 0.04 | 0.10 | -0.06 | 0.00 | 0.05 |
| -0.02 | 0.06 | 0.07 | -0.01 | -0.06 | 0.10 | -0.05 | 0.01 | -0.03 | 0.02 | 0.03 | 0.07 | 0.04 |
| -0.02 | 0.02 | -0.05 | -0.06 | 0.00 | -0.01 | -0.03 | 0.00 | -0.02 | -0.07 | 0.05 | 0.03 | -0.08 |
| 0.02 | -0.06 | -0.04 | -0.06 | -0.05 | -0.06 | -0.01 | 0.11 | -0.07 | 0.03 | 0.06 | 0.03 | 0.05 |
| 0.02 | 0.00 | -0.13 | -0.07 | -0.14 | 0.03 | -0.02 | 0.04 | -0.08 | 0.02 | 0.00 | 0.11 | 0.03 |
| 0.04 | 0.02 | -0.04 | 0.03 | -0.01 | 0.01 | -0.02 | 0.01 | 0.04 | 0.02 | 0.01 | 0.02 | -0.07 |
| -0.01 | -0.04 | 0.01 | -0.01 | -0.03 | -0.02 | -0.12 | -0.01 | -0.02 | -0.09 | 0.03 | 0.02 | 0.03 |
| -0.03 | 0.03 | -0.08 | 0.01 | -0.03 | -0.02 | -0.01 | 0.05 | -0.06 | 0.10 | 0.04 | 0.02 | 0.00 |
| -0.03 | 0.02 | -0.06 | -0.05 | 0.01 | -0.01 | -0.11 | -0.04 | 0.05 | 0.04 | 0.01 | 0.02 | -0.04 |
| 0.05 | 0.01 | 0.08 | 0.00 | -0.11 | 0.01 | 0.05 | 0.10 | 0.10 | -0.11 | -0.01 | 0.12 | 0.01 |
| 0.07 | 0.00 | 0.09 | 0.04 | -0.03 | 0.00 | 0.02 | 0.01 | 0.03 | -0.01 | -0.02 | 0.00 | -0.01 |
| 0.08 | 0.07 | -0.03 | -0.04 | 0.05 | -0.03 | -0.02 | 0.07 | 0.02 | -0.04 | -0.01 | 0.01 | -0.02 |
| -0.05 | -0.01 | -0.03 | -0.01 | -0.03 | -0.02 | -0.01 | -0.01 | 0.02 | 0.04 | 0.04 | -0.04 | -0.08 |
| -0.01 | 0.00 | -0.02 | 0.06 | 0.05 | -0.09 | -0.09 | -0.09 | -0.07 | 0.07 | -0.01 | 0.07 | 0.00 |
| 0.01 | 0.02 | 0.07 | 0.06 | -0.02 | -0.02 | -0.04 | 0.04 | 0.03 | -0.02 | 0.01 | -0.07 | 0.03 |
| -0.03 | 0.02 | -0.05 | 0.07 | 0.05 | 0.04 | 0.05 | 0.04 | -0.02 | -0.03 | 0.06 | -0.03 | -0.02 |
| -0.02 | -0.03 | 0.04 | 0.06 | 0.02 | 0.03 | 0.00 | -0.05 | -0.01 | 0.07 | 0.04 | -0.02 | -0.06 |
| -0.04 | -0.02 | 0.00 | 0.00 | 0.02 | 0.06 | 0.03 | 0.01 | -0.05 | 0.00 | -0.02 | 0.03 | -0.01 |
| -0.01 | -0.01 | 0.01 | -0.03 | 0.05 | 0.04 | 0.05 | -0.04 | 0.03 | 0.11 | 0.07 | -0.02 | 0.03 |
| -0.02 | -0.01 | 0.02 | 0.01 | -0.06 | 0.04 | -0.07 | -0.06 | -0.04 | -0.03 | 0.01 | 0.04 | 0.01 |
| -0.01 | 0.03 | -0.02 | -0.01 | 0.00 | 0.00 | 0.00 | 0.00 | 0.01 | -0.09 | 0.00 | -0.01 | -0.02 |
| -0.01 | -0.04 | 0.08 | 0.07 | -0.05 | 0.13 | 0.00 | 0.04 | 0.05 | 0.09 | -0.02 | 0.06 | 0.06 |
| 0.01 | 0.00 | -0.07 | -0.08 | 0.01 | -0.04 | 0.06 | -0.01 | -0.03 | -0.07 | -0.02 | -0.03 | 0.04 |
| 0.01 | 0.00 | -0.02 | -0.01 | -0.07 | -0.06 | 0.04 | 0.00 | -0.03 | 0.08 | 0.13 | -0.08 | 0.03 |
| 0.03 | -0.01 | -0.07 | 0.01 | -0.10 | -0.01 | 0.08 | 0.11 | 0.07 | -0.02 | 0.08 | -0.09 | -0.03 |
| -0.10 | -0.04 | -0.05 | 0.04 | -0.05 | -0.03 | 0.09 | 0.02 | -0.06 | -0.01 | 0.00 | 0.00 | -0.02 |
| -0.07 | 0.05 | -0.05 | 0.01 | 0.04 | -0.02 | 0.08 | 0.03 | -0.05 | 0.03 | 0.00 | 0.00 | 0.03 |
| 0.04 | -0.05 | -0.05 | 0.01 | -0.01 | 0.06 | -0.04 | -0.05 | -0.02 | -0.05 | 0.01 | 0.00 | -0.01 |
| 0.02 | -0.02 | -0.01 | 0.03 | -0.02 | -0.05 | -0.01 | -0.07 | -0.05 | 0.04 | -0.01 | -0.03 | 0.00 |
| 0.05 | 0.00 | 0.04 | 0.00 | 0.10 | 0.01 | -0.04 | 0.04 | -0.02 | 0.00 | -0.01 | -0.02 | 0.05 |
| 0.00 | 0.01 | 0.00 | 0.00 | -0.02 | 0.02 | -0.01 | -0.03 | 0.06 | 0.05 | 0.00 | 0.00 | 0.02 |

| | | | | | | | | | | | | |
|---|---|---|---|---|---|---|---|---|---|---|---|---|
| -0.01 | 0.05 | -0.01 | 0.01 | 0.03 | 0.06 | -0.03 | 0.04 | 0.04 | -0.04 | 0.02 | 0.02 | 0.00 |
| 0.04 | -0.08 | 0.04 | 0.06 | 0.01 | 0.00 | 0.06 | 0.02 | 0.08 | 0.02 | 0.02 | -0.04 | 0.05 |
| -0.02 | -0.01 | -0.04 | -0.03 | -0.03 | 0.02 | -0.01 | 0.00 | -0.01 | 0.00 | 0.04 | -0.05 | 0.06 |
| -0.02 | -0.07 | -0.02 | 0.02 | -0.01 | -0.06 | -0.06 | 0.03 | -0.04 | -0.04 | -0.06 | -0.03 | 0.01 |
| -0.07 | 0.05 | 0.09 | -0.04 | -0.01 | -0.06 | -0.01 | 0.03 | 0.06 | 0.05 | -0.01 | -0.02 | -0.01 |
| 0.01 | -0.01 | 0.05 | -0.01 | 0.02 | 0.11 | -0.05 | 0.00 | -0.04 | 0.02 | 0.02 | 0.01 | -0.05 |
| -0.03 | -0.05 | 0.04 | 0.06 | -0.01 | 0.05 | 0.06 | -0.08 | -0.02 | -0.03 | 0.09 | -0.09 | 0.04 |
| -0.03 | -0.04 | 0.02 | -0.05 | -0.04 | 0.01 | 0.04 | -0.06 | 0.07 | 0.00 | -0.02 | 0.04 | 0.07 |
| -0.04 | 0.03 | -0.05 | -0.04 | 0.03 | -0.03 | -0.05 | 0.01 | -0.01 | -0.07 | 0.03 | -0.06 | 0.01 |
| -0.10 | 0.00 | -0.02 | 0.06 | 0.13 | -0.12 | -0.02 | 0.05 | -0.02 | -0.05 | 0.09 | 0.02 | 0.01 |
| -0.05 | -0.02 | -0.05 | 0.03 | 0.02 | 0.05 | 0.04 | 0.08 | 0.01 | -0.09 | 0.05 | 0.01 | -0.01 |
| -0.02 | 0.03 | -0.09 | -0.01 | -0.08 | -0.05 | 0.00 | -0.01 | -0.02 | 0.02 | 0.08 | -0.04 | 0.03 |
| -0.02 | 0.02 | -0.10 | -0.04 | -0.08 | -0.06 | -0.05 | -0.01 | 0.00 | 0.01 | 0.05 | -0.04 | 0.00 |
| -0.11 | 0.02 | 0.01 | -0.05 | -0.08 | -0.08 | 0.10 | 0.02 | 0.01 | 0.05 | 0.06 | -0.02 | -0.05 |
| 0.02 | -0.06 | 0.01 | 0.02 | 0.01 | -0.05 | -0.06 | 0.05 | -0.01 | -0.03 | 0.06 | 0.06 | 0.07 |
| -0.01 | -0.03 | -0.04 | 0.03 | 0.07 | 0.02 | 0.02 | -0.01 | 0.05 | 0.01 | 0.01 | 0.06 | -0.05 |
| 0.02 | -0.05 | -0.02 | -0.08 | 0.01 | -0.02 | -0.03 | 0.08 | 0.01 | 0.00 | 0.04 | -0.03 | -0.03 |
| -0.02 | -0.01 | 0.02 | 0.06 | -0.03 | 0.02 | -0.05 | -0.04 | -0.11 | 0.00 | 0.07 | 0.02 | 0.00 |
| -0.08 | -0.04 | 0.00 | 0.00 | 0.10 | 0.05 | -0.01 | -0.05 | 0.09 | -0.03 | 0.11 | -0.02 | -0.05 |
| -0.04 | -0.02 | 0.05 | 0.03 | -0.04 | -0.02 | -0.04 | -0.02 | 0.04 | -0.02 | -0.01 | 0.02 | 0.03 |
| -0.03 | -0.07 | 0.11 | 0.01 | -0.03 | 0.03 | -0.07 | 0.01 | -0.02 | 0.03 | 0.02 | 0.00 | 0.04 |
| -0.03 | 0.03 | 0.02 | 0.00 | 0.01 | 0.01 | -0.04 | -0.02 | 0.00 | 0.01 | 0.03 | -0.03 | 0.06 |
| 0.01 | 0.02 | 0.00 | 0.02 | -0.01 | -0.08 | -0.06 | -0.03 | -0.02 | -0.08 | -0.09 | 0.01 | -0.03 |
| 0.04 | 0.03 | 0.01 | 0.07 | 0.04 | -0.01 | 0.10 | 0.07 | -0.04 | 0.04 | 0.02 | -0.04 | 0.01 |
| -0.11 | -0.06 | -0.04 | -0.07 | -0.03 | 0.05 | 0.02 | 0.03 | -0.04 | -0.01 | 0.02 | -0.01 | -0.09 |
| -0.07 | -0.07 | 0.02 | 0.12 | -0.03 | -0.03 | 0.03 | -0.07 | 0.05 | -0.04 | 0.01 | -0.02 | 0.03 |
| -0.02 | -0.03 | 0.03 | -0.08 | -0.05 | -0.04 | 0.09 | -0.04 | -0.06 | -0.06 | 0.00 | -0.05 | 0.02 |
| 0.02 | 0.05 | 0.04 | -0.05 | 0.03 | 0.05 | 0.03 | -0.01 | -0.03 | 0.01 | 0.03 | -0.05 | -0.01 |
| 0.06 | -0.01 | -0.04 | -0.01 | 0.00 | -0.04 | -0.08 | 0.00 | 0.01 | 0.10 | 0.06 | 0.12 | -0.01 |
| -0.07 | 0.02 | -0.02 | 0.06 | 0.01 | -0.06 | -0.04 | -0.13 | 0.12 | 0.02 | -0.12 | 0.02 | 0.13 |
| 0.01 | -0.01 | 0.04 | -0.06 | 0.05 | -0.03 | -0.02 | -0.01 | 0.00 | -0.05 | 0.01 | -0.06 | 0.02 |

| | | | | | | | | | | | | |
|---|---|---|---|---|---|---|---|---|---|---|---|---|
| 0.02 | -0.03 | 0.00 | 0.01 | 0.00 | -0.01 | 0.05 | -0.05 | -0.04 | 0.05 | -0.02 | -0.02 | 0.05 |
| 0.03 | 0.08 | 0.01 | 0.04 | 0.02 | -0.07 | -0.10 | 0.01 | -0.03 | 0.08 | 0.03 | 0.04 | -0.12 |
| 0.03 | 0.02 | -0.01 | 0.00 | 0.00 | 0.00 | -0.10 | -0.10 | 0.03 | -0.03 | -0.02 | -0.03 | -0.03 |
| 0.01 | 0.07 | 0.00 | 0.05 | -0.01 | 0.01 | -0.09 | -0.01 | 0.01 | -0.02 | -0.04 | 0.02 | -0.01 |
| 0.07 | 0.04 | -0.04 | 0.04 | 0.06 | -0.01 | -0.05 | -0.09 | -0.01 | 0.10 | -0.03 | 0.05 | -0.12 |
| -0.02 | 0.05 | 0.00 | 0.08 | -0.05 | 0.00 | -0.09 | 0.04 | -0.01 | -0.07 | -0.03 | -0.01 | -0.07 |
| -0.03 | 0.03 | -0.01 | 0.09 | -0.06 | 0.09 | -0.04 | 0.05 | -0.03 | -0.01 | -0.04 | -0.06 | 0.01 |
| -0.01 | -0.06 | -0.03 | -0.04 | 0.02 | 0.03 | -0.01 | -0.01 | 0.03 | -0.06 | -0.04 | 0.03 | 0.02 |
| 0.00 | -0.05 | -0.03 | 0.02 | -0.10 | 0.04 | 0.04 | 0.02 | 0.07 | 0.02 | -0.07 | 0.03 | -0.02 |
| -0.05 | 0.00 | -0.05 | -0.02 | 0.01 | 0.01 | 0.02 | -0.01 | 0.02 | 0.02 | 0.02 | -0.02 | -0.01 |
| -0.04 | -0.11 | -0.09 | 0.02 | -0.07 | 0.00 | -0.03 | 0.01 | -0.04 | 0.00 | 0.00 | -0.03 | -0.05 |
| -0.07 | -0.04 | -0.04 | 0.04 | -0.01 | 0.06 | -0.04 | -0.02 | -0.03 | -0.01 | 0.03 | -0.10 | -0.04 |
| -0.01 | 0.00 | -0.09 | -0.01 | -0.04 | -0.03 | -0.07 | 0.03 | 0.00 | 0.05 | -0.02 | -0.03 | 0.03 |
| -0.02 | -0.06 | 0.01 | -0.01 | -0.04 | 0.02 | -0.08 | 0.05 | 0.09 | 0.07 | -0.01 | -0.17 | 0.01 |
| -0.04 | -0.01 | 0.02 | 0.05 | 0.08 | -0.04 | -0.01 | -0.02 | 0.00 | -0.06 | -0.07 | 0.04 | 0.04 |
| -0.04 | 0.06 | 0.02 | 0.01 | -0.06 | -0.02 | 0.02 | -0.05 | 0.03 | 0.07 | -0.03 | 0.02 | 0.03 |
| -0.03 | -0.03 | -0.07 | -0.03 | 0.07 | -0.03 | -0.03 | -0.08 | -0.03 | 0.04 | 0.07 | 0.04 | 0.04 |
| 0.05 | 0.01 | 0.07 | -0.01 | -0.06 | -0.01 | -0.02 | 0.15 | -0.02 | -0.04 | -0.03 | 0.03 | -0.05 |
| -0.01 | 0.00 | 0.01 | 0.01 | 0.03 | -0.02 | -0.02 | -0.05 | -0.02 | -0.11 | -0.01 | -0.04 | 0.00 |
| 0.04 | -0.01 | 0.10 | 0.07 | -0.04 | 0.01 | 0.06 | 0.14 | -0.01 | 0.06 | 0.02 | -0.01 | 0.01 |
| 0.00 | 0.00 | 0.04 | 0.01 | -0.04 | 0.01 | 0.03 | -0.08 | 0.04 | -0.05 | 0.02 | -0.07 | 0.03 |
| 0.06 | -0.03 | 0.03 | -0.02 | 0.04 | 0.03 | -0.02 | 0.02 | 0.00 | 0.02 | 0.03 | 0.04 | 0.03 |
| -0.09 | -0.01 | -0.01 | 0.04 | -0.01 | -0.01 | 0.02 | 0.14 | 0.03 | -0.02 | 0.06 | 0.13 | -0.02 |
| 0.05 | -0.02 | 0.07 | 0.03 | -0.03 | 0.00 | 0.01 | 0.00 | 0.02 | -0.02 | -0.02 | 0.00 | -0.02 |
| -0.06 | -0.08 | -0.05 | 0.01 | -0.02 | -0.05 | -0.05 | 0.03 | -0.02 | 0.03 | 0.04 | -0.01 | -0.05 |
| -0.05 | 0.04 | 0.06 | 0.00 | 0.01 | 0.04 | -0.09 | 0.08 | 0.02 | -0.06 | -0.01 | -0.05 | -0.05 |
| 0.09 | 0.06 | 0.05 | 0.03 | 0.11 | -0.05 | 0.04 | -0.08 | -0.04 | 0.05 | -0.07 | 0.01 | -0.17 |
| -0.03 | 0.00 | 0.00 | 0.04 | 0.03 | -0.04 | 0.04 | 0.01 | 0.02 | 0.04 | -0.06 | 0.06 | -0.14 |
| 0.02 | 0.01 | 0.01 | 0.01 | 0.01 | -0.08 | -0.05 | 0.04 | 0.04 | -0.01 | 0.03 | 0.00 | -0.02 |
| 0.03 | -0.04 | -0.10 | -0.02 | 0.12 | -0.07 | 0.04 | 0.06 | 0.02 | -0.02 | -0.05 | -0.03 | 0.03 |
| 0.04 | -0.05 | 0.05 | 0.01 | 0.02 | -0.03 | 0.01 | 0.00 | -0.03 | 0.03 | 0.04 | 0.02 | 0.01 |

| | | | | | | | | | | | | |
|---|---|---|---|---|---|---|---|---|---|---|---|---|
| 0.02 | -0.03 | 0.07 | -0.01 | -0.07 | -0.01 | 0.05 | -0.04 | 0.05 | -0.09 | 0.02 | -0.01 | 0.06 |
| -0.05 | 0.04 | 0.03 | 0.02 | 0.08 | 0.06 | -0.05 | 0.08 | 0.02 | 0.05 | 0.05 | 0.01 | 0.00 |
| -0.07 | 0.05 | 0.02 | -0.01 | 0.03 | -0.01 | -0.04 | -0.09 | 0.06 | -0.12 | -0.06 | -0.02 | 0.00 |
| 0.03 | -0.03 | -0.13 | -0.10 | 0.03 | 0.03 | 0.07 | 0.06 | 0.00 | 0.09 | -0.04 | -0.03 | -0.04 |
| 0.03 | -0.01 | 0.02 | -0.02 | -0.05 | 0.00 | 0.00 | 0.01 | 0.00 | 0.04 | 0.02 | 0.05 | -0.05 |
| -0.02 | 0.05 | -0.02 | 0.00 | 0.08 | 0.03 | 0.00 | 0.08 | 0.02 | 0.07 | -0.06 | 0.08 | -0.01 |
| 0.06 | -0.01 | 0.06 | 0.00 | 0.00 | -0.02 | 0.03 | 0.00 | 0.02 | -0.01 | 0.07 | -0.01 | 0.01 |
| 0.03 | 0.00 | 0.04 | -0.01 | -0.02 | 0.03 | 0.01 | -0.02 | -0.02 | 0.01 | 0.00 | -0.01 | 0.01 |
| 0.10 | -0.02 | -0.02 | -0.07 | 0.00 | 0.01 | 0.05 | -0.03 | 0.04 | 0.06 | -0.07 | -0.09 | 0.07 |
| -0.01 | 0.03 | -0.06 | 0.02 | 0.04 | 0.00 | -0.01 | 0.03 | 0.01 | -0.02 | -0.02 | -0.01 | 0.02 |
| -0.13 | -0.03 | 0.01 | 0.07 | 0.07 | 0.04 | 0.01 | 0.04 | -0.09 | 0.05 | 0.12 | 0.03 | -0.07 |
| -0.01 | 0.00 | 0.03 | 0.04 | -0.04 | -0.03 | -0.05 | -0.04 | -0.04 | 0.00 | 0.00 | -0.01 | 0.02 |
| -0.07 | 0.00 | -0.02 | -0.05 | 0.00 | 0.02 | 0.09 | -0.03 | 0.02 | -0.03 | 0.03 | 0.07 | -0.07 |
| -0.01 | 0.08 | -0.01 | -0.06 | 0.01 | -0.06 | 0.05 | -0.03 | 0.01 | -0.08 | -0.01 | -0.01 | 0.00 |
| 0.03 | 0.01 | -0.07 | -0.07 | 0.04 | -0.09 | 0.12 | 0.08 | 0.01 | -0.07 | 0.07 | 0.08 | -0.10 |
| -0.01 | -0.02 | 0.00 | 0.02 | -0.08 | -0.01 | 0.00 | -0.04 | -0.02 | 0.00 | -0.01 | -0.08 | -0.01 |
| 0.05 | 0.06 | 0.03 | -0.11 | 0.06 | -0.05 | 0.01 | 0.07 | -0.08 | -0.10 | -0.08 | -0.02 | -0.02 |
| -0.07 | -0.04 | 0.05 | -0.10 | -0.02 | -0.04 | 0.10 | -0.03 | -0.01 | -0.01 | -0.04 | -0.03 | -0.04 |
| 0.06 | 0.00 | 0.03 | 0.11 | -0.07 | -0.04 | 0.06 | 0.01 | 0.11 | -0.10 | -0.02 | -0.02 | -0.04 |
| 0.01 | -0.02 | -0.06 | 0.00 | -0.07 | -0.03 | -0.02 | -0.01 | 0.06 | -0.01 | -0.06 | 0.10 | 0.01 |
| -0.08 | -0.08 | -0.04 | -0.01 | 0.05 | -0.08 | 0.01 | -0.03 | 0.03 | 0.02 | -0.01 | 0.03 | 0.00 |
| -0.07 | -0.04 | -0.07 | -0.04 | -0.01 | 0.06 | 0.05 | -0.05 | 0.07 | 0.08 | -0.03 | 0.03 | -0.01 |
| 0.02 | 0.10 | 0.03 | -0.01 | -0.01 | -0.01 | 0.06 | -0.01 | -0.09 | -0.05 | -0.01 | -0.01 | 0.03 |
| 0.06 | -0.03 | 0.03 | -0.05 | 0.01 | 0.02 | -0.01 | -0.03 | -0.04 | 0.06 | -0.02 | 0.04 | 0.03 |
| -0.04 | 0.02 | 0.02 | -0.01 | -0.02 | 0.04 | -0.01 | 0.01 | 0.01 | -0.13 | 0.00 | -0.08 | 0.07 |
| 0.02 | -0.07 | 0.01 | 0.02 | -0.02 | -0.01 | 0.00 | 0.01 | 0.00 | -0.01 | 0.00 | -0.03 | -0.01 |
| -0.05 | -0.03 | -0.06 | 0.01 | 0.03 | 0.00 | 0.05 | -0.02 | 0.07 | 0.02 | 0.02 | 0.00 | 0.01 |
| -0.03 | -0.01 | 0.00 | -0.01 | 0.05 | -0.04 | 0.03 | 0.00 | -0.02 | 0.00 | -0.04 | -0.04 | 0.00 |
| 0.02 | -0.10 | 0.04 | -0.01 | 0.02 | 0.02 | 0.00 | 0.02 | 0.03 | 0.00 | 0.04 | 0.10 | 0.00 |
| -0.03 | -0.02 | -0.03 | 0.05 | 0.02 | 0.02 | -0.04 | 0.03 | 0.05 | -0.04 | 0.01 | -0.02 | 0.01 |
| 0.04 | -0.03 | -0.03 | -0.05 | 0.07 | 0.08 | -0.02 | -0.04 | 0.00 | 0.00 | -0.06 | -0.01 | 0.01 |

| | | | | | | | | | | | | |
|---|---|---|---|---|---|---|---|---|---|---|---|---|
| -0.02 | 0.00 | 0.02 | -0.04 | -0.04 | -0.01 | -0.03 | -0.02 | 0.02 | -0.01 | 0.00 | 0.00 | -0.02 |
| -0.06 | 0.02 | 0.05 | 0.02 | 0.02 | 0.10 | 0.01 | -0.06 | 0.02 | 0.00 | 0.10 | -0.07 | 0.08 |
| 0.01 | -0.09 | -0.03 | -0.09 | 0.01 | 0.06 | 0.02 | -0.05 | -0.05 | -0.04 | 0.01 | -0.04 | -0.02 |
| -0.01 | -0.02 | -0.09 | 0.04 | 0.08 | 0.01 | 0.02 | -0.03 | -0.04 | 0.03 | 0.04 | 0.05 | 0.01 |
| 0.02 | -0.02 | 0.07 | 0.01 | -0.02 | -0.05 | 0.03 | 0.01 | -0.06 | -0.05 | -0.04 | 0.05 | -0.04 |
| -0.01 | -0.05 | 0.10 | 0.00 | 0.03 | 0.00 | 0.04 | -0.05 | -0.01 | 0.06 | -0.07 | -0.04 | -0.01 |
| 0.00 | 0.00 | -0.03 | 0.07 | -0.02 | 0.07 | 0.00 | 0.01 | 0.06 | 0.05 | -0.02 | 0.01 | -0.03 |
| 0.05 | -0.06 | -0.01 | -0.10 | -0.01 | 0.00 | 0.03 | -0.04 | -0.08 | -0.05 | 0.00 | -0.05 | 0.05 |
| 0.07 | 0.05 | -0.01 | -0.02 | -0.03 | 0.00 | 0.03 | 0.01 | 0.02 | 0.00 | 0.04 | 0.01 | 0.01 |
| -0.08 | 0.05 | 0.06 | -0.06 | -0.01 | 0.03 | 0.03 | 0.03 | 0.01 | 0.01 | -0.02 | 0.03 | 0.00 |
| -0.06 | 0.03 | -0.07 | 0.06 | 0.04 | -0.04 | 0.06 | -0.02 | -0.10 | 0.05 | 0.03 | -0.01 | -0.02 |
| 0.09 | -0.03 | -0.08 | 0.08 | 0.06 | 0.08 | 0.01 | -0.01 | -0.02 | -0.04 | 0.02 | -0.01 | 0.03 |
| 0.00 | 0.08 | -0.04 | 0.05 | -0.01 | 0.05 | -0.09 | 0.02 | 0.02 | -0.09 | -0.01 | 0.01 | 0.03 |
| 0.01 | -0.03 | 0.04 | -0.01 | -0.06 | 0.03 | -0.14 | 0.03 | 0.03 | -0.04 | -0.02 | 0.00 | -0.05 |
| -0.03 | 0.08 | -0.06 | 0.06 | -0.05 | 0.08 | -0.01 | 0.04 | -0.04 | -0.02 | -0.04 | 0.01 | 0.05 |
| 0.03 | 0.01 | 0.05 | 0.01 | 0.00 | -0.01 | -0.03 | -0.02 | -0.02 | -0.06 | 0.04 | 0.00 | 0.00 |
| 0.00 | -0.03 | -0.03 | 0.00 | -0.03 | -0.04 | 0.01 | 0.04 | 0.04 | 0.01 | -0.07 | -0.03 | 0.06 |
| -0.04 | 0.02 | 0.03 | 0.00 | 0.04 | 0.04 | 0.00 | 0.02 | 0.02 | 0.01 | -0.01 | -0.01 | 0.01 |
| -0.07 | 0.04 | -0.01 | 0.03 | 0.01 | -0.02 | 0.00 | 0.00 | 0.01 | 0.00 | -0.02 | -0.07 | 0.05 |
| 0.01 | -0.09 | -0.02 | 0.00 | -0.02 | 0.05 | 0.08 | 0.02 | 0.01 | 0.04 | -0.08 | -0.04 | 0.00 |
| 0.01 | 0.02 | 0.08 | 0.04 | -0.02 | 0.04 | 0.08 | 0.09 | 0.00 | -0.03 | 0.08 | -0.03 | 0.06 |
| 0.01 | -0.03 | 0.01 | 0.06 | -0.02 | -0.08 | -0.03 | -0.04 | -0.05 | -0.01 | -0.01 | -0.05 | 0.00 |
| -0.05 | 0.02 | -0.05 | 0.07 | -0.03 | 0.01 | -0.03 | 0.00 | -0.03 | 0.07 | 0.03 | -0.03 | -0.03 |
| -0.04 | 0.08 | 0.00 | 0.05 | -0.06 | 0.03 | 0.00 | -0.03 | -0.01 | -0.02 | 0.01 | -0.04 | 0.03 |
| -0.01 | -0.06 | 0.05 | 0.04 | 0.02 | -0.01 | 0.05 | -0.03 | -0.01 | -0.06 | -0.07 | -0.07 | -0.07 |
| 0.05 | -0.03 | 0.11 | -0.06 | 0.06 | -0.05 | 0.06 | 0.00 | 0.10 | 0.07 | 0.01 | 0.06 | 0.00 |
| -0.04 | 0.04 | 0.04 | 0.04 | 0.04 | 0.04 | -0.02 | 0.04 | -0.03 | -0.09 | 0.00 | -0.05 | -0.04 |
| -0.04 | -0.08 | -0.04 | -0.04 | -0.01 | -0.04 | -0.01 | -0.04 | 0.01 | -0.02 | 0.05 | -0.03 | 0.12 |
| -0.01 | -0.11 | -0.02 | 0.02 | -0.01 | 0.04 | -0.01 | -0.02 | -0.05 | -0.01 | -0.04 | -0.07 | 0.08 |
| -0.08 | 0.03 | 0.05 | -0.02 | -0.01 | -0.01 | -0.04 | 0.03 | 0.02 | 0.05 | 0.04 | 0.07 | -0.01 |
| -0.11 | 0.00 | 0.04 | -0.03 | 0.02 | -0.02 | 0.03 | 0.08 | 0.00 | 0.01 | -0.08 | 0.04 | 0.02 |

| | | | | | | | | | | | | |
|---|---|---|---|---|---|---|---|---|---|---|---|---|
| -0.06 | -0.02 | 0.06 | -0.02 | 0.02 | -0.10 | -0.06 | -0.02 | 0.07 | -0.02 | 0.08 | -0.13 | -0.08 |
| 0.07 | -0.11 | 0.04 | 0.03 | -0.03 | -0.03 | -0.05 | 0.09 | -0.03 | 0.02 | 0.01 | 0.00 | -0.05 |
| -0.11 | 0.00 | 0.05 | -0.01 | 0.03 | 0.01 | 0.00 | 0.02 | -0.01 | 0.01 | -0.04 | 0.01 | 0.00 |
| 0.04 | -0.04 | -0.05 | 0.03 | 0.00 | -0.11 | -0.04 | 0.07 | -0.01 | -0.01 | -0.05 | -0.12 | -0.01 |
| 0.03 | -0.01 | 0.13 | 0.05 | -0.03 | -0.03 | -0.01 | -0.03 | -0.04 | -0.11 | 0.01 | -0.06 | -0.04 |
| -0.04 | 0.01 | -0.02 | 0.10 | 0.05 | -0.06 | 0.03 | 0.04 | -0.07 | 0.09 | -0.04 | 0.06 | 0.06 |
| -0.01 | 0.03 | -0.06 | 0.07 | 0.00 | -0.07 | -0.06 | 0.00 | -0.02 | -0.10 | 0.02 | -0.03 | 0.11 |
| 0.02 | -0.03 | 0.02 | 0.04 | 0.01 | -0.03 | 0.00 | -0.08 | -0.06 | -0.03 | 0.04 | 0.01 | 0.08 |
| 0.00 | -0.12 | -0.01 | 0.04 | -0.06 | -0.08 | 0.04 | -0.05 | 0.02 | 0.05 | -0.01 | -0.02 | 0.11 |
| 0.04 | -0.02 | -0.12 | 0.08 | -0.07 | -0.03 | 0.06 | 0.02 | 0.03 | -0.04 | 0.08 | -0.10 | -0.06 |
| 0.06 | 0.01 | 0.04 | 0.05 | 0.08 | 0.04 | 0.05 | 0.09 | 0.08 | -0.06 | 0.05 | 0.04 | 0.16 |
| -0.02 | -0.03 | 0.02 | 0.07 | 0.00 | -0.07 | -0.01 | -0.07 | -0.04 | -0.03 | -0.06 | 0.02 | 0.03 |
| -0.06 | 0.04 | -0.01 | 0.11 | 0.09 | 0.10 | -0.07 | 0.00 | -0.05 | -0.02 | -0.04 | -0.02 | 0.09 |
| 0.06 | -0.10 | -0.08 | 0.07 | 0.09 | -0.05 | 0.02 | 0.11 | 0.05 | 0.01 | -0.02 | -0.09 | -0.10 |
| -0.05 | 0.03 | 0.01 | -0.03 | 0.00 | 0.03 | 0.05 | 0.02 | -0.02 | 0.00 | -0.01 | 0.04 | 0.01 |
| 0.02 | -0.04 | -0.09 | -0.03 | 0.01 | 0.06 | -0.02 | 0.06 | -0.05 | -0.02 | 0.02 | 0.01 | 0.02 |
| -0.03 | -0.07 | 0.02 | 0.09 | 0.03 | -0.01 | 0.07 | -0.10 | 0.07 | -0.06 | 0.06 | 0.19 | -0.07 |
| 0.04 | -0.02 | 0.04 | 0.07 | 0.02 | 0.00 | -0.01 | 0.01 | 0.02 | 0.00 | -0.01 | -0.02 | 0.00 |
| -0.02 | 0.03 | -0.08 | 0.05 | -0.13 | -0.08 | -0.05 | -0.08 | -0.08 | 0.05 | 0.01 | -0.01 | -0.06 |
| 0.01 | -0.02 | 0.07 | 0.04 | 0.03 | -0.05 | -0.02 | -0.02 | 0.01 | 0.02 | -0.01 | -0.02 | 0.00 |
| 0.06 | -0.06 | -0.04 | 0.06 | 0.00 | 0.12 | -0.08 | 0.05 | -0.07 | 0.04 | 0.01 | -0.01 | 0.04 |
| -0.04 | 0.03 | -0.05 | 0.03 | -0.05 | 0.01 | 0.06 | 0.00 | 0.02 | 0.04 | -0.01 | -0.04 | 0.02 |
| 0.01 | -0.10 | -0.02 | 0.11 | 0.01 | -0.08 | 0.03 | 0.05 | -0.07 | -0.02 | -0.04 | 0.08 | -0.01 |
| -0.03 | -0.06 | 0.01 | 0.04 | 0.03 | 0.00 | -0.02 | -0.05 | -0.04 | 0.09 | 0.09 | -0.01 | 0.04 |
| -0.05 | -0.06 | 0.06 | 0.00 | 0.04 | -0.07 | 0.05 | -0.06 | -0.07 | 0.06 | 0.04 | 0.01 | 0.08 |
| -0.06 | 0.07 | -0.03 | -0.02 | 0.03 | -0.15 | -0.02 | 0.07 | 0.03 | -0.03 | 0.08 | -0.03 | 0.05 |
| -0.02 | 0.02 | 0.04 | 0.07 | 0.00 | -0.07 | -0.11 | -0.04 | 0.10 | 0.02 | 0.06 | -0.04 | -0.02 |
| 0.13 | -0.01 | -0.07 | 0.02 | -0.01 | 0.05 | -0.02 | -0.04 | 0.01 | -0.04 | 0.05 | -0.02 | 0.00 |
| 0.03 | -0.02 | 0.03 | 0.05 | 0.04 | -0.02 | 0.02 | -0.01 | 0.00 | 0.06 | 0.06 | 0.03 | 0.02 |
| -0.06 | -0.02 | 0.07 | 0.05 | -0.02 | 0.03 | -0.05 | -0.08 | 0.10 | 0.01 | 0.05 | 0.00 | 0.01 |
| -0.16 | 0.01 | 0.08 | -0.08 | -0.02 | -0.01 | -0.01 | 0.01 | 0.00 | 0.01 | -0.05 | 0.03 | -0.02 |

| | | | | | | | | | | | | |
|---|---|---|---|---|---|---|---|---|---|---|---|---|
| 0.07 | 0.04 | 0.04 | -0.07 | -0.01 | -0.01 | -0.03 | 0.08 | 0.03 | -0.04 | 0.03 | 0.02 | 0.01 |
| -0.02 | -0.10 | -0.01 | 0.07 | -0.05 | -0.05 | 0.01 | 0.10 | -0.02 | 0.04 | -0.05 | 0.02 | -0.08 |
| 0.02 | -0.05 | 0.07 | -0.01 | -0.03 | 0.00 | -0.05 | -0.09 | -0.12 | -0.01 | 0.00 | 0.05 | -0.06 |
| -0.14 | -0.12 | 0.01 | 0.00 | 0.01 | 0.13 | -0.02 | 0.01 | -0.07 | 0.00 | 0.03 | 0.00 | -0.08 |
| -0.04 | -0.03 | 0.01 | 0.02 | 0.06 | 0.06 | 0.01 | 0.04 | -0.06 | 0.05 | -0.02 | -0.05 | -0.04 |
| 0.04 | 0.00 | 0.06 | 0.00 | -0.01 | 0.03 | -0.10 | 0.07 | -0.05 | 0.03 | -0.06 | 0.06 | 0.04 |
| -0.04 | -0.11 | 0.06 | 0.13 | 0.07 | -0.08 | -0.06 | 0.07 | 0.02 | -0.04 | 0.11 | 0.11 | 0.02 |
| -0.03 | 0.06 | -0.07 | 0.06 | -0.07 | 0.07 | -0.03 | 0.05 | 0.01 | 0.13 | -0.10 | -0.09 | 0.02 |
| -0.07 | -0.09 | -0.05 | 0.04 | -0.06 | -0.03 | -0.06 | -0.08 | -0.03 | -0.11 | 0.10 | 0.08 | -0.10 |
| 0.06 | 0.00 | -0.04 | -0.12 | 0.05 | 0.04 | -0.01 | 0.04 | -0.06 | 0.02 | -0.02 | 0.05 | -0.03 |
| 0.01 | 0.05 | 0.01 | 0.02 | 0.04 | 0.08 | -0.12 | 0.06 | 0.06 | -0.06 | 0.03 | -0.06 | -0.03 |
| 0.04 | 0.05 | 0.05 | -0.05 | -0.01 | 0.04 | -0.07 | 0.09 | 0.01 | -0.02 | 0.07 | 0.02 | -0.03 |
| -0.02 | 0.00 | 0.02 | -0.02 | -0.10 | -0.03 | 0.03 | 0.10 | 0.07 | -0.10 | -0.01 | 0.13 | 0.01 |
| 0.01 | 0.10 | 0.05 | -0.04 | 0.00 | -0.01 | -0.02 | 0.05 | -0.03 | -0.07 | 0.07 | 0.00 | 0.04 |
| -0.01 | -0.07 | 0.02 | -0.04 | -0.02 | 0.03 | 0.03 | -0.10 | 0.05 | -0.04 | 0.04 | -0.07 | -0.02 |
| 0.02 | 0.06 | 0.03 | 0.03 | 0.00 | 0.07 | 0.01 | -0.02 | 0.05 | 0.08 | -0.07 | 0.06 | 0.04 |
| 0.08 | 0.03 | -0.05 | 0.04 | 0.05 | -0.04 | -0.08 | 0.06 | -0.07 | 0.08 | -0.11 | 0.03 | 0.05 |
| -0.03 | 0.11 | -0.05 | -0.01 | -0.07 | 0.00 | 0.08 | 0.03 | -0.06 | 0.00 | 0.01 | -0.01 | 0.01 |
| -0.02 | 0.01 | -0.06 | 0.03 | 0.02 | 0.04 | 0.05 | -0.04 | 0.04 | 0.03 | -0.04 | -0.15 | -0.17 |
| -0.03 | -0.04 | 0.02 | 0.00 | 0.03 | -0.01 | -0.08 | -0.02 | 0.04 | 0.03 | 0.00 | -0.07 | -0.01 |
| 0.00 | 0.05 | 0.08 | 0.11 | -0.03 | 0.07 | -0.03 | -0.01 | -0.02 | 0.03 | -0.05 | 0.07 | -0.02 |
| 0.02 | 0.06 | 0.09 | 0.09 | -0.01 | 0.07 | 0.01 | 0.03 | -0.03 | -0.01 | -0.02 | 0.07 | -0.02 |
| -0.09 | 0.02 | -0.03 | 0.03 | -0.06 | 0.15 | -0.01 | 0.03 | -0.12 | 0.02 | -0.02 | 0.01 | -0.01 |
| 0.00 | 0.05 | 0.08 | 0.10 | 0.00 | 0.09 | 0.01 | 0.02 | -0.04 | -0.01 | -0.03 | 0.09 | -0.02 |
| 0.00 | 0.02 | -0.06 | -0.04 | 0.07 | -0.04 | -0.01 | -0.05 | -0.02 | -0.02 | -0.07 | 0.04 | -0.04 |
| -0.01 | 0.13 | -0.02 | 0.10 | -0.09 | 0.04 | -0.01 | -0.03 | -0.04 | 0.02 | -0.06 | 0.04 | 0.00 |
| 0.16 | 0.00 | -0.08 | 0.10 | 0.05 | 0.03 | 0.01 | 0.00 | 0.01 | -0.01 | 0.05 | -0.04 | 0.03 |
| 0.03 | 0.08 | 0.03 | -0.03 | -0.03 | -0.02 | 0.05 | 0.03 | 0.03 | 0.03 | 0.03 | 0.01 | 0.01 |
| 0.02 | 0.04 | -0.07 | 0.08 | 0.04 | -0.04 | -0.01 | -0.08 | -0.02 | 0.03 | 0.01 | 0.13 | 0.04 |
| -0.03 | -0.05 | -0.02 | 0.01 | 0.02 | 0.07 | -0.04 | -0.03 | -0.01 | -0.04 | -0.02 | 0.00 | 0.00 |
| 0.02 | 0.03 | -0.05 | 0.15 | -0.07 | 0.05 | -0.02 | -0.02 | -0.06 | 0.05 | -0.03 | 0.06 | 0.00 |

| | | | | | | | | | | | | |
|---|---|---|---|---|---|---|---|---|---|---|---|---|
| 0.03 | -0.04 | 0.04 | -0.06 | 0.00 | 0.02 | 0.04 | -0.08 | -0.03 | -0.04 | -0.13 | -0.03 | 0.01 |
| -0.02 | -0.04 | -0.05 | 0.01 | -0.04 | 0.00 | -0.02 | -0.04 | -0.01 | 0.04 | -0.01 | -0.04 | -0.02 |
| 0.02 | 0.02 | 0.06 | 0.04 | 0.08 | 0.09 | -0.06 | -0.01 | -0.02 | 0.04 | 0.04 | -0.04 | -0.02 |
| -0.04 | -0.02 | -0.01 | -0.06 | 0.07 | 0.02 | 0.01 | -0.01 | -0.01 | 0.03 | -0.03 | 0.03 | 0.00 |
| 0.01 | 0.01 | -0.01 | 0.01 | 0.04 | 0.05 | 0.04 | 0.00 | 0.05 | 0.04 | -0.04 | -0.04 | 0.04 |
| -0.01 | -0.02 | 0.03 | -0.02 | -0.01 | 0.03 | 0.00 | 0.05 | 0.01 | -0.01 | 0.00 | 0.02 | -0.01 |
| 0.04 | -0.03 | 0.06 | -0.03 | -0.02 | 0.00 | -0.02 | 0.00 | -0.04 | -0.01 | 0.01 | 0.00 | 0.06 |
| 0.04 | 0.06 | 0.05 | 0.00 | -0.04 | 0.00 | 0.04 | 0.04 | -0.03 | 0.02 | -0.02 | 0.02 | 0.05 |
| 0.05 | -0.10 | -0.04 | -0.06 | -0.05 | 0.00 | 0.02 | 0.06 | 0.05 | -0.05 | -0.04 | -0.03 | -0.03 |
| 0.06 | -0.06 | 0.05 | 0.02 | -0.02 | 0.03 | -0.02 | 0.01 | -0.04 | 0.09 | 0.02 | 0.03 | -0.05 |
| 0.00 | 0.04 | 0.00 | 0.03 | 0.03 | 0.03 | 0.01 | 0.02 | -0.02 | -0.06 | 0.02 | 0.04 | 0.05 |
| 0.07 | -0.08 | 0.05 | -0.05 | -0.10 | 0.08 | -0.03 | -0.11 | -0.06 | 0.03 | 0.05 | 0.05 | 0.07 |
| -0.01 | 0.05 | -0.04 | -0.02 | 0.00 | -0.03 | -0.04 | 0.04 | 0.02 | -0.05 | 0.00 | -0.04 | -0.03 |
| 0.01 | -0.06 | 0.03 | 0.03 | 0.05 | -0.04 | 0.00 | 0.04 | -0.01 | 0.05 | -0.05 | 0.07 | 0.06 |
| 0.02 | 0.03 | -0.08 | 0.03 | 0.01 | -0.04 | 0.02 | 0.00 | 0.04 | 0.03 | -0.06 | 0.04 | 0.05 |
| 0.03 | -0.02 | 0.01 | -0.03 | 0.12 | 0.03 | 0.04 | -0.07 | 0.04 | 0.02 | 0.00 | 0.03 | 0.00 |
| -0.04 | 0.01 | 0.01 | 0.01 | 0.01 | 0.02 | -0.05 | -0.02 | 0.08 | -0.03 | 0.02 | 0.08 | -0.09 |
| 0.08 | -0.01 | 0.03 | -0.03 | -0.01 | -0.03 | 0.08 | 0.01 | -0.04 | -0.09 | -0.10 | 0.05 | 0.01 |
| 0.07 | -0.04 | -0.05 | 0.03 | 0.00 | 0.08 | 0.00 | -0.09 | 0.01 | -0.05 | 0.01 | 0.02 | -0.13 |
| -0.04 | -0.01 | 0.05 | 0.01 | 0.07 | 0.10 | -0.02 | 0.05 | -0.12 | -0.06 | 0.03 | -0.06 | 0.03 |
| -0.08 | -0.03 | 0.00 | 0.03 | -0.02 | 0.00 | 0.00 | 0.03 | 0.01 | 0.04 | 0.04 | 0.01 | -0.06 |
| -0.02 | -0.02 | -0.09 | 0.10 | -0.09 | -0.01 | 0.00 | 0.02 | 0.07 | -0.04 | 0.09 | 0.10 | 0.09 |
| 0.08 | 0.06 | -0.02 | 0.03 | -0.01 | 0.04 | 0.01 | -0.04 | -0.12 | 0.00 | 0.01 | 0.04 | 0.01 |
| 0.00 | 0.02 | -0.01 | -0.02 | -0.04 | -0.01 | -0.01 | -0.02 | 0.01 | 0.00 | 0.04 | 0.02 | -0.04 |
| 0.02 | 0.05 | -0.08 | -0.09 | -0.03 | 0.05 | -0.04 | 0.00 | 0.04 | 0.03 | 0.08 | 0.05 | -0.03 |
| 0.02 | 0.02 | 0.04 | -0.02 | 0.07 | -0.06 | 0.02 | -0.04 | -0.01 | -0.10 | 0.01 | 0.00 | -0.05 |
| 0.01 | 0.02 | 0.02 | -0.09 | 0.05 | 0.00 | 0.02 | 0.05 | -0.03 | -0.04 | 0.01 | 0.05 | 0.00 |
| -0.07 | -0.02 | -0.02 | 0.04 | -0.01 | -0.01 | -0.05 | 0.12 | -0.01 | 0.04 | -0.02 | -0.04 | 0.02 |
| -0.06 | 0.00 | -0.05 | -0.03 | -0.02 | -0.08 | -0.07 | 0.04 | 0.03 | 0.06 | -0.03 | 0.05 | 0.00 |
| -0.06 | 0.02 | -0.01 | -0.05 | -0.04 | 0.00 | 0.04 | -0.06 | 0.01 | -0.07 | 0.03 | 0.02 | -0.02 |
| -0.09 | 0.00 | 0.00 | 0.02 | 0.03 | 0.03 | -0.01 | 0.06 | 0.05 | 0.06 | -0.05 | 0.04 | 0.04 |

| | | | | | | | | | | | | |
|---|---|---|---|---|---|---|---|---|---|---|---|---|
| -0.03 | 0.04 | -0.07 | 0.04 | 0.07 | -0.05 | 0.00 | -0.09 | -0.08 | -0.03 | -0.08 | -0.02 | 0.15 |
| -0.04 | -0.12 | -0.01 | 0.05 | -0.01 | -0.15 | 0.02 | 0.04 | -0.05 | 0.03 | -0.05 | 0.00 | 0.02 |
| 0.01 | -0.10 | 0.01 | -0.05 | 0.00 | 0.01 | -0.12 | -0.03 | -0.02 | 0.04 | 0.00 | -0.06 | 0.03 |
| 0.00 | 0.02 | -0.05 | 0.03 | -0.03 | 0.06 | -0.07 | 0.03 | -0.02 | -0.06 | 0.01 | 0.04 | -0.01 |
| 0.10 | -0.06 | 0.04 | -0.06 | 0.02 | -0.01 | 0.00 | -0.07 | 0.05 | 0.06 | -0.03 | 0.07 | 0.02 |
| 0.01 | 0.06 | 0.04 | -0.02 | -0.01 | -0.01 | 0.09 | 0.02 | 0.10 | -0.01 | -0.01 | -0.01 | 0.04 |
| 0.05 | -0.01 | 0.02 | 0.00 | -0.04 | -0.02 | -0.07 | -0.03 | -0.03 | 0.05 | -0.02 | 0.03 | 0.08 |
| -0.02 | -0.05 | -0.03 | -0.03 | -0.02 | 0.04 | -0.02 | -0.05 | 0.04 | 0.00 | 0.01 | -0.02 | -0.02 |
| -0.08 | 0.04 | -0.04 | 0.10 | -0.04 | 0.01 | -0.05 | -0.04 | 0.04 | -0.02 | -0.03 | -0.01 | 0.05 |
| 0.03 | -0.05 | 0.03 | -0.04 | 0.02 | 0.01 | 0.05 | 0.04 | 0.01 | -0.01 | 0.03 | -0.02 | -0.03 |
| -0.04 | 0.07 | 0.00 | -0.04 | -0.02 | 0.04 | 0.03 | 0.01 | 0.00 | -0.04 | 0.07 | -0.02 | 0.04 |
| -0.06 | -0.04 | 0.01 | 0.00 | 0.04 | -0.03 | 0.07 | 0.06 | -0.04 | -0.03 | 0.06 | 0.03 | 0.12 |
| 0.00 | -0.03 | -0.11 | -0.02 | 0.05 | 0.05 | -0.06 | 0.05 | 0.07 | 0.06 | 0.00 | 0.06 | 0.00 |
| -0.01 | 0.05 | -0.07 | -0.05 | -0.01 | 0.05 | -0.07 | 0.03 | 0.07 | 0.05 | 0.06 | 0.03 | -0.01 |
| 0.00 | -0.06 | 0.01 | 0.00 | 0.02 | 0.01 | -0.05 | 0.05 | 0.03 | 0.00 | 0.05 | -0.09 | 0.04 |
| 0.00 | -0.04 | -0.12 | -0.01 | 0.00 | -0.01 | -0.07 | 0.04 | -0.04 | 0.01 | 0.07 | 0.01 | 0.09 |
| 0.02 | 0.06 | 0.01 | 0.04 | -0.03 | -0.13 | 0.04 | -0.03 | -0.08 | 0.02 | 0.02 | 0.01 | -0.01 |
| 0.01 | 0.03 | -0.07 | -0.06 | 0.00 | 0.02 | 0.00 | -0.01 | 0.00 | -0.03 | 0.02 | 0.01 | -0.04 |
| 0.04 | 0.02 | -0.06 | -0.03 | 0.07 | -0.02 | 0.01 | 0.01 | 0.03 | -0.03 | 0.07 | 0.06 | 0.00 |
| 0.03 | -0.06 | 0.01 | -0.06 | 0.04 | -0.05 | -0.03 | 0.14 | -0.09 | -0.03 | -0.04 | -0.01 | 0.05 |
| -0.11 | 0.03 | -0.01 | 0.05 | 0.05 | -0.01 | 0.07 | 0.00 | 0.06 | -0.08 | -0.03 | -0.07 | -0.07 |
| 0.05 | 0.01 | 0.04 | -0.01 | -0.01 | 0.01 | -0.06 | 0.06 | -0.08 | 0.02 | -0.06 | -0.08 | 0.01 |
| 0.00 | -0.01 | -0.02 | 0.01 | 0.05 | 0.00 | -0.05 | 0.04 | 0.00 | -0.01 | 0.06 | 0.02 | -0.01 |
| -0.08 | 0.01 | 0.02 | -0.06 | -0.02 | 0.05 | 0.03 | 0.00 | -0.03 | -0.02 | -0.03 | 0.05 | 0.00 |
| -0.07 | 0.05 | 0.05 | 0.00 | -0.07 | -0.04 | 0.01 | 0.05 | 0.03 | 0.03 | 0.00 | -0.01 | 0.01 |
| 0.05 | 0.01 | -0.03 | 0.03 | 0.01 | 0.04 | -0.01 | 0.02 | 0.00 | 0.01 | -0.08 | 0.01 | 0.04 |
| -0.01 | -0.04 | 0.01 | 0.08 | 0.00 | -0.13 | 0.03 | -0.01 | -0.02 | 0.00 | 0.03 | 0.04 | 0.04 |
| 0.12 | -0.04 | 0.10 | 0.02 | -0.10 | 0.00 | 0.05 | 0.02 | -0.02 | -0.01 | 0.01 | -0.01 | 0.05 |
| 0.10 | 0.00 | -0.06 | 0.04 | 0.00 | 0.00 | 0.03 | -0.01 | -0.02 | -0.01 | 0.03 | 0.00 | 0.01 |
| 0.04 | 0.05 | 0.04 | 0.08 | 0.03 | -0.07 | -0.03 | 0.00 | -0.15 | 0.00 | 0.07 | 0.00 | 0.01 |
| 0.07 | -0.04 | -0.09 | 0.04 | 0.04 | 0.13 | 0.01 | -0.03 | -0.04 | -0.07 | 0.01 | 0.01 | 0.02 |

| | | | | | | | | | | | | |
|---|---|---|---|---|---|---|---|---|---|---|---|---|
| 0.04 | -0.08 | -0.09 | 0.01 | -0.01 | 0.02 | 0.09 | 0.02 | -0.03 | 0.00 | -0.09 | -0.10 | 0.03 |
| -0.05 | 0.04 | -0.09 | 0.01 | 0.06 | -0.01 | 0.08 | 0.01 | -0.10 | -0.03 | 0.03 | -0.03 | 0.01 |
| -0.13 | -0.04 | 0.02 | -0.06 | -0.03 | 0.01 | 0.00 | -0.02 | -0.06 | -0.02 | -0.06 | 0.05 | -0.02 |
| 0.01 | -0.06 | 0.03 | -0.04 | 0.00 | -0.01 | -0.08 | 0.03 | -0.02 | 0.09 | -0.04 | -0.01 | -0.01 |
| -0.04 | -0.01 | -0.04 | 0.05 | 0.00 | -0.01 | -0.02 | -0.01 | 0.00 | -0.04 | -0.01 | -0.05 | -0.03 |
| 0.07 | -0.09 | -0.02 | 0.04 | -0.10 | 0.04 | 0.05 | 0.06 | 0.01 | -0.04 | -0.02 | 0.14 | -0.02 |
| 0.01 | -0.03 | 0.03 | 0.01 | -0.06 | -0.09 | -0.01 | -0.08 | -0.07 | 0.05 | -0.04 | -0.02 | 0.02 |
| 0.00 | 0.06 | -0.04 | 0.03 | 0.05 | -0.03 | 0.06 | 0.00 | -0.06 | -0.05 | -0.02 | 0.03 | -0.02 |
| -0.01 | -0.02 | 0.00 | 0.02 | 0.00 | 0.01 | 0.08 | -0.02 | 0.09 | 0.03 | 0.00 | 0.03 | 0.01 |
| -0.03 | 0.03 | 0.02 | -0.04 | 0.02 | 0.05 | -0.05 | -0.05 | 0.00 | -0.04 | -0.10 | -0.01 | -0.02 |
| -0.07 | 0.04 | 0.05 | -0.06 | -0.02 | 0.02 | 0.01 | 0.01 | 0.02 | 0.00 | -0.01 | 0.02 | -0.01 |
| -0.09 | 0.02 | 0.02 | 0.03 | -0.06 | 0.00 | 0.04 | -0.02 | -0.04 | 0.03 | -0.08 | -0.01 | 0.05 |
| 0.04 | 0.01 | -0.04 | 0.07 | -0.06 | -0.03 | 0.05 | 0.03 | -0.06 | 0.00 | -0.05 | -0.02 | -0.03 |
| -0.02 | 0.11 | -0.01 | 0.07 | 0.00 | -0.03 | 0.02 | 0.10 | -0.05 | -0.06 | -0.08 | -0.04 | 0.00 |
| 0.04 | -0.03 | -0.03 | 0.02 | -0.01 | -0.06 | -0.01 | -0.02 | -0.02 | 0.00 | 0.01 | -0.01 | -0.01 |
| 0.01 | -0.04 | 0.03 | 0.01 | -0.01 | 0.07 | 0.03 | -0.01 | -0.11 | 0.06 | -0.04 | -0.03 | -0.02 |
| -0.04 | 0.10 | -0.02 | 0.04 | 0.00 | 0.00 | 0.01 | -0.01 | -0.02 | -0.09 | -0.02 | -0.03 | 0.05 |
| 0.03 | -0.03 | 0.08 | -0.03 | -0.01 | 0.01 | -0.06 | 0.03 | -0.01 | 0.07 | 0.02 | -0.11 | 0.08 |
| 0.00 | 0.04 | -0.04 | 0.04 | 0.04 | 0.00 | 0.00 | 0.08 | 0.09 | -0.04 | -0.04 | -0.07 | 0.02 |
| -0.06 | 0.07 | -0.01 | 0.05 | 0.02 | -0.07 | 0.03 | -0.05 | 0.05 | 0.04 | 0.00 | -0.02 | -0.03 |
| 0.06 | 0.01 | 0.07 | 0.07 | 0.00 | -0.01 | 0.06 | 0.03 | 0.00 | 0.00 | 0.01 | 0.00 | -0.01 |
| 0.01 | 0.03 | 0.02 | 0.05 | 0.00 | -0.03 | 0.02 | 0.02 | 0.00 | 0.03 | 0.04 | 0.02 | -0.02 |
| -0.01 | 0.11 | -0.06 | -0.05 | -0.04 | 0.02 | -0.01 | 0.00 | 0.00 | 0.06 | 0.02 | -0.04 | 0.06 |
| 0.02 | 0.01 | 0.02 | 0.00 | 0.00 | -0.07 | -0.06 | -0.04 | 0.03 | 0.06 | 0.00 | -0.01 | 0.02 |
| 0.06 | 0.08 | 0.01 | -0.11 | -0.03 | 0.07 | 0.04 | 0.02 | 0.00 | 0.04 | -0.03 | 0.02 | 0.00 |
| -0.04 | -0.03 | 0.05 | 0.01 | 0.01 | 0.03 | 0.01 | -0.07 | 0.05 | 0.05 | 0.07 | 0.06 | 0.16 |
| -0.06 | 0.04 | 0.04 | 0.00 | -0.01 | -0.03 | 0.03 | -0.05 | -0.05 | 0.00 | 0.10 | 0.03 | 0.08 |
| -0.19 | 0.01 | 0.09 | -0.06 | -0.01 | -0.05 | 0.01 | 0.03 | -0.01 | 0.04 | -0.06 | 0.03 | -0.01 |
| -0.09 | -0.13 | 0.04 | 0.01 | -0.02 | 0.05 | 0.00 | 0.15 | -0.03 | 0.01 | -0.01 | -0.01 | 0.05 |
| -0.03 | 0.00 | 0.00 | -0.04 | 0.00 | -0.02 | 0.02 | 0.02 | -0.04 | 0.05 | 0.05 | 0.00 | -0.05 |
| 0.04 | 0.04 | 0.00 | 0.04 | -0.07 | -0.06 | -0.05 | -0.04 | 0.04 | 0.01 | -0.01 | -0.03 | -0.01 |

| | | | | | | | | | | | | |
|---|---|---|---|---|---|---|---|---|---|---|---|---|
| 0.04 | -0.01 | -0.04 | 0.01 | 0.02 | -0.07 | -0.06 | -0.01 | 0.02 | -0.08 | -0.05 | 0.18 | 0.08 |
| -0.06 | -0.04 | 0.09 | 0.05 | 0.02 | -0.03 | 0.00 | 0.02 | 0.01 | -0.02 | -0.02 | 0.08 | -0.04 |
| 0.01 | 0.00 | -0.04 | 0.06 | -0.07 | -0.04 | -0.06 | 0.06 | 0.06 | -0.07 | -0.03 | -0.01 | -0.05 |
| 0.09 | -0.09 | 0.08 | -0.03 | -0.04 | -0.05 | -0.07 | -0.03 | 0.01 | -0.01 | 0.03 | -0.04 | 0.07 |
| -0.03 | 0.04 | -0.05 | 0.04 | -0.02 | -0.04 | 0.00 | -0.02 | 0.02 | 0.02 | -0.11 | 0.04 | -0.02 |
| 0.00 | -0.08 | 0.03 | 0.00 | 0.07 | -0.01 | -0.03 | -0.01 | 0.11 | 0.00 | 0.04 | -0.01 | 0.05 |
| 0.02 | 0.03 | -0.04 | 0.02 | -0.01 | -0.07 | -0.06 | -0.02 | 0.02 | -0.03 | 0.03 | 0.04 | -0.07 |
| 0.00 | -0.01 | -0.01 | 0.00 | 0.00 | -0.01 | -0.03 | 0.01 | 0.06 | 0.00 | 0.02 | 0.01 | -0.01 |
| 0.06 | 0.01 | 0.08 | 0.02 | 0.01 | -0.14 | 0.00 | 0.00 | 0.02 | 0.06 | -0.03 | -0.03 | -0.03 |
| 0.00 | 0.07 | -0.07 | -0.07 | -0.02 | -0.03 | -0.02 | 0.04 | -0.02 | -0.06 | 0.02 | -0.01 | 0.12 |
| 0.01 | -0.07 | 0.02 | 0.00 | -0.05 | 0.00 | -0.05 | 0.06 | 0.01 | 0.00 | -0.03 | 0.06 | 0.08 |
| 0.00 | -0.03 | -0.04 | 0.00 | -0.02 | -0.01 | 0.13 | -0.09 | 0.09 | -0.06 | -0.03 | 0.10 | 0.01 |
| -0.03 | -0.04 | 0.05 | 0.02 | -0.05 | 0.02 | 0.03 | 0.00 | -0.04 | -0.03 | 0.04 | 0.02 | -0.03 |
| 0.13 | 0.05 | -0.03 | 0.06 | 0.04 | 0.01 | 0.03 | 0.03 | 0.04 | 0.01 | 0.05 | -0.04 | 0.03 |
| -0.06 | 0.03 | 0.05 | -0.06 | -0.05 | -0.09 | 0.02 | 0.01 | 0.00 | 0.04 | -0.01 | 0.02 | -0.02 |
| 0.00 | 0.04 | 0.04 | -0.02 | 0.00 | 0.05 | -0.02 | 0.03 | 0.07 | 0.03 | 0.07 | -0.01 | -0.02 |
| 0.03 | 0.01 | 0.09 | 0.01 | 0.01 | 0.03 | 0.03 | 0.01 | 0.08 | 0.02 | 0.00 | -0.07 | 0.00 |
| -0.09 | 0.02 | 0.03 | 0.00 | -0.03 | -0.01 | -0.05 | -0.02 | -0.05 | 0.05 | 0.00 | 0.01 | -0.05 |
| 0.02 | 0.00 | 0.09 | 0.07 | 0.00 | 0.07 | 0.00 | 0.00 | 0.00 | 0.07 | -0.06 | 0.00 | -0.03 |
| -0.01 | 0.10 | 0.00 | -0.01 | -0.13 | 0.03 | 0.01 | -0.04 | -0.06 | 0.10 | 0.04 | 0.03 | 0.02 |
| -0.02 | 0.11 | 0.00 | 0.04 | -0.07 | 0.06 | 0.04 | 0.01 | -0.06 | 0.06 | -0.09 | 0.03 | 0.04 |
| 0.01 | -0.01 | 0.01 | -0.02 | -0.07 | 0.07 | 0.00 | 0.05 | 0.10 | -0.09 | 0.00 | 0.03 | 0.04 |
| -0.06 | 0.02 | 0.05 | 0.06 | -0.08 | 0.00 | 0.04 | 0.05 | -0.03 | -0.01 | -0.09 | -0.06 | -0.08 |
| 0.00 | 0.01 | 0.02 | 0.02 | -0.03 | -0.04 | -0.03 | 0.01 | -0.02 | 0.00 | 0.03 | -0.06 | -0.01 |
| 0.01 | 0.07 | 0.02 | 0.01 | 0.01 | -0.02 | 0.13 | -0.04 | -0.06 | 0.10 | 0.12 | -0.02 | -0.10 |
| -0.03 | 0.08 | -0.03 | 0.05 | 0.03 | 0.02 | 0.06 | 0.03 | 0.00 | 0.07 | 0.08 | -0.04 | 0.10 |
| -0.03 | -0.07 | -0.05 | 0.03 | -0.10 | -0.01 | 0.08 | 0.00 | 0.01 | 0.04 | -0.03 | -0.05 | -0.10 |
| 0.01 | -0.02 | -0.06 | 0.13 | -0.05 | 0.01 | -0.04 | -0.02 | -0.10 | 0.01 | 0.06 | 0.07 | -0.03 |
| 0.02 | -0.03 | 0.04 | -0.08 | 0.03 | 0.04 | 0.01 | -0.01 | -0.02 | 0.02 | 0.06 | 0.00 | -0.04 |
| 0.03 | -0.03 | -0.08 | -0.03 | 0.07 | -0.04 | 0.09 | 0.00 | 0.05 | -0.04 | -0.04 | 0.06 | -0.03 |
| 0.01 | -0.05 | -0.03 | -0.05 | -0.07 | -0.02 | 0.07 | 0.10 | 0.00 | 0.08 | -0.05 | 0.05 | 0.00 |

| | | | | | | | | | | | | |
|---|---|---|---|---|---|---|---|---|---|---|---|---|
| -0.03 | 0.03 | 0.01 | -0.02 | 0.07 | 0.03 | 0.05 | -0.03 | -0.11 | -0.10 | -0.03 | 0.04 | -0.06 |
| 0.00 | 0.08 | 0.05 | -0.06 | -0.05 | -0.05 | 0.04 | 0.03 | 0.03 | 0.04 | 0.02 | 0.01 | 0.00 |
| 0.01 | 0.07 | 0.09 | -0.01 | 0.03 | 0.03 | 0.07 | -0.09 | 0.00 | -0.06 | -0.02 | -0.01 | 0.05 |
| 0.01 | 0.02 | -0.05 | -0.04 | 0.03 | 0.03 | -0.01 | 0.06 | -0.04 | -0.02 | -0.04 | -0.04 | -0.01 |
| 0.00 | 0.01 | -0.05 | -0.07 | -0.03 | 0.02 | -0.04 | 0.01 | -0.05 | -0.03 | 0.03 | 0.03 | 0.01 |
| 0.02 | 0.03 | -0.01 | 0.05 | 0.05 | 0.04 | 0.01 | -0.01 | 0.06 | 0.02 | 0.03 | 0.05 | -0.02 |
| -0.02 | -0.01 | -0.09 | 0.01 | 0.06 | 0.05 | -0.05 | -0.05 | 0.17 | 0.04 | -0.06 | -0.03 | 0.04 |
| -0.03 | -0.04 | -0.04 | 0.15 | -0.02 | 0.00 | 0.02 | 0.02 | 0.00 | 0.00 | -0.09 | -0.08 | 0.02 |
| -0.01 | 0.08 | -0.01 | 0.09 | 0.05 | 0.04 | 0.07 | 0.00 | 0.05 | 0.10 | 0.06 | 0.05 | 0.01 |
| 0.09 | -0.09 | -0.11 | 0.07 | 0.04 | 0.12 | -0.02 | -0.05 | -0.05 | -0.08 | 0.01 | 0.00 | 0.01 |
| -0.09 | -0.08 | -0.03 | -0.02 | 0.04 | 0.00 | -0.10 | -0.05 | 0.05 | 0.03 | 0.04 | 0.04 | -0.01 |
| -0.10 | -0.06 | -0.01 | 0.01 | 0.11 | -0.03 | 0.01 | 0.07 | -0.02 | -0.01 | -0.01 | -0.03 | 0.17 |
| -0.09 | -0.02 | -0.03 | -0.07 | -0.03 | -0.02 | -0.09 | -0.01 | -0.09 | -0.04 | -0.04 | -0.05 | -0.02 |
| -0.08 | -0.07 | -0.02 | 0.00 | -0.02 | 0.16 | 0.01 | -0.09 | 0.06 | -0.14 | -0.10 | 0.07 | 0.08 |
| -0.01 | 0.00 | 0.03 | -0.08 | -0.07 | 0.03 | 0.02 | 0.06 | -0.07 | 0.00 | 0.09 | 0.01 | 0.04 |
| 0.02 | -0.02 | 0.00 | 0.02 | -0.08 | -0.08 | 0.05 | -0.07 | -0.05 | 0.05 | -0.06 | 0.00 | -0.03 |
| -0.05 | 0.05 | -0.07 | 0.07 | -0.02 | -0.03 | -0.07 | 0.01 | 0.09 | 0.03 | -0.02 | 0.01 | 0.08 |
| 0.00 | -0.02 | -0.07 | 0.01 | 0.09 | -0.04 | 0.05 | -0.03 | -0.01 | 0.03 | -0.01 | 0.04 | 0.03 |
| 0.02 | 0.10 | 0.06 | 0.01 | 0.03 | 0.03 | 0.00 | -0.04 | 0.00 | 0.04 | 0.01 | -0.08 | 0.03 |
| -0.05 | 0.04 | -0.05 | -0.03 | 0.03 | 0.01 | 0.01 | 0.01 | -0.04 | 0.01 | 0.00 | 0.07 | 0.07 |
| 0.02 | -0.01 | -0.05 | -0.02 | -0.02 | 0.05 | 0.01 | 0.02 | 0.01 | 0.00 | 0.00 | 0.04 | 0.02 |
| 0.00 | 0.06 | -0.05 | -0.07 | 0.03 | 0.03 | 0.01 | -0.01 | -0.03 | 0.02 | -0.04 | -0.04 | -0.03 |

| PC26 | PC27 | PC28 | PC29 | PC30 | PC31 | PC32 |
|---|---|---|---|---|---|---|
| -0.09 | 0.03 | 0.04 | 0.03 | 0.04 | 0.05 | 0.26 |
| 0.02 | -0.02 | -0.03 | -0.01 | -0.10 | -0.03 | -0.04 |
| 0.00 | -0.03 | -0.01 | 0.00 | -0.03 | -0.01 | -0.01 |
| 0.03 | -0.07 | -0.09 | -0.06 | 0.03 | 0.00 | 0.27 |
| 0.08 | -0.05 | -0.10 | -0.02 | 0.07 | -0.06 | 0.08 |
| 0.03 | 0.06 | -0.06 | 0.00 | -0.02 | -0.04 | 0.05 |
| 0.06 | -0.05 | -0.11 | 0.04 | 0.00 | 0.02 | 0.12 |
| 0.09 | 0.08 | -0.06 | 0.02 | 0.01 | 0.04 | 0.11 |
| -0.06 | -0.11 | -0.01 | 0.00 | -0.01 | -0.07 | 0.03 |
| -0.06 | 0.04 | 0.01 | 0.00 | 0.01 | -0.02 | -0.02 |
| 0.00 | 0.05 | 0.05 | -0.07 | -0.03 | -0.01 | -0.34 |
| -0.06 | -0.03 | -0.03 | -0.02 | 0.04 | -0.04 | 0.07 |
| 0.06 | 0.00 | -0.04 | 0.07 | 0.01 | 0.00 | 0.10 |
| -0.02 | 0.01 | 0.11 | -0.03 | 0.03 | 0.01 | 0.39 |
| -0.01 | 0.01 | 0.02 | -0.03 | -0.03 | -0.03 | -0.11 |
| -0.11 | 0.05 | 0.00 | -0.02 | -0.08 | -0.11 | 0.13 |
| -0.09 | 0.03 | -0.03 | 0.02 | -0.04 | -0.03 | -0.15 |
| -0.03 | 0.04 | 0.04 | -0.06 | -0.01 | -0.03 | -0.34 |
| -0.01 | -0.01 | -0.02 | 0.01 | 0.00 | 0.00 | 0.19 |
| -0.13 | -0.04 | 0.01 | 0.13 | 0.03 | -0.06 | -0.02 |
| 0.03 | 0.02 | 0.05 | -0.01 | 0.15 | 0.00 | -0.11 |
| 0.01 | -0.02 | 0.01 | 0.00 | -0.01 | -0.01 | 0.23 |
| -0.11 | 0.05 | -0.07 | -0.09 | 0.02 | -0.02 | 0.18 |
| -0.04 | -0.05 | 0.05 | 0.03 | 0.02 | 0.04 | 0.14 |
| -0.05 | -0.03 | 0.02 | 0.01 | -0.01 | 0.00 | 0.14 |
| 0.06 | 0.04 | 0.06 | 0.04 | -0.01 | -0.01 | 0.12 |
| -0.08 | -0.04 | 0.03 | -0.09 | 0.08 | 0.01 | 0.04 |
| 0.01 | -0.01 | 0.00 | -0.01 | 0.02 | 0.03 | 0.02 |
| -0.05 | 0.03 | 0.04 | -0.03 | -0.04 | -0.01 | -0.18 |

| | | | | | | |
|---|---|---|---|---|---|---|
| -0.05 | -0.01 | -0.02 | -0.01 | -0.08 | 0.00 | 0.07 |
| 0.00 | 0.04 | 0.04 | -0.02 | -0.07 | 0.02 | -0.11 |
| 0.09 | -0.03 | 0.04 | 0.03 | 0.01 | 0.04 | 0.02 |
| 0.01 | -0.09 | -0.01 | -0.10 | -0.03 | -0.03 | 0.00 |
| -0.02 | 0.02 | 0.01 | -0.01 | -0.07 | 0.02 | 0.03 |
| -0.03 | 0.06 | -0.02 | 0.00 | 0.02 | 0.03 | 0.01 |
| -0.05 | 0.05 | 0.01 | -0.04 | 0.03 | -0.05 | 0.00 |
| 0.01 | 0.07 | 0.03 | -0.02 | -0.01 | 0.10 | 0.02 |
| -0.02 | 0.00 | -0.01 | -0.01 | -0.02 | 0.02 | 0.00 |
| 0.00 | 0.03 | 0.03 | 0.13 | 0.03 | -0.15 | -0.02 |
| 0.02 | -0.10 | 0.00 | -0.02 | -0.09 | 0.01 | -0.03 |
| -0.05 | -0.02 | -0.04 | 0.04 | -0.02 | 0.01 | 0.03 |
| 0.01 | -0.03 | 0.03 | -0.01 | -0.02 | -0.01 | -0.03 |
| -0.04 | -0.05 | -0.02 | -0.03 | 0.04 | -0.14 | 0.03 |
| 0.01 | 0.02 | 0.07 | -0.02 | -0.06 | 0.03 | 0.03 |
| 0.00 | -0.01 | 0.01 | -0.14 | -0.13 | -0.07 | 0.03 |
| -0.01 | -0.05 | 0.02 | -0.11 | -0.10 | -0.02 | -0.03 |
| -0.03 | -0.03 | -0.01 | 0.11 | -0.03 | 0.07 | 0.00 |
| -0.02 | -0.06 | 0.02 | -0.07 | -0.08 | -0.07 | 0.01 |
| 0.04 | -0.05 | 0.01 | 0.00 | -0.18 | 0.09 | -0.01 |
| 0.10 | -0.05 | 0.05 | 0.03 | 0.12 | -0.05 | 0.02 |
| -0.02 | 0.04 | -0.06 | 0.08 | 0.07 | 0.02 | 0.00 |
| 0.04 | 0.12 | -0.07 | -0.03 | 0.02 | 0.09 | 0.00 |
| -0.02 | 0.02 | -0.04 | 0.02 | 0.04 | 0.01 | -0.01 |
| -0.17 | -0.03 | -0.07 | 0.08 | 0.03 | -0.03 | 0.03 |
| -0.01 | 0.01 | 0.01 | -0.01 | -0.02 | 0.01 | 0.01 |
| 0.00 | 0.00 | 0.01 | 0.02 | 0.00 | 0.06 | 0.00 |
| 0.03 | 0.05 | 0.03 | -0.05 | 0.01 | 0.14 | 0.02 |
| 0.02 | -0.03 | -0.06 | -0.02 | 0.03 | 0.01 | -0.01 |
| 0.00 | -0.05 | -0.01 | -0.03 | 0.01 | -0.14 | -0.01 |
| 0.00 | 0.00 | -0.07 | 0.01 | 0.03 | -0.05 | -0.03 |

| | | | | | | |
|---|---|---|---|---|---|---|
| -0.05 | 0.00 | -0.01 | 0.00 | 0.02 | 0.05 | 0.02 |
| -0.03 | 0.02 | -0.01 | -0.01 | 0.04 | 0.13 | -0.02 |
| 0.05 | -0.03 | -0.08 | -0.02 | -0.04 | -0.01 | 0.00 |
| 0.09 | 0.04 | -0.17 | -0.01 | -0.07 | -0.02 | -0.01 |
| -0.11 | -0.03 | 0.00 | -0.10 | -0.02 | 0.03 | 0.00 |
| 0.01 | 0.06 | 0.09 | -0.09 | -0.01 | -0.03 | 0.02 |
| 0.03 | 0.04 | 0.08 | 0.04 | 0.06 | -0.03 | 0.01 |
| 0.06 | -0.01 | 0.13 | 0.03 | 0.12 | -0.01 | 0.00 |
| 0.00 | 0.04 | -0.03 | -0.08 | 0.02 | -0.02 | 0.00 |
| 0.04 | -0.07 | 0.12 | 0.00 | -0.01 | -0.04 | 0.01 |
| 0.00 | 0.01 | 0.05 | 0.02 | 0.00 | 0.00 | 0.01 |
| 0.01 | -0.03 | -0.04 | -0.02 | -0.07 | 0.05 | -0.01 |
| 0.08 | 0.01 | 0.06 | -0.02 | 0.04 | -0.01 | 0.00 |
| -0.02 | 0.04 | 0.02 | -0.17 | 0.01 | 0.02 | 0.00 |
| 0.03 | -0.04 | -0.12 | 0.14 | -0.06 | -0.04 | -0.02 |
| -0.01 | 0.06 | -0.05 | -0.03 | 0.00 | -0.06 | -0.01 |
| 0.01 | -0.01 | -0.07 | 0.02 | 0.03 | 0.05 | 0.00 |
| 0.00 | 0.04 | -0.05 | 0.01 | 0.00 | -0.01 | 0.01 |
| 0.03 | -0.10 | -0.05 | 0.00 | -0.01 | 0.02 | 0.02 |
| 0.04 | 0.02 | 0.05 | 0.05 | -0.04 | -0.11 | 0.01 |
| 0.00 | 0.02 | -0.04 | 0.05 | -0.03 | 0.08 | 0.00 |
| 0.11 | -0.15 | -0.01 | -0.08 | -0.01 | 0.00 | 0.04 |
| 0.09 | -0.02 | 0.03 | -0.12 | 0.03 | -0.02 | 0.02 |
| 0.08 | 0.00 | -0.10 | -0.09 | -0.03 | -0.06 | 0.00 |
| -0.02 | -0.01 | -0.08 | -0.03 | 0.09 | 0.08 | -0.05 |
| 0.00 | 0.00 | -0.01 | -0.05 | -0.04 | 0.09 | 0.00 |
| -0.05 | -0.02 | -0.08 | -0.02 | -0.01 | -0.04 | -0.01 |
| 0.02 | -0.04 | -0.01 | -0.02 | 0.01 | 0.00 | -0.02 |
| -0.02 | 0.01 | 0.00 | -0.01 | -0.04 | 0.05 | 0.01 |
| 0.04 | -0.04 | 0.05 | -0.05 | -0.02 | 0.00 | 0.02 |
| -0.02 | 0.05 | 0.06 | 0.07 | 0.01 | 0.02 | 0.01 |

| | | | | | | |
|---|---|---|---|---|---|---|
| 0.00 | -0.06 | -0.03 | -0.04 | 0.08 | 0.01 | -0.03 |
| -0.03 | -0.01 | -0.06 | -0.02 | 0.08 | -0.05 | 0.00 |
| 0.02 | -0.03 | 0.01 | -0.02 | -0.04 | 0.03 | -0.01 |
| -0.08 | 0.00 | 0.01 | -0.06 | 0.02 | -0.03 | -0.01 |
| -0.01 | -0.01 | 0.07 | 0.00 | 0.01 | -0.04 | -0.02 |
| -0.07 | 0.10 | -0.06 | 0.13 | 0.02 | -0.03 | -0.02 |
| -0.03 | 0.04 | 0.00 | -0.01 | 0.01 | 0.02 | -0.02 |
| 0.06 | 0.08 | -0.05 | -0.08 | -0.01 | 0.08 | -0.01 |
| 0.00 | 0.01 | 0.01 | 0.06 | 0.00 | -0.06 | 0.00 |
| 0.02 | 0.01 | 0.00 | 0.10 | -0.02 | 0.01 | -0.01 |
| 0.00 | -0.05 | 0.02 | -0.03 | 0.00 | -0.05 | 0.03 |
| -0.05 | 0.00 | 0.01 | -0.02 | -0.03 | -0.01 | 0.01 |
| -0.02 | -0.01 | 0.01 | -0.01 | -0.02 | -0.06 | 0.00 |
| 0.01 | 0.05 | 0.00 | -0.05 | -0.03 | 0.05 | 0.00 |
| -0.06 | -0.02 | -0.08 | 0.00 | 0.04 | -0.05 | -0.01 |
| 0.07 | -0.01 | -0.11 | -0.01 | 0.10 | -0.06 | -0.02 |
| -0.07 | 0.02 | 0.11 | 0.09 | -0.08 | -0.08 | 0.02 |
| 0.03 | -0.01 | 0.04 | -0.07 | 0.05 | 0.01 | -0.01 |
| 0.03 | -0.06 | 0.01 | -0.05 | 0.02 | -0.05 | -0.02 |
| 0.00 | -0.05 | -0.03 | 0.02 | 0.05 | 0.02 | 0.00 |
| 0.04 | 0.03 | -0.03 | -0.07 | 0.01 | 0.02 | 0.01 |
| 0.09 | 0.03 | 0.00 | 0.01 | 0.06 | 0.01 | 0.03 |
| 0.01 | 0.07 | 0.03 | 0.04 | -0.02 | 0.05 | 0.00 |
| -0.08 | 0.03 | -0.04 | 0.03 | -0.01 | 0.06 | 0.01 |
| -0.05 | 0.02 | 0.00 | -0.02 | -0.12 | -0.05 | 0.01 |
| -0.02 | -0.01 | -0.07 | -0.02 | 0.04 | 0.02 | -0.02 |
| -0.11 | -0.06 | -0.07 | -0.06 | -0.03 | 0.05 | -0.01 |
| 0.00 | -0.02 | 0.04 | 0.02 | -0.01 | 0.00 | 0.00 |
| -0.05 | 0.00 | -0.05 | -0.02 | -0.09 | -0.08 | 0.03 |
| 0.00 | -0.07 | -0.10 | -0.08 | -0.06 | -0.06 | -0.01 |
| -0.03 | 0.10 | -0.01 | -0.02 | 0.05 | -0.01 | 0.02 |

| | | | | | | |
|---|---|---|---|---|---|---|
| 0.02 | -0.01 | 0.02 | 0.04 | -0.04 | 0.01 | 0.03 |
| 0.07 | -0.01 | -0.03 | 0.00 | -0.07 | 0.06 | 0.02 |
| -0.03 | 0.02 | -0.01 | 0.01 | 0.02 | -0.02 | 0.01 |
| -0.01 | 0.03 | 0.03 | 0.02 | -0.02 | -0.05 | 0.02 |
| 0.07 | 0.03 | 0.09 | 0.03 | 0.00 | -0.07 | 0.02 |
| 0.04 | -0.05 | 0.06 | 0.02 | -0.04 | -0.02 | 0.02 |
| -0.02 | 0.10 | -0.01 | -0.05 | 0.03 | -0.05 | -0.01 |
| 0.00 | -0.03 | -0.08 | 0.01 | -0.03 | 0.04 | 0.03 |
| -0.02 | -0.05 | -0.06 | 0.05 | 0.03 | 0.06 | -0.01 |
| 0.05 | -0.01 | 0.04 | -0.02 | 0.02 | 0.05 | -0.02 |
| -0.04 | -0.02 | -0.01 | -0.03 | 0.04 | -0.01 | -0.02 |
| -0.09 | 0.01 | -0.01 | 0.07 | 0.08 | 0.03 | -0.04 |
| 0.02 | 0.00 | 0.01 | -0.03 | 0.00 | -0.02 | 0.00 |
| 0.01 | -0.01 | 0.01 | 0.04 | 0.11 | -0.02 | 0.00 |
| -0.03 | 0.00 | 0.05 | 0.02 | -0.04 | 0.05 | -0.01 |
| -0.01 | -0.04 | 0.07 | 0.07 | -0.02 | -0.01 | -0.04 |
| -0.03 | 0.03 | 0.05 | 0.00 | 0.04 | 0.04 | 0.01 |
| 0.07 | -0.09 | -0.11 | -0.03 | -0.02 | 0.08 | 0.00 |
| 0.02 | 0.06 | -0.02 | -0.01 | -0.04 | 0.07 | 0.00 |
| 0.01 | -0.05 | -0.02 | -0.01 | -0.03 | -0.02 | 0.01 |
| -0.04 | 0.05 | 0.00 | -0.03 | 0.05 | 0.01 | -0.01 |
| 0.04 | 0.06 | 0.07 | -0.03 | -0.01 | 0.01 | 0.04 |
| -0.03 | -0.05 | 0.06 | -0.02 | 0.03 | -0.04 | 0.00 |
| 0.00 | 0.01 | 0.04 | 0.02 | 0.00 | 0.00 | -0.04 |
| -0.05 | 0.02 | -0.06 | 0.04 | 0.00 | 0.11 | -0.02 |
| 0.07 | -0.04 | -0.11 | 0.03 | -0.10 | -0.04 | -0.01 |
| 0.00 | -0.02 | -0.04 | -0.05 | -0.10 | -0.01 | 0.01 |
| 0.13 | 0.08 | -0.02 | -0.02 | 0.06 | 0.06 | 0.00 |
| 0.07 | -0.01 | -0.04 | 0.06 | -0.02 | -0.04 | -0.01 |
| -0.07 | 0.06 | -0.01 | 0.04 | -0.07 | -0.08 | 0.02 |
| 0.01 | 0.01 | 0.05 | 0.03 | -0.05 | -0.01 | 0.01 |

| | | | | | | |
|---|---|---|---|---|---|---|
| -0.06 | -0.04 | 0.08 | 0.01 | 0.04 | -0.03 | -0.01 |
| -0.07 | -0.08 | 0.09 | -0.06 | 0.08 | 0.02 | -0.02 |
| -0.12 | -0.06 | 0.03 | -0.11 | 0.05 | 0.00 | -0.01 |
| -0.06 | 0.01 | -0.02 | 0.06 | 0.00 | 0.01 | 0.03 |
| 0.00 | 0.06 | -0.01 | -0.06 | 0.03 | -0.03 | -0.02 |
| -0.01 | -0.08 | 0.00 | -0.02 | -0.01 | 0.07 | 0.04 |
| -0.09 | 0.03 | 0.06 | 0.04 | 0.00 | 0.05 | 0.00 |
| 0.00 | 0.01 | 0.01 | 0.02 | -0.01 | -0.02 | 0.04 |
| -0.12 | 0.17 | 0.07 | 0.02 | 0.00 | -0.04 | 0.00 |
| 0.00 | 0.01 | -0.01 | 0.13 | -0.04 | -0.02 | -0.01 |
| -0.01 | -0.01 | 0.02 | -0.01 | -0.01 | -0.01 | -0.01 |
| -0.02 | -0.01 | 0.00 | -0.03 | -0.02 | 0.05 | 0.01 |
| -0.08 | 0.09 | -0.03 | 0.00 | -0.01 | 0.06 | -0.01 |
| 0.01 | 0.03 | 0.01 | 0.00 | -0.01 | -0.01 | 0.01 |
| 0.02 | -0.01 | 0.03 | -0.03 | -0.09 | 0.10 | 0.01 |
| 0.03 | 0.04 | 0.08 | 0.06 | 0.02 | 0.04 | 0.00 |
| 0.00 | -0.06 | -0.01 | 0.02 | 0.00 | 0.07 | -0.01 |
| -0.02 | -0.07 | -0.05 | -0.06 | 0.06 | -0.06 | 0.01 |
| 0.03 | 0.07 | 0.01 | 0.02 | 0.01 | -0.02 | 0.01 |
| 0.05 | -0.04 | 0.06 | 0.05 | 0.03 | -0.09 | -0.02 |
| 0.07 | -0.04 | 0.00 | 0.04 | -0.01 | -0.02 | -0.02 |
| 0.04 | 0.01 | 0.04 | 0.03 | 0.03 | -0.03 | 0.01 |
| -0.02 | -0.05 | -0.08 | 0.00 | 0.03 | -0.02 | -0.04 |
| -0.01 | 0.01 | -0.03 | 0.03 | -0.01 | 0.02 | 0.02 |
| -0.07 | -0.02 | -0.03 | -0.06 | 0.00 | 0.02 | -0.03 |
| 0.00 | -0.03 | 0.01 | -0.05 | 0.02 | -0.02 | -0.01 |
| -0.05 | -0.05 | -0.04 | 0.01 | 0.00 | -0.09 | 0.00 |
| 0.04 | 0.03 | -0.04 | 0.00 | 0.00 | -0.01 | -0.01 |
| 0.05 | -0.03 | 0.00 | 0.00 | 0.02 | -0.05 | 0.00 |
| 0.06 | 0.00 | 0.00 | 0.00 | 0.04 | -0.03 | 0.01 |
| 0.05 | 0.02 | 0.02 | -0.04 | 0.00 | -0.04 | 0.02 |

| | | | | | | |
|---|---|---|---|---|---|---|
| 0.00 | -0.02 | 0.02 | 0.00 | 0.03 | -0.02 | -0.02 |
| -0.07 | 0.00 | 0.03 | 0.04 | -0.10 | 0.01 | 0.00 |
| 0.00 | 0.01 | -0.03 | 0.02 | 0.03 | -0.05 | 0.00 |
| -0.03 | 0.03 | 0.03 | 0.03 | 0.01 | 0.01 | 0.02 |
| -0.06 | -0.03 | -0.01 | 0.01 | 0.05 | -0.03 | 0.00 |
| 0.04 | -0.03 | 0.15 | -0.02 | 0.01 | 0.04 | -0.02 |
| -0.04 | 0.00 | -0.02 | 0.03 | -0.03 | -0.15 | 0.00 |
| -0.04 | -0.14 | 0.00 | 0.00 | -0.01 | -0.05 | 0.00 |
| 0.00 | 0.02 | -0.02 | -0.01 | 0.04 | 0.01 | 0.01 |
| -0.02 | 0.00 | 0.00 | -0.01 | 0.01 | -0.01 | 0.00 |
| -0.06 | -0.01 | -0.01 | 0.02 | -0.02 | 0.04 | -0.01 |
| 0.02 | -0.01 | -0.03 | -0.04 | -0.02 | 0.03 | 0.02 |
| 0.02 | -0.04 | -0.04 | 0.05 | 0.01 | 0.09 | -0.02 |
| -0.01 | -0.05 | -0.08 | 0.03 | 0.06 | -0.02 | 0.00 |
| 0.05 | 0.02 | -0.03 | -0.02 | 0.00 | -0.03 | -0.02 |
| -0.01 | 0.00 | -0.03 | 0.03 | 0.05 | -0.01 | -0.04 |
| -0.04 | 0.05 | 0.04 | -0.11 | -0.01 | -0.01 | 0.00 |
| 0.00 | -0.02 | 0.03 | -0.03 | -0.01 | -0.01 | -0.02 |
| 0.07 | -0.01 | 0.01 | 0.02 | -0.03 | 0.01 | 0.01 |
| 0.10 | 0.03 | 0.03 | -0.08 | -0.09 | 0.04 | 0.00 |
| 0.10 | -0.03 | -0.07 | 0.05 | -0.10 | -0.12 | -0.01 |
| -0.07 | -0.03 | 0.07 | -0.06 | 0.03 | 0.02 | 0.00 |
| 0.04 | 0.06 | 0.03 | -0.06 | -0.06 | -0.02 | 0.03 |
| -0.10 | -0.05 | -0.01 | -0.03 | -0.03 | -0.01 | 0.00 |
| 0.00 | 0.03 | 0.02 | -0.02 | -0.01 | -0.03 | -0.01 |
| 0.00 | -0.07 | 0.04 | 0.03 | -0.02 | 0.18 | 0.01 |
| -0.02 | 0.03 | -0.01 | -0.09 | 0.03 | 0.05 | -0.01 |
| 0.02 | -0.06 | -0.06 | 0.03 | 0.11 | 0.10 | -0.05 |
| -0.02 | 0.05 | -0.02 | 0.00 | -0.05 | 0.02 | -0.02 |
| -0.01 | 0.00 | -0.01 | 0.06 | 0.09 | 0.02 | -0.01 |
| 0.02 | 0.05 | -0.06 | -0.02 | 0.02 | 0.00 | -0.01 |

| | | | | | | |
|---|---|---|---|---|---|---|
| 0.09 | -0.05 | 0.00 | 0.03 | -0.05 | 0.12 | -0.03 |
| -0.02 | -0.07 | -0.10 | 0.05 | 0.00 | -0.07 | -0.01 |
| -0.01 | -0.01 | 0.02 | 0.00 | -0.03 | -0.01 | -0.02 |
| -0.01 | 0.02 | 0.08 | 0.06 | 0.08 | 0.11 | -0.02 |
| -0.10 | 0.04 | -0.09 | 0.00 | -0.04 | 0.03 | -0.03 |
| -0.13 | -0.04 | 0.06 | 0.03 | -0.03 | 0.09 | -0.03 |
| 0.02 | -0.02 | 0.10 | 0.11 | -0.01 | 0.00 | 0.01 |
| 0.13 | 0.05 | 0.08 | 0.03 | -0.01 | 0.01 | 0.03 |
| -0.02 | 0.00 | -0.01 | 0.08 | -0.07 | 0.00 | 0.02 |
| 0.04 | 0.08 | -0.05 | -0.11 | 0.00 | 0.01 | 0.02 |
| 0.01 | -0.01 | 0.10 | -0.05 | 0.01 | -0.02 | -0.02 |
| 0.06 | 0.04 | -0.04 | -0.02 | 0.04 | 0.03 | -0.01 |
| -0.06 | 0.02 | 0.03 | -0.08 | 0.04 | 0.00 | -0.01 |
| 0.04 | 0.02 | -0.09 | 0.02 | -0.02 | -0.06 | 0.01 |
| -0.01 | 0.02 | -0.04 | 0.01 | 0.00 | 0.02 | 0.02 |
| 0.01 | 0.10 | 0.04 | -0.02 | 0.01 | 0.03 | -0.01 |
| -0.05 | -0.07 | 0.01 | -0.08 | 0.03 | 0.03 | -0.03 |
| 0.01 | -0.01 | 0.05 | 0.00 | -0.03 | 0.00 | 0.02 |
| 0.03 | -0.06 | 0.08 | 0.00 | 0.01 | 0.06 | 0.00 |
| -0.01 | 0.07 | -0.03 | -0.01 | 0.00 | 0.11 | 0.02 |
| -0.01 | -0.02 | 0.06 | -0.07 | 0.05 | 0.07 | 0.00 |
| 0.00 | -0.03 | 0.03 | -0.07 | -0.03 | 0.01 | 0.02 |
| -0.05 | -0.02 | 0.06 | 0.09 | 0.07 | 0.00 | 0.04 |
| -0.07 | 0.01 | 0.01 | 0.01 | 0.05 | -0.05 | 0.00 |
| 0.04 | 0.04 | 0.03 | -0.01 | 0.03 | -0.11 | 0.00 |
| -0.02 | 0.01 | 0.08 | 0.06 | -0.05 | 0.00 | 0.02 |
| -0.05 | -0.02 | 0.01 | 0.10 | -0.01 | 0.04 | 0.01 |
| 0.03 | -0.02 | -0.01 | -0.03 | 0.03 | 0.00 | 0.01 |
| 0.01 | 0.05 | 0.03 | 0.07 | -0.06 | -0.02 | 0.00 |
| 0.00 | -0.04 | -0.03 | -0.02 | 0.04 | -0.02 | -0.02 |
| -0.02 | -0.01 | 0.02 | 0.02 | -0.01 | -0.03 | -0.05 |

| | | | | | | |
|---|---|---|---|---|---|---|
| -0.05 | 0.00 | -0.02 | -0.05 | -0.02 | -0.01 | 0.00 |
| -0.06 | -0.07 | 0.06 | 0.02 | 0.03 | -0.03 | -0.03 |
| 0.00 | -0.01 | -0.01 | 0.05 | -0.08 | -0.01 | -0.03 |
| 0.02 | 0.00 | 0.08 | -0.03 | -0.06 | -0.01 | 0.01 |
| 0.00 | -0.06 | 0.00 | 0.10 | -0.01 | 0.04 | -0.02 |
| -0.04 | 0.10 | -0.01 | 0.00 | 0.01 | -0.04 | -0.01 |
| -0.05 | -0.02 | -0.07 | -0.08 | 0.07 | 0.11 | 0.00 |
| 0.03 | 0.00 | 0.01 | 0.04 | -0.03 | -0.08 | 0.03 |
| 0.02 | 0.09 | 0.11 | -0.03 | 0.05 | 0.00 | 0.00 |
| 0.07 | -0.05 | 0.00 | 0.01 | 0.01 | 0.05 | 0.03 |
| 0.01 | 0.03 | -0.01 | -0.07 | -0.03 | -0.03 | -0.02 |
| 0.08 | 0.12 | 0.02 | 0.05 | -0.10 | -0.08 | -0.01 |
| 0.03 | -0.06 | 0.08 | 0.02 | -0.03 | -0.04 | 0.00 |
| -0.04 | -0.09 | 0.06 | -0.14 | 0.04 | 0.00 | 0.02 |
| -0.05 | -0.06 | 0.01 | 0.03 | 0.05 | 0.04 | 0.00 |
| 0.02 | -0.05 | 0.00 | -0.04 | -0.03 | 0.08 | 0.01 |
| 0.01 | -0.05 | 0.01 | -0.03 | 0.04 | -0.02 | 0.01 |
| -0.01 | -0.01 | -0.03 | -0.04 | -0.01 | -0.01 | -0.02 |
| 0.00 | -0.02 | 0.03 | 0.05 | 0.05 | 0.06 | -0.01 |
| -0.06 | -0.03 | 0.02 | 0.05 | 0.04 | 0.02 | -0.03 |
| 0.01 | 0.02 | -0.02 | -0.01 | 0.00 | -0.02 | -0.04 |
| -0.03 | 0.04 | -0.06 | -0.02 | -0.01 | -0.03 | 0.00 |
| 0.03 | 0.01 | 0.05 | -0.04 | -0.06 | -0.02 | -0.01 |
| -0.03 | 0.03 | -0.07 | -0.01 | -0.03 | -0.03 | -0.02 |
| 0.12 | -0.08 | -0.05 | -0.05 | -0.07 | 0.01 | 0.00 |
| 0.04 | -0.02 | 0.02 | -0.01 | 0.01 | 0.05 | -0.02 |
| 0.03 | 0.00 | 0.00 | -0.04 | 0.00 | 0.03 | 0.01 |
| -0.01 | 0.03 | 0.00 | -0.01 | 0.04 | 0.00 | 0.01 |
| 0.01 | 0.03 | -0.05 | -0.04 | -0.02 | 0.02 | 0.02 |
| 0.01 | -0.05 | 0.01 | -0.03 | -0.02 | -0.01 | -0.01 |
| 0.00 | -0.07 | 0.03 | 0.05 | 0.03 | -0.07 | -0.02 |

| | | | | | | |
|---|---|---|---|---|---|---|
| 0.13 | -0.01 | 0.07 | 0.03 | -0.02 | -0.01 | 0.00 |
| 0.06 | -0.01 | 0.02 | 0.05 | 0.07 | -0.02 | -0.01 |
| -0.04 | 0.06 | -0.08 | 0.05 | 0.11 | -0.07 | -0.03 |
| 0.01 | 0.02 | -0.04 | -0.03 | 0.03 | -0.02 | -0.01 |
| 0.01 | 0.04 | 0.02 | 0.05 | -0.02 | 0.00 | 0.02 |
| 0.05 | 0.07 | -0.05 | 0.06 | 0.07 | 0.04 | 0.01 |
| -0.01 | 0.03 | 0.02 | 0.04 | 0.02 | 0.03 | 0.02 |
| -0.06 | -0.02 | 0.01 | 0.03 | 0.07 | -0.04 | 0.00 |
| 0.02 | -0.02 | 0.01 | -0.01 | 0.09 | 0.05 | 0.00 |
| 0.02 | -0.01 | 0.03 | 0.09 | 0.03 | -0.06 | 0.01 |
| -0.03 | 0.14 | -0.05 | -0.04 | 0.01 | 0.08 | -0.01 |
| 0.03 | -0.04 | -0.02 | 0.02 | 0.07 | -0.07 | 0.01 |
| -0.02 | 0.00 | -0.07 | -0.02 | -0.01 | 0.01 | -0.01 |
| 0.03 | 0.01 | -0.06 | -0.01 | 0.03 | -0.08 | -0.01 |
| -0.07 | 0.07 | 0.07 | -0.02 | 0.11 | 0.04 | 0.03 |
| -0.02 | -0.04 | 0.07 | 0.02 | -0.11 | -0.03 | 0.01 |
| 0.02 | 0.03 | -0.10 | -0.02 | 0.03 | -0.04 | -0.03 |
| -0.02 | 0.01 | 0.03 | -0.03 | 0.02 | -0.04 | -0.01 |
| 0.01 | -0.04 | 0.04 | 0.12 | 0.00 | -0.04 | -0.04 |
| 0.01 | 0.04 | 0.01 | 0.03 | -0.05 | 0.04 | 0.01 |
| 0.06 | -0.04 | -0.04 | -0.04 | 0.00 | 0.03 | -0.01 |
| -0.11 | -0.15 | -0.02 | 0.06 | -0.03 | -0.01 | 0.01 |
| -0.15 | -0.01 | -0.09 | 0.10 | -0.10 | -0.02 | 0.04 |
| -0.04 | 0.04 | -0.04 | 0.02 | 0.05 | -0.05 | -0.01 |
| 0.02 | 0.03 | -0.03 | 0.01 | 0.01 | -0.03 | 0.01 |
| -0.01 | 0.00 | 0.05 | 0.06 | -0.03 | -0.14 | 0.01 |
| -0.05 | 0.00 | 0.04 | -0.05 | 0.01 | -0.03 | 0.03 |
| -0.03 | 0.01 | 0.05 | 0.09 | 0.02 | 0.07 | 0.02 |
| -0.03 | 0.08 | 0.00 | -0.04 | -0.04 | -0.01 | 0.02 |
| -0.04 | 0.06 | -0.03 | 0.03 | 0.04 | -0.06 | 0.00 |
| 0.09 | 0.05 | 0.02 | 0.00 | 0.04 | 0.00 | 0.01 |

| | | | | | | |
|---|---|---|---|---|---|---|
| 0.05 | 0.02 | 0.03 | 0.04 | 0.05 | -0.04 | -0.01 |
| 0.06 | -0.02 | -0.03 | -0.04 | 0.05 | -0.08 | 0.01 |
| -0.03 | 0.04 | 0.00 | -0.02 | -0.08 | 0.00 | 0.04 |
| -0.04 | 0.00 | -0.07 | -0.01 | -0.08 | -0.01 | 0.02 |
| 0.03 | 0.11 | -0.05 | 0.00 | 0.09 | 0.04 | 0.01 |
| 0.03 | 0.06 | -0.02 | 0.07 | -0.05 | -0.03 | 0.01 |
| 0.01 | 0.04 | -0.08 | 0.06 | -0.05 | 0.07 | 0.00 |
| 0.03 | -0.08 | 0.06 | 0.00 | 0.00 | 0.02 | 0.02 |
| 0.06 | 0.05 | -0.03 | 0.09 | -0.01 | 0.04 | -0.01 |
| -0.03 | 0.08 | 0.04 | -0.05 | 0.06 | 0.01 | 0.00 |
| 0.01 | 0.00 | 0.01 | -0.01 | -0.07 | -0.01 | -0.02 |
| 0.04 | 0.13 | 0.00 | -0.07 | -0.01 | -0.07 | 0.02 |
| 0.05 | -0.02 | -0.03 | 0.07 | 0.07 | -0.05 | 0.02 |
| 0.01 | 0.08 | 0.00 | -0.02 | 0.01 | -0.02 | 0.00 |
| -0.06 | 0.01 | -0.06 | -0.01 | 0.01 | -0.06 | -0.01 |
| 0.01 | -0.01 | 0.01 | 0.11 | -0.09 | -0.01 | -0.01 |
| 0.03 | 0.03 | -0.10 | 0.02 | -0.02 | -0.01 | -0.01 |
| -0.04 | 0.00 | -0.02 | -0.02 | 0.03 | -0.02 | 0.01 |
| -0.01 | -0.05 | 0.01 | 0.05 | 0.01 | -0.02 | 0.03 |
| 0.02 | 0.03 | -0.04 | -0.03 | 0.06 | -0.03 | -0.01 |
| 0.04 | -0.06 | -0.08 | -0.04 | 0.02 | 0.02 | -0.05 |
| -0.10 | -0.09 | 0.04 | -0.05 | 0.09 | -0.04 | 0.00 |
| 0.00 | -0.05 | 0.04 | 0.01 | -0.02 | 0.04 | -0.03 |
| -0.01 | 0.00 | -0.04 | 0.00 | 0.01 | 0.00 | -0.02 |
| -0.04 | 0.00 | 0.05 | -0.01 | -0.01 | -0.04 | -0.01 |
| -0.04 | 0.01 | -0.01 | 0.00 | -0.04 | 0.17 | 0.03 |
| 0.06 | 0.00 | 0.00 | -0.05 | -0.08 | -0.07 | 0.03 |
| 0.02 | -0.04 | 0.05 | 0.00 | -0.05 | 0.02 | -0.01 |
| 0.01 | 0.03 | -0.05 | 0.00 | 0.01 | 0.03 | 0.04 |
| 0.02 | 0.05 | -0.01 | -0.05 | 0.01 | -0.03 | 0.01 |
| 0.02 | -0.03 | -0.05 | -0.04 | -0.01 | 0.03 | 0.01 |

| | | | | | | |
|---|---|---|---|---|---|---|
| -0.13 | 0.03 | -0.02 | 0.02 | -0.11 | 0.03 | 0.00 |
| -0.05 | -0.05 | -0.07 | -0.02 | -0.01 | -0.02 | 0.01 |
| -0.02 | 0.01 | -0.05 | 0.04 | -0.02 | 0.00 | -0.03 |
| 0.05 | 0.00 | 0.03 | -0.01 | -0.03 | 0.04 | 0.00 |
| 0.00 | 0.00 | 0.01 | 0.02 | -0.03 | 0.04 | -0.03 |
| 0.00 | 0.04 | -0.03 | -0.10 | -0.07 | 0.01 | 0.00 |
| -0.01 | -0.05 | 0.00 | -0.05 | -0.04 | -0.01 | 0.04 |
| 0.07 | -0.10 | 0.12 | 0.00 | 0.01 | -0.02 | -0.02 |
| -0.04 | 0.04 | 0.03 | -0.03 | -0.05 | 0.01 | 0.01 |
| 0.02 | -0.02 | -0.02 | 0.01 | 0.01 | 0.00 | -0.03 |
| -0.01 | -0.02 | 0.02 | -0.01 | 0.02 | -0.02 | -0.01 |
| 0.03 | 0.01 | -0.01 | 0.05 | 0.02 | 0.00 | -0.02 |
| -0.01 | -0.08 | -0.01 | 0.01 | -0.14 | 0.04 | -0.02 |
| -0.01 | 0.10 | 0.07 | -0.06 | 0.05 | 0.00 | -0.04 |
| 0.00 | 0.02 | -0.02 | 0.03 | -0.01 | 0.01 | 0.00 |
| 0.00 | -0.02 | 0.00 | -0.03 | 0.03 | 0.04 | -0.02 |
| -0.04 | 0.01 | 0.07 | 0.02 | -0.03 | -0.03 | -0.03 |
| -0.03 | -0.11 | 0.09 | -0.03 | -0.09 | 0.00 | -0.02 |
| -0.01 | -0.01 | 0.10 | 0.05 | 0.03 | 0.03 | 0.01 |
| 0.06 | -0.02 | -0.05 | 0.09 | 0.02 | -0.03 | -0.02 |
| -0.03 | 0.02 | 0.02 | 0.02 | -0.01 | 0.01 | -0.02 |
| 0.01 | 0.02 | 0.00 | 0.03 | 0.01 | -0.01 | 0.01 |
| 0.00 | 0.10 | 0.02 | 0.01 | -0.04 | -0.11 | 0.05 |
| 0.05 | -0.01 | -0.06 | -0.07 | 0.07 | -0.09 | 0.03 |
| 0.01 | -0.06 | 0.04 | -0.02 | -0.01 | 0.01 | -0.02 |
| -0.06 | -0.07 | -0.03 | 0.07 | -0.05 | 0.01 | 0.00 |
| 0.04 | 0.09 | -0.01 | 0.10 | 0.09 | -0.04 | -0.05 |
| -0.03 | 0.02 | 0.01 | 0.05 | -0.03 | -0.01 | -0.01 |
| -0.01 | -0.08 | -0.01 | 0.05 | -0.10 | 0.00 | 0.00 |
| 0.02 | -0.08 | -0.07 | 0.02 | 0.03 | 0.08 | -0.05 |
| 0.01 | -0.02 | -0.10 | 0.03 | 0.04 | 0.07 | -0.04 |

| | | | | | | |
|---|---|---|---|---|---|---|
| -0.05 | -0.01 | -0.08 | -0.07 | -0.01 | 0.05 | 0.01 |
| -0.01 | 0.03 | -0.06 | 0.01 | 0.03 | -0.01 | 0.00 |
| 0.01 | 0.01 | 0.08 | -0.02 | -0.08 | 0.03 | 0.00 |
| 0.01 | 0.05 | -0.07 | -0.02 | -0.09 | 0.01 | 0.01 |
| -0.05 | -0.09 | -0.02 | 0.06 | 0.01 | 0.03 | -0.01 |
| -0.03 | 0.09 | 0.08 | 0.00 | -0.12 | 0.04 | 0.02 |
| -0.12 | 0.06 | -0.01 | -0.05 | 0.05 | 0.02 | 0.01 |
| 0.00 | -0.02 | -0.03 | -0.01 | 0.04 | 0.07 | 0.01 |
| 0.02 | -0.02 | -0.01 | 0.03 | -0.06 | 0.02 | -0.01 |
| -0.01 | 0.05 | -0.03 | 0.10 | -0.02 | 0.06 | 0.00 |
| -0.03 | -0.02 | 0.09 | -0.07 | 0.03 | 0.01 | 0.01 |
| 0.12 | 0.03 | 0.01 | 0.00 | 0.02 | 0.03 | 0.03 |
| -0.03 | 0.03 | -0.02 | 0.01 | 0.01 | -0.04 | 0.01 |
| 0.02 | 0.01 | 0.02 | -0.04 | 0.01 | 0.01 | 0.02 |
| -0.03 | 0.04 | -0.02 | 0.05 | 0.01 | 0.00 | 0.01 |
| -0.02 | 0.06 | -0.03 | 0.01 | 0.06 | 0.09 | -0.01 |
| 0.01 | -0.06 | 0.06 | -0.04 | 0.09 | 0.02 | -0.02 |
| 0.03 | -0.03 | 0.05 | 0.04 | -0.03 | 0.04 | 0.00 |
| -0.02 | 0.09 | 0.01 | -0.03 | -0.03 | -0.05 | -0.01 |
| 0.02 | 0.05 | -0.02 | 0.02 | 0.02 | 0.01 | 0.00 |
| -0.06 | -0.01 | -0.01 | 0.02 | -0.04 | -0.01 | 0.02 |
| 0.12 | 0.09 | -0.05 | 0.04 | 0.01 | 0.07 | 0.04 |
| 0.08 | 0.07 | -0.01 | -0.05 | -0.10 | -0.02 | -0.02 |
| 0.09 | 0.03 | -0.04 | -0.03 | 0.03 | -0.07 | -0.02 |
| 0.06 | -0.16 | 0.01 | 0.01 | 0.10 | -0.05 | -0.03 |
| 0.05 | -0.01 | -0.05 | 0.08 | 0.05 | 0.01 | 0.01 |
| 0.02 | 0.03 | -0.01 | 0.01 | -0.01 | 0.04 | -0.02 |
| -0.01 | -0.04 | 0.02 | 0.02 | 0.03 | 0.05 | 0.02 |
| 0.05 | 0.00 | -0.04 | 0.01 | 0.03 | 0.00 | 0.00 |
| 0.02 | 0.05 | -0.02 | -0.02 | -0.01 | -0.02 | 0.02 |
| -0.01 | 0.08 | 0.03 | 0.00 | 0.05 | -0.02 | 0.01 |

| | | | | | | |
|---|---|---|---|---|---|---|
| -0.02 | 0.07 | 0.06 | 0.01 | 0.02 | -0.03 | -0.01 |
| -0.02 | 0.03 | 0.00 | 0.01 | 0.04 | -0.01 | 0.01 |
| 0.01 | -0.05 | -0.04 | 0.02 | -0.10 | 0.06 | -0.01 |
| -0.03 | 0.02 | -0.03 | -0.04 | 0.02 | 0.08 | -0.01 |
| 0.05 | 0.05 | 0.07 | 0.04 | -0.04 | 0.04 | 0.03 |
| 0.01 | 0.00 | -0.03 | 0.04 | 0.00 | 0.02 | 0.02 |
| -0.11 | -0.05 | -0.03 | 0.00 | 0.04 | 0.05 | 0.03 |
| -0.03 | 0.03 | -0.03 | 0.07 | 0.06 | 0.02 | -0.03 |
| -0.08 | 0.03 | 0.01 | 0.00 | -0.06 | 0.06 | 0.03 |
| 0.03 | -0.04 | -0.04 | -0.04 | -0.02 | 0.03 | -0.02 |
| -0.03 | 0.14 | 0.01 | -0.09 | -0.12 | -0.06 | 0.04 |
| 0.06 | 0.02 | 0.05 | -0.05 | 0.02 | -0.02 | 0.02 |
| 0.03 | -0.06 | -0.07 | -0.03 | 0.02 | -0.01 | -0.02 |
| 0.10 | -0.02 | 0.08 | 0.08 | -0.04 | 0.01 | 0.02 |
| 0.04 | -0.01 | 0.01 | 0.04 | -0.01 | 0.09 | -0.02 |
| -0.05 | -0.06 | 0.05 | -0.01 | -0.01 | 0.04 | -0.01 |
| 0.00 | 0.00 | -0.03 | -0.08 | -0.02 | -0.06 | 0.01 |
| -0.01 | -0.03 | -0.02 | 0.03 | 0.01 | -0.06 | 0.03 |
| -0.02 | 0.00 | 0.01 | -0.09 | 0.10 | -0.02 | 0.00 |
| -0.03 | 0.02 | -0.02 | 0.08 | 0.03 | 0.09 | -0.01 |
| -0.03 | -0.01 | -0.03 | 0.01 | 0.01 | -0.09 | 0.01 |
| 0.02 | 0.01 | -0.04 | 0.00 | 0.00 | -0.03 | 0.00 |

| EXPLAINED PCA VARIANCE | PC1 | PC2 | PC3 | PC4 | PC5 | PC6 | PC7 | PC8 | PC9 | PC10 | PC11 | PC12 |
|---|---|---|---|---|---|---|---|---|---|---|---|---|
| Individual | 0.13 | 0.09 | 0.08 | 0.06 | 0.05 | 0.05 | 0.05 | 0.04 | 0.04 | 0.03 | 0.03 | 0.03 |
| Cumulative | 0.13 | 0.22 | 0.29 | 0.36 | 0.41 | 0.46 | 0.51 | 0.55 | 0.58 | 0.62 | 0.65 | 0.68 |

| PC13 | PC14 | PC15 | PC16 | PC17 | PC18 | PC19 | PC20 | PC21 | PC22 | PC23 | PC24 | PC25 |
|---|---|---|---|---|---|---|---|---|---|---|---|---|
| 0.03 | 0.02 | 0.02 | 0.02 | 0.02 | 0.02 | 0.02 | 0.02 | 0.02 | 0.02 | 0.02 | 0.01 | 0.01 |
| 0.70 | 0.73 | 0.75 | 0.78 | 0.80 | 0.82 | 0.84 | 0.85 | 0.87 | 0.89 | 0.90 | 0.92 | 0.93 |

| | | | | | | |
|---|---|---|---|---|---|---|
| PC26 | PC27 | PC28 | PC29 | PC30 | PC31 | PC32 |
| 0.01 | 0.01 | 0.01 | 0.01 | 0.01 | 0.01 | 0.00 |
| 0.94 | 0.96 | 0.97 | 0.98 | 0.99 | 1.00 | 1.00 |

## SUPPLEMENTARY DATASET 2

| MODULE 0 | | | | | |
|---|---|---|---|---|---|
| Pathway | Total | Expected | Hits | P.Value | FDR |
| Translation | 249 | 9.58 | 113 | 5.3E-101 | 7.41E-98 |
| Metabolism of mRNA | 317 | 12.2 | 116 | 1.06E-90 | 7.41E-88 |
| Metabolism of RNA | 339 | 13 | 116 | 9.47E-87 | 4.42E-84 |
| GTP hydrolysis and joining of the 60S ribosomal subunit | 201 | 7.74 | 95 | 1.56E-85 | 5.46E-83 |
| Eukaryotic Translation Initiation | 209 | 8.04 | 96 | 5.57E-85 | 1.30E-82 |
| Cap-dependent Translation Initiation | 209 | 8.04 | 96 | 5.57E-85 | 1.30E-82 |
| 3' -UTR-mediated translational regulation | 201 | 7.74 | 94 | 5.36E-84 | 9.40E-82 |
| L13a-mediated translational silencing of Ceruloplasmin expression | 201 | 7.74 | 94 | 5.36E-84 | 9.40E-82 |
| Formation of a pool of free 40S subunits | 189 | 7.27 | 88 | 5.36E-78 | 8.35E-76 |
| SRP-dependent cotranslational protein targeting to membrane | 204 | 7.85 | 90 | 3.11E-77 | 4.36E-75 |
| Eukaryotic Translation Elongation | 186 | 7.16 | 84 | 8.86E-73 | 1.13E-70 |
| Nonsense Mediated Decay Independent of the Exon Junction Complex | 184 | 7.08 | 83 | 8.46E-72 | 9.89E-70 |
| Nonsense Mediated Decay Enhanced by the Exon Junction Complex | 203 | 7.81 | 86 | 1.18E-71 | 1.18E-69 |
| Nonsense-Mediated Decay | 203 | 7.81 | 86 | 1.18E-71 | 1.18E-69 |
| Peptide chain elongation | 178 | 6.85 | 81 | 2.41E-70 | 2.26E-68 |
| Eukaryotic Translation Termination | 178 | 6.85 | 80 | 7.13E-69 | 6.25E-67 |
| Influenza Viral RNA Transcription and Replication | 176 | 6.77 | 79 | 6.67E-68 | 5.19E-66 |
| Viral mRNA Translation | 176 | 6.77 | 79 | 6.67E-68 | 5.19E-66 |
| Influenza Life Cycle | 180 | 6.93 | 79 | 6.19E-67 | 4.57E-65 |
| Influenza Infection | 185 | 7.12 | 79 | 9.10E-66 | 6.38E-64 |
| Gene Expression | 1090 | 41.8 | 157 | 2.75E-62 | 1.84E-60 |
| Metabolism of proteins | 689 | 26.5 | 113 | 1.69E-46 | 1.08E-44 |
| Ribosomal scanning and start codon recognition | 91 | 3.5 | 48 | 6.35E-45 | 3.87E-43 |
| Translation initiation complex formation | 92 | 3.54 | 48 | 1.29E-44 | 7.52E-43 |
| Activation of the mRNA upon binding of the cap-binding complex and eIFs, and subsequent binding to 43S | 93 | 3.58 | 48 | 2.58E-44 | 1.45E-42 |
| Switching of origins to a post-replicative state | 69 | 2.66 | 42 | 5.91E-43 | 3.07E-41 |
| Orc1 removal from chromatin | 69 | 2.66 | 42 | 5.91E-43 | 3.07E-41 |
| Disease | 945 | 36.4 | 126 | 1.45E-42 | 7.28E-41 |
| Removal of licensing factors from origins | 71 | 2.73 | 42 | 3.39E-42 | 1.58E-40 |

| | | | | | |
|---|---|---|---|---|---|
| Regulation of DNA replication | 71 | 2.73 | 42 | 3.39E-42 | 1.58E-40 |
| Assembly of the pre-replicative complex | 63 | 2.42 | 40 | 5.28E-42 | 2.39E-40 |
| Formation of the ternary complex, and subsequently, the 43S complex | 83 | 3.19 | 44 | 2.75E-41 | 1.21E-39 |
| Cell Cycle Checkpoints | 131 | 5.04 | 52 | 1.33E-40 | 5.64E-39 |
| Regulation of APC/C activators between G1/S and early anaphase | 84 | 3.23 | 42 | 5.46E-38 | 2.25E-36 |
| Activation of APC/C and APC/C:Cdc20 mediated degradation of mitotic proteins | 77 | 2.96 | 40 | 4.60E-37 | 1.84E-35 |
| APC/C-mediated degradation of cell cycle proteins | 89 | 3.43 | 42 | 1.26E-36 | 4.76E-35 |
| Regulation of mitotic cell cycle | 89 | 3.43 | 42 | 1.26E-36 | 4.76E-35 |
| Synthesis of DNA | 95 | 3.66 | 43 | 1.63E-36 | 6.01E-35 |
| DNA Replication Pre-Initiation | 80 | 3.08 | 40 | 3.47E-36 | 1.22E-34 |
| M/G1 Transition | 80 | 3.08 | 40 | 3.47E-36 | 1.22E-34 |
| APC/C:Cdc20 mediated degradation of mitotic proteins | 76 | 2.93 | 39 | 7.25E-36 | 2.48E-34 |
| Antigen processing: Ubiquitination & Proteasome degradation | 224 | 8.62 | 60 | 1.42E-35 | 4.75E-34 |
| G1/S Transition | 113 | 4.35 | 45 | 3.63E-35 | 1.18E-33 |
| DNA Replication | 102 | 3.93 | 43 | 7.03E-35 | 2.24E-33 |
| Cdc20:Phospho-APC/C mediated degradation of Cyclin A | 76 | 2.93 | 38 | 2.17E-34 | 6.52E-33 |
| APC/C:Cdh1 mediated degradation of Cdc20 and other APC/C:Cdh1 targeted proteins in late mitosis/early G1 | 76 | 2.93 | 38 | 2.17E-34 | 6.52E-33 |
| CDT1 association with the CDC6:ORC:origin complex | 57 | 2.19 | 34 | 2.19E-34 | 6.52E-33 |
| APC/C:Cdc20 mediated degradation of Securin | 71 | 2.73 | 36 | 6.92E-33 | 2.02E-31 |
| Class I MHC mediated antigen processing & presentation | 267 | 10.3 | 62 | 7.43E-33 | 2.13E-31 |
| SCF-beta-TrCP mediated degradation of Emi1 | 53 | 2.04 | 32 | 1.22E-32 | 3.42E-31 |
| S Phase | 122 | 4.7 | 44 | 3.54E-32 | 9.74E-31 |
| Vif-mediated degradation of APOBEC3G | 55 | 2.12 | 32 | 6.70E-32 | 1.81E-30 |
| CDK-mediated phosphorylation and removal of Cdc6 | 49 | 1.89 | 30 | 6.63E-31 | 1.75E-29 |
| Autodegradation of Cdh1 by Cdh1:APC/C | 68 | 2.62 | 34 | 8.13E-31 | 2.11E-29 |
| Vpu mediated degradation of CD4 | 50 | 1.92 | 30 | 1.60E-30 | 4.09E-29 |
| Mitotic G1-G1/S phases | 140 | 5.39 | 45 | 2.21E-30 | 5.54E-29 |
| Ubiquitin Mediated Degradation of Phosphorylated Cdc25A | 52 | 2 | 30 | 8.60E-30 | 2.04E-28 |
| p53-Independent DNA Damage Response | 52 | 2 | 30 | 8.60E-30 | 2.04E-28 |
| p53-Independent G1/S DNA damage checkpoint | 52 | 2 | 30 | 8.60E-30 | 2.04E-28 |
| Regulation of activated PAK-2p34 by proteasome mediated degradation | 48 | 1.85 | 29 | 1.18E-29 | 2.76E-28 |

| | | | | | |
|---|---|---|---|---|---|
| G1/S DNA Damage Checkpoints | 62 | 2.39 | 32 | 1.28E-29 | 2.94E-28 |
| Autodegradation of the E3 ubiquitin ligase COP1 | 53 | 2.04 | 30 | 1.92E-29 | 4.33E-28 |
| Ubiquitin-dependent degradation of Cyclin D1 | 49 | 1.89 | 29 | 2.80E-29 | 6.13E-28 |
| Ubiquitin-dependent degradation of Cyclin D | 49 | 1.89 | 29 | 2.80E-29 | 6.13E-28 |
| Stabilization of p53 | 54 | 2.08 | 30 | 4.17E-29 | 8.86E-28 |
| Destabilization of mRNA by AUF1 (hnRNP D0) | 54 | 2.08 | 30 | 4.17E-29 | 8.86E-28 |
| p53-Dependent G1/S DNA damage checkpoint | 59 | 2.27 | 31 | 4.92E-29 | 1.02E-27 |
| p53-Dependent G1 DNA Damage Response | 59 | 2.27 | 31 | 4.92E-29 | 1.02E-27 |
| Degradation of beta-catenin by the destruction complex | 65 | 2.5 | 32 | 9.27E-29 | 1.86E-27 |
| Signaling by Wnt | 65 | 2.5 | 32 | 9.27E-29 | 1.86E-27 |
| Activation of NF-kappaB in B Cells | 66 | 2.54 | 32 | 1.74E-28 | 3.44E-27 |
| ER-Phagosome pathway | 63 | 2.42 | 31 | 7.13E-28 | 1.39E-26 |
| Cross-presentation of soluble exogenous antigens (endosomes) | 48 | 1.85 | 27 | 1.78E-26 | 3.43E-25 |
| Mitotic M-M/G1 phases | 266 | 10.2 | 55 | 2.56E-26 | 4.86E-25 |
| Regulation of ornithine decarboxylase (ODC) | 49 | 1.89 | 27 | 3.84E-26 | 7.18E-25 |
| Regulation of Apoptosis | 59 | 2.27 | 29 | 4.17E-26 | 7.70E-25 |
| Cyclin A:Cdk2-associated events at S phase entry | 66 | 2.54 | 30 | 1.09E-25 | 1.99E-24 |
| Antigen processing-Cross presentation | 78 | 3 | 32 | 1.30E-25 | 2.33E-24 |
| SCF(Skp2)-mediated degradation of p27/p21 | 58 | 2.23 | 28 | 5.94E-25 | 1.05E-23 |
| Cyclin E associated events during G1/S transition | 65 | 2.5 | 29 | 1.44E-24 | 2.53E-23 |
| Separation of Sister Chromatids | 186 | 7.16 | 42 | 1.16E-21 | 2.00E-20 |
| Regulation of mRNA Stability by Proteins that Bind AU-rich Elements | 88 | 3.39 | 30 | 2.81E-21 | 4.80E-20 |
| Mitotic Anaphase | 198 | 7.62 | 42 | 1.50E-20 | 2.53E-19 |
| Mitotic Metaphase and Anaphase | 199 | 7.66 | 42 | 1.84E-20 | 3.07E-19 |
| Cell Cycle, Mitotic | 411 | 15.8 | 60 | 1.90E-20 | 3.14E-19 |
| M Phase | 233 | 8.97 | 44 | 2.10E-19 | 3.43E-18 |
| Host Interactions of HIV factors | 141 | 5.43 | 33 | 1.16E-17 | 1.88E-16 |
| Cell Cycle | 508 | 19.6 | 61 | 1.77E-16 | 2.82E-15 |
| Downstream Signaling Events Of B Cell Receptor (BCR) | 173 | 6.66 | 33 | 7.57E-15 | 1.19E-13 |
| Adaptive Immune System | 654 | 25.2 | 67 | 1.47E-14 | 2.29E-13 |
| Signaling by the B Cell Receptor (BCR) | 199 | 7.66 | 34 | 8.69E-14 | 1.34E-12 |

| | | | | | |
|---|---|---|---|---|---|
| HIV Infection | 214 | 8.24 | 35 | 1.41E-13 | 2.15E-12 |
| Apoptosis | 158 | 6.08 | 30 | 1.59E-13 | 2.39E-12 |
| Immune System | 1140 | 43.8 | 85 | 1.28E-10 | 1.91E-09 |
| Downregulation of SMAD2/3:SMAD4 transcriptional activity | 23 | 0.885 | 11 | 2.00E-10 | 2.94E-09 |
| Metabolism of amino acids and derivatives | 190 | 7.31 | 27 | 2.75E-09 | 4.02E-08 |
| APC/C:Cdc20 mediated degradation of Cyclin B | 29 | 1.12 | 11 | 4.15E-09 | 6.00E-08 |
| Activation of the pre-replicative complex | 32 | 1.23 | 11 | 1.40E-08 | 2.00E-07 |
| G2/M Checkpoints | 48 | 1.85 | 13 | 1.74E-08 | 2.47E-07 |
| Oxygen-dependent Proline Hydroxylation of Hypoxia-inducible Factor Alpha | 20 | 0.77 | 9 | 1.87E-08 | 2.62E-07 |
| Unwinding of DNA | 11 | 0.423 | 7 | 3.34E-08 | 4.64E-07 |
| E2F mediated regulation of DNA replication | 35 | 1.35 | 11 | 4.08E-08 | 5.60E-07 |
| NOD1/2 Signaling Pathway | 38 | 1.46 | 11 | 1.06E-07 | 1.44E-06 |
| APC-Cdc20 mediated degradation of Nek2A | 31 | 1.19 | 10 | 1.30E-07 | 1.75E-06 |
| TRIF-mediated TLR3/TLR4 signaling | 87 | 3.35 | 16 | 1.48E-07 | 1.98E-06 |
| MyD88-independent cascade | 88 | 3.39 | 16 | 1.75E-07 | 2.30E-06 |
| Toll Like Receptor 3 (TLR3) Cascade | 88 | 3.39 | 16 | 1.75E-07 | 2.30E-06 |
| Phosphorylation of the APC/C | 25 | 0.962 | 9 | 1.92E-07 | 2.49E-06 |
| Activation of ATR in response to replication stress | 41 | 1.58 | 11 | 2.51E-07 | 3.23E-06 |
| Negative regulators of RIG-I/MDA5 signaling | 33 | 1.27 | 10 | 2.54E-07 | 3.23E-06 |
| Cellular responses to stress | 27 | 1.04 | 9 | 4.11E-07 | 5.10E-06 |
| Cellular response to hypoxia | 27 | 1.04 | 9 | 4.11E-07 | 5.10E-06 |
| Regulation of Hypoxia-inducible Factor (HIF) by Oxygen | 27 | 1.04 | 9 | 4.11E-07 | 5.10E-06 |
| Transcriptional activity of SMAD2/SMAD3:SMAD4 heterotrimer | 43 | 1.66 | 11 | 4.27E-07 | 5.25E-06 |
| Phosphorylation of Emi1 | 6 | 0.231 | 5 | 4.73E-07 | 5.76E-06 |
| E2F-enabled inhibition of pre-replication complex formation | 10 | 0.385 | 6 | 5.66E-07 | 6.84E-06 |
| Endosomal Sorting Complex Required For Transport (ESCRT) | 28 | 1.08 | 9 | 5.86E-07 | 7.02E-06 |
| TAK1 activates NFkB by phosphorylation and activation of IKKs complex | 22 | 0.847 | 8 | 8.65E-07 | 1.03E-05 |
| Activated TLR4 signalling | 100 | 3.85 | 16 | 1.07E-06 | 1.27E-05 |
| RIG-I/MDA5 mediated induction of IFN-alpha/beta pathways | 67 | 2.58 | 13 | 1.19E-06 | 1.39E-05 |

| MODULE 1 | | | | | |
|---|---|---|---|---|---|
| Pathway | Total | Expected | Hits | P.Value | FDR |
| Rho GTPase cycle | 123 | 2.62 | 61 | 1.10E-73 | 7.70E-71 |
| Signaling by Rho GTPases | 123 | 2.62 | 61 | 1.10E-73 | 7.70E-71 |
| G alpha (12/13) signalling events | 80 | 1.7 | 29 | 3.51E-29 | 1.64E-26 |
| NRAGE signals death through JNK | 45 | 0.957 | 23 | 1.53E-27 | 5.36E-25 |
| Signal Transduction | 1690 | 35.9 | 96 | 9.56E-27 | 2.68E-24 |
| p75 NTR receptor-mediated signalling | 85 | 1.81 | 26 | 5.61E-24 | 1.31E-21 |
| Cell death signalling via NRAGE, NRIF and NADE | 62 | 1.32 | 23 | 1.65E-23 | 3.31E-21 |
| Signalling by NGF | 290 | 6.17 | 38 | 1.47E-20 | 2.58E-18 |
| Axon guidance | 292 | 6.21 | 32 | 5.28E-15 | 8.23E-13 |
| Sema4D in semaphorin signaling | 34 | 0.723 | 13 | 6.63E-14 | 9.29E-12 |
| Sema4D induced cell migration and growth-cone collapse | 29 | 0.617 | 12 | 2.04E-13 | 2.60E-11 |
| Semaphorin interactions | 72 | 1.53 | 16 | 1.11E-12 | 1.30E-10 |
| Signaling by Robo receptor | 34 | 0.723 | 12 | 1.97E-12 | 2.13E-10 |
| Developmental Biology | 417 | 8.87 | 33 | 2.04E-11 | 2.04E-09 |
| GPVI-mediated activation cascade | 33 | 0.702 | 11 | 3.54E-11 | 3.31E-09 |
| Activation of Rac | 15 | 0.319 | 8 | 1.95E-10 | 1.71E-08 |
| Fcgamma receptor (FCGR) dependent phagocytosis | 86 | 1.83 | 13 | 2.42E-08 | 1.99E-06 |
| Smooth Muscle Contraction | 25 | 0.532 | 8 | 2.74E-08 | 2.13E-06 |
| CD28 co-stimulation | 30 | 0.638 | 8 | 1.35E-07 | 9.99E-06 |
| Hemostasis | 511 | 10.9 | 29 | 7.36E-07 | 5.16E-05 |
| Platelet activation, signaling and aggregation | 220 | 4.68 | 18 | 7.80E-07 | 5.21E-05 |
| Interleukin-3, 5 and GM-CSF signaling | 51 | 1.08 | 9 | 9.83E-07 | 6.26E-05 |
| GPCR downstream signaling | 812 | 17.3 | 38 | 1.36E-06 | 8.31E-05 |
| CD28 dependent Vav1 pathway | 12 | 0.255 | 5 | 2.85E-06 | 0.000166 |
| Regulation of signaling by CBL | 22 | 0.468 | 6 | 4.69E-06 | 0.000263 |
| Regulation of actin dynamics for phagocytic cup formation | 62 | 1.32 | 9 | 5.37E-06 | 0.00029 |
| Signaling by GPCR | 931 | 19.8 | 40 | 5.82E-06 | 0.000302 |
| DCC mediated attractive signaling | 14 | 0.298 | 5 | 6.96E-06 | 0.000348 |
| Downstream signal transduction | 163 | 3.47 | 14 | 8.17E-06 | 0.000395 |

| | | | | | |
|---|---|---|---|---|---|
| DAP12 signaling | 164 | 3.49 | 14 | 8.77E-06 | 0.00041 |
| Muscle contraction | 52 | 1.11 | 8 | 1.17E-05 | 0.00053 |
| DAP12 interactions | 182 | 3.87 | 14 | 2.88E-05 | 0.00126 |
| NGF signalling via TRKA from the plasma membrane | 207 | 4.4 | 15 | 2.99E-05 | 0.00127 |
| Signaling by PDGF | 189 | 4.02 | 14 | 4.38E-05 | 0.00181 |
| Costimulation by the CD28 family | 68 | 1.45 | 8 | 8.65E-05 | 0.00346 |
| Signaling by FGFR in disease | 178 | 3.79 | 13 | 9.61E-05 | 0.00374 |
| Signaling by EGFR | 179 | 3.81 | 13 | 0.000102 | 0.00385 |
| Signaling by EGFR in Cancer | 181 | 3.85 | 13 | 0.000114 | 0.0042 |
| Signaling by FGFR | 162 | 3.45 | 12 | 0.000157 | 0.00566 |
| Signaling by ERBB2 | 164 | 3.49 | 12 | 0.000177 | 0.0062 |
| Cell-Cell communication | 143 | 3.04 | 11 | 0.000214 | 0.00732 |
| Innate Immune System | 521 | 11.1 | 24 | 0.000227 | 0.00758 |
| Interleukin-2 signaling | 42 | 0.893 | 6 | 0.000232 | 0.00758 |
| Interleukin receptor SHC signaling | 28 | 0.595 | 5 | 0.000269 | 0.00856 |
| Downstream signaling of activated FGFR | 150 | 3.19 | 11 | 0.000325 | 0.0101 |
| TGF-beta receptor signaling in EMT (epithelial to mesenchymal transition) | 17 | 0.362 | 4 | 0.000376 | 0.0115 |
| Regulation of PAK-2p34 activity by PS-GAP/RHG10 | 2 | 0.0425 | 2 | 0.000449 | 0.0134 |
| Nef and signal transduction | 8 | 0.17 | 3 | 0.000488 | 0.014 |
| Sema4D mediated inhibition of cell attachment and migration | 8 | 0.17 | 3 | 0.000488 | 0.014 |
| Antigen Activates B Cell Receptor Leading to Generation of Second Messengers | 32 | 0.681 | 5 | 0.000514 | 0.0144 |
| Netrin-1 signaling | 49 | 1.04 | 6 | 0.000548 | 0.0151 |
| Synthesis of PIPs at the plasma membrane | 33 | 0.702 | 5 | 0.000595 | 0.0161 |
| Axonal growth inhibition (RHOA activation) | 9 | 0.191 | 3 | 0.00072 | 0.0191 |
| Signaling by Interleukins | 116 | 2.47 | 9 | 0.000764 | 0.0198 |
| Signaling by SCF-KIT | 142 | 3.02 | 10 | 0.000837 | 0.0213 |
| p75NTR regulates axonogenesis | 10 | 0.213 | 3 | 0.00101 | 0.0249 |
| Inactivation of Cdc42 and Rac | 10 | 0.213 | 3 | 0.00101 | 0.0249 |
| PLCG1 events in ERBB2 signaling | 38 | 0.808 | 5 | 0.00116 | 0.028 |
| Factors involved in megakaryocyte development and platelet production | 155 | 3.3 | 10 | 0.00164 | 0.0389 |
| GAB1 signalosome | 106 | 2.25 | 8 | 0.00179 | 0.0412 |

| | | | | | |
|---|---|---|---|---|---|
| PI3K/AKT activation | 106 | 2.25 | 8 | 0.00179 | 0.0412 |
| Integrin alphaIIb beta3 signaling | 27 | 0.574 | 4 | 0.00235 | 0.0532 |
| Constitutive PI3K/AKT Signaling in Cancer | 89 | 1.89 | 7 | 0.00273 | 0.0608 |
| G-protein beta:gamma signalling | 30 | 0.638 | 4 | 0.0035 | 0.0766 |
| FCGR activation | 15 | 0.319 | 3 | 0.00355 | 0.0766 |
| Striated Muscle Contraction | 31 | 0.659 | 4 | 0.00395 | 0.0833 |
| PI Metabolism | 50 | 1.06 | 5 | 0.00398 | 0.0833 |
| Sema3A PAK dependent Axon repulsion | 16 | 0.34 | 3 | 0.0043 | 0.0875 |
| Disinhibition of SNARE formation | 5 | 0.106 | 2 | 0.00431 | 0.0875 |
| Signaling by ERBB4 | 152 | 3.23 | 9 | 0.00493 | 0.0988 |
| Regulation of KIT signaling | 17 | 0.362 | 3 | 0.00515 | 0.102 |
| PI3K events in ERBB4 signaling | 103 | 2.19 | 7 | 0.00615 | 0.113 |
| PIP3 activates AKT signaling | 103 | 2.19 | 7 | 0.00615 | 0.113 |
| PI-3K cascade | 103 | 2.19 | 7 | 0.00615 | 0.113 |
| PI3K/AKT Signaling in Cancer | 103 | 2.19 | 7 | 0.00615 | 0.113 |
| PI3K events in ERBB2 signaling | 103 | 2.19 | 7 | 0.00615 | 0.113 |
| Regulation of PLK1 Activity at G2/M Transition | 6 | 0.128 | 2 | 0.00637 | 0.116 |
| Platelet Aggregation (Plug Formation) | 36 | 0.766 | 4 | 0.00681 | 0.122 |
| Signaling by constitutively active EGFR | 19 | 0.404 | 3 | 0.00711 | 0.125 |
| CD28 dependent PI3K/Akt signaling | 19 | 0.404 | 3 | 0.00711 | 0.125 |
| Signalling to ERKs | 37 | 0.787 | 4 | 0.00751 | 0.128 |
| EGFR interacts with phospholipase C-gamma | 37 | 0.787 | 4 | 0.00751 | 0.128 |
| G alpha (z) signalling events | 38 | 0.808 | 4 | 0.00826 | 0.14 |
| Cell-cell junction organization | 60 | 1.28 | 5 | 0.00867 | 0.145 |
| Signaling by the B Cell Receptor (BCR) | 199 | 4.23 | 10 | 0.00965 | 0.159 |
| Cell junction organization | 89 | 1.89 | 6 | 0.0114 | 0.186 |
| GRB2 events in ERBB2 signaling | 23 | 0.489 | 3 | 0.0122 | 0.197 |
| CTLA4 inhibitory signaling | 24 | 0.51 | 3 | 0.0137 | 0.217 |
| Nephrin interactions | 24 | 0.51 | 3 | 0.0137 | 0.217 |
| EGFR Transactivation by Gastrin | 9 | 0.191 | 2 | 0.0147 | 0.226 |
| eNOS activation | 9 | 0.191 | 2 | 0.0147 | 0.226 |

| | | | | | |
|---|---|---|---|---|---|
| SHC1 events in ERBB2 signaling | 25 | 0.532 | 3 | 0.0154 | 0.234 |
| Signaling by TGF-beta Receptor Complex | 70 | 1.49 | 5 | 0.0162 | 0.245 |
| COPI Mediated Transport | 10 | 0.213 | 2 | 0.0181 | 0.264 |
| Golgi to ER Retrograde Transport | 10 | 0.213 | 2 | 0.0181 | 0.264 |
| GP1b-IX-V activation signalling | 10 | 0.213 | 2 | 0.0181 | 0.264 |
| Signalling to RAS | 27 | 0.574 | 3 | 0.019 | 0.269 |
| G beta:gamma signalling through PI3Kgamma | 27 | 0.574 | 3 | 0.019 | 0.269 |
| Role of phospholipids in phagocytosis | 27 | 0.574 | 3 | 0.019 | 0.269 |
| mTOR signalling | 29 | 0.617 | 3 | 0.023 | 0.319 |
| Tight junction interactions | 29 | 0.617 | 3 | 0.023 | 0.319 |
| PKB-mediated events | 30 | 0.638 | 3 | 0.0251 | 0.346 |
| Netrin mediated repulsion signals | 12 | 0.255 | 2 | 0.0258 | 0.351 |
| The role of Nef in HIV-1 replication and disease pathogenesis | 31 | 0.659 | 3 | 0.0274 | 0.363 |
| Trafficking of AMPA receptors | 31 | 0.659 | 3 | 0.0274 | 0.363 |
| Glutamate Binding, Activation of AMPA Receptors and Synaptic Plasticity | 31 | 0.659 | 3 | 0.0274 | 0.363 |
| p38MAPK events | 13 | 0.276 | 2 | 0.03 | 0.386 |
| PECAM1 interactions | 13 | 0.276 | 2 | 0.03 | 0.386 |
| Downregulation of ERBB2:ERBB3 signaling | 13 | 0.276 | 2 | 0.03 | 0.386 |
| Downstream Signaling Events Of B Cell Receptor (BCR) | 173 | 3.68 | 8 | 0.0307 | 0.392 |
| Gastrin-CREB signalling pathway via PKC and MAPK | 209 | 4.44 | 9 | 0.0336 | 0.425 |
| Immune System | 1140 | 24.2 | 33 | 0.0342 | 0.429 |
| GRB2 events in EGFR signaling | 14 | 0.298 | 2 | 0.0346 | 0.429 |
| IRS-mediated signalling | 87 | 1.85 | 5 | 0.0373 | 0.454 |
| Signal transduction by L1 | 35 | 0.744 | 3 | 0.0375 | 0.454 |
| DAG and IP3 signaling | 35 | 0.744 | 3 | 0.0375 | 0.454 |
| SHC1 events in EGFR signaling | 15 | 0.319 | 2 | 0.0393 | 0.467 |
| GRB2:SOS provides linkage to MAPK signaling for Intergrins | 15 | 0.319 | 2 | 0.0393 | 0.467 |
| Activation, translocation and oligomerization of BAX | 2 | 0.0425 | 1 | 0.0421 | 0.485 |
| Activation and oligomerization of BAK protein | 2 | 0.0425 | 1 | 0.0421 | 0.485 |

| MODULE 2 | | | | | |
|---|---|---|---|---|---|
| Pathway | Total | Expected | Hits | P.Value | FDR |
| Unfolded Protein Response | 66 | 0.751 | 9 | 4.34E-08 | 6.09E-05 |
| Activation of Chaperones by IRE1alpha | 49 | 0.558 | 6 | 1.69E-05 | 0.0118 |
| Mitochondrial Protein Import | 59 | 0.672 | 6 | 4.97E-05 | 0.0135 |
| Inflammasomes | 19 | 0.216 | 4 | 5.28E-05 | 0.0135 |
| Innate Immune System | 521 | 5.93 | 17 | 5.66E-05 | 0.0135 |
| Immune System | 1140 | 13 | 27 | 7.45E-05 | 0.0135 |
| Activation of Chaperones by ATF6-alpha | 8 | 0.0911 | 3 | 7.62E-05 | 0.0135 |
| Signaling by ERBB2 | 164 | 1.87 | 9 | 9.08E-05 | 0.0135 |
| Interferon alpha/beta signaling | 68 | 0.774 | 6 | 0.000111 | 0.0135 |
| G2/M Transition | 102 | 1.16 | 7 | 0.000146 | 0.0135 |
| PI3K events in ERBB4 signaling | 103 | 1.17 | 7 | 0.000155 | 0.0135 |
| PIP3 activates AKT signaling | 103 | 1.17 | 7 | 0.000155 | 0.0135 |
| PI-3K cascade | 103 | 1.17 | 7 | 0.000155 | 0.0135 |
| PI3K/AKT Signaling in Cancer | 103 | 1.17 | 7 | 0.000155 | 0.0135 |
| PI3K events in ERBB2 signaling | 103 | 1.17 | 7 | 0.000155 | 0.0135 |
| Mitotic G2-G2/M phases | 105 | 1.2 | 7 | 0.000175 | 0.0135 |
| GAB1 signalosome | 106 | 1.21 | 7 | 0.000185 | 0.0135 |
| PI3K/AKT activation | 106 | 1.21 | 7 | 0.000185 | 0.0135 |
| Signaling by SCF-KIT | 142 | 1.62 | 8 | 0.000191 | 0.0135 |
| Signaling by EGFR in Cancer | 181 | 2.06 | 9 | 0.000193 | 0.0135 |
| Signaling by ERBB4 | 152 | 1.73 | 8 | 0.000305 | 0.0204 |
| Nucleotide-binding domain, leucine rich repeat containing receptor (NLR) signaling pathways | 55 | 0.626 | 5 | 0.000373 | 0.0238 |
| The NLRP3 inflammasome | 14 | 0.159 | 3 | 0.000471 | 0.0287 |
| Cell Cycle | 508 | 5.78 | 15 | 0.000497 | 0.029 |
| Sema3A PAK dependent Axon repulsion | 16 | 0.182 | 3 | 0.000713 | 0.0391 |
| Downstream Signaling Events Of B Cell Receptor (BCR) | 173 | 1.97 | 8 | 0.000726 | 0.0391 |
| Polo-like kinase mediated events | 4 | 0.0455 | 2 | 0.000756 | 0.0393 |
| Trafficking and processing of endosomal TLR | 17 | 0.194 | 3 | 0.000859 | 0.043 |
| Activation of Chaperone Genes by ATF6-alpha | 5 | 0.0569 | 2 | 0.00125 | 0.0605 |

| | | | | | |
|---|---|---|---|---|---|
| Cytokine Signaling in Immune system | 286 | 3.26 | 10 | 0.00138 | 0.0646 |
| Downstream signaling of activated FGFR | 150 | 1.71 | 7 | 0.0015 | 0.0679 |
| Metabolism of proteins | 689 | 7.84 | 17 | 0.00155 | 0.0681 |
| Activation of Chaperone Genes by XBP1(S) | 46 | 0.524 | 4 | 0.00176 | 0.074 |
| Signaling by the B Cell Receptor (BCR) | 199 | 2.27 | 8 | 0.00179 | 0.074 |
| Activation of PKB | 6 | 0.0683 | 2 | 0.00186 | 0.0746 |
| Cell Cycle, Mitotic | 411 | 4.68 | 12 | 0.00214 | 0.0834 |
| Signaling by FGFR | 162 | 1.84 | 7 | 0.00233 | 0.0884 |
| Downstream signal transduction | 163 | 1.86 | 7 | 0.00241 | 0.0891 |
| DAP12 signaling | 164 | 1.87 | 7 | 0.0025 | 0.0899 |
| Post-transcriptional Silencing By Small RNAs | 7 | 0.0797 | 2 | 0.00259 | 0.0907 |
| Centrosome maturation | 86 | 0.979 | 5 | 0.00284 | 0.0949 |
| Recruitment of mitotic centrosome proteins and complexes | 86 | 0.979 | 5 | 0.00284 | 0.0949 |
| Constitutive PI3K/AKT Signaling in Cancer | 89 | 1.01 | 5 | 0.0033 | 0.107 |
| Interferon Signaling | 173 | 1.97 | 7 | 0.00337 | 0.107 |
| IRS-related events | 90 | 1.02 | 5 | 0.00346 | 0.108 |
| Signaling by FGFR in disease | 178 | 2.03 | 7 | 0.00395 | 0.12 |
| Signaling by EGFR | 179 | 2.04 | 7 | 0.00407 | 0.121 |
| eNOS activation | 9 | 0.102 | 2 | 0.00437 | 0.123 |
| Small Interfering RNA (siRNA) Biogenesis | 9 | 0.102 | 2 | 0.00437 | 0.123 |
| Insulin receptor signalling cascade | 95 | 1.08 | 5 | 0.00437 | 0.123 |
| DAP12 interactions | 182 | 2.07 | 7 | 0.00446 | 0.123 |
| Signaling by PDGF | 189 | 2.15 | 7 | 0.00548 | 0.148 |
| Loss of proteins required for interphase microtubule organization√Ç¬†from the centrosome | 65 | 0.74 | 4 | 0.00621 | 0.161 |
| Loss of Nlp from mitotic centrosomes | 65 | 0.74 | 4 | 0.00621 | 0.161 |
| Pre-NOTCH Transcription and Translation | 12 | 0.137 | 2 | 0.00784 | 0.2 |
| Intrinsic Pathway for Apoptosis | 37 | 0.421 | 3 | 0.00834 | 0.208 |
| NGF signalling via TRKA from the plasma membrane | 207 | 2.36 | 7 | 0.00889 | 0.208 |
| DAI mediated induction of type I IFNs | 13 | 0.148 | 2 | 0.0092 | 0.208 |
| AKT phosphorylates targets in the cytosol | 13 | 0.148 | 2 | 0.0092 | 0.208 |
| PERK regulated gene expression | 13 | 0.148 | 2 | 0.0092 | 0.208 |

| | | | | | |
|---|---|---|---|---|---|
| Downregulation of ERBB2:ERBB3 signaling | 13 | 0.148 | 2 | 0.0092 | 0.208 |
| Tetrahydrobiopterin (BH4) synthesis, recycling, salvage and regulation | 13 | 0.148 | 2 | 0.0092 | 0.208 |
| Signaling by Insulin receptor | 117 | 1.33 | 5 | 0.0104 | 0.231 |
| Cyclin A/B1 associated events during G2/M transition | 14 | 0.159 | 2 | 0.0106 | 0.231 |
| PI3K Cascade | 76 | 0.865 | 4 | 0.0107 | 0.231 |
| t(4;14) translocations of FGFR3 | 1 | 0.0114 | 1 | 0.0114 | 0.242 |
| Toll-Like Receptors Cascades | 123 | 1.4 | 5 | 0.0128 | 0.267 |
| Interleukin-1 signaling | 45 | 0.512 | 3 | 0.0143 | 0.294 |
| Activation of BAD and translocation to mitochondria | 17 | 0.194 | 2 | 0.0156 | 0.312 |
| TRAF6 mediated IRF7 activation in TLR7/8 or 9 signaling | 17 | 0.194 | 2 | 0.0156 | 0.312 |
| IRS-mediated signalling | 87 | 0.99 | 4 | 0.0169 | 0.334 |
| GABA synthesis, release, reuptake and degradation | 18 | 0.205 | 2 | 0.0174 | 0.339 |
| Cytosolic sensors of pathogen-associated DNA | 19 | 0.216 | 2 | 0.0193 | 0.361 |
| Signaling by constitutively active EGFR | 19 | 0.216 | 2 | 0.0193 | 0.361 |
| CD28 dependent PI3K/Akt signaling | 19 | 0.216 | 2 | 0.0193 | 0.361 |
| IRS-related events triggered by IGF1R | 91 | 1.04 | 4 | 0.0197 | 0.363 |
| Metabolism of nitric oxide | 20 | 0.228 | 2 | 0.0213 | 0.378 |
| eNOS activation and regulation | 20 | 0.228 | 2 | 0.0213 | 0.378 |
| Signaling by Type 1 Insulin-like Growth Factor 1 Receptor (IGF1R) | 94 | 1.07 | 4 | 0.0219 | 0.378 |
| IGF1R signaling cascade | 94 | 1.07 | 4 | 0.0219 | 0.378 |
| PDE3B signalling | 2 | 0.0228 | 1 | 0.0226 | 0.378 |
| Inhibition of HSL | 2 | 0.0228 | 1 | 0.0226 | 0.378 |
| Telomere Extension By Telomerase | 2 | 0.0228 | 1 | 0.0226 | 0.378 |
| NOSIP mediated eNOS trafficking | 2 | 0.0228 | 1 | 0.0226 | 0.378 |
| Pre-NOTCH Expression and Processing | 22 | 0.25 | 2 | 0.0255 | 0.421 |
| Activation of BH3-only proteins | 24 | 0.273 | 2 | 0.03 | 0.478 |
| Cytosolic tRNA aminoacylation | 24 | 0.273 | 2 | 0.03 | 0.478 |
| CTLA4 inhibitory signaling | 24 | 0.273 | 2 | 0.03 | 0.478 |
| MicroRNA (miRNA) Biogenesis | 25 | 0.285 | 2 | 0.0324 | 0.504 |
| IRF3 mediated activation of type 1 IFN | 3 | 0.0341 | 1 | 0.0338 | 0.504 |
| Formation of apoptosome | 3 | 0.0341 | 1 | 0.0338 | 0.504 |

| | | | | | |
|---|---|---|---|---|---|
| Inhibition of TSC complex formation by PKB | 3 | 0.0341 | 1 | 0.0338 | 0.504 |
| The NLRP1 inflammasome | 3 | 0.0341 | 1 | 0.0338 | 0.504 |
| Localization of the PINCH-ILK-PARVIN complex to focal adhesions | 3 | 0.0341 | 1 | 0.0338 | 0.504 |
| Integrin alphaIIb beta3 signaling | 27 | 0.307 | 2 | 0.0374 | 0.535 |
| G beta:gamma signalling through PI3Kgamma | 27 | 0.307 | 2 | 0.0374 | 0.535 |
| Nitric oxide stimulates guanylate cyclase | 27 | 0.307 | 2 | 0.0374 | 0.535 |
| Cyclin E associated events during G1/S transition | 65 | 0.74 | 3 | 0.0374 | 0.535 |
| Regulatory RNA pathways | 28 | 0.319 | 2 | 0.0399 | 0.566 |
| RIG-I/MDA5 mediated induction of IFN-alpha/beta pathways | 67 | 0.763 | 3 | 0.0404 | 0.566 |
| Antiviral mechanism by IFN-stimulated genes | 69 | 0.785 | 3 | 0.0435 | 0.594 |
| ISG15 antiviral mechanism | 69 | 0.785 | 3 | 0.0435 | 0.594 |
| G2/M DNA replication checkpoint | 4 | 0.0455 | 1 | 0.0448 | 0.594 |
| SHC activation | 4 | 0.0455 | 1 | 0.0448 | 0.594 |
| AKT-mediated inactivation of FOXO1A | 4 | 0.0455 | 1 | 0.0448 | 0.594 |
| CD28 co-stimulation | 30 | 0.341 | 2 | 0.0453 | 0.594 |
| G-protein beta:gamma signalling | 30 | 0.341 | 2 | 0.0453 | 0.594 |
| Signalling by NGF | 290 | 3.3 | 7 | 0.0461 | 0.598 |
| Semaphorin interactions | 72 | 0.82 | 3 | 0.0483 | 0.622 |

| MODULE 3 | | | | | |
|---|---|---|---|---|---|
| Pathway | Total | Expected | Hits | P.Value | FDR |
| Integrin cell surface interactions | 85 | 1.77 | 37 | 2.73E-41 | 3.83E-38 |
| Hemostasis | 511 | 10.6 | 54 | 1.34E-25 | 9.37E-23 |
| Platelet degranulation | 89 | 1.85 | 25 | 2.77E-22 | 1.29E-19 |
| Platelet activation, signaling and aggregation | 220 | 4.58 | 35 | 4.26E-22 | 1.49E-19 |
| Response to elevated platelet cytosolic Ca2+ | 94 | 1.96 | 25 | 1.24E-21 | 3.49E-19 |
| Regulation of Insulin-like Growth Factor (IGF) Transport and Uptake by Insulin-like Growth Factor Binding Proteins (IGFBPs) | 21 | 0.437 | 13 | 1.38E-17 | 3.23E-15 |
| Extracellular matrix organization | 157 | 3.27 | 25 | 7.12E-16 | 1.43E-13 |
| Cell surface interactions at the vascular wall | 99 | 2.06 | 20 | 6.28E-15 | 1.10E-12 |
| Signal Transduction | 1690 | 35.1 | 74 | 1.03E-12 | 1.60E-10 |
| Integrin alphaIIb beta3 signaling | 27 | 0.562 | 11 | 2.10E-12 | 2.94E-10 |
| Platelet Aggregation (Plug Formation) | 36 | 0.749 | 11 | 8.24E-11 | 1.05E-08 |
| GRB2:SOS provides linkage to MAPK signaling for Intergrins | 15 | 0.312 | 8 | 1.64E-10 | 1.77E-08 |
| p130Cas linkage to MAPK signaling for integrins | 15 | 0.312 | 8 | 1.64E-10 | 1.77E-08 |
| Axon guidance | 292 | 6.08 | 25 | 1.02E-09 | 1.02E-07 |
| Developmental Biology | 417 | 8.68 | 30 | 1.19E-09 | 1.11E-07 |
| Degradation of collagen | 61 | 1.27 | 12 | 3.00E-09 | 2.63E-07 |
| Degradation of the extracellular matrix | 77 | 1.6 | 13 | 4.62E-09 | 3.72E-07 |
| Cell-Cell communication | 143 | 2.98 | 17 | 4.78E-09 | 3.72E-07 |
| Assembly of collagen fibrils and other multimeric structures | 54 | 1.12 | 11 | 9.54E-09 | 7.04E-07 |
| Collagen formation | 85 | 1.77 | 13 | 1.61E-08 | 1.13E-06 |
| Elastic fibre formation | 45 | 0.937 | 10 | 1.89E-08 | 1.26E-06 |
| Molecules associated with elastic fibres | 38 | 0.791 | 9 | 5.53E-08 | 3.52E-06 |
| L1CAM interactions | 112 | 2.33 | 14 | 6.26E-08 | 3.82E-06 |
| Signaling by PDGF | 189 | 3.93 | 17 | 3.11E-07 | 1.82E-05 |
| Signal transduction by L1 | 35 | 0.729 | 8 | 4.22E-07 | 2.37E-05 |
| Signaling by VEGF | 10 | 0.208 | 5 | 8.43E-07 | 4.38E-05 |
| VEGF ligand-receptor interactions | 10 | 0.208 | 5 | 8.43E-07 | 4.38E-05 |
| FasL/ CD95L signaling | 5 | 0.104 | 4 | 8.85E-07 | 4.43E-05 |
| SHC-related events triggered by IGF1R | 18 | 0.375 | 6 | 1.10E-06 | 5.34E-05 |

| | | | | | |
|---|---|---|---|---|---|
| Common Pathway | 11 | 0.229 | 5 | 1.52E-06 | 7.10E-05 |
| Tie2 Signaling | 20 | 0.416 | 6 | 2.23E-06 | 0.000101 |
| Collagen biosynthesis and modifying enzymes | 62 | 1.29 | 9 | 4.51E-06 | 0.000197 |
| Nephrin interactions | 24 | 0.5 | 6 | 7.22E-06 | 0.000307 |
| Fibronectin matrix formation | 3 | 0.0625 | 3 | 8.83E-06 | 0.000364 |
| VEGF binds to VEGFR leading to receptor dimerization | 8 | 0.167 | 4 | 1.18E-05 | 0.00046 |
| CHL1 interactions | 8 | 0.167 | 4 | 1.18E-05 | 0.00046 |
| Signal attenuation | 16 | 0.333 | 5 | 1.32E-05 | 0.000501 |
| Dimerization of procaspase-8 | 9 | 0.187 | 4 | 2.09E-05 | 0.000751 |
| Caspase-8 activation | 9 | 0.187 | 4 | 2.09E-05 | 0.000751 |
| Formation of Fibrin Clot (Clotting Cascade) | 29 | 0.604 | 6 | 2.34E-05 | 0.00082 |
| Death Receptor  Signalling | 12 | 0.25 | 4 | 7.82E-05 | 0.00255 |
| Platelet Adhesion to exposed collagen | 12 | 0.25 | 4 | 7.82E-05 | 0.00255 |
| Extrinsic Pathway for Apoptosis | 12 | 0.25 | 4 | 7.82E-05 | 0.00255 |
| Apoptosis | 158 | 3.29 | 12 | 0.000101 | 0.00322 |
| Caspase-mediated cleavage of cytoskeletal proteins | 13 | 0.271 | 4 | 0.000111 | 0.00346 |
| Signaling by Type 1 Insulin-like Growth Factor 1 Receptor (IGF1R) | 94 | 1.96 | 9 | 0.000134 | 0.00401 |
| IGF1R signaling cascade | 94 | 1.96 | 9 | 0.000134 | 0.00401 |
| DCC mediated attractive signaling | 14 | 0.291 | 4 | 0.000153 | 0.00447 |
| Apoptotic execution  phase | 57 | 1.19 | 7 | 0.000162 | 0.00462 |
| Anchoring fibril formation | 15 | 0.312 | 4 | 0.000205 | 0.00576 |
| Apoptotic cleavage of cellular proteins | 43 | 0.895 | 6 | 0.000236 | 0.00637 |
| Immunoregulatory interactions between a Lymphoid and a non-Lymphoid cell | 80 | 1.67 | 8 | 0.000236 | 0.00637 |
| NGF signalling via TRKA from the plasma membrane | 207 | 4.31 | 13 | 0.000349 | 0.00923 |
| SHC-related events | 18 | 0.375 | 4 | 0.000439 | 0.0114 |
| Netrin-1 signaling | 49 | 1.02 | 6 | 0.000489 | 0.0125 |
| GPVI-mediated activation cascade | 33 | 0.687 | 5 | 0.00054 | 0.0135 |
| Downstream signal transduction | 163 | 3.39 | 11 | 0.000551 | 0.0136 |
| Interleukin-3, 5 and GM-CSF signaling | 51 | 1.06 | 6 | 0.000609 | 0.0147 |
| Signal regulatory protein (SIRP) family interactions | 20 | 0.416 | 4 | 0.000672 | 0.016 |
| Semaphorin interactions | 72 | 1.5 | 7 | 0.000694 | 0.0162 |

| | | | | | |
|---|---|---|---|---|---|
| Insulin receptor signalling cascade | 95 | 1.98 | 8 | 0.000763 | 0.0175 |
| Signalling to ERKs | 37 | 0.77 | 5 | 0.000928 | 0.021 |
| Regulation of signaling by CBL | 22 | 0.458 | 4 | 0.000983 | 0.0219 |
| Signaling by EGFR | 179 | 3.73 | 11 | 0.0012 | 0.0263 |
| Signaling by EGFR in Cancer | 181 | 3.77 | 11 | 0.00131 | 0.0284 |
| GAB1 signalosome | 106 | 2.21 | 8 | 0.00156 | 0.0332 |
| Netrin mediated repulsion signals | 12 | 0.25 | 3 | 0.00169 | 0.0354 |
| DAP12 signaling | 164 | 3.41 | 10 | 0.00212 | 0.0438 |
| Constitutive PI3K/AKT Signaling in Cancer | 89 | 1.85 | 7 | 0.00242 | 0.0492 |
| SHC activation | 4 | 0.0833 | 2 | 0.00251 | 0.0496 |
| Neurophilin interactions with VEGF and VEGFR | 4 | 0.0833 | 2 | 0.00251 | 0.0496 |
| SEMA3A-Plexin repulsion signaling by inhibiting Integrin adhesion | 14 | 0.291 | 3 | 0.00272 | 0.0514 |
| Signaling by SCF-KIT | 142 | 2.96 | 9 | 0.00272 | 0.0514 |
| Signalling by NGF | 290 | 6.04 | 14 | 0.00273 | 0.0514 |
| IRS-related events triggered by IGF1R | 91 | 1.89 | 7 | 0.00275 | 0.0514 |
| Signaling by Insulin receptor | 117 | 2.44 | 8 | 0.00292 | 0.0539 |
| SHC-mediated signalling | 15 | 0.312 | 3 | 0.00334 | 0.0609 |
| Activation of Matrix Metalloproteinases | 31 | 0.645 | 4 | 0.00366 | 0.0657 |
| Downstream signaling of activated FGFR | 150 | 3.12 | 9 | 0.00393 | 0.0697 |
| Basigin interactions | 32 | 0.666 | 4 | 0.00411 | 0.0715 |
| TRAIL  signaling | 5 | 0.104 | 2 | 0.00413 | 0.0715 |
| Signaling by ERBB4 | 152 | 3.16 | 9 | 0.00428 | 0.0728 |
| Muscle contraction | 52 | 1.08 | 5 | 0.00431 | 0.0728 |
| DAP12 interactions | 182 | 3.79 | 10 | 0.00451 | 0.0753 |
| PI3K events in ERBB4 signaling | 103 | 2.14 | 7 | 0.00548 | 0.0863 |
| PIP3 activates AKT signaling | 103 | 2.14 | 7 | 0.00548 | 0.0863 |
| PI-3K cascade | 103 | 2.14 | 7 | 0.00548 | 0.0863 |
| PI3K/AKT Signaling in Cancer | 103 | 2.14 | 7 | 0.00548 | 0.0863 |
| PI3K events in ERBB2 signaling | 103 | 2.14 | 7 | 0.00548 | 0.0863 |
| PI3K/AKT activation | 106 | 2.21 | 7 | 0.0064 | 0.0997 |
| Signaling by FGFR | 162 | 3.37 | 9 | 0.00648 | 0.0999 |

| | | | | | |
|---|---|---|---|---|---|
| Signaling by ERBB2 | 164 | 3.41 | 9 | 0.00701 | 0.107 |
| TNF signaling | 7 | 0.146 | 2 | 0.00844 | 0.127 |
| Fcgamma receptor (FCGR) dependent phagocytosis | 86 | 1.79 | 6 | 0.00879 | 0.131 |
| SHC1 events in ERBB4 signaling | 21 | 0.437 | 3 | 0.00892 | 0.132 |
| Regulation of actin dynamics for phagocytic cup formation | 62 | 1.29 | 5 | 0.0091 | 0.133 |
| Signaling by Interleukins | 116 | 2.41 | 7 | 0.0103 | 0.148 |
| Cell junction organization | 89 | 1.85 | 6 | 0.0103 | 0.148 |
| NCAM signaling for neurite out-growth | 65 | 1.35 | 5 | 0.0111 | 0.154 |
| Cross-presentation of particulate exogenous antigens (phagosomes) | 8 | 0.167 | 2 | 0.0111 | 0.154 |
| Sema4D mediated inhibition of cell attachment and migration | 8 | 0.167 | 2 | 0.0111 | 0.154 |
| Innate Immune System | 521 | 10.8 | 19 | 0.0112 | 0.154 |
| Signaling by FGFR in disease | 178 | 3.71 | 9 | 0.0117 | 0.159 |
| Costimulation by the CD28 family | 68 | 1.42 | 5 | 0.0133 | 0.179 |
| SHC1 events in ERBB2 signaling | 25 | 0.52 | 3 | 0.0145 | 0.192 |
| Smooth Muscle Contraction | 25 | 0.52 | 3 | 0.0145 | 0.192 |
| Adaptive Immune System | 654 | 13.6 | 22 | 0.0157 | 0.205 |
| Dissolution of Fibrin Clot | 10 | 0.208 | 2 | 0.0174 | 0.223 |
| Dermatan sulfate biosynthesis | 10 | 0.208 | 2 | 0.0174 | 0.223 |
| Signalling to RAS | 27 | 0.562 | 3 | 0.0179 | 0.226 |
| A tetrasaccharide linker sequence is required for GAG synthesis | 27 | 0.562 | 3 | 0.0179 | 0.226 |
| SHC-mediated cascade | 28 | 0.583 | 3 | 0.0198 | 0.245 |
| Interleukin receptor SHC signaling | 28 | 0.583 | 3 | 0.0198 | 0.245 |
| Crosslinking of collagen fibrils | 11 | 0.229 | 2 | 0.0209 | 0.253 |
| TRIF-mediated programmed cell death | 11 | 0.229 | 2 | 0.0209 | 0.253 |
| DSCAM interactions | 11 | 0.229 | 2 | 0.0209 | 0.253 |
| Signaling by the B Cell Receptor (BCR) | 199 | 4.14 | 9 | 0.0227 | 0.271 |
| CD28 co-stimulation | 30 | 0.625 | 3 | 0.0238 | 0.283 |
| CD28 dependent Vav1 pathway | 12 | 0.25 | 2 | 0.0248 | 0.285 |
| Type I hemidesmosome assembly | 12 | 0.25 | 2 | 0.0248 | 0.285 |

| MODULE 4 | | | | | |
|---|---|---|---|---|---|
| Pathway | Total | Expected | Hits | P.Value | FDR |
| NGF signalling via TRKA from the plasma membrane | 207 | 2.82 | 30 | 1.31E-23 | 1.84E-20 |
| G2/M Transition | 102 | 1.39 | 23 | 1.17E-22 | 8.18E-20 |
| Mitotic G2-G2/M phases | 105 | 1.43 | 23 | 2.40E-22 | 1.12E-19 |
| Signaling by FGFR | 162 | 2.21 | 26 | 1.43E-21 | 5.00E-19 |
| Signaling by FGFR in disease | 178 | 2.43 | 26 | 1.73E-20 | 4.85E-18 |
| Signalling by NGF | 290 | 3.95 | 31 | 2.35E-20 | 5.48E-18 |
| Signalling to ERKs | 37 | 0.504 | 15 | 2.30E-19 | 4.61E-17 |
| SOS-mediated signalling | 14 | 0.191 | 11 | 5.70E-19 | 9.99E-17 |
| Cell Cycle, Mitotic | 411 | 5.6 | 34 | 8.08E-19 | 1.26E-16 |
| Cell Cycle | 508 | 6.92 | 37 | 1.03E-18 | 1.45E-16 |
| Downstream signaling of activated FGFR | 150 | 2.04 | 23 | 1.28E-18 | 1.63E-16 |
| Signalling to RAS | 27 | 0.368 | 13 | 3.97E-18 | 4.51E-16 |
| Loss of proteins required for interphase microtubule organization√Ç¬†from the centrosome | 65 | 0.886 | 17 | 4.50E-18 | 4.51E-16 |
| Loss of Nlp from mitotic centrosomes | 65 | 0.886 | 17 | 4.50E-18 | 4.51E-16 |
| Signaling by EGFR | 179 | 2.44 | 24 | 5.14E-18 | 4.81E-16 |
| Signaling by SCF-KIT | 142 | 1.94 | 22 | 6.35E-18 | 5.22E-16 |
| ARMS-mediated activation | 16 | 0.218 | 11 | 6.69E-18 | 5.22E-16 |
| Signaling by EGFR in Cancer | 181 | 2.47 | 24 | 6.71E-18 | 5.22E-16 |
| Downstream signal transduction | 163 | 2.22 | 23 | 8.80E-18 | 6.49E-16 |
| Signaling by ERBB2 | 164 | 2.24 | 23 | 1.01E-17 | 6.76E-16 |
| DAP12 signaling | 164 | 2.24 | 23 | 1.01E-17 | 6.76E-16 |
| Centrosome maturation | 86 | 1.17 | 18 | 3.33E-17 | 2.03E-15 |
| Recruitment of mitotic centrosome proteins and complexes | 86 | 1.17 | 18 | 3.33E-17 | 2.03E-15 |
| Frs2-mediated activation | 18 | 0.245 | 11 | 4.77E-17 | 2.79E-15 |
| RAF/MAP kinase cascade | 10 | 0.136 | 9 | 1.07E-16 | 5.91E-15 |
| DAP12 interactions | 182 | 2.48 | 23 | 1.10E-16 | 5.91E-15 |
| Prolonged ERK activation events | 20 | 0.273 | 11 | 2.46E-16 | 1.28E-14 |
| Signaling by PDGF | 189 | 2.58 | 23 | 2.58E-16 | 1.29E-14 |
| Signalling to p38 via RIT and RIN | 15 | 0.204 | 10 | 3.79E-16 | 1.83E-14 |

| | | | | | |
|---|---|---|---|---|---|
| Rap1 signalling | 17 | 0.232 | 10 | 2.40E-15 | 1.12E-13 |
| Signaling by ERBB4 | 152 | 2.07 | 20 | 6.53E-15 | 2.96E-13 |
| IRS-mediated signalling | 87 | 1.19 | 16 | 1.97E-14 | 8.64E-13 |
| GRB2 events in EGFR signaling | 14 | 0.191 | 9 | 2.05E-14 | 8.72E-13 |
| IRS-related events | 90 | 1.23 | 16 | 3.46E-14 | 1.43E-12 |
| IRS-related events triggered by IGF1R | 91 | 1.24 | 16 | 4.15E-14 | 1.66E-12 |
| SHC-mediated signalling | 15 | 0.204 | 9 | 5.08E-14 | 1.92E-12 |
| SHC1 events in EGFR signaling | 15 | 0.204 | 9 | 5.08E-14 | 1.92E-12 |
| RAF phosphorylates MEK | 7 | 0.0954 | 7 | 6.92E-14 | 2.42E-12 |
| MEK activation | 7 | 0.0954 | 7 | 6.92E-14 | 2.42E-12 |
| Signaling by Type 1 Insulin-like Growth Factor 1 Receptor (IGF1R) | 94 | 1.28 | 16 | 7.08E-14 | 2.42E-12 |
| IGF1R signaling cascade | 94 | 1.28 | 16 | 7.08E-14 | 2.42E-12 |
| Insulin receptor signalling cascade | 95 | 1.29 | 16 | 8.43E-14 | 2.81E-12 |
| SHC-related events | 18 | 0.245 | 9 | 4.77E-13 | 1.52E-11 |
| SHC-related events triggered by IGF1R | 18 | 0.245 | 9 | 4.77E-13 | 1.52E-11 |
| Signaling by Insulin receptor | 117 | 1.59 | 16 | 2.43E-12 | 7.56E-11 |
| SHC1 events in ERBB4 signaling | 21 | 0.286 | 9 | 2.79E-12 | 8.50E-11 |
| Immune System | 1140 | 15.5 | 44 | 3.85E-12 | 1.15E-10 |
| Translocation of GLUT4 to the Plasma Membrane | 71 | 0.968 | 13 | 7.09E-12 | 2.07E-10 |
| GRB2 events in ERBB2 signaling | 23 | 0.313 | 9 | 7.58E-12 | 2.17E-10 |
| SHC1 events in ERBB2 signaling | 25 | 0.341 | 9 | 1.85E-11 | 5.20E-10 |
| Activation of BAD and translocation to mitochondria | 17 | 0.232 | 8 | 1.92E-11 | 5.27E-10 |
| Adaptive Immune System | 654 | 8.91 | 32 | 3.44E-11 | 9.28E-10 |
| Innate Immune System | 521 | 7.1 | 28 | 1.00E-10 | 2.65E-09 |
| p38MAPK events | 13 | 0.177 | 7 | 1.11E-10 | 2.88E-09 |
| Interleukin-2 signaling | 42 | 0.572 | 10 | 1.37E-10 | 3.50E-09 |
| RAF activation | 5 | 0.0681 | 5 | 4.21E-10 | 1.05E-08 |
| Activation of BH3-only proteins | 24 | 0.327 | 8 | 5.36E-10 | 1.32E-08 |
| Intrinsic Pathway for Apoptosis | 37 | 0.504 | 9 | 9.87E-10 | 2.39E-08 |
| FRS2-mediated cascade | 39 | 0.532 | 9 | 1.65E-09 | 3.91E-08 |
| Signaling by Interleukins | 116 | 1.58 | 13 | 4.09E-09 | 9.57E-08 |

| | | | | | |
|---|---|---|---|---|---|
| G2/M Checkpoints | 48 | 0.654 | 9 | 1.18E-08 | 2.71E-07 |
| Regulation of mRNA Stability by Proteins that Bind AU-rich Elements | 88 | 1.2 | 11 | 2.21E-08 | 4.99E-07 |
| Disease | 945 | 12.9 | 34 | 2.62E-08 | 5.84E-07 |
| MAP kinase activation in TLR cascade | 55 | 0.75 | 9 | 4.13E-08 | 9.05E-07 |
| Signaling by the B Cell Receptor (BCR) | 199 | 2.71 | 15 | 5.73E-08 | 1.22E-06 |
| Activation of ATR in response to replication stress | 41 | 0.559 | 8 | 5.77E-08 | 1.22E-06 |
| Downstream Signaling Events Of B Cell Receptor (BCR) | 173 | 2.36 | 14 | 6.83E-08 | 1.43E-06 |
| Mitotic M-M/G1 phases | 266 | 3.63 | 17 | 7.79E-08 | 1.61E-06 |
| TRAF6 Mediated Induction of proinflammatory cytokines | 62 | 0.845 | 9 | 1.22E-07 | 2.48E-06 |
| ERK activation | 5 | 0.0681 | 4 | 1.60E-07 | 3.20E-06 |
| Signal Transduction | 1690 | 23 | 46 | 1.66E-07 | 3.28E-06 |
| NCAM signaling for neurite out-growth | 65 | 0.886 | 9 | 1.86E-07 | 3.62E-06 |
| Cytokine Signaling in Immune system | 286 | 3.9 | 17 | 2.23E-07 | 4.29E-06 |
| Constitutive PI3K/AKT Signaling in Cancer | 89 | 1.21 | 10 | 2.82E-07 | 5.33E-06 |
| Resolution of Sister Chromatid Cohesion | 118 | 1.61 | 11 | 4.73E-07 | 8.84E-06 |
| Toll Like Receptor 10 (TLR10) Cascade | 74 | 1.01 | 9 | 5.81E-07 | 1.04E-05 |
| Toll Like Receptor 5 (TLR5) Cascade | 74 | 1.01 | 9 | 5.81E-07 | 1.04E-05 |
| MyD88 cascade initiated on plasma membrane | 74 | 1.01 | 9 | 5.81E-07 | 1.04E-05 |
| Nuclear Events (kinase and transcription factor activation) | 24 | 0.327 | 6 | 6.00E-07 | 1.06E-05 |
| TRAF6 mediated induction of NFkB and MAP kinases upon TLR7/8 or 9 activation | 76 | 1.04 | 9 | 7.32E-07 | 1.28E-05 |
| Toll Like Receptor 7/8 (TLR7/8) Cascade | 77 | 1.05 | 9 | 8.20E-07 | 1.40E-05 |
| MyD88 dependent cascade initiated on endosome | 77 | 1.05 | 9 | 8.20E-07 | 1.40E-05 |
| Mitotic Prometaphase | 127 | 1.73 | 11 | 9.94E-07 | 1.68E-05 |
| Toll Like Receptor 9 (TLR9) Cascade | 79 | 1.08 | 9 | 1.02E-06 | 1.71E-05 |
| PI3K events in ERBB4 signaling | 103 | 1.4 | 10 | 1.12E-06 | 1.76E-05 |
| PIP3 activates AKT signaling | 103 | 1.4 | 10 | 1.12E-06 | 1.76E-05 |
| PI-3K cascade | 103 | 1.4 | 10 | 1.12E-06 | 1.76E-05 |
| PI3K/AKT Signaling in Cancer | 103 | 1.4 | 10 | 1.12E-06 | 1.76E-05 |
| PI3K events in ERBB2 signaling | 103 | 1.4 | 10 | 1.12E-06 | 1.76E-05 |
| MyD88:Mal cascade initiated on plasma membrane | 81 | 1.1 | 9 | 1.27E-06 | 1.91E-05 |
| Toll Like Receptor TLR1:TLR2 Cascade | 81 | 1.1 | 9 | 1.27E-06 | 1.91E-05 |

| | | | | | |
|---|---|---|---|---|---|
| Toll Like Receptor TLR6:TLR2 Cascade | 81 | 1.1 | 9 | 1.27E-06 | 1.91E-05 |
| Toll Like Receptor 2 (TLR2) Cascade | 81 | 1.1 | 9 | 1.27E-06 | 1.91E-05 |
| Cell Cycle Checkpoints | 131 | 1.79 | 11 | 1.36E-06 | 2.02E-05 |
| GAB1 signalosome | 106 | 1.44 | 10 | 1.46E-06 | 2.13E-05 |
| PI3K/AKT activation | 106 | 1.44 | 10 | 1.46E-06 | 2.13E-05 |
| TRIF-mediated TLR3/TLR4 signaling | 87 | 1.19 | 9 | 2.33E-06 | 3.37E-05 |
| Developmental Biology | 417 | 5.68 | 19 | 2.39E-06 | 3.42E-05 |
| Processing of DNA double-strand break ends | 3 | 0.0409 | 3 | 2.45E-06 | 3.47E-05 |
| MAPK targets/ Nuclear events mediated by MAP kinases | 30 | 0.409 | 6 | 2.48E-06 | 3.47E-05 |
| MyD88-independent cascade | 88 | 1.2 | 9 | 2.57E-06 | 3.53E-05 |
| Toll Like Receptor 3 (TLR3) Cascade | 88 | 1.2 | 9 | 2.57E-06 | 3.53E-05 |
| M Phase | 233 | 3.18 | 14 | 2.63E-06 | 3.57E-05 |
| Membrane Trafficking | 203 | 2.77 | 13 | 3.07E-06 | 4.14E-05 |
| Hemostasis | 511 | 6.96 | 21 | 3.36E-06 | 4.48E-05 |
| EGFR Transactivation by Gastrin | 9 | 0.123 | 4 | 3.86E-06 | 5.11E-05 |
| Activation of the AP-1 family of transcription factors | 10 | 0.136 | 4 | 6.37E-06 | 8.35E-05 |
| ERK/MAPK targets | 21 | 0.286 | 5 | 7.21E-06 | 9.36E-05 |
| Activated TLR4 signalling | 100 | 1.36 | 9 | 7.48E-06 | 9.63E-05 |
| Apoptosis | 158 | 2.15 | 11 | 8.51E-06 | 0.000108 |
| Toll Like Receptor 4 (TLR4) Cascade | 103 | 1.4 | 9 | 9.55E-06 | 0.000121 |
| AKT phosphorylates targets in the cytosol | 13 | 0.177 | 4 | 2.10E-05 | 0.000263 |
| Recruitment of NuMA to mitotic centrosomes | 26 | 0.354 | 5 | 2.21E-05 | 0.000274 |
| Activation of RAS in B Cells | 5 | 0.0681 | 3 | 2.40E-05 | 0.00029 |
| Assembly of the RAD51-ssDNA nucleoprotein complex | 5 | 0.0681 | 3 | 2.40E-05 | 0.00029 |
| Cyclin B2 mediated events | 5 | 0.0681 | 3 | 2.40E-05 | 0.00029 |
| Axon guidance | 292 | 3.98 | 14 | 3.50E-05 | 0.000419 |
| Repair synthesis of patch ~27-30 bases long by DNA polymerase | 15 | 0.204 | 4 | 3.93E-05 | 0.000462 |
| Repair synthesis for gap-filling by DNA polymerase in TC-NER | 15 | 0.204 | 4 | 3.93E-05 | 0.000462 |
| Separation of Sister Chromatids | 186 | 2.53 | 11 | 3.96E-05 | 0.000462 |

| MODULE 5 | | | | | |
|---|---|---|---|---|---|
| Pathway | Total | Expected | Hits | P.Value | FDR |
| Protein folding | 56 | 0.453 | 22 | 1.20E-33 | 1.69E-30 |
| Cooperation of Prefoldin and TriC/CCT in actin and tubulin folding | 30 | 0.243 | 18 | 7.42E-32 | 5.20E-29 |
| Prefoldin mediated transfer of substrate to CCT/TriC | 29 | 0.235 | 17 | 8.00E-30 | 3.74E-27 |
| Chaperonin-mediated protein folding | 51 | 0.412 | 18 | 2.15E-26 | 7.53E-24 |
| Fcgamma receptor (FCGR) dependent phagocytosis | 86 | 0.696 | 20 | 3.43E-25 | 9.63E-23 |
| Regulation of actin dynamics for phagocytic cup formation | 62 | 0.501 | 17 | 9.58E-23 | 2.24E-20 |
| Formation of tubulin folding intermediates by CCT/TriC | 23 | 0.186 | 13 | 1.44E-22 | 2.88E-20 |
| Post-chaperonin tubulin folding pathway | 20 | 0.162 | 10 | 8.64E-17 | 1.49E-14 |
| Folding of actin by CCT/TriC | 9 | 0.0728 | 8 | 9.54E-17 | 1.49E-14 |
| Innate Immune System | 521 | 4.21 | 22 | 2.03E-11 | 2.85E-09 |
| Translocation of GLUT4 to the Plasma Membrane | 71 | 0.574 | 10 | 1.59E-10 | 2.02E-08 |
| Immune System | 1140 | 9.2 | 29 | 8.03E-10 | 9.38E-08 |
| Microtubule-dependent trafficking of connexons from Golgi to the plasma membrane | 16 | 0.129 | 6 | 1.58E-09 | 1.71E-07 |
| Association of TriC/CCT with target proteins during biosynthesis | 29 | 0.235 | 7 | 2.06E-09 | 2.06E-07 |
| Transport of connexons to the plasma membrane | 17 | 0.137 | 6 | 2.43E-09 | 2.27E-07 |
| Metabolism of proteins | 689 | 5.57 | 22 | 4.59E-09 | 4.02E-07 |
| Recruitment of NuMA to mitotic centrosomes | 26 | 0.21 | 6 | 4.28E-08 | 3.40E-06 |
| Gap junction trafficking | 44 | 0.356 | 7 | 4.61E-08 | 3.40E-06 |
| Recycling pathway of L1 | 44 | 0.356 | 7 | 4.61E-08 | 3.40E-06 |
| Gap junction trafficking and regulation | 46 | 0.372 | 7 | 6.36E-08 | 4.46E-06 |
| Gap junction assembly | 33 | 0.267 | 6 | 1.97E-07 | 1.32E-05 |
| Kinesins | 41 | 0.332 | 6 | 7.62E-07 | 4.85E-05 |
| Membrane Trafficking | 203 | 1.64 | 10 | 4.02E-06 | 0.000245 |
| L1CAM interactions | 112 | 0.906 | 7 | 2.86E-05 | 0.00167 |
| MHC class II antigen presentation | 118 | 0.954 | 7 | 4.01E-05 | 0.00216 |
| Resolution of Sister Chromatid Cohesion | 118 | 0.954 | 7 | 4.01E-05 | 0.00216 |
| Centrosome maturation | 86 | 0.696 | 6 | 6.03E-05 | 0.00302 |
| Recruitment of mitotic centrosome proteins and complexes | 86 | 0.696 | 6 | 6.03E-05 | 0.00302 |
| Mitotic Prometaphase | 127 | 1.03 | 7 | 6.43E-05 | 0.00311 |

| | | | | | |
|---|---|---|---|---|---|
| Axon guidance | 292 | 2.36 | 10 | 9.48E-05 | 0.00443 |
| G2/M Transition | 102 | 0.825 | 6 | 0.000156 | 0.00707 |
| Mitotic G2-G2/M phases | 105 | 0.849 | 6 | 0.000183 | 0.00804 |
| Factors involved in megakaryocyte development and platelet production | 155 | 1.25 | 7 | 0.000225 | 0.00955 |
| Separation of Sister Chromatids | 186 | 1.5 | 7 | 0.00068 | 0.028 |
| Mitotic Anaphase | 198 | 1.6 | 7 | 0.000985 | 0.0394 |
| Mitotic Metaphase and Anaphase | 199 | 1.61 | 7 | 0.00101 | 0.0395 |
| Role of phospholipids in phagocytosis | 27 | 0.218 | 3 | 0.00128 | 0.0483 |
| Synthesis of PG | 7 | 0.0566 | 2 | 0.00131 | 0.0485 |
| Developmental Biology | 417 | 3.37 | 10 | 0.00162 | 0.0582 |
| M Phase | 233 | 1.88 | 7 | 0.00252 | 0.0882 |
| Hormone-sensitive lipase (HSL)-mediated triacylglycerol hydrolysis | 12 | 0.097 | 2 | 0.00402 | 0.138 |
| Mitotic M-M/G1 phases | 266 | 2.15 | 7 | 0.00524 | 0.175 |
| Muscle contraction | 52 | 0.421 | 3 | 0.00836 | 0.272 |
| Signaling by TGF-beta Receptor Complex | 70 | 0.566 | 3 | 0.0187 | 0.596 |
| Downregulation of TGF-beta receptor signaling | 27 | 0.218 | 2 | 0.0198 | 0.613 |
| Hemostasis | 511 | 4.13 | 9 | 0.0201 | 0.613 |
| Synthesis of PA | 28 | 0.226 | 2 | 0.0212 | 0.633 |
| Sema4D induced cell migration and growth-cone collapse | 29 | 0.235 | 2 | 0.0227 | 0.662 |
| TGF-beta receptor signaling activates SMADs | 30 | 0.243 | 2 | 0.0242 | 0.691 |
| Striated Muscle Contraction | 31 | 0.251 | 2 | 0.0257 | 0.721 |
| Sema4D in semaphorin signaling | 34 | 0.275 | 2 | 0.0305 | 0.839 |
| Lipid digestion, mobilization, and transport | 41 | 0.332 | 2 | 0.0431 | 1 |
| Cell Cycle, Mitotic | 411 | 3.32 | 7 | 0.0462 | 1 |

| MODULE 6 | | | | | |
|---|---|---|---|---|---|
| Pathway | Total | Expected | Hits | P.Value | FDR |
| Class A/1 (Rhodopsin-like receptors) | 312 | 1.68 | 10 | 3.69E-06 | 0.00517 |
| GPCR ligand binding | 415 | 2.24 | 10 | 4.54E-05 | 0.021 |
| G alpha (i) signalling events | 184 | 0.992 | 7 | 4.57E-05 | 0.021 |
| Peptide ligand-binding receptors | 192 | 1.04 | 7 | 5.99E-05 | 0.021 |
| Amyloids | 84 | 0.453 | 5 | 7.76E-05 | 0.0217 |
| Signal Transduction | 1690 | 9.1 | 19 | 0.000363 | 0.0849 |
| p75 NTR receptor-mediated signalling | 85 | 0.458 | 4 | 0.00106 | 0.212 |
| Apoptotic cleavage of cellular proteins | 43 | 0.232 | 3 | 0.00153 | 0.268 |
| Caspase-mediated cleavage of cytoskeletal proteins | 13 | 0.0701 | 2 | 0.00212 | 0.298 |
| Apoptotic cleavage of cell adhesion proteins | 13 | 0.0701 | 2 | 0.00212 | 0.298 |
| NRIF signals cell death from the nucleus | 15 | 0.0809 | 2 | 0.00284 | 0.362 |
| G alpha (q) signalling events | 188 | 1.01 | 5 | 0.00311 | 0.364 |
| Apoptotic execution phase | 57 | 0.307 | 3 | 0.00345 | 0.372 |
| Cell death signalling via NRAGE, NRIF and NADE | 62 | 0.334 | 3 | 0.00438 | 0.438 |
| Gastrin-CREB signalling pathway via PKC and MAPK | 209 | 1.13 | 5 | 0.0049 | 0.438 |
| Cell-Cell communication | 143 | 0.771 | 4 | 0.00698 | 0.438 |
| Signaling by NOTCH1 t(7;9)(NOTCH1:M1580_K2555) Translocation Mutant | 74 | 0.399 | 3 | 0.00718 | 0.438 |
| Signaling by NOTCH1 in Cancer | 74 | 0.399 | 3 | 0.00718 | 0.438 |
| Signaling by NOTCH1 PEST Domain Mutants in Cancer | 74 | 0.399 | 3 | 0.00718 | 0.438 |
| FBXW7 Mutants and NOTCH1 in Cancer | 74 | 0.399 | 3 | 0.00718 | 0.438 |
| Signaling by NOTCH1 HD Domain Mutants in Cancer | 74 | 0.399 | 3 | 0.00718 | 0.438 |
| Signaling by NOTCH1 HD+PEST Domain Mutants in Cancer | 74 | 0.399 | 3 | 0.00718 | 0.438 |
| Signaling by NOTCH1 | 74 | 0.399 | 3 | 0.00718 | 0.438 |
| GPCR downstream signaling | 812 | 4.38 | 10 | 0.00879 | 0.514 |
| Apoptosis | 158 | 0.852 | 4 | 0.00986 | 0.52 |
| Activated NOTCH1 Transmits Signal to the Nucleus | 29 | 0.156 | 2 | 0.0105 | 0.52 |
| G-protein activation | 29 | 0.156 | 2 | 0.0105 | 0.52 |
| Translesion synthesis by Pol zeta | 2 | 0.0108 | 1 | 0.0108 | 0.52 |
| Stimulation of the cell death response by PAK-2p34 | 2 | 0.0108 | 1 | 0.0108 | 0.52 |

| | | | | | |
|---|---|---|---|---|---|
| Adherens junctions interactions | 31 | 0.167 | 2 | 0.0119 | 0.557 |
| Signaling by NOTCH | 95 | 0.512 | 3 | 0.0142 | 0.642 |
| Activation of BIM and translocation to mitochondria | 3 | 0.0162 | 1 | 0.0161 | 0.684 |
| Activation of BMF and translocation to mitochondria | 3 | 0.0162 | 1 | 0.0161 | 0.684 |
| Intrinsic Pathway for Apoptosis | 37 | 0.199 | 2 | 0.0167 | 0.69 |
| Signalling by NGF | 290 | 1.56 | 5 | 0.0186 | 0.747 |
| Cyclin D associated events in G1 | 41 | 0.221 | 2 | 0.0203 | 0.769 |
| G1 Phase | 41 | 0.221 | 2 | 0.0203 | 0.769 |
| DNA Damage Bypass | 4 | 0.0216 | 1 | 0.0214 | 0.769 |
| Translesion synthesis by DNA polymerases bypassing lesion on DNA template | 4 | 0.0216 | 1 | 0.0214 | 0.769 |
| Signaling by GPCR | 931 | 5.02 | 10 | 0.0219 | 0.769 |
| Disease | 945 | 5.1 | 10 | 0.0241 | 0.813 |
| SMAC-mediated dissociation of IAP:caspase complexes | 5 | 0.027 | 1 | 0.0267 | 0.813 |
| SMAC-mediated apoptotic response | 5 | 0.027 | 1 | 0.0267 | 0.813 |
| Activation of caspases through apoptosome-mediated cleavage | 5 | 0.027 | 1 | 0.0267 | 0.813 |
| SMAC binds to IAPs | 5 | 0.027 | 1 | 0.0267 | 0.813 |
| Cytochrome c-mediated apoptotic response | 5 | 0.027 | 1 | 0.0267 | 0.813 |
| Constitutive Signaling by NOTCH1 HD+PEST Domain Mutants | 52 | 0.28 | 2 | 0.0317 | 0.914 |
| p75NTR negatively regulates cell cycle via SC1 | 6 | 0.0323 | 1 | 0.0319 | 0.914 |
| Formyl peptide receptors bind formyl peptides and many other ligands | 6 | 0.0323 | 1 | 0.0319 | 0.914 |
| Apoptotic factor-mediated response | 7 | 0.0377 | 1 | 0.0372 | 1 |
| Neurotransmitter Clearance In The Synaptic Cleft | 7 | 0.0377 | 1 | 0.0372 | 1 |
| NADE modulates death signalling | 7 | 0.0377 | 1 | 0.0372 | 1 |
| Constitutive Signaling by NOTCH1 PEST Domain Mutants | 59 | 0.318 | 2 | 0.0399 | 1 |
| Cell-cell junction organization | 60 | 0.323 | 2 | 0.0411 | 1 |
| Peptide hormone metabolism | 60 | 0.323 | 2 | 0.0411 | 1 |
| Lysosphingolipid and LPA receptors | 8 | 0.0431 | 1 | 0.0423 | 1 |
| TRAF6 Mediated Induction of proinflammatory cytokines | 62 | 0.334 | 2 | 0.0437 | 1 |
| A third proteolytic cleavage releases NICD | 9 | 0.0485 | 1 | 0.0475 | 1 |
| Peptide hormone biosynthesis | 9 | 0.0485 | 1 | 0.0475 | 1 |
| Relaxin receptors | 9 | 0.0485 | 1 | 0.0475 | 1 |

| | | | | | |
|---|---|---|---|---|---|
| Role of Abl in Robo-Slit signaling | 9 | 0.0485 | 1 | 0.0475 | 1 |

| MODULE 7 | | | | | |
|---|---|---|---|---|---|
| Pathway | Total | Expected | Hits | P.Value | FDR |
| Lipoprotein metabolism | 22 | 0.119 | 13 | 1.34E-25 | 1.88E-22 |
| Lipid digestion, mobilization, and transport | 41 | 0.221 | 13 | 4.47E-21 | 3.13E-18 |
| HDL-mediated lipid transport | 13 | 0.0701 | 9 | 9.18E-19 | 4.29E-16 |
| Retinoid metabolism and transport | 41 | 0.221 | 10 | 5.29E-15 | 1.86E-12 |
| Chylomicron-mediated lipid transport | 12 | 0.0647 | 7 | 5.54E-14 | 1.55E-11 |
| Visual phototransduction | 56 | 0.302 | 10 | 1.59E-13 | 3.72E-11 |
| Diseases associated with visual transduction | 57 | 0.307 | 10 | 1.93E-13 | 3.86E-11 |
| Scavenging of Heme from Plasma | 15 | 0.0809 | 5 | 9.86E-09 | 1.54E-06 |
| Binding and Uptake of Ligands by Scavenger Receptors | 15 | 0.0809 | 5 | 9.86E-09 | 1.54E-06 |
| Metabolism of lipids and lipoproteins | 507 | 2.73 | 15 | 1.64E-08 | 2.30E-06 |
| Platelet degranulation | 89 | 0.48 | 7 | 3.59E-07 | 4.58E-05 |
| Response to elevated platelet cytosolic Ca2+ | 94 | 0.507 | 7 | 5.24E-07 | 6.12E-05 |
| LDL-mediated lipid transport | 4 | 0.0216 | 3 | 5.74E-07 | 6.19E-05 |
| Vitamin D (calciferol) metabolism | 7 | 0.0377 | 3 | 4.96E-06 | 0.000497 |
| Platelet activation, signaling and aggregation | 220 | 1.19 | 7 | 0.000142 | 0.0132 |
| Metabolism of steroid hormones and vitamin D | 26 | 0.14 | 3 | 0.000344 | 0.0283 |
| Steroid hormones | 26 | 0.14 | 3 | 0.000344 | 0.0283 |
| Hemostasis | 511 | 2.76 | 9 | 0.00122 | 0.0913 |
| Retinoid cycle disease events | 10 | 0.0539 | 2 | 0.00124 | 0.0913 |
| The canonical retinoid cycle in rods (twilight vision) | 15 | 0.0809 | 2 | 0.00284 | 0.199 |
| PPARA Activates Gene Expression | 59 | 0.318 | 3 | 0.0038 | 0.254 |
| Platelet sensitization by LDL | 19 | 0.102 | 2 | 0.00456 | 0.291 |
| Metabolism | 1490 | 8.02 | 15 | 0.00707 | 0.431 |
| Regulation of Lipid Metabolism by Peroxisome proliferator-activated receptor alpha (PPARalpha) | 76 | 0.41 | 3 | 0.00773 | 0.451 |
| Transferrin endocytosis and recycling | 26 | 0.14 | 2 | 0.00847 | 0.474 |
| Disease | 945 | 5.1 | 11 | 0.0088 | 0.474 |
| Activation, translocation and oligomerization of BAX | 2 | 0.0108 | 1 | 0.0108 | 0.558 |
| Iron uptake and transport | 38 | 0.205 | 2 | 0.0176 | 0.881 |
| Processing of DNA ends prior to end rejoining | 6 | 0.0323 | 1 | 0.0319 | 1 |

| | | | | | |
|---|---|---|---|---|---|
| Fatty acid, triacylglycerol, and ketone body metabolism | 139 | 0.749 | 3 | 0.0382 | 1 |
| Nonhomologous End-joining (NHEJ) | 8 | 0.0431 | 1 | 0.0423 | 1 |
| 2-LTR circle formation | 9 | 0.0485 | 1 | 0.0475 | 1 |

| MODULE 8 | | | | | |
|---|---|---|---|---|---|
| Pathway | Total | Expected | Hits | P.Value | FDR |
| G alpha (s) signalling events | 127 | 0.913 | 32 | 2.33E-45 | 3.26E-42 |
| Opioid Signalling | 86 | 0.618 | 29 | 7.65E-45 | 5.36E-42 |
| Glucagon signaling in metabolic regulation | 37 | 0.266 | 23 | 5.23E-43 | 2.44E-40 |
| G alpha (z) signalling events | 38 | 0.273 | 23 | 1.32E-42 | 4.62E-40 |
| Regulation of Water Balance by Renal Aquaporins | 47 | 0.338 | 24 | 5.03E-42 | 1.41E-39 |
| GABA B receptor activation | 40 | 0.288 | 23 | 7.51E-42 | 1.50E-39 |
| Activation of GABAB receptors | 40 | 0.288 | 23 | 7.51E-42 | 1.50E-39 |
| Integration of energy metabolism | 109 | 0.784 | 29 | 2.42E-41 | 4.24E-39 |
| Aquaporin-mediated transport | 55 | 0.395 | 24 | 7.56E-40 | 1.18E-37 |
| G-protein activation | 29 | 0.208 | 20 | 1.30E-38 | 1.83E-36 |
| GABA receptor activation | 55 | 0.395 | 23 | 1.50E-37 | 1.91E-35 |
| Signal amplification | 33 | 0.237 | 19 | 2.40E-34 | 2.80E-32 |
| Inhibition of Insulin Secretion by Adrenaline/Noradrenaline | 28 | 0.201 | 18 | 8.65E-34 | 9.33E-32 |
| ADP signalling through P2Y purinoceptor 12 | 23 | 0.165 | 17 | 1.45E-33 | 1.46E-31 |
| GPCR downstream signaling | 812 | 5.84 | 42 | 8.55E-33 | 7.99E-31 |
| G alpha (i) signalling events | 184 | 1.32 | 28 | 2.72E-32 | 2.39E-30 |
| Signaling by GPCR | 931 | 6.69 | 43 | 5.86E-32 | 4.77E-30 |
| Prostacyclin signalling through prostacyclin receptor | 21 | 0.151 | 16 | 6.12E-32 | 4.77E-30 |
| Neurotransmitter Receptor Binding And Downstream Transmission In The Postsynaptic Cell | 143 | 1.03 | 26 | 6.97E-32 | 5.14E-30 |
| Thrombin signalling through proteinase activated receptors (PARs) | 34 | 0.244 | 18 | 1.42E-31 | 9.93E-30 |
| Transmission across Chemical Synapses | 196 | 1.41 | 28 | 1.77E-31 | 1.18E-29 |
| Regulation of Insulin Secretion | 82 | 0.589 | 22 | 9.35E-31 | 5.96E-29 |
| Thromboxane signalling through TP receptor | 25 | 0.18 | 16 | 6.04E-30 | 3.68E-28 |
| Regulation of Insulin Secretion by Glucagon-like Peptide-1 | 47 | 0.338 | 18 | 2.78E-28 | 1.62E-26 |
| Glucagon-type ligand receptors | 32 | 0.23 | 16 | 1.72E-27 | 9.65E-26 |
| ADP signalling through P2Y purinoceptor 1 | 27 | 0.194 | 15 | 1.02E-26 | 5.51E-25 |
| Neuronal System | 292 | 2.1 | 28 | 1.86E-26 | 9.67E-25 |
| G beta:gamma signalling through PLC beta | 22 | 0.158 | 14 | 3.75E-26 | 1.88E-24 |
| Adenylate cyclase inhibitory pathway | 13 | 0.0935 | 12 | 5.55E-26 | 2.59E-24 |

| | | | | | |
|---|---|---|---|---|---|
| Inhibition of adenylate cyclase pathway | 13 | 0.0935 | 12 | 5.55E-26 | 2.59E-24 |
| Presynaptic function of Kainate receptors | 23 | 0.165 | 14 | 9.53E-26 | 4.31E-24 |
| Signal Transduction | 1690 | 12.1 | 46 | 1.38E-25 | 6.03E-24 |
| G beta:gamma signalling through PI3Kgamma | 27 | 0.194 | 14 | 2.30E-24 | 9.75E-23 |
| G-protein beta:gamma signalling | 30 | 0.216 | 14 | 1.64E-23 | 6.76E-22 |
| Activation of Kainate Receptors upon glutamate binding | 34 | 0.244 | 14 | 1.54E-22 | 6.17E-21 |
| PLC beta mediated events | 46 | 0.331 | 15 | 2.75E-22 | 1.07E-20 |
| G-protein mediated events | 47 | 0.338 | 15 | 4.02E-22 | 1.52E-20 |
| G alpha (12/13) signalling events | 80 | 0.575 | 16 | 6.20E-20 | 2.29E-18 |
| Adenylate cyclase activating pathway | 10 | 0.0719 | 9 | 2.31E-19 | 8.30E-18 |
| Class B/2 (Secretin family receptors) | 87 | 0.625 | 16 | 2.64E-19 | 9.25E-18 |
| Platelet activation, signaling and aggregation | 220 | 1.58 | 21 | 2.94E-19 | 1.01E-17 |
| Platelet homeostasis | 88 | 0.633 | 16 | 3.21E-19 | 1.07E-17 |
| Activation of G protein gated Potassium channels | 27 | 0.194 | 11 | 9.29E-18 | 2.89E-16 |
| G protein gated Potassium channels | 27 | 0.194 | 11 | 9.29E-18 | 2.89E-16 |
| Inhibition of voltage gated Ca2+ channels via Gbeta/gamma subunits | 27 | 0.194 | 11 | 9.29E-18 | 2.89E-16 |
| PKA activation in glucagon signalling | 19 | 0.137 | 10 | 1.20E-17 | 3.65E-16 |
| GPCR ligand binding | 415 | 2.98 | 24 | 4.67E-17 | 1.39E-15 |
| Inwardly rectifying K+ channels | 33 | 0.237 | 11 | 1.34E-16 | 3.91E-15 |
| G alpha (q) signalling events | 188 | 1.35 | 18 | 1.91E-16 | 5.48E-15 |
| PKA activation | 18 | 0.129 | 9 | 1.08E-15 | 3.02E-14 |
| Gastrin-CREB signalling pathway via PKC and MAPK | 209 | 1.5 | 18 | 1.28E-15 | 3.52E-14 |
| PKA-mediated phosphorylation of CREB | 19 | 0.137 | 9 | 2.03E-15 | 5.48E-14 |
| Transmembrane transport of small molecules | 504 | 3.62 | 24 | 4.03E-15 | 1.07E-13 |
| Hemostasis | 511 | 3.67 | 23 | 6.70E-14 | 1.74E-12 |
| Calmodulin induced events | 30 | 0.216 | 9 | 2.97E-13 | 7.44E-12 |
| CaM pathway | 30 | 0.216 | 9 | 2.97E-13 | 7.44E-12 |
| Ca-dependent events | 32 | 0.23 | 9 | 5.76E-13 | 1.42E-11 |
| DAG and IP3 signaling | 35 | 0.252 | 9 | 1.43E-12 | 3.45E-11 |
| PLC-gamma1 signalling | 37 | 0.266 | 9 | 2.49E-12 | 5.82E-11 |
| EGFR interacts with phospholipase C-gamma | 37 | 0.266 | 9 | 2.49E-12 | 5.82E-11 |

| | | | | | |
|---|---|---|---|---|---|
| PLCG1 events in ERBB2 signaling | 38 | 0.273 | 9 | 3.24E-12 | 7.46E-11 |
| Potassium Channels | 103 | 0.74 | 11 | 9.63E-11 | 2.18E-09 |
| Phospholipase C-mediated cascade | 57 | 0.41 | 9 | 1.62E-10 | 3.60E-09 |
| Metabolism | 1490 | 10.7 | 29 | 1.25E-08 | 2.75E-07 |
| NGF signalling via TRKA from the plasma membrane | 207 | 1.49 | 11 | 1.61E-07 | 3.47E-06 |
| Downstream signaling of activated FGFR | 150 | 1.08 | 9 | 9.10E-07 | 1.93E-05 |
| Signaling by FGFR | 162 | 1.16 | 9 | 1.74E-06 | 3.64E-05 |
| Downstream signal transduction | 163 | 1.17 | 9 | 1.83E-06 | 3.77E-05 |
| Signaling by ERBB2 | 164 | 1.18 | 9 | 1.93E-06 | 3.86E-05 |
| DAP12 signaling | 164 | 1.18 | 9 | 1.93E-06 | 3.86E-05 |
| CREB phosphorylation through the activation of Adenylate Cyclase | 5 | 0.0359 | 3 | 3.45E-06 | 6.82E-05 |
| Signaling by FGFR in disease | 178 | 1.28 | 9 | 3.80E-06 | 7.41E-05 |
| Signaling by EGFR | 179 | 1.29 | 9 | 3.98E-06 | 7.65E-05 |
| Signaling by EGFR in Cancer | 181 | 1.3 | 9 | 4.37E-06 | 8.27E-05 |
| DAP12 interactions | 182 | 1.31 | 9 | 4.57E-06 | 8.50E-05 |
| Signalling by NGF | 290 | 2.08 | 11 | 4.61E-06 | 8.50E-05 |
| Signaling by PDGF | 189 | 1.36 | 9 | 6.23E-06 | 0.000113 |
| Prostanoid ligand receptors | 11 | 0.0791 | 3 | 5.53E-05 | 0.000993 |
| Eicosanoid ligand-binding receptors | 17 | 0.122 | 3 | 0.000221 | 0.00392 |
| Innate Immune System | 521 | 3.75 | 11 | 0.000929 | 0.0163 |
| Class A/1 (Rhodopsin-like receptors) | 312 | 2.24 | 8 | 0.00153 | 0.0264 |
| Post NMDA receptor activation events | 35 | 0.252 | 3 | 0.00194 | 0.0332 |
| Hormone ligand-binding receptors | 10 | 0.0719 | 2 | 0.0022 | 0.0371 |
| Activation of NMDA receptor upon glutamate binding and postsynaptic events | 39 | 0.28 | 3 | 0.00266 | 0.0441 |
| DSCAM interactions | 11 | 0.0791 | 2 | 0.00267 | 0.0441 |
| Depolarization of the Presynaptic Terminal Triggers the Opening of Calcium Channels | 12 | 0.0863 | 2 | 0.00319 | 0.052 |
| p38MAPK events | 13 | 0.0935 | 2 | 0.00375 | 0.0605 |
| Signalling to RAS | 27 | 0.194 | 2 | 0.0158 | 0.252 |
| Signalling to ERKs | 37 | 0.266 | 2 | 0.0287 | 0.453 |
| NOD1/2 Signaling Pathway | 38 | 0.273 | 2 | 0.0302 | 0.465 |
| Amine ligand-binding receptors | 38 | 0.273 | 2 | 0.0302 | 0.465 |

| | | | | | |
|---|---|---|---|---|---|
| Vasopressin-like receptors | 6 | 0.0431 | 1 | 0.0424 | 0.646 |

| MODULE 9 | | | | | |
|---|---|---|---|---|---|
| Pathway | Total | Expected | Hits | P.Value | FDR |
| Formation of Fibrin Clot (Clotting Cascade) | 29 | 0.104 | 11 | 2.86E-21 | 4.01E-18 |
| Hemostasis | 511 | 1.84 | 20 | 2.66E-19 | 1.87E-16 |
| Intrinsic Pathway | 17 | 0.0611 | 9 | 4.33E-19 | 2.02E-16 |
| Platelet activation, signaling and aggregation | 220 | 0.791 | 11 | 6.64E-11 | 2.33E-08 |
| Dissolution of Fibrin Clot | 10 | 0.0359 | 5 | 9.59E-11 | 2.69E-08 |
| Peptide ligand-binding receptors | 192 | 0.69 | 8 | 2.00E-07 | 4.68E-05 |
| G alpha (q) signalling events | 188 | 0.676 | 7 | 2.89E-06 | 0.00058 |
| Transport of gamma-carboxylated protein precursors from the endoplasmic reticulum to the Golgi apparatus | 9 | 0.0323 | 3 | 3.38E-06 | 0.000592 |
| Gamma-carboxylation of protein precursors | 10 | 0.0359 | 3 | 4.82E-06 | 0.000614 |
| Removal of aminoterminal propeptides from gamma-carboxylated proteins | 10 | 0.0359 | 3 | 4.82E-06 | 0.000614 |
| GP1b-IX-V activation signalling | 10 | 0.0359 | 3 | 4.82E-06 | 0.000614 |
| Thrombin signalling through proteinase activated receptors (PARs) | 34 | 0.122 | 4 | 5.54E-06 | 0.000632 |
| Gastrin-CREB signalling pathway via PKC and MAPK | 209 | 0.751 | 7 | 5.86E-06 | 0.000632 |
| Common Pathway | 11 | 0.0395 | 3 | 6.61E-06 | 0.000655 |
| Platelet Aggregation (Plug Formation) | 36 | 0.129 | 4 | 7.01E-06 | 0.000655 |
| Class A/1 (Rhodopsin-like receptors) | 312 | 1.12 | 8 | 7.93E-06 | 0.000685 |
| Platelet Adhesion to exposed collagen | 12 | 0.0431 | 3 | 8.79E-06 | 0.000685 |
| Gamma-carboxylation, transport, and amino-terminal cleavage of proteins | 12 | 0.0431 | 3 | 8.79E-06 | 0.000685 |
| GPCR ligand binding | 415 | 1.49 | 8 | 6.32E-05 | 0.00467 |
| PTM: gamma carboxylation, hypusine formation and arylsulfatase activation | 29 | 0.104 | 3 | 0.00014 | 0.00983 |
| Platelet degranulation | 89 | 0.32 | 4 | 0.000256 | 0.0171 |
| Response to elevated platelet cytosolic Ca2+ | 94 | 0.338 | 4 | 0.000315 | 0.0201 |
| Cell surface interactions at the vascular wall | 99 | 0.356 | 4 | 0.000385 | 0.0235 |
| Regulation of Complement cascade | 15 | 0.0539 | 2 | 0.00126 | 0.0738 |
| Regulation of Insulin-like Growth Factor (IGF) Transport and Uptake by Insulin-like Growth Factor Binding Proteins (IGFBPs) | 21 | 0.0755 | 2 | 0.00249 | 0.14 |
| Activation of Matrix Metalloproteinases | 31 | 0.111 | 2 | 0.0054 | 0.291 |
| GPCR downstream signaling | 812 | 2.92 | 8 | 0.00569 | 0.295 |
| Complement cascade | 33 | 0.119 | 2 | 0.00611 | 0.306 |
| Signaling by GPCR | 931 | 3.35 | 8 | 0.0129 | 0.625 |

| | | | | | |
|---|---|---|---|---|---|
| Signal Transduction | 1690 | 6.07 | 11 | 0.0229 | 1 |
| G alpha (i) signalling events | 184 | 0.661 | 3 | 0.0272 | 1 |
| Degradation of the extracellular matrix | 77 | 0.277 | 2 | 0.0308 | 1 |
| Relaxin receptors | 9 | 0.0323 | 1 | 0.0319 | 1 |
| Post-translational protein modification | 200 | 0.719 | 3 | 0.0337 | 1 |
| BMAL1:CLOCK/NPAS2 Activates Circadian Expression | 14 | 0.0503 | 1 | 0.0492 | 1 |

| MODULE 10 | | | | | |
|---|---|---|---|---|---|
| Pathway | Total | Expected | Hits | P.Value | FDR |
| Antigen Presentation: Folding, assembly and peptide loading of class I MHC | 25 | 0.0637 | 5 | 2.89E-09 | 4.05E-06 |
| Calnexin/calreticulin cycle | 11 | 0.028 | 4 | 9.39E-09 | 6.58E-06 |
| N-glycan trimming in the ER and Calnexin/Calreticulin cycle | 13 | 0.0331 | 4 | 2.03E-08 | 9.48E-06 |
| Collagen biosynthesis and modifying enzymes | 62 | 0.158 | 5 | 3.33E-07 | 0.000117 |
| Collagen formation | 85 | 0.216 | 5 | 1.63E-06 | 0.000457 |
| Extracellular matrix organization | 157 | 0.4 | 5 | 3.32E-05 | 0.00776 |
| Asparagine N-linked glycosylation | 86 | 0.219 | 4 | 5.37E-05 | 0.0108 |
| Chylomicron-mediated lipid transport | 12 | 0.0306 | 2 | 0.000397 | 0.0639 |
| Class I MHC mediated antigen processing & presentation | 267 | 0.68 | 5 | 0.00041 | 0.0639 |
| Unfolded Protein Response | 66 | 0.168 | 3 | 0.000568 | 0.0797 |
| Metabolism of proteins | 689 | 1.75 | 7 | 0.00093 | 0.118 |
| Lipoprotein metabolism | 22 | 0.056 | 2 | 0.00137 | 0.148 |
| Post-translational protein modification | 200 | 0.509 | 4 | 0.00137 | 0.148 |
| Lipid digestion, mobilization, and transport | 41 | 0.104 | 2 | 0.00472 | 0.473 |
| Signalling to STAT3 | 3 | 0.00764 | 1 | 0.00762 | 0.712 |
| Assembly of the RAD50-MRE11-NBS1 complex at DNA double-strand breaks | 4 | 0.0102 | 1 | 0.0101 | 0.837 |
| MRN complex relocalizes to nuclear foci | 4 | 0.0102 | 1 | 0.0101 | 0.837 |
| ER-Phagosome pathway | 63 | 0.16 | 2 | 0.0109 | 0.847 |
| Activation of Chaperone Genes by ATF6-alpha | 5 | 0.0127 | 1 | 0.0127 | 0.935 |
| ATM mediated phosphorylation of repair proteins | 6 | 0.0153 | 1 | 0.0152 | 1 |
| ATM mediated response to DNA double-strand break | 6 | 0.0153 | 1 | 0.0152 | 1 |
| Antigen processing-Cross presentation | 78 | 0.199 | 2 | 0.0164 | 1 |
| Recycling of eIF2:GDP | 8 | 0.0204 | 1 | 0.0202 | 1 |
| Activation of Chaperones by ATF6-alpha | 8 | 0.0204 | 1 | 0.0202 | 1 |
| Adaptive Immune System | 654 | 1.67 | 5 | 0.0202 | 1 |
| Recruitment of repair and signaling proteins to double-strand breaks | 9 | 0.0229 | 1 | 0.0227 | 1 |
| PERK regulated gene expression | 13 | 0.0331 | 1 | 0.0326 | 1 |
| Interleukin-6 signaling | 14 | 0.0356 | 1 | 0.0351 | 1 |
| Homologous recombination repair of replication-independent double-strand breaks | 16 | 0.0407 | 1 | 0.04 | 1 |

| | | | | | |
|---|---|---|---|---|---|
| Homologous Recombination Repair | 16 | 0.0407 | 1 | 0.04 | 1 |
| Signaling by FGFR1 fusion mutants | 20 | 0.0509 | 1 | 0.0498 | 1 |

| MODULE 11 | | | | | |
|---|---|---|---|---|---|
| Pathway | Total | Expected | Hits | P.Value | FDR |
| Global Genomic NER (GG-NER) | 36 | 0.124 | 12 | 1.01E-22 | 1.41E-19 |
| Nucleotide Excision Repair | 53 | 0.183 | 12 | 2.09E-20 | 9.30E-18 |
| Dual incision reaction in GG-NER | 23 | 0.0792 | 10 | 2.65E-20 | 9.30E-18 |
| Formation of incision complex in GG-NER | 23 | 0.0792 | 10 | 2.65E-20 | 9.30E-18 |
| DNA Repair | 117 | 0.403 | 13 | 7.35E-18 | 2.06E-15 |
| HIV Infection | 214 | 0.737 | 14 | 5.01E-16 | 1.17E-13 |
| S Phase | 122 | 0.42 | 12 | 9.13E-16 | 1.83E-13 |
| Synthesis of DNA | 95 | 0.327 | 11 | 3.14E-15 | 5.51E-13 |
| Cyclin E associated events during G1/S transition | 65 | 0.224 | 10 | 3.85E-15 | 6.00E-13 |
| Cyclin A:Cdk2-associated events at S phase entry | 66 | 0.227 | 10 | 4.53E-15 | 6.35E-13 |
| DNA Replication | 102 | 0.351 | 11 | 7.09E-15 | 9.03E-13 |
| Regulation of activated PAK-2p34 by proteasome mediated degradation | 48 | 0.165 | 9 | 1.76E-14 | 1.77E-12 |
| Cross-presentation of soluble exogenous antigens (endosomes) | 48 | 0.165 | 9 | 1.76E-14 | 1.77E-12 |
| Ubiquitin-dependent degradation of Cyclin D1 | 49 | 0.169 | 9 | 2.15E-14 | 1.77E-12 |
| CDK-mediated phosphorylation and removal of Cdc6 | 49 | 0.169 | 9 | 2.15E-14 | 1.77E-12 |
| Ubiquitin-dependent degradation of Cyclin D | 49 | 0.169 | 9 | 2.15E-14 | 1.77E-12 |
| Regulation of ornithine decarboxylase (ODC) | 49 | 0.169 | 9 | 2.15E-14 | 1.77E-12 |
| G1/S Transition | 113 | 0.389 | 11 | 2.27E-14 | 1.77E-12 |
| Vpu mediated degradation of CD4 | 50 | 0.172 | 9 | 2.62E-14 | 1.93E-12 |
| DNA Replication Pre-Initiation | 80 | 0.276 | 10 | 3.45E-14 | 2.24E-12 |
| M/G1 Transition | 80 | 0.276 | 10 | 3.45E-14 | 2.24E-12 |
| Ubiquitin Mediated Degradation of Phosphorylated Cdc25A | 52 | 0.179 | 9 | 3.83E-14 | 2.24E-12 |
| p53-Independent DNA Damage Response | 52 | 0.179 | 9 | 3.83E-14 | 2.24E-12 |
| p53-Independent G1/S DNA damage checkpoint | 52 | 0.179 | 9 | 3.83E-14 | 2.24E-12 |
| SCF-beta-TrCP mediated degradation of Emi1 | 53 | 0.183 | 9 | 4.61E-14 | 2.48E-12 |
| Autodegradation of the E3 ubiquitin ligase COP1 | 53 | 0.183 | 9 | 4.61E-14 | 2.48E-12 |
| Stabilization of p53 | 54 | 0.186 | 9 | 5.52E-14 | 2.76E-12 |
| Destabilization of mRNA by AUF1 (hnRNP D0) | 54 | 0.186 | 9 | 5.52E-14 | 2.76E-12 |
| Vif-mediated degradation of APOBEC3G | 55 | 0.189 | 9 | 6.59E-14 | 3.18E-12 |

| | | | | | |
|---|---|---|---|---|---|
| CDT1 association with the CDC6:ORC:origin complex | 57 | 0.196 | 9 | 9.28E-14 | 4.34E-12 |
| SCF(Skp2)-mediated degradation of p27/p21 | 58 | 0.2 | 9 | 1.10E-13 | 4.96E-12 |
| p53-Dependent G1/S DNA damage checkpoint | 59 | 0.203 | 9 | 1.29E-13 | 5.33E-12 |
| p53-Dependent G1 DNA Damage Response | 59 | 0.203 | 9 | 1.29E-13 | 5.33E-12 |
| Regulation of Apoptosis | 59 | 0.203 | 9 | 1.29E-13 | 5.33E-12 |
| G1/S DNA Damage Checkpoints | 62 | 0.214 | 9 | 2.07E-13 | 8.31E-12 |
| Assembly of the pre-replicative complex | 63 | 0.217 | 9 | 2.41E-13 | 9.15E-12 |
| ER-Phagosome pathway | 63 | 0.217 | 9 | 2.41E-13 | 9.15E-12 |
| Mitotic G1-G1/S phases | 140 | 0.482 | 11 | 2.53E-13 | 9.34E-12 |
| Degradation of beta-catenin by the destruction complex | 65 | 0.224 | 9 | 3.25E-13 | 1.14E-11 |
| Signaling by Wnt | 65 | 0.224 | 9 | 3.25E-13 | 1.14E-11 |
| Activation of NF-kappaB in B Cells | 66 | 0.227 | 9 | 3.75E-13 | 1.28E-11 |
| Autodegradation of Cdh1 by Cdh1:APC/C | 68 | 0.234 | 9 | 4.98E-13 | 1.66E-11 |
| Switching of origins to a post-replicative state | 69 | 0.238 | 9 | 5.72E-13 | 1.82E-11 |
| Orc1 removal from chromatin | 69 | 0.238 | 9 | 5.72E-13 | 1.82E-11 |
| Removal of licensing factors from origins | 71 | 0.245 | 9 | 7.48E-13 | 2.23E-11 |
| Regulation of DNA replication | 71 | 0.245 | 9 | 7.48E-13 | 2.23E-11 |
| APC/C:Cdc20 mediated degradation of Securin | 71 | 0.245 | 9 | 7.48E-13 | 2.23E-11 |
| Cdc20:Phospho-APC/C mediated degradation of Cyclin A | 76 | 0.262 | 9 | 1.42E-12 | 3.98E-11 |
| APC/C:Cdh1 mediated degradation of Cdc20 and other APC/C:Cdh1 targeted proteins in late mitosis/early G1 | 76 | 0.262 | 9 | 1.42E-12 | 3.98E-11 |
| APC/C:Cdc20 mediated degradation of mitotic proteins | 76 | 0.262 | 9 | 1.42E-12 | 3.98E-11 |
| Activation of APC/C and APC/C:Cdc20 mediated degradation of mitotic proteins | 77 | 0.265 | 9 | 1.60E-12 | 4.41E-11 |
| Antigen processing-Cross presentation | 78 | 0.269 | 9 | 1.81E-12 | 4.88E-11 |
| Regulation of APC/C activators between G1/S and early anaphase | 84 | 0.289 | 9 | 3.61E-12 | 9.55E-11 |
| Regulation of mRNA Stability by Proteins that Bind AU-rich Elements | 88 | 0.303 | 9 | 5.56E-12 | 1.44E-10 |
| APC/C-mediated degradation of cell cycle proteins | 89 | 0.307 | 9 | 6.17E-12 | 1.55E-10 |
| Regulation of mitotic cell cycle | 89 | 0.307 | 9 | 6.17E-12 | 1.55E-10 |
| Cell Cycle, Mitotic | 411 | 1.42 | 13 | 9.84E-11 | 2.42E-09 |
| Transcription-coupled NER (TC-NER) | 47 | 0.162 | 7 | 1.21E-10 | 2.93E-09 |
| Cell Cycle Checkpoints | 131 | 0.451 | 9 | 2.12E-10 | 5.03E-09 |
| Host Interactions of HIV factors | 141 | 0.486 | 9 | 4.11E-10 | 9.61E-09 |

| | | | | | |
|---|---|---|---|---|---|
| Apoptosis | 158 | 0.544 | 9 | 1.14E-09 | 2.62E-08 |
| Cell Cycle | 508 | 1.75 | 13 | 1.39E-09 | 3.14E-08 |
| Downstream Signaling Events Of B Cell Receptor (BCR) | 173 | 0.596 | 9 | 2.56E-09 | 5.69E-08 |
| Separation of Sister Chromatids | 186 | 0.641 | 9 | 4.86E-09 | 1.06E-07 |
| Metabolism of amino acids and derivatives | 190 | 0.654 | 9 | 5.87E-09 | 1.27E-07 |
| Mitotic M-M/G1 phases | 266 | 0.916 | 10 | 6.16E-09 | 1.31E-07 |
| Mitotic Anaphase | 198 | 0.682 | 9 | 8.44E-09 | 1.77E-07 |
| Mitotic Metaphase and Anaphase | 199 | 0.685 | 9 | 8.82E-09 | 1.79E-07 |
| Signaling by the B Cell Receptor (BCR) | 199 | 0.685 | 9 | 8.82E-09 | 1.79E-07 |
| Antigen processing: Ubiquitination & Proteasome degradation | 224 | 0.772 | 9 | 2.49E-08 | 4.99E-07 |
| M Phase | 233 | 0.803 | 9 | 3.51E-08 | 6.93E-07 |
| Dual incision reaction in TC-NER | 31 | 0.107 | 5 | 4.88E-08 | 9.38E-07 |
| Formation of transcription-coupled NER (TC-NER) repair complex | 31 | 0.107 | 5 | 4.88E-08 | 9.38E-07 |
| Class I MHC mediated antigen processing & presentation | 267 | 0.92 | 9 | 1.14E-07 | 2.16E-06 |
| Disease | 945 | 3.26 | 14 | 2.77E-07 | 5.19E-06 |
| Metabolism of mRNA | 317 | 1.09 | 9 | 4.96E-07 | 9.15E-06 |
| RNA Polymerase I Chain Elongation | 21 | 0.0723 | 4 | 6.16E-07 | 1.12E-05 |
| RNA Polymerase I Promoter Escape | 22 | 0.0758 | 4 | 7.51E-07 | 1.35E-05 |
| Metabolism of RNA | 339 | 1.17 | 9 | 8.76E-07 | 1.55E-05 |
| RNA Polymerase I Transcription Initiation | 23 | 0.0792 | 4 | 9.07E-07 | 1.57E-05 |
| RNA Polymerase I Transcription Termination | 23 | 0.0792 | 4 | 9.07E-07 | 1.57E-05 |
| RNA Polymerase I Promoter Clearance | 24 | 0.0827 | 4 | 1.09E-06 | 1.86E-05 |
| RNA Polymerase I Transcription | 26 | 0.0896 | 4 | 1.52E-06 | 2.57E-05 |
| RNA Pol II CTD phosphorylation and interaction with CE | 28 | 0.0965 | 4 | 2.07E-06 | 3.42E-05 |
| RNA Pol II CTD phosphorylation and interaction with CE | 28 | 0.0965 | 4 | 2.07E-06 | 3.42E-05 |
| mRNA Capping | 30 | 0.103 | 4 | 2.76E-06 | 4.51E-05 |
| Formation of the Early Elongation Complex | 34 | 0.117 | 4 | 4.63E-06 | 7.38E-05 |
| Formation of the HIV-1 Early Elongation Complex | 34 | 0.117 | 4 | 4.63E-06 | 7.38E-05 |
| RNA Polymerase II Promoter Escape | 41 | 0.141 | 4 | 9.96E-06 | 0.000149 |
| RNA Polymerase II Transcription Pre-Initiation And Promoter Opening | 41 | 0.141 | 4 | 9.96E-06 | 0.000149 |
| RNA Polymerase II Transcription Initiation | 41 | 0.141 | 4 | 9.96E-06 | 0.000149 |

| | | | | | |
|---|---|---|---|---|---|
| RNA Polymerase II Transcription Initiation And Promoter Clearance | 41 | 0.141 | 4 | 9.96E-06 | 0.000149 |
| HIV-1 Transcription Initiation | 41 | 0.141 | 4 | 9.96E-06 | 0.000149 |
| RNA Polymerase II HIV-1 Promoter Escape | 41 | 0.141 | 4 | 9.96E-06 | 0.000149 |
| DNA Damage Recognition in GG-NER | 2 | 0.00689 | 2 | 1.14E-05 | 0.000168 |
| Gene Expression | 1090 | 3.74 | 13 | 1.20E-05 | 0.000175 |
| Tat-mediated elongation of the HIV-1 transcript | 45 | 0.155 | 4 | 1.45E-05 | 0.000206 |
| Formation of HIV-1 elongation complex containing HIV-1 Tat | 45 | 0.155 | 4 | 1.45E-05 | 0.000206 |
| HIV-1 Transcription Elongation | 45 | 0.155 | 4 | 1.45E-05 | 0.000206 |
| RNA Polymerase II Transcription Elongation | 46 | 0.158 | 4 | 1.59E-05 | 0.000218 |
| Formation of RNA Pol II elongation complex | 46 | 0.158 | 4 | 1.59E-05 | 0.000218 |
| Formation of HIV-1 elongation complex in the absence of HIV-1 Tat | 46 | 0.158 | 4 | 1.59E-05 | 0.000218 |
| RNA Polymerase I, RNA Polymerase III, and Mitochondrial Transcription | 59 | 0.203 | 4 | 4.30E-05 | 0.000585 |
| RNA Polymerase II Pre-transcription Events | 62 | 0.214 | 4 | 5.23E-05 | 0.000705 |
| Transcription of the HIV genome | 64 | 0.22 | 4 | 5.93E-05 | 0.000792 |
| HIV Life Cycle | 128 | 0.441 | 5 | 6.11E-05 | 0.000808 |
| Adaptive Immune System | 654 | 2.25 | 9 | 0.000182 | 0.00238 |
| RNA Polymerase II Transcription | 107 | 0.369 | 4 | 0.000437 | 0.00567 |
| Late Phase of HIV Life Cycle | 108 | 0.372 | 4 | 0.000452 | 0.00582 |
| Repair synthesis of patch ~27-30 bases long by DNA polymerase | 15 | 0.0517 | 2 | 0.00116 | 0.0146 |
| Repair synthesis for gap-filling by DNA polymerase in TC-NER | 15 | 0.0517 | 2 | 0.00116 | 0.0146 |
| mRNA Processing | 140 | 0.482 | 4 | 0.0012 | 0.0151 |
| Gap-filling DNA repair synthesis and ligation in GG-NER | 16 | 0.0551 | 2 | 0.00132 | 0.0161 |
| Gap-filling DNA repair synthesis and ligation in TC-NER | 16 | 0.0551 | 2 | 0.00132 | 0.0161 |
| Cytosolic Iron-sulfur Cluster Assembly | 16 | 0.0551 | 2 | 0.00132 | 0.0161 |
| Transcription | 149 | 0.513 | 4 | 0.00152 | 0.0183 |
| Telomere C-strand (Lagging Strand) Synthesis | 22 | 0.0758 | 2 | 0.00251 | 0.0301 |
| Extension of Telomeres | 24 | 0.0827 | 2 | 0.00299 | 0.0355 |
| Metabolism | 1490 | 5.13 | 11 | 0.00599 | 0.0705 |
| Immune System | 1140 | 3.92 | 9 | 0.00984 | 0.115 |

| MODULE 12 | | | | | |
|---|---|---|---|---|---|
| Pathway | Total | Expected | Hits | P.Value | FDR |
| FCGR activation | 15 | 0.0225 | 4 | 3.44E-09 | 4.82E-06 |
| Signaling by Interleukins | 116 | 0.174 | 5 | 3.41E-07 | 0.000239 |
| PECAM1 interactions | 13 | 0.0195 | 3 | 6.87E-07 | 0.000321 |
| Regulation of KIT signaling | 17 | 0.0255 | 3 | 1.63E-06 | 0.00057 |
| Regulation of signaling by CBL | 22 | 0.0329 | 3 | 3.67E-06 | 0.00102 |
| CTLA4 inhibitory signaling | 24 | 0.0359 | 3 | 4.82E-06 | 0.00102 |
| Fcgamma receptor (FCGR) dependent phagocytosis | 86 | 0.129 | 4 | 5.08E-06 | 0.00102 |
| CD28 co-stimulation | 30 | 0.0449 | 3 | 9.62E-06 | 0.00169 |
| Cytokine Signaling in Immune system | 286 | 0.428 | 5 | 2.94E-05 | 0.00458 |
| Innate Immune System | 521 | 0.78 | 6 | 3.51E-05 | 0.00492 |
| Interleukin-3, 5 and GM-CSF signaling | 51 | 0.0764 | 3 | 4.85E-05 | 0.00618 |
| Nef and signal transduction | 8 | 0.012 | 2 | 5.63E-05 | 0.00657 |
| Costimulation by the CD28 family | 68 | 0.102 | 3 | 0.000115 | 0.0124 |
| Signaling by the B Cell Receptor (BCR) | 199 | 0.298 | 4 | 0.00014 | 0.014 |
| Immune System | 1140 | 1.7 | 7 | 0.000305 | 0.0285 |
| Cell surface interactions at the vascular wall | 99 | 0.148 | 3 | 0.000352 | 0.0308 |
| The role of Nef in HIV-1 replication and disease pathogenesis | 31 | 0.0464 | 2 | 0.000917 | 0.0729 |
| Antigen Activates B Cell Receptor Leading to Generation of Second Messengers | 32 | 0.0479 | 2 | 0.000978 | 0.0729 |
| Signaling by SCF-KIT | 142 | 0.213 | 3 | 0.00101 | 0.0729 |
| GPVI-mediated activation cascade | 33 | 0.0494 | 2 | 0.00104 | 0.0729 |
| Adaptive Immune System | 654 | 0.979 | 5 | 0.00147 | 0.0982 |
| Downstream TCR signaling | 48 | 0.0719 | 2 | 0.0022 | 0.14 |
| TCR signaling | 65 | 0.0973 | 2 | 0.00399 | 0.243 |
| Hemostasis | 511 | 0.765 | 4 | 0.0049 | 0.286 |
| Nef Mediated CD4 Down-regulation | 9 | 0.0135 | 1 | 0.0134 | 0.752 |
| IRAK2 mediated activation of TAK1 complex upon TLR7/8 or 9 stimulation | 10 | 0.015 | 1 | 0.0149 | 0.773 |
| IRAK2 mediated activation of TAK1 complex | 10 | 0.015 | 1 | 0.0149 | 0.773 |
| Host Interactions of HIV factors | 141 | 0.211 | 2 | 0.0178 | 0.834 |
| Platelet Adhesion to exposed collagen | 12 | 0.018 | 1 | 0.0178 | 0.834 |

| | | | | | |
|---|---|---|---|---|---|
| CD28 dependent Vav1 pathway | 12 | 0.018 | 1 | 0.0178 | 0.834 |
| Activation of Rac | 15 | 0.0225 | 1 | 0.0223 | 0.978 |
| Downstream signal transduction | 163 | 0.244 | 2 | 0.0234 | 0.978 |
| Signaling by ERBB2 | 164 | 0.246 | 2 | 0.0237 | 0.978 |
| TRAF6 mediated induction of TAK1 complex | 16 | 0.024 | 1 | 0.0237 | 0.978 |
| Downstream Signaling Events Of B Cell Receptor (BCR) | 173 | 0.259 | 2 | 0.0262 | 1 |
| Translocation of ZAP-70 to Immunological synapse | 18 | 0.027 | 1 | 0.0267 | 1 |
| CD28 dependent PI3K/Akt signaling | 19 | 0.0285 | 1 | 0.0281 | 1 |
| Platelet sensitization by LDL | 19 | 0.0285 | 1 | 0.0281 | 1 |
| Phosphorylation of CD3 and TCR zeta chains | 20 | 0.03 | 1 | 0.0296 | 1 |
| JNK (c-Jun kinases) phosphorylation and activation mediated by activated human TAK1 | 20 | 0.03 | 1 | 0.0296 | 1 |
| Signaling by PDGF | 189 | 0.283 | 2 | 0.0309 | 1 |
| TAK1 activates NFkB by phosphorylation and activation of IKKs complex | 22 | 0.0329 | 1 | 0.0325 | 1 |
| Growth hormone receptor signaling | 23 | 0.0344 | 1 | 0.0339 | 1 |
| activated TAK1 mediates p38 MAPK activation | 23 | 0.0344 | 1 | 0.0339 | 1 |
| Nef-mediates down modulation of cell surface receptors by recruiting them to clathrin adapters | 24 | 0.0359 | 1 | 0.0354 | 1 |
| Nephrin interactions | 24 | 0.0359 | 1 | 0.0354 | 1 |
| PD-1 signaling | 25 | 0.0374 | 1 | 0.0368 | 1 |
| HIV Infection | 214 | 0.321 | 2 | 0.0388 | 1 |
| Platelet activation, signaling and aggregation | 220 | 0.329 | 2 | 0.0409 | 1 |
| Generation of second messenger molecules | 31 | 0.0464 | 1 | 0.0455 | 1 |
| Signaling by Robo receptor | 34 | 0.0509 | 1 | 0.0498 | 1 |

| MODULE 13 | | | | | |
|---|---|---|---|---|---|
| Pathway | Total | Expected | Hits | P.Value | FDR |
| Glycolysis | 30 | 0.0809 | 12 | 9.75E-26 | 1.37E-22 |
| Gluconeogenesis | 35 | 0.0944 | 11 | 4.44E-22 | 3.11E-19 |
| Glucose metabolism | 70 | 0.189 | 12 | 1.16E-20 | 5.42E-18 |
| Metabolism of carbohydrates | 258 | 0.696 | 13 | 2.29E-15 | 8.04E-13 |
| Metabolism | 1490 | 4.01 | 13 | 8.73E-06 | 0.00245 |
| Fructose catabolism | 2 | 0.00539 | 1 | 0.00538 | 1 |
| Nuclear Receptor transcription pathway | 53 | 0.143 | 2 | 0.00872 | 1 |
| Facilitative Na+-independent glucose transporters | 12 | 0.0323 | 1 | 0.0319 | 1 |
| Golgi Cisternae Pericentriolar Stack Reorganization | 14 | 0.0377 | 1 | 0.0371 | 1 |
| Mitotic Prophase | 36 | 0.097 | 1 | 0.0928 | 1 |

| MODULE 14 | | | | | |
|---|---|---|---|---|---|
| Pathway | Total | Expected | Hits | P.Value | FDR |
| Synthesis and interconversion of nucleotide di- and triphosphates | 20 | 0.0479 | 7 | 7.46E-15 | 1.05E-11 |
| Metabolism of nucleotides | 81 | 0.194 | 8 | 3.92E-12 | 2.75E-09 |
| RIG-I/MDA5 mediated induction of IFN-alpha/beta pathways | 67 | 0.161 | 4 | 1.54E-05 | 0.00719 |
| IKK complex recruitment mediated by RIP1 | 25 | 0.0599 | 3 | 2.52E-05 | 0.00882 |
| TRIF-mediated TLR3/TLR4 signaling | 87 | 0.208 | 4 | 4.34E-05 | 0.0091 |
| MyD88-independent cascade | 88 | 0.211 | 4 | 4.54E-05 | 0.0091 |
| Toll Like Receptor 3 (TLR3) Cascade | 88 | 0.211 | 4 | 4.54E-05 | 0.0091 |
| Activated TLR4 signalling | 100 | 0.24 | 4 | 7.51E-05 | 0.0131 |
| Toll Like Receptor 4 (TLR4) Cascade | 103 | 0.247 | 4 | 8.43E-05 | 0.0131 |
| Interleukin-1 signaling | 45 | 0.108 | 3 | 0.000151 | 0.0211 |
| Toll-Like Receptors Cascades | 123 | 0.295 | 4 | 0.000168 | 0.0214 |
| Downstream TCR signaling | 48 | 0.115 | 3 | 0.000183 | 0.0214 |
| Regulated proteolysis of p75NTR | 10 | 0.024 | 2 | 0.00024 | 0.0258 |
| Nucleotide-binding domain, leucine rich repeat containing receptor (NLR) signaling pathways | 55 | 0.132 | 3 | 0.000275 | 0.0275 |
| NF-kB is activated and signals survival | 13 | 0.0312 | 2 | 0.000414 | 0.0377 |
| TCR signaling | 65 | 0.156 | 3 | 0.00045 | 0.0377 |
| p75NTR signals via NF-kB | 16 | 0.0383 | 2 | 0.000634 | 0.0377 |
| IRAK1 recruits IKK complex upon TLR7/8 or 9 stimulation | 16 | 0.0383 | 2 | 0.000634 | 0.0377 |
| IRAK1 recruits IKK complex | 16 | 0.0383 | 2 | 0.000634 | 0.0377 |
| TRAF6 mediated NF-kB activation | 16 | 0.0383 | 2 | 0.000634 | 0.0377 |
| Toll Like Receptor 10 (TLR10) Cascade | 74 | 0.177 | 3 | 0.00066 | 0.0377 |
| Toll Like Receptor 5 (TLR5) Cascade | 74 | 0.177 | 3 | 0.00066 | 0.0377 |
| MyD88 cascade initiated on plasma membrane | 74 | 0.177 | 3 | 0.00066 | 0.0377 |
| TRAF6 mediated induction of NFkB and MAP kinases upon TLR7/8 or 9 activation | 76 | 0.182 | 3 | 0.000713 | 0.0377 |
| TRAF6 mediated IRF7 activation in TLR7/8 or 9 signaling | 17 | 0.0407 | 2 | 0.000717 | 0.0377 |
| Toll Like Receptor 7/8 (TLR7/8) Cascade | 77 | 0.185 | 3 | 0.000741 | 0.0377 |
| MyD88 dependent cascade initiated on endosome | 77 | 0.185 | 3 | 0.000741 | 0.0377 |
| Toll Like Receptor 9 (TLR9) Cascade | 79 | 0.189 | 3 | 0.000799 | 0.0377 |
| MyD88:Mal cascade initiated on plasma membrane | 81 | 0.194 | 3 | 0.00086 | 0.0377 |

| | | | | | |
|---|---|---|---|---|---|
| Toll Like Receptor TLR1:TLR2 Cascade | 81 | 0.194 | 3 | 0.00086 | 0.0377 |
| Toll Like Receptor TLR6:TLR2 Cascade | 81 | 0.194 | 3 | 0.00086 | 0.0377 |
| Toll Like Receptor 2 (TLR2) Cascade | 81 | 0.194 | 3 | 0.00086 | 0.0377 |
| Innate Immune System | 521 | 1.25 | 6 | 0.000891 | 0.0379 |
| TAK1 activates NFkB by phosphorylation and activation of IKKs complex | 22 | 0.0527 | 2 | 0.00121 | 0.0499 |
| Signaling by Interleukins | 116 | 0.278 | 3 | 0.00243 | 0.0958 |
| Immune System | 1140 | 2.73 | 8 | 0.00246 | 0.0958 |
| Negative regulators of RIG-I/MDA5 signaling | 33 | 0.0791 | 2 | 0.00272 | 0.103 |
| Adaptive Immune System | 654 | 1.57 | 6 | 0.00293 | 0.108 |
| Class I MHC mediated antigen processing & presentation | 267 | 0.64 | 4 | 0.00311 | 0.112 |
| NOD1/2 Signaling Pathway | 38 | 0.0911 | 2 | 0.0036 | 0.126 |
| Cytokine Signaling in Immune system | 286 | 0.685 | 4 | 0.00399 | 0.136 |
| TRAF6 Mediated Induction of proinflammatory cytokines | 62 | 0.149 | 2 | 0.00936 | 0.313 |
| Metabolism | 1490 | 3.57 | 8 | 0.0136 | 0.436 |
| Synthesis of 12-eicosatetraenoic acid derivatives | 6 | 0.0144 | 1 | 0.0143 | 0.436 |
| Synthesis of 5-eicosatetraenoic acids | 6 | 0.0144 | 1 | 0.0143 | 0.436 |
| Synthesis of 15-eicosatetraenoic acid derivatives | 6 | 0.0144 | 1 | 0.0143 | 0.436 |
| Antigen processing: Ubiquitination & Proteasome degradation | 224 | 0.537 | 3 | 0.0151 | 0.45 |
| p75 NTR receptor-mediated signalling | 85 | 0.204 | 2 | 0.0171 | 0.5 |
| Cross-presentation of particulate exogenous antigens (phagosomes) | 8 | 0.0192 | 1 | 0.019 | 0.544 |
| Displacement of DNA glycosylase by APE1 | 9 | 0.0216 | 1 | 0.0214 | 0.599 |
| Base-free sugar-phosphate removal via the single-nucleotide replacement pathway | 10 | 0.024 | 1 | 0.0237 | 0.628 |
| IRAK2 mediated activation of TAK1 complex upon TLR7/8 or 9 stimulation | 10 | 0.024 | 1 | 0.0237 | 0.628 |
| IRAK2 mediated activation of TAK1 complex | 10 | 0.024 | 1 | 0.0237 | 0.628 |
| Purine catabolism | 11 | 0.0264 | 1 | 0.0261 | 0.664 |
| RIP-mediated NFkB activation via DAI | 11 | 0.0264 | 1 | 0.0261 | 0.664 |
| Resolution of AP sites via the single-nucleotide replacement pathway | 12 | 0.0288 | 1 | 0.0284 | 0.711 |
| DAI mediated induction of type I IFNs | 13 | 0.0312 | 1 | 0.0307 | 0.743 |
| p75NTR recruits signalling complexes | 13 | 0.0312 | 1 | 0.0307 | 0.743 |
| The NLRP3 inflammasome | 14 | 0.0335 | 1 | 0.0331 | 0.786 |
| NRIF signals cell death from the nucleus | 15 | 0.0359 | 1 | 0.0354 | 0.827 |

| | | | | | |
|---|---|---|---|---|---|
| G1/S-Specific Transcription | 16 | 0.0383 | 1 | 0.0377 | 0.853 |
| TRAF6 mediated induction of TAK1 complex | 16 | 0.0383 | 1 | 0.0377 | 0.853 |
| Resolution of AP sites via the multiple-nucleotide patch replacement pathway | 17 | 0.0407 | 1 | 0.04 | 0.863 |
| Removal of DNA patch containing abasic residue | 17 | 0.0407 | 1 | 0.04 | 0.863 |
| Regulation of IFNG signaling | 17 | 0.0407 | 1 | 0.04 | 0.863 |
| Resolution of Abasic Sites (AP sites) | 19 | 0.0455 | 1 | 0.0446 | 0.907 |
| Base Excision Repair | 19 | 0.0455 | 1 | 0.0446 | 0.907 |
| Inflammasomes | 19 | 0.0455 | 1 | 0.0446 | 0.907 |
| Cytosolic sensors of pathogen-associated DNA | 19 | 0.0455 | 1 | 0.0446 | 0.907 |
| Oxygen-dependent Proline Hydroxylation of Hypoxia-inducible Factor Alpha | 20 | 0.0479 | 1 | 0.0469 | 0.926 |
| JNK (c-Jun kinases) phosphorylation and activation mediated by activated human TAK1 | 20 | 0.0479 | 1 | 0.0469 | 0.926 |

| MODULE 15 | | | | | |
|---|---|---|---|---|---|
| Pathway | Total | Expected | Hits | P.Value | FDR |
| Rho GTPase cycle | 123 | 0.332 | 14 | 7.01E-22 | 4.91E-19 |
| Signaling by Rho GTPases | 123 | 0.332 | 14 | 7.01E-22 | 4.91E-19 |
| Sema4D induced cell migration and growth-cone collapse | 29 | 0.0782 | 4 | 8.42E-07 | 0.000394 |
| Sema4D in semaphorin signaling | 34 | 0.0917 | 4 | 1.63E-06 | 0.000572 |
| Signal Transduction | 1690 | 4.55 | 14 | 4.41E-06 | 0.00124 |
| Semaphorin interactions | 72 | 0.194 | 4 | 3.39E-05 | 0.00793 |
| G alpha (12/13) signalling events | 80 | 0.216 | 4 | 5.15E-05 | 0.0103 |
| GPVI-mediated activation cascade | 33 | 0.089 | 3 | 8.53E-05 | 0.015 |
| Axon guidance | 292 | 0.787 | 4 | 0.00674 | 1 |
| Axonal growth stimulation | 4 | 0.0108 | 1 | 0.0107 | 1 |
| Platelet activation, signaling and aggregation | 220 | 0.593 | 3 | 0.02 | 1 |
| Developmental Biology | 417 | 1.12 | 4 | 0.0228 | 1 |
| Axonal growth inhibition (RHOA activation) | 9 | 0.0243 | 1 | 0.024 | 1 |
| p75NTR regulates axonogenesis | 10 | 0.027 | 1 | 0.0267 | 1 |
| Golgi Cisternae Pericentriolar Stack Reorganization | 14 | 0.0377 | 1 | 0.0371 | 1 |

| MODULE 16 | | | | | |
|---|---|---|---|---|---|
| Pathway | Total | Expected | Hits | P.Value | FDR |
| TGF-beta receptor signaling activates SMADs | 30 | 0.0674 | 5 | 3.76E-09 | 5.27E-06 |
| TGF-beta receptor signaling in EMT (epithelial to mesenchymal transition) | 17 | 0.0382 | 4 | 3.86E-08 | 2.71E-05 |
| Signaling by BMP | 24 | 0.0539 | 4 | 1.71E-07 | 7.98E-05 |
| Downregulation of TGF-beta receptor signaling | 27 | 0.0607 | 4 | 2.81E-07 | 8.51E-05 |
| Signaling by TGF-beta Receptor Complex | 70 | 0.157 | 5 | 3.03E-07 | 8.51E-05 |
| Molecules associated with elastic fibres | 38 | 0.0854 | 2 | 0.00316 | 0.739 |
| Elastic fibre formation | 45 | 0.101 | 2 | 0.00441 | 0.789 |
| Signal Transduction | 1690 | 3.79 | 9 | 0.0045 | 0.789 |
| Stimuli-sensing channels | 63 | 0.142 | 2 | 0.0085 | 1 |
| Platelet activation, signaling and aggregation | 220 | 0.494 | 3 | 0.012 | 1 |
| Release of eIF4E | 6 | 0.0135 | 1 | 0.0134 | 1 |
| Platelet degranulation | 89 | 0.2 | 2 | 0.0165 | 1 |
| Response to elevated platelet cytosolic Ca2+ | 94 | 0.211 | 2 | 0.0183 | 1 |
| S6K1-mediated signalling | 9 | 0.0202 | 1 | 0.02 | 1 |
| Regulation of Signaling by NODAL | 9 | 0.0202 | 1 | 0.02 | 1 |
| mTORC1-mediated signalling | 11 | 0.0247 | 1 | 0.0245 | 1 |
| Elevation of cytosolic Ca2+ levels | 12 | 0.027 | 1 | 0.0266 | 1 |
| Keratan sulfate degradation | 12 | 0.027 | 1 | 0.0266 | 1 |
| Signaling by Activin | 12 | 0.027 | 1 | 0.0266 | 1 |
| Ion channel transport | 128 | 0.288 | 2 | 0.0325 | 1 |
| Regulation of AMPK activity via LKB1 | 16 | 0.0359 | 1 | 0.0354 | 1 |
| Signaling by NODAL | 17 | 0.0382 | 1 | 0.0376 | 1 |
| Energy dependent regulation of mTOR by LKB1-AMPK | 19 | 0.0427 | 1 | 0.0419 | 1 |
| cGMP effects | 21 | 0.0472 | 1 | 0.0462 | 1 |
| Extracellular matrix organization | 157 | 0.353 | 2 | 0.0472 | 1 |
| Platelet calcium homeostasis | 22 | 0.0494 | 1 | 0.0483 | 1 |

| MODULE 17 | | | | | |
|---|---|---|---|---|---|
| Pathway | Total | Expected | Hits | P.Value | FDR |
| p75 NTR receptor-mediated signalling | 85 | 0.165 | 8 | 6.03E-13 | 8.45E-10 |
| p75NTR regulates axonogenesis | 10 | 0.0195 | 5 | 2.92E-12 | 2.05E-09 |
| Signalling by NGF | 290 | 0.565 | 10 | 5.23E-12 | 2.44E-09 |
| Axonal growth inhibition (RHOA activation) | 9 | 0.0175 | 4 | 1.08E-09 | 3.80E-07 |
| p75NTR recruits signalling complexes | 13 | 0.0253 | 4 | 6.12E-09 | 1.72E-06 |
| NRIF signals cell death from the nucleus | 15 | 0.0292 | 4 | 1.17E-08 | 2.72E-06 |
| p75NTR signals via NF-kB | 16 | 0.0312 | 4 | 1.55E-08 | 3.11E-06 |
| Activation of TRKA receptors | 6 | 0.0117 | 3 | 1.15E-07 | 2.01E-05 |
| NF-kB is activated and signals survival | 13 | 0.0253 | 3 | 1.63E-06 | 0.000254 |
| TRKA activation by NGF | 2 | 0.00389 | 2 | 3.50E-06 | 0.000491 |
| Cell death signalling via NRAGE, NRIF and NADE | 62 | 0.121 | 4 | 4.53E-06 | 0.000577 |
| Ceramide signalling | 3 | 0.00584 | 2 | 1.05E-05 | 0.00105 |
| NFG and proNGF binds to p75NTR | 3 | 0.00584 | 2 | 1.05E-05 | 0.00105 |
| Signalling to STAT3 | 3 | 0.00584 | 2 | 1.05E-05 | 0.00105 |
| Signal Transduction | 1690 | 3.29 | 11 | 1.22E-05 | 0.00114 |
| Axonal growth stimulation | 4 | 0.00779 | 2 | 2.10E-05 | 0.00184 |
| NGF-independant TRKA activation | 5 | 0.00973 | 2 | 3.49E-05 | 0.00288 |
| p75NTR negatively regulates cell cycle via SC1 | 6 | 0.0117 | 2 | 5.23E-05 | 0.00407 |
| NADE modulates death signalling | 7 | 0.0136 | 2 | 7.31E-05 | 0.00539 |
| Regulated proteolysis of p75NTR | 10 | 0.0195 | 2 | 0.000156 | 0.0109 |
| Retrograde neurotrophin signalling | 13 | 0.0253 | 2 | 0.00027 | 0.018 |
| Signalling to p38 via RIT and RIN | 15 | 0.0292 | 2 | 0.000362 | 0.0231 |
| ARMS-mediated activation | 16 | 0.0312 | 2 | 0.000414 | 0.0252 |
| Frs2-mediated activation | 18 | 0.035 | 2 | 0.000526 | 0.0307 |
| Prolonged ERK activation events | 20 | 0.0389 | 2 | 0.000652 | 0.0366 |
| Signalling to RAS | 27 | 0.0526 | 2 | 0.0012 | 0.0644 |
| PLC-gamma1 signalling | 37 | 0.072 | 2 | 0.00224 | 0.112 |
| Signalling to ERKs | 37 | 0.072 | 2 | 0.00224 | 0.112 |
| NRAGE signals death through JNK | 45 | 0.0876 | 2 | 0.0033 | 0.154 |

| | | | | | |
|---|---|---|---|---|---|
| Interleukin-1 signaling | 45 | 0.0876 | 2 | 0.0033 | 0.154 |
| NGF signalling via TRKA from the plasma membrane | 207 | 0.403 | 3 | 0.00667 | 0.302 |
| NGF processing | 4 | 0.00779 | 1 | 0.00777 | 0.34 |
| PI3K/AKT activation | 106 | 0.206 | 2 | 0.0174 | 0.717 |
| A third proteolytic cleavage releases NICD | 9 | 0.0175 | 1 | 0.0174 | 0.717 |
| Signaling by Interleukins | 116 | 0.226 | 2 | 0.0206 | 0.804 |
| Signaling by NOTCH3 | 11 | 0.0214 | 1 | 0.0212 | 0.804 |
| Signaling by NOTCH4 | 11 | 0.0214 | 1 | 0.0212 | 0.804 |
| Signaling by NOTCH2 | 18 | 0.035 | 1 | 0.0345 | 1 |
| NOTCH2 Activation and Transmission of Signal to the Nucleus | 18 | 0.035 | 1 | 0.0345 | 1 |
| JNK (c-Jun kinases) phosphorylation and activation mediated by activated human TAK1 | 20 | 0.0389 | 1 | 0.0383 | 1 |
| TAK1 activates NFkB by phosphorylation and activation of IKKs complex | 22 | 0.0428 | 1 | 0.042 | 1 |
| activated TAK1 mediates p38 MAPK activation | 23 | 0.0448 | 1 | 0.0439 | 1 |
| Activation of Gene Expression by SREBP (SREBF) | 26 | 0.0506 | 1 | 0.0495 | 1 |

| MODULE 18 | | | | | |
|---|---|---|---|---|---|
| Pathway | Total | Expected | Hits | P.Value | FDR |
| Downregulation of TGF-beta receptor signaling | 27 | 0.00809 | 1 | 0.00807 | 1 |
| TGF-beta receptor signaling activates SMADs | 30 | 0.00899 | 1 | 0.00897 | 1 |
| Deposition of New CENPA-containing Nucleosomes at the Centromere | 65 | 0.0195 | 1 | 0.0194 | 1 |
| Nucleosome assembly | 65 | 0.0195 | 1 | 0.0194 | 1 |
| Signaling by TGF-beta Receptor Complex | 70 | 0.021 | 1 | 0.0209 | 1 |
| Chromosome Maintenance | 124 | 0.0371 | 1 | 0.0368 | 1 |
| Cell Cycle | 508 | 0.152 | 1 | 0.146 | 1 |
| Signal Transduction | 1690 | 0.506 | 1 | 0.442 | 1 |

| MODULE 19 | | | | | |
|---|---|---|---|---|---|
| Pathway | Total | Expected | Hits | P.Value | FDR |
| Molecules associated with elastic fibres | 38 | 0.0797 | 6 | 6.53E-11 | 9.16E-08 |
| Elastic fibre formation | 45 | 0.0944 | 6 | 1.91E-10 | 1.34E-07 |
| Extracellular matrix organization | 157 | 0.329 | 7 | 1.04E-08 | 4.86E-06 |
| Lipoprotein metabolism | 22 | 0.0461 | 2 | 0.000921 | 0.132 |
| A tetrasaccharide linker sequence is required for GAG synthesis | 27 | 0.0566 | 2 | 0.00139 | 0.132 |
| Glycosaminoglycan metabolism | 118 | 0.247 | 3 | 0.0017 | 0.132 |
| MPS VI - Maroteaux-Lamy syndrome | 118 | 0.247 | 3 | 0.0017 | 0.132 |
| Mucopolysaccharidoses | 118 | 0.247 | 3 | 0.0017 | 0.132 |
| MPS IX - Natowicz syndrome | 118 | 0.247 | 3 | 0.0017 | 0.132 |
| MPS IIIB - Sanfilippo syndrome B | 118 | 0.247 | 3 | 0.0017 | 0.132 |
| MPS I - Hurler syndrome | 118 | 0.247 | 3 | 0.0017 | 0.132 |
| MPS II - Hunter syndrome | 118 | 0.247 | 3 | 0.0017 | 0.132 |
| MPS VII - Sly syndrome | 118 | 0.247 | 3 | 0.0017 | 0.132 |
| MPS IV - Morquio syndrome A | 118 | 0.247 | 3 | 0.0017 | 0.132 |
| MPS IIIC - Sanfilippo syndrome C | 118 | 0.247 | 3 | 0.0017 | 0.132 |
| MPS IV - Morquio syndrome B | 118 | 0.247 | 3 | 0.0017 | 0.132 |
| MPS IIIA - Sanfilippo syndrome A | 118 | 0.247 | 3 | 0.0017 | 0.132 |
| MPS IIID - Sanfilippo syndrome D | 118 | 0.247 | 3 | 0.0017 | 0.132 |
| Lipid digestion, mobilization, and transport | 41 | 0.086 | 2 | 0.0032 | 0.224 |
| Nuclear signaling by ERBB4 | 41 | 0.086 | 2 | 0.0032 | 0.224 |
| Signaling by ERBB4 | 152 | 0.319 | 3 | 0.0035 | 0.234 |
| Chondroitin sulfate/dermatan sulfate metabolism | 53 | 0.111 | 2 | 0.00529 | 0.337 |
| Heparan sulfate/heparin (HS-GAG) metabolism | 56 | 0.117 | 2 | 0.00589 | 0.359 |
| Disease | 945 | 1.98 | 6 | 0.00861 | 0.503 |
| FGFR1b ligand binding and activation | 5 | 0.0105 | 1 | 0.0104 | 0.586 |
| FGFR2b ligand binding and activation | 6 | 0.0126 | 1 | 0.0125 | 0.67 |
| Amyloids | 84 | 0.176 | 2 | 0.0129 | 0.67 |
| Constitutive Signaling by NOTCH1 t(7;9)(NOTCH1:M1580_K2555) Translocation Mutant | 7 | 0.0147 | 1 | 0.0146 | 0.705 |
| FGFR3b ligand binding and activation | 7 | 0.0147 | 1 | 0.0146 | 0.705 |

| | | | | | |
|---|---|---|---|---|---|
| Metabolism of carbohydrates | 258 | 0.541 | 3 | 0.0151 | 0.707 |
| Dermatan sulfate biosynthesis | 10 | 0.021 | 1 | 0.0208 | 0.793 |
| Crosslinking of collagen fibrils | 11 | 0.0231 | 1 | 0.0228 | 0.793 |
| FGFR1c ligand binding and activation | 11 | 0.0231 | 1 | 0.0228 | 0.793 |
| FGFR3c ligand binding and activation | 11 | 0.0231 | 1 | 0.0228 | 0.793 |
| FGFR3 ligand binding and activation | 11 | 0.0231 | 1 | 0.0228 | 0.793 |
| Signaling by activated point mutants of FGFR3 | 11 | 0.0231 | 1 | 0.0228 | 0.793 |
| Signaling by activated point mutants of FGFR1 | 11 | 0.0231 | 1 | 0.0228 | 0.793 |
| Signaling by FGFR3 mutants | 11 | 0.0231 | 1 | 0.0228 | 0.793 |
| Signaling by NOTCH3 | 11 | 0.0231 | 1 | 0.0228 | 0.793 |
| Signaling by NOTCH4 | 11 | 0.0231 | 1 | 0.0228 | 0.793 |
| Chylomicron-mediated lipid transport | 12 | 0.0252 | 1 | 0.0249 | 0.793 |
| FGFR2c ligand binding and activation | 12 | 0.0252 | 1 | 0.0249 | 0.793 |
| FGFR4 ligand binding and activation | 12 | 0.0252 | 1 | 0.0249 | 0.793 |
| Keratan sulfate degradation | 12 | 0.0252 | 1 | 0.0249 | 0.793 |
| HDL-mediated lipid transport | 13 | 0.0273 | 1 | 0.0269 | 0.829 |
| Receptor-ligand binding initiates the second proteolytic cleavage of Notch receptor | 14 | 0.0294 | 1 | 0.029 | 0.829 |
| FGFR1 ligand binding and activation | 14 | 0.0294 | 1 | 0.029 | 0.829 |
| Constitutive Signaling by NOTCH1 HD Domain Mutants | 14 | 0.0294 | 1 | 0.029 | 0.829 |
| CS/DS degradation | 14 | 0.0294 | 1 | 0.029 | 0.829 |
| Anchoring fibril formation | 15 | 0.0315 | 1 | 0.031 | 0.87 |
| FGFR2 ligand binding and activation | 16 | 0.0335 | 1 | 0.0331 | 0.875 |
| Signaling by FGFR2 mutants | 16 | 0.0335 | 1 | 0.0331 | 0.875 |
| Activated point mutants of FGFR2 | 16 | 0.0335 | 1 | 0.0331 | 0.875 |
| Prolactin receptor signaling | 17 | 0.0356 | 1 | 0.0351 | 0.911 |
| Signaling by NOTCH2 | 18 | 0.0377 | 1 | 0.0371 | 0.929 |
| NOTCH2 Activation and Transmission of Signal to the Nucleus | 18 | 0.0377 | 1 | 0.0371 | 0.929 |
| FGFR ligand binding and activation | 22 | 0.0461 | 1 | 0.0452 | 1 |
| HS-GAG degradation | 22 | 0.0461 | 1 | 0.0452 | 1 |
| Growth hormone receptor signaling | 23 | 0.0482 | 1 | 0.0472 | 1 |
| Chondroitin sulfate biosynthesis | 23 | 0.0482 | 1 | 0.0472 | 1 |

| MODULE 20 | | | | | |
|---|---|---|---|---|---|
| Pathway | Total | Expected | Hits | P.Value | FDR |
| Axon guidance | 292 | 0.7 | 9 | 4.52E-09 | 6.34E-06 |
| Developmental Biology | 417 | 0.999 | 9 | 1.03E-07 | 7.19E-05 |
| Signaling by Robo receptor | 34 | 0.0815 | 4 | 9.77E-07 | 0.000457 |
| Cell-extracellular matrix interactions | 19 | 0.0455 | 3 | 1.07E-05 | 0.00375 |
| Cell-Cell communication | 143 | 0.343 | 5 | 1.52E-05 | 0.00425 |
| Platelet degranulation | 89 | 0.213 | 4 | 4.75E-05 | 0.00951 |
| Cell junction organization | 89 | 0.213 | 4 | 4.75E-05 | 0.00951 |
| Response to elevated platelet cytosolic Ca2+ | 94 | 0.225 | 4 | 5.89E-05 | 0.0103 |
| Platelet activation, signaling and aggregation | 220 | 0.527 | 5 | 0.00012 | 0.0188 |
| Role of Abl in Robo-Slit signaling | 9 | 0.0216 | 2 | 0.000192 | 0.0269 |
| Regulation of actin dynamics for phagocytic cup formation | 62 | 0.149 | 3 | 0.000392 | 0.0499 |
| Semaphorin interactions | 72 | 0.173 | 3 | 0.000609 | 0.0711 |
| Fcgamma receptor (FCGR) dependent phagocytosis | 86 | 0.206 | 3 | 0.00102 | 0.11 |
| Sema4D induced cell migration and growth-cone collapse | 29 | 0.0695 | 2 | 0.0021 | 0.211 |
| Adherens junctions interactions | 31 | 0.0743 | 2 | 0.0024 | 0.225 |
| Sema4D in semaphorin signaling | 34 | 0.0815 | 2 | 0.00289 | 0.253 |
| Hemostasis | 511 | 1.22 | 5 | 0.00553 | 0.456 |
| Muscle contraction | 52 | 0.125 | 2 | 0.00666 | 0.519 |
| Cell-cell junction organization | 60 | 0.144 | 2 | 0.00879 | 0.648 |
| NOSTRIN mediated eNOS trafficking | 5 | 0.012 | 1 | 0.0119 | 0.836 |
| Regulation of cytoskeletal remodeling and cell spreading by IPP complex components | 9 | 0.0216 | 1 | 0.0214 | 1 |
| Formation of annular gap junctions | 11 | 0.0264 | 1 | 0.0261 | 1 |
| Gap junction degradation | 12 | 0.0288 | 1 | 0.0284 | 1 |
| DCC mediated attractive signaling | 14 | 0.0335 | 1 | 0.0331 | 1 |
| GRB2:SOS provides linkage to MAPK signaling for Intergrins | 15 | 0.0359 | 1 | 0.0354 | 1 |
| p130Cas linkage to MAPK signaling for integrins | 15 | 0.0359 | 1 | 0.0354 | 1 |
| Sema3A PAK dependent Axon repulsion | 16 | 0.0383 | 1 | 0.0377 | 1 |
| Metabolism of nitric oxide | 20 | 0.0479 | 1 | 0.0469 | 1 |
| eNOS activation and regulation | 20 | 0.0479 | 1 | 0.0469 | 1 |

| MODULE 21 | | | | | |
|---|---|---|---|---|---|
| Pathway | Total | Expected | Hits | P.Value | FDR |
| Integrin cell surface interactions | 85 | 0.0891 | 6 | 2.47E-11 | 3.46E-08 |
| Type I hemidesmosome assembly | 12 | 0.0126 | 2 | 6.19E-05 | 0.0434 |
| Anchoring fibril formation | 15 | 0.0157 | 2 | 9.83E-05 | 0.0459 |
| L1CAM interactions | 112 | 0.117 | 3 | 0.000153 | 0.0537 |
| Assembly of collagen fibrils and other multimeric structures | 54 | 0.0566 | 2 | 0.00131 | 0.333 |
| Signal Transduction | 1690 | 1.77 | 6 | 0.00142 | 0.333 |
| Axon guidance | 292 | 0.306 | 3 | 0.00254 | 0.509 |
| Collagen formation | 85 | 0.0891 | 2 | 0.00323 | 0.55 |
| Cell junction organization | 89 | 0.0933 | 2 | 0.00353 | 0.55 |
| Developmental Biology | 417 | 0.437 | 3 | 0.00701 | 0.982 |
| Cell-Cell communication | 143 | 0.15 | 2 | 0.00891 | 1 |
| Extracellular matrix organization | 157 | 0.165 | 2 | 0.0107 | 1 |
| Striated Muscle Contraction | 31 | 0.0325 | 1 | 0.0321 | 1 |

| MODULE 22 | | | | | |
|---|---|---|---|---|---|
| Pathway | Total | Expected | Hits | P.Value | FDR |
| Pyruvate metabolism and Citric Acid (TCA) cycle | 49 | 0.0954 | 6 | 1.88E-10 | 2.64E-07 |
| The citric acid (TCA) cycle and respiratory electron transport | 145 | 0.282 | 6 | 1.43E-07 | 7.01E-05 |
| Metabolism | 1490 | 2.9 | 12 | 1.50E-07 | 7.01E-05 |
| Pyruvate metabolism | 21 | 0.0409 | 3 | 7.52E-06 | 0.00263 |
| Citric acid cycle (TCA cycle) | 26 | 0.0506 | 3 | 1.46E-05 | 0.0041 |
| Glucose metabolism | 70 | 0.136 | 3 | 0.000293 | 0.0684 |
| Metabolism of carbohydrates | 258 | 0.502 | 4 | 0.00118 | 0.237 |
| Gluconeogenesis | 35 | 0.0681 | 2 | 0.00201 | 0.352 |
| Abnormal metabolism in phenylketonuria | 4 | 0.00779 | 1 | 0.00777 | 1 |
| Galactose catabolism | 5 | 0.00973 | 1 | 0.0097 | 1 |
| Glycogen synthesis | 8 | 0.0156 | 1 | 0.0155 | 1 |
| ChREBP activates metabolic gene expression | 8 | 0.0156 | 1 | 0.0155 | 1 |
| Pentose phosphate pathway (hexose monophosphate shunt) | 9 | 0.0175 | 1 | 0.0174 | 1 |
| Regulation of Gene Expression by Hypoxia-inducible Factor | 9 | 0.0175 | 1 | 0.0174 | 1 |
| Regulation of pyruvate dehydrogenase (PDH) complex | 13 | 0.0253 | 1 | 0.025 | 1 |
| Fatty acid, triacylglycerol, and ketone body metabolism | 139 | 0.271 | 2 | 0.0289 | 1 |
| Glycogen breakdown (glycogenolysis) | 18 | 0.035 | 1 | 0.0345 | 1 |
| Fatty Acyl-CoA Biosynthesis | 18 | 0.035 | 1 | 0.0345 | 1 |

| MODULE 23 | | | | | |
|---|---|---|---|---|---|
| Pathway | Total | Expected | Hits | P.Value | FDR |
| Regulation of Insulin Secretion by Free Fatty Acids | 8 | 0.0144 | 2 | 8.24E-05 | 0.0578 |
| Regulation of Insulin Secretion by Fatty Acids Bound to GPR40 (FFAR1) | 8 | 0.0144 | 2 | 8.24E-05 | 0.0578 |
| Regulation of Insulin Secretion by Acetylcholine | 11 | 0.0198 | 2 | 0.000161 | 0.0754 |
| Transport of inorganic cations/anions and amino acids/oligopeptides | 96 | 0.173 | 3 | 0.000577 | 0.202 |
| Thromboxane signalling through TP receptor | 25 | 0.0449 | 2 | 0.000868 | 0.237 |
| ADP signalling through P2Y purinoceptor 1 | 27 | 0.0485 | 2 | 0.00101 | 0.237 |
| Signal amplification | 33 | 0.0593 | 2 | 0.00152 | 0.282 |
| Thrombin signalling through proteinase activated receptors (PARs) | 34 | 0.0611 | 2 | 0.00161 | 0.282 |
| Type II Na+/Pi cotransporters | 4 | 0.00719 | 1 | 0.00717 | 0.866 |
| SLC-mediated transmembrane transport | 251 | 0.451 | 3 | 0.00897 | 0.866 |
| Regulation of Insulin Secretion | 82 | 0.147 | 2 | 0.00908 | 0.866 |
| Constitutive PI3K/AKT Signaling in Cancer | 89 | 0.16 | 2 | 0.0106 | 0.866 |
| Sodium-coupled phosphate cotransporters | 6 | 0.0108 | 1 | 0.0107 | 0.866 |
| The retinoid cycle in cones (daylight vision) | 7 | 0.0126 | 1 | 0.0125 | 0.866 |
| PI3K events in ERBB4 signaling | 103 | 0.185 | 2 | 0.0141 | 0.866 |
| PIP3 activates AKT signaling | 103 | 0.185 | 2 | 0.0141 | 0.866 |
| PI-3K cascade | 103 | 0.185 | 2 | 0.0141 | 0.866 |
| PI3K/AKT Signaling in Cancer | 103 | 0.185 | 2 | 0.0141 | 0.866 |
| PI3K events in ERBB2 signaling | 103 | 0.185 | 2 | 0.0141 | 0.866 |
| Adrenoceptors | 8 | 0.0144 | 1 | 0.0143 | 0.866 |
| GAB1 signalosome | 106 | 0.191 | 2 | 0.0149 | 0.866 |
| PI3K/AKT activation | 106 | 0.191 | 2 | 0.0149 | 0.866 |
| Signal Transduction | 1690 | 3.03 | 7 | 0.0151 | 0.866 |
| Integration of energy metabolism | 109 | 0.196 | 2 | 0.0157 | 0.866 |
| Bicarbonate transporters | 9 | 0.0162 | 1 | 0.0161 | 0.866 |
| Sodium/Proton exchangers | 9 | 0.0162 | 1 | 0.0161 | 0.866 |
| Retinoid cycle disease events | 10 | 0.018 | 1 | 0.0178 | 0.926 |
| Negative regulation of the PI3K/AKT network | 11 | 0.0198 | 1 | 0.0196 | 0.982 |
| G alpha (s) signalling events | 127 | 0.228 | 2 | 0.0209 | 1 |

| | | | | | |
|---|---|---|---|---|---|
| Role of second messengers in netrin-1 signaling | 13 | 0.0234 | 1 | 0.0231 | 1 |
| Signaling by SCF-KIT | 142 | 0.255 | 2 | 0.0258 | 1 |
| The canonical retinoid cycle in rods (twilight vision) | 15 | 0.027 | 1 | 0.0266 | 1 |
| Downstream signaling of activated FGFR | 150 | 0.27 | 2 | 0.0285 | 1 |
| Signaling by ERBB4 | 152 | 0.273 | 2 | 0.0293 | 1 |
| Signaling by FGFR | 162 | 0.291 | 2 | 0.0329 | 1 |
| Downstream signal transduction | 163 | 0.293 | 2 | 0.0333 | 1 |
| Signaling by ERBB2 | 164 | 0.295 | 2 | 0.0337 | 1 |
| DAP12 signaling | 164 | 0.295 | 2 | 0.0337 | 1 |
| Downstream Signaling Events Of B Cell Receptor (BCR) | 173 | 0.311 | 2 | 0.0371 | 1 |
| Signaling by FGFR in disease | 178 | 0.32 | 2 | 0.0391 | 1 |
| Signaling by EGFR | 179 | 0.322 | 2 | 0.0395 | 1 |
| Signaling by EGFR in Cancer | 181 | 0.325 | 2 | 0.0403 | 1 |
| DAP12 interactions | 182 | 0.327 | 2 | 0.0408 | 1 |
| G alpha (q) signalling events | 188 | 0.338 | 2 | 0.0432 | 1 |
| Signaling by PDGF | 189 | 0.34 | 2 | 0.0437 | 1 |
| Synthesis of IP3 and IP4 in the cytosol | 27 | 0.0485 | 1 | 0.0475 | 1 |
| Signaling by the B Cell Receptor (BCR) | 199 | 0.358 | 2 | 0.0479 | 1 |
| GPCR downstream signaling | 812 | 1.46 | 4 | 0.0483 | 1 |

| MODULE 24 | | | | | |
|---|---|---|---|---|---|
| Pathway | Total | Expected | Hits | P.Value | FDR |
| NCAM signaling for neurite out-growth | 65 | 0.146 | 8 | 3.16E-13 | 4.42E-10 |
| Interaction between L1 and Ankyrins | 29 | 0.0651 | 6 | 1.89E-11 | 1.32E-08 |
| L1CAM interactions | 112 | 0.252 | 8 | 2.84E-11 | 1.33E-08 |
| Axon guidance | 292 | 0.656 | 9 | 2.06E-09 | 7.21E-07 |
| Developmental Biology | 417 | 0.937 | 9 | 4.75E-08 | 1.33E-05 |
| Interferon gamma signaling | 74 | 0.166 | 5 | 4.02E-07 | 9.39E-05 |
| Interferon Signaling | 173 | 0.389 | 6 | 1.14E-06 | 0.000229 |
| Interferon alpha/beta signaling | 68 | 0.153 | 4 | 1.23E-05 | 0.00216 |
| Cytokine Signaling in Immune system | 286 | 0.643 | 6 | 2.12E-05 | 0.00329 |
| Immune System | 1140 | 2.56 | 10 | 2.61E-05 | 0.00367 |
| Signaling by FGFR mutants | 45 | 0.101 | 3 | 0.000123 | 0.0157 |
| Phospholipase C-mediated cascade | 57 | 0.128 | 3 | 0.00025 | 0.0292 |
| Downstream signaling of activated FGFR | 150 | 0.337 | 4 | 0.000275 | 0.0297 |
| Signaling by FGFR | 162 | 0.364 | 4 | 0.00037 | 0.032 |
| Downstream signal transduction | 163 | 0.366 | 4 | 0.000379 | 0.032 |
| Signaling by ERBB2 | 164 | 0.368 | 4 | 0.000388 | 0.032 |
| DAP12 signaling | 164 | 0.368 | 4 | 0.000388 | 0.032 |
| Signaling by FGFR in disease | 178 | 0.4 | 4 | 0.000529 | 0.0385 |
| Signaling by EGFR | 179 | 0.402 | 4 | 0.000541 | 0.0385 |
| Signaling by EGFR in Cancer | 181 | 0.407 | 4 | 0.000564 | 0.0385 |
| DAP12 interactions | 182 | 0.409 | 4 | 0.000576 | 0.0385 |
| Signaling by PDGF | 189 | 0.425 | 4 | 0.000664 | 0.0423 |
| Signaling by the B Cell Receptor (BCR) | 199 | 0.447 | 4 | 0.000807 | 0.0492 |
| Constitutive PI3K/AKT Signaling in Cancer | 89 | 0.2 | 3 | 0.000928 | 0.0525 |
| NGF signalling via TRKA from the plasma membrane | 207 | 0.465 | 4 | 0.000936 | 0.0525 |
| FGFR ligand binding and activation | 22 | 0.0494 | 2 | 0.00106 | 0.0572 |
| Nephrin interactions | 24 | 0.0539 | 2 | 0.00126 | 0.0621 |
| PI3K events in ERBB4 signaling | 103 | 0.231 | 3 | 0.00142 | 0.0621 |
| PIP3 activates AKT signaling | 103 | 0.231 | 3 | 0.00142 | 0.0621 |

| | | | | | |
|---|---|---|---|---|---|
| PI-3K cascade | 103 | 0.231 | 3 | 0.00142 | 0.0621 |
| PI3K/AKT Signaling in Cancer | 103 | 0.231 | 3 | 0.00142 | 0.0621 |
| PI3K events in ERBB2 signaling | 103 | 0.231 | 3 | 0.00142 | 0.0621 |
| GAB1 signalosome | 106 | 0.238 | 3 | 0.00154 | 0.0635 |
| PI3K/AKT activation | 106 | 0.238 | 3 | 0.00154 | 0.0635 |
| SHC-mediated cascade | 28 | 0.0629 | 2 | 0.00172 | 0.069 |
| Signaling by FGFR1 mutants | 31 | 0.0696 | 2 | 0.00211 | 0.0822 |
| Signaling by FGFR1 amplification mutants | 1 | 0.00225 | 1 | 0.00225 | 0.0829 |
| Signaling by FGFR2 amplification mutants | 1 | 0.00225 | 1 | 0.00225 | 0.0829 |
| Signal transduction by L1 | 35 | 0.0786 | 2 | 0.00269 | 0.0965 |
| Signalling by NGF | 290 | 0.651 | 4 | 0.00325 | 0.114 |
| FRS2-mediated cascade | 39 | 0.0876 | 2 | 0.00333 | 0.114 |
| Signaling by SCF-KIT | 142 | 0.319 | 3 | 0.00355 | 0.119 |
| Negative regulation of FGFR signaling | 41 | 0.0921 | 2 | 0.00367 | 0.12 |
| Signaling by ERBB4 | 152 | 0.341 | 3 | 0.0043 | 0.137 |
| Downstream Signaling Events Of B Cell Receptor (BCR) | 173 | 0.389 | 3 | 0.00618 | 0.193 |
| FGFR1c and Klotho ligand binding and activation | 3 | 0.00674 | 1 | 0.00673 | 0.205 |
| Adaptive Immune System | 654 | 1.47 | 5 | 0.0116 | 0.345 |
| PI3K Cascade | 76 | 0.171 | 2 | 0.0122 | 0.356 |
| FGFR2b ligand binding and activation | 6 | 0.0135 | 1 | 0.0134 | 0.376 |
| Klotho-mediated ligand binding | 6 | 0.0135 | 1 | 0.0134 | 0.376 |
| IRS-mediated signalling | 87 | 0.195 | 2 | 0.0158 | 0.434 |
| IRS-related events | 90 | 0.202 | 2 | 0.0168 | 0.454 |
| IRS-related events triggered by IGF1R | 91 | 0.204 | 2 | 0.0172 | 0.455 |
| Signaling by Type 1 Insulin-like Growth Factor 1 Receptor (IGF1R) | 94 | 0.211 | 2 | 0.0183 | 0.466 |
| IGF1R signaling cascade | 94 | 0.211 | 2 | 0.0183 | 0.466 |
| Insulin receptor signalling cascade | 95 | 0.213 | 2 | 0.0186 | 0.467 |
| FGFR1c ligand binding and activation | 11 | 0.0247 | 1 | 0.0245 | 0.591 |
| Signaling by activated point mutants of FGFR1 | 11 | 0.0247 | 1 | 0.0245 | 0.591 |
| Innate Immune System | 521 | 1.17 | 4 | 0.025 | 0.594 |
| FGFR2c ligand binding and activation | 12 | 0.027 | 1 | 0.0266 | 0.623 |

| | | | | | |
|---|---|---|---|---|---|
| Signaling by Insulin receptor | 117 | 0.263 | 2 | 0.0275 | 0.632 |
| PECAM1 interactions | 13 | 0.0292 | 1 | 0.0288 | 0.632 |
| Caspase-mediated cleavage of cytoskeletal proteins | 13 | 0.0292 | 1 | 0.0288 | 0.632 |
| Role of second messengers in netrin-1 signaling | 13 | 0.0292 | 1 | 0.0288 | 0.632 |
| FGFR1 ligand binding and activation | 14 | 0.0315 | 1 | 0.031 | 0.669 |
| FGFR2 ligand binding and activation | 16 | 0.0359 | 1 | 0.0354 | 0.73 |
| Signaling by FGFR2 mutants | 16 | 0.0359 | 1 | 0.0354 | 0.73 |
| Activated point mutants of FGFR2 | 16 | 0.0359 | 1 | 0.0354 | 0.73 |
| Regulation of KIT signaling | 17 | 0.0382 | 1 | 0.0376 | 0.763 |
| Frs2-mediated activation | 18 | 0.0404 | 1 | 0.0397 | 0.787 |
| Cell-Cell communication | 143 | 0.321 | 2 | 0.0399 | 0.787 |
| Signaling by constitutively active EGFR | 19 | 0.0427 | 1 | 0.0419 | 0.816 |
| Prolonged ERK activation events | 20 | 0.0449 | 1 | 0.044 | 0.834 |
| Signaling by FGFR1 fusion mutants | 20 | 0.0449 | 1 | 0.044 | 0.834 |

| MODULE 25 | | | | | |
|---|---|---|---|---|---|
| Pathway | Total | Expected | Hits | P.Value | FDR |
| Interleukin-6 signaling | 14 | 0.0126 | 6 | 2.45E-17 | 3.43E-14 |
| Signaling by Interleukins | 116 | 0.104 | 6 | 2.42E-11 | 1.69E-08 |
| Cytokine Signaling in Immune system | 286 | 0.257 | 6 | 5.87E-09 | 2.74E-06 |
| Regulation of IFNG signaling | 17 | 0.0153 | 3 | 2.73E-07 | 9.57E-05 |
| Regulation of IFNA signaling | 27 | 0.0243 | 3 | 1.17E-06 | 0.000328 |
| Interferon alpha/beta signaling | 68 | 0.0611 | 3 | 1.98E-05 | 0.00447 |
| Immune System | 1140 | 1.02 | 6 | 2.42E-05 | 0.00447 |
| Interferon gamma signaling | 74 | 0.0665 | 3 | 2.55E-05 | 0.00447 |
| Growth hormone receptor signaling | 23 | 0.0207 | 2 | 0.000169 | 0.0263 |
| Interferon Signaling | 173 | 0.155 | 3 | 0.000323 | 0.0453 |
| Antiviral mechanism by IFN-stimulated genes | 69 | 0.062 | 2 | 0.00154 | 0.18 |
| ISG15 antiviral mechanism | 69 | 0.062 | 2 | 0.00154 | 0.18 |
| Interleukin-7 signaling | 12 | 0.0108 | 1 | 0.0107 | 1 |
| Signaling by FGFR1 fusion mutants | 20 | 0.018 | 1 | 0.0178 | 1 |
| Interleukin receptor SHC signaling | 28 | 0.0252 | 1 | 0.0249 | 1 |
| Signaling by FGFR1 mutants | 31 | 0.0279 | 1 | 0.0275 | 1 |
| Interleukin-2 signaling | 42 | 0.0377 | 1 | 0.0372 | 1 |
| Signaling by FGFR mutants | 45 | 0.0404 | 1 | 0.0398 | 1 |
| Interleukin-3, 5 and GM-CSF signaling | 51 | 0.0458 | 1 | 0.045 | 1 |

| MODULE 26 | | | | | |
|---|---|---|---|---|---|
| Pathway | Total | Expected | Hits | P.Value | FDR |
| Antigen Presentation: Folding, assembly and peptide loading of class I MHC | 25 | 0.0412 | 7 | 1.34E-15 | 1.88E-12 |
| Immunoregulatory interactions between a Lymphoid and a non-Lymphoid cell | 80 | 0.132 | 8 | 4.76E-14 | 3.34E-11 |
| Endosomal/Vacuolar pathway | 10 | 0.0165 | 5 | 1.05E-12 | 4.91E-10 |
| Adaptive Immune System | 654 | 1.08 | 10 | 7.66E-10 | 2.69E-07 |
| ER-Phagosome pathway | 63 | 0.104 | 5 | 2.82E-08 | 7.89E-06 |
| Class I MHC mediated antigen processing & presentation | 267 | 0.44 | 7 | 4.35E-08 | 1.02E-05 |
| Interferon gamma signaling | 74 | 0.122 | 5 | 6.40E-08 | 1.28E-05 |
| Antigen processing-Cross presentation | 78 | 0.129 | 5 | 8.36E-08 | 1.47E-05 |
| Immune System | 1140 | 1.87 | 10 | 1.86E-07 | 2.90E-05 |
| Interferon alpha/beta signaling | 68 | 0.112 | 4 | 3.08E-06 | 0.000431 |
| Interferon Signaling | 173 | 0.285 | 5 | 4.49E-06 | 0.000572 |
| Nef-mediates down modulation of cell surface receptors by recruiting them to clathrin adapters | 24 | 0.0395 | 3 | 6.61E-06 | 0.000772 |
| The role of Nef in HIV-1 replication and disease pathogenesis | 31 | 0.0511 | 3 | 1.46E-05 | 0.00157 |
| Cytokine Signaling in Immune system | 286 | 0.471 | 5 | 5.20E-05 | 0.00521 |
| COPII (Coat Protein 2) Mediated Vesicle Transport | 9 | 0.0148 | 2 | 8.83E-05 | 0.00774 |
| ER to Golgi Transport | 9 | 0.0148 | 2 | 8.83E-05 | 0.00774 |
| Nef mediated downregulation of MHC class I complex cell surface expression | 13 | 0.0214 | 2 | 0.000191 | 0.0157 |
| Host Interactions of HIV factors | 141 | 0.232 | 3 | 0.00134 | 0.105 |
| Transport to the Golgi and subsequent modification | 35 | 0.0577 | 2 | 0.00143 | 0.105 |
| HIV Infection | 214 | 0.353 | 3 | 0.00443 | 0.31 |
| Asparagine N-linked glycosylation | 86 | 0.142 | 2 | 0.00836 | 0.558 |
| Nef Mediated CD8 Down-regulation | 7 | 0.0115 | 1 | 0.0115 | 0.732 |
| MHC class II antigen presentation | 118 | 0.194 | 2 | 0.0153 | 0.936 |
| Crosslinking of collagen fibrils | 11 | 0.0181 | 1 | 0.018 | 1 |
| DAP12 signaling | 164 | 0.27 | 2 | 0.0285 | 1 |
| DAP12 interactions | 182 | 0.3 | 2 | 0.0346 | 1 |
| Post-translational protein modification | 200 | 0.329 | 2 | 0.0411 | 1 |
| Membrane Trafficking | 203 | 0.334 | 2 | 0.0422 | 1 |

| MODULE 27 | | | | | |
|---|---|---|---|---|---|
| Pathway | Total | Expected | Hits | P.Value | FDR |
| Regulation of Complement cascade | 15 | 0.0157 | 5 | 5.70E-13 | 7.99E-10 |
| Complement cascade | 33 | 0.0346 | 5 | 4.48E-11 | 3.14E-08 |
| Alternative complement activation | 6 | 0.00629 | 3 | 1.41E-08 | 6.59E-06 |
| Activation of C3 and C5 | 7 | 0.00734 | 3 | 2.47E-08 | 8.64E-06 |
| Initial triggering of complement | 17 | 0.0178 | 3 | 4.77E-07 | 0.000134 |
| Innate Immune System | 521 | 0.546 | 5 | 5.22E-05 | 0.0122 |
| Immune System | 1140 | 1.19 | 5 | 0.00221 | 0.443 |
| G alpha (i) signalling events | 184 | 0.193 | 2 | 0.0145 | 1 |
| Peptide ligand-binding receptors | 192 | 0.201 | 2 | 0.0157 | 1 |
| Class A/1 (Rhodopsin-like receptors) | 312 | 0.327 | 2 | 0.0391 | 1 |

| MODULE 28 | | | | | |
|---|---|---|---|---|---|
| Pathway | Total | Expected | Hits | P.Value | FDR |
| Striated Muscle Contraction | 31 | 0.0371 | 6 | 1.67E-13 | 2.34E-10 |
| Muscle contraction | 52 | 0.0623 | 6 | 4.59E-12 | 3.22E-09 |
| NRAGE signals death through JNK | 45 | 0.0539 | 1 | 0.0527 | 1 |
| Cell death signalling via NRAGE, NRIF and NADE | 62 | 0.0743 | 1 | 0.072 | 1 |
| G alpha (12/13) signalling events | 80 | 0.0959 | 1 | 0.092 | 1 |
| p75 NTR receptor-mediated signalling | 85 | 0.102 | 1 | 0.0975 | 1 |
| Rho GTPase cycle | 123 | 0.147 | 1 | 0.138 | 1 |
| Signaling by Rho GTPases | 123 | 0.147 | 1 | 0.138 | 1 |
| Antigen processing: Ubiquitination & Proteasome degradation | 224 | 0.268 | 1 | 0.239 | 1 |
| Class I MHC mediated antigen processing & presentation | 267 | 0.32 | 1 | 0.279 | 1 |

| MODULE 29 | | | | | |
|---|---|---|---|---|---|
| Pathway | Total | Expected | Hits | P.Value | FDR |
| Metabolism of Angiotensinogen to Angiotensins | 14 | 0.0252 | 9 | 6.08E-24 | 8.52E-21 |
| Peptide hormone metabolism | 60 | 0.108 | 9 | 4.41E-17 | 3.09E-14 |
| Metabolism of proteins | 689 | 1.24 | 9 | 2.09E-07 | 9.75E-05 |
| Peptide ligand-binding receptors | 192 | 0.345 | 3 | 0.00425 | 1 |
| Class A/1 (Rhodopsin-like receptors) | 312 | 0.561 | 3 | 0.0162 | 1 |
| Activation of the AP-1 family of transcription factors | 10 | 0.018 | 1 | 0.0178 | 1 |
| GPCR ligand binding | 415 | 0.746 | 3 | 0.0344 | 1 |
| G alpha (i) signalling events | 184 | 0.331 | 2 | 0.0416 | 1 |
| G alpha (q) signalling events | 188 | 0.338 | 2 | 0.0432 | 1 |
| Lysosome Vesicle Biogenesis | 27 | 0.0485 | 1 | 0.0475 | 1 |

| MODULE 30 | | | | | |
|---|---|---|---|---|---|
| Pathway | Total | Expected | Hits | P.Value | FDR |
| Scavenging of Heme from Plasma | 15 | 0.0157 | 5 | 5.70E-13 | 3.99E-10 |
| Binding and Uptake of Ligands by Scavenger Receptors | 15 | 0.0157 | 5 | 5.70E-13 | 3.99E-10 |
| Uptake of Oxygen and Release of Carbon Dioxide by Erythrocytes | 10 | 0.0105 | 3 | 8.44E-08 | 2.37E-05 |
| Uptake of Carbon Dioxide and Release of Oxygen by Erythrocytes | 10 | 0.0105 | 3 | 8.44E-08 | 2.37E-05 |
| O2/CO2 exchange in erythrocytes | 10 | 0.0105 | 3 | 8.44E-08 | 2.37E-05 |
| Factors involved in megakaryocyte development and platelet production | 155 | 0.162 | 3 | 0.000401 | 0.0937 |
| Hemostasis | 511 | 0.536 | 3 | 0.0124 | 1 |
| Metabolism | 1490 | 1.56 | 3 | 0.189 | 1 |

| MODULE 31 | | | | | |
|---|---|---|---|---|---|
| Pathway | Total | Expected | Hits | P.Value | FDR |
| Activation of Matrix Metalloproteinases | 31 | 0.0511 | 10 | 1.01E-23 | 1.41E-20 |
| Degradation of the extracellular matrix | 77 | 0.127 | 10 | 2.48E-19 | 1.74E-16 |
| Extracellular matrix organization | 157 | 0.259 | 10 | 4.18E-16 | 1.96E-13 |
| Degradation of collagen | 61 | 0.1 | 7 | 1.20E-12 | 4.19E-10 |
| Assembly of collagen fibrils and other multimeric structures | 54 | 0.089 | 2 | 0.00337 | 0.945 |
| Collagen formation | 85 | 0.14 | 2 | 0.00818 | 1 |
| Platelet degranulation | 89 | 0.147 | 2 | 0.00894 | 1 |
| Response to elevated platelet cytosolic Ca2+ | 94 | 0.155 | 2 | 0.00993 | 1 |
| EGFR Transactivation by Gastrin | 9 | 0.0148 | 1 | 0.0147 | 1 |
| Regulation of Insulin-like Growth Factor (IGF) Transport and Uptake by Insulin-like Growth Factor Binding Proteins (IGFBPs) | 21 | 0.0346 | 1 | 0.0341 | 1 |
| Hemostasis | 511 | 0.842 | 3 | 0.0463 | 1 |
| Platelet activation, signaling and aggregation | 220 | 0.362 | 2 | 0.0489 | 1 |

| MODULE 32 | | | | | |
|---|---|---|---|---|---|
| Pathway | Total | Expected | Hits | P.Value | FDR |
| Formation of Fibrin Clot (Clotting Cascade) | 29 | 0.0347 | 7 | 1.06E-16 | 1.49E-13 |
| Extrinsic Pathway | 5 | 0.00599 | 5 | 5.07E-16 | 3.55E-13 |
| Gamma-carboxylation of protein precursors | 10 | 0.012 | 4 | 1.77E-10 | 8.28E-08 |
| Gamma-carboxylation, transport, and amino-terminal cleavage of proteins | 12 | 0.0144 | 4 | 4.17E-10 | 1.46E-07 |
| PTM: gamma carboxylation, hypusine formation and arylsulfatase activation | 29 | 0.0347 | 4 | 1.99E-08 | 5.57E-06 |
| Transport of gamma-carboxylated protein precursors from the endoplasmic reticulum to the Golgi apparatus | 9 | 0.0108 | 3 | 9.45E-08 | 2.21E-05 |
| Hemostasis | 511 | 0.612 | 7 | 1.11E-07 | 2.21E-05 |
| Removal of aminoterminal propeptides from gamma-carboxylated proteins | 10 | 0.012 | 3 | 1.35E-07 | 2.37E-05 |
| Common Pathway | 11 | 0.0132 | 3 | 1.85E-07 | 2.89E-05 |
| Post-translational protein modification | 200 | 0.24 | 4 | 4.98E-05 | 0.00698 |
| Intrinsic Pathway | 17 | 0.0204 | 2 | 0.000169 | 0.0216 |
| Metabolism of proteins | 689 | 0.826 | 4 | 0.0056 | 0.654 |
| BMAL1:CLOCK/NPAS2 Activates Circadian Expression | 14 | 0.0168 | 1 | 0.0167 | 1 |
| Circadian Clock | 35 | 0.0419 | 1 | 0.0412 | 1 |

| MODULE 33 | | | | | |
|---|---|---|---|---|---|
| Pathway | Total | Expected | Hits | P.Value | FDR |
| Glycoprotein hormones | 7 | 0.0105 | 7 | 1.03E-21 | 1.44E-18 |
| Peptide hormone biosynthesis | 9 | 0.0135 | 7 | 3.69E-20 | 2.59E-17 |
| Peptide hormone metabolism | 60 | 0.0899 | 7 | 3.88E-13 | 1.81E-10 |
| Hormone ligand-binding receptors | 10 | 0.015 | 5 | 5.73E-13 | 2.01E-10 |
| G alpha (s) signalling events | 127 | 0.19 | 5 | 5.37E-07 | 0.000151 |
| Metabolism of proteins | 689 | 1.03 | 7 | 1.10E-05 | 0.00257 |
| Class A/1 (Rhodopsin-like receptors) | 312 | 0.467 | 5 | 4.48E-05 | 0.00897 |
| GPCR ligand binding | 415 | 0.622 | 5 | 0.000176 | 0.0308 |
| Signal regulatory protein (SIRP) family interactions | 20 | 0.03 | 2 | 0.000378 | 0.0589 |
| GPCR downstream signaling | 812 | 1.22 | 5 | 0.00391 | 0.548 |
| Signaling by GPCR | 931 | 1.39 | 5 | 0.00715 | 0.912 |
| Cell surface interactions at the vascular wall | 99 | 0.148 | 2 | 0.00906 | 1 |
| Cell-Cell communication | 143 | 0.214 | 2 | 0.0183 | 1 |

| MODULE 34 | | | | | |
|---|---|---|---|---|---|
| Pathway | Total | Expected | Hits | P.Value | FDR |
| Utilization of Ketone Bodies | 3 | 0.0018 | 1 | 0.0018 | 1 |
| Ketone body metabolism | 5 | 0.003 | 1 | 0.00299 | 1 |
| Attachment of GPI anchor to uPAR | 7 | 0.00419 | 1 | 0.00419 | 1 |
| Dissolution of Fibrin Clot | 10 | 0.00599 | 1 | 0.00598 | 1 |
| Interleukin-6 signaling | 14 | 0.00839 | 1 | 0.00836 | 1 |
| Post-translational modification: synthesis of GPI-anchored proteins | 26 | 0.0156 | 1 | 0.0155 | 1 |
| Regulation of IFNA signaling | 27 | 0.0162 | 1 | 0.0161 | 1 |
| Golgi Associated Vesicle Biogenesis | 57 | 0.0341 | 1 | 0.0337 | 1 |
| trans-Golgi Network Vesicle Budding | 64 | 0.0383 | 1 | 0.0378 | 1 |
| Clathrin derived vesicle budding | 64 | 0.0383 | 1 | 0.0378 | 1 |
| Interferon alpha/beta signaling | 68 | 0.0407 | 1 | 0.0401 | 1 |
